# Amino Acid Modified RNA Bases as Building Blocks of an Early Earth RNA‐Peptide World

**DOI:** 10.1002/chem.202002929

**Published:** 2020-10-14

**Authors:** Milda Nainytė, Felix Müller, Giacomo Ganazzoli, Chun‐Yin Chan, Antony Crisp, Daniel Globisch, Thomas Carell

**Affiliations:** ^1^ Department of Chemistry LMU München Butenandtstr. 5–13 81377 München Germany; ^2^ Department of Medicinal Chemistry Uppsala University Husargatan 3 75123 Uppsala Sweden

**Keywords:** amino acid nucleosides, origin of life, prebiotic chemistry, RNA world, RNA-peptide world

## Abstract

Fossils of extinct species allow us to reconstruct the process of Darwinian evolution that led to the species diversity we see on Earth today. The origin of the first functional molecules able to undergo molecular evolution and thus eventually able to create life, are largely unknown. The most prominent idea in the field posits that biology was preceded by an era of molecular evolution, in which RNA molecules encoded information and catalysed their own replication. This RNA world concept stands against other hypotheses, that argue for example that life may have begun with catalytic peptides and primitive metabolic cycles. The question whether RNA or peptides were first is addressed by the RNA‐peptide world concept, which postulates a parallel existence of both molecular species. A plausible experimental model of how such an RNA‐peptide world may have looked like, however, is absent. Here we report the synthesis and physicochemical evaluation of amino acid containing adenosine bases, which are closely related to molecules that are found today in the anticodon stem‐loop of tRNAs from all three kingdoms of life. We show that these adenosines lose their base pairing properties, which allow them to equip RNA with amino acids independent of the sequence context. As such we may consider them to be living molecular fossils of an extinct molecular RNA‐peptide world.

## Graphical abstract

**Naturally occurring amino acid** containing nucleobases could be vestiges of an extinct early Earth RNA‐peptide world. Today they help to translate genetic information, but in the RNA‐peptide world, they might have been the units that equipped RNA with broad catalytic functions. These genotype‐phenotype bridging nucleosides were synthesized, and their properties were investigated. This study shows that these nucleosides do not base pair, which allows them to position amino acids in unpaired parts of complex RNA structures.

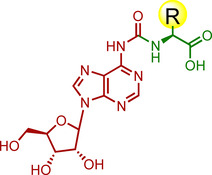

The RNA‐peptide co‐evolution hypothesis describes the emergence of self‐replicating molecules that contained amino acids and RNA.^[1]^ At the macromolecular level, this tight coexistence of peptides and RNA is established in the ribosome, where encoding and catalytic RNA is supported by proteins.^[2]^ Although we cannot delineate how such an early RNA‐peptide world may have looked like, it seems not too implausible to assume that some of the molecular components may have survived until today as vestiges of this extinct world.^[3]^ tRNAs derived from all three kingdoms of life contain a large number of modified bases,^[4]^ and some of them are indeed modified with amino acids.^[3]^ The most wide spread amino acid modified bases are adenosine nucleosides, in which the amino acid is linked via urea connector to the *N* ^6^‐amino group of the heterocycle as depicted in Figure 1 a. Particularly ubiquitous are adenosine modifications containing the amino acids threonine (t^6^A)[^5^, ^6^, ^7^] and glycine (g^6^A),^[8]^ together with hn^6^A.[^9^, ^10^] Based upon recent phylogenetic analyses and the fact that t^6^A is found in all three kingdoms of life, it has been suggested that such amino acid modified bases were already present in the last universal common ancestor (LUCA), from which all life forms descended.[^11^, ^12^, ^13^, ^14^] t^6^A is for example today found in nearly all ANN decoding tRNAs.^[15]^ We recently reported a plausible prebiotic route to some of these amino acid modified A‐bases, which strengthens the idea that they could indeed be living chemical fossils of the extinct RNA‐peptide world.^[16]^ Despite the interesting philosophical genotype‐phenotype dualism that characterizes these structures and their contemporary importance for the faithful decoding of genetic information, a general synthesis of aa^6^A modified bases (Figure 1 a) and a systematic study of their properties is lacking.

**Figure 1:**
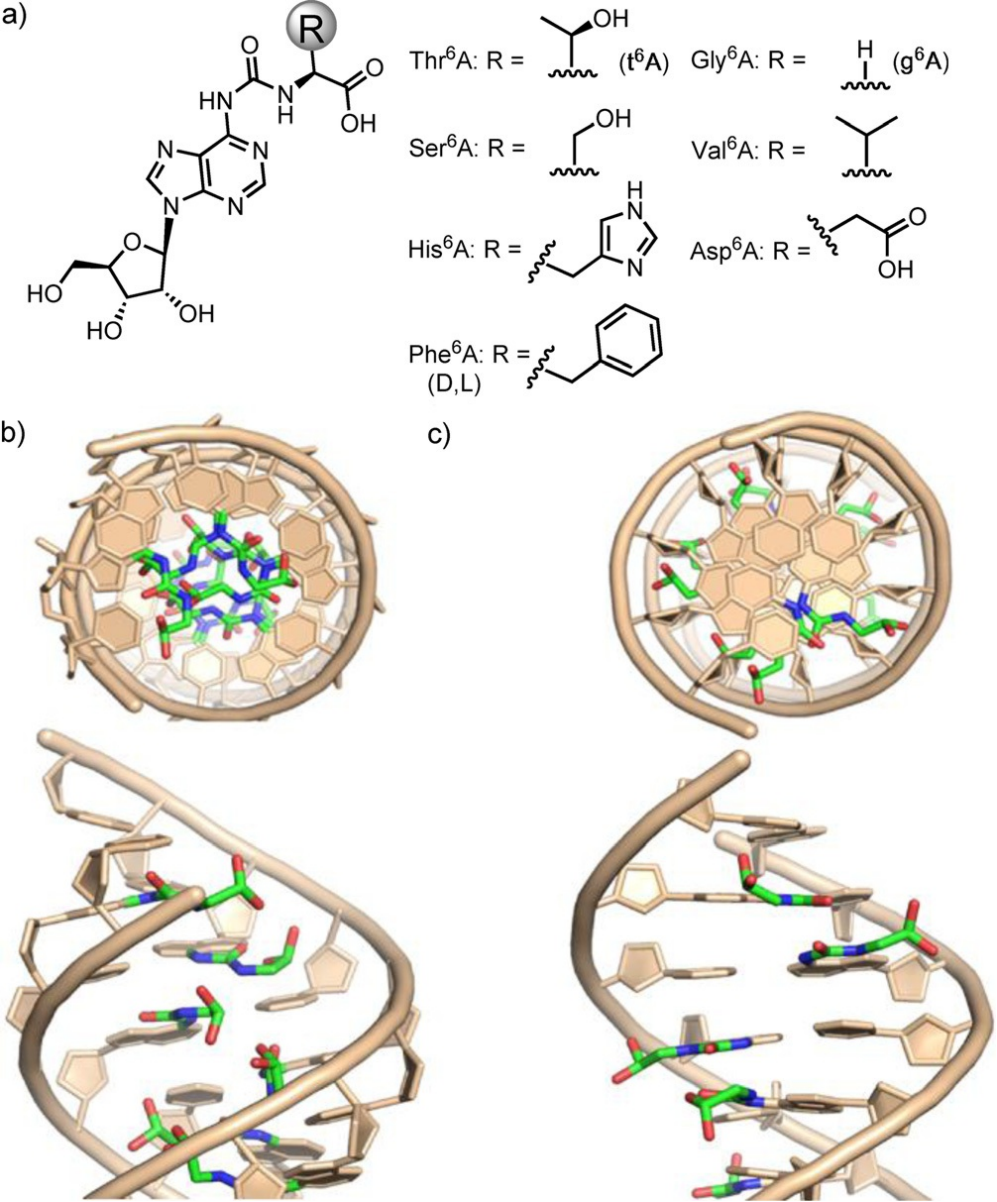
(a) Depiction of the amino acid modified A‐bases (aa^6^A) together with computer visualizations that show how such bases may reside in an (b) A‐form RNA duplex and a (c) B‐form DNA duplex. The sequence used for the visualization is: 5′‐CAUAUAUAUAUG‐3′ with A=g^6^A.

Here we report the synthesis of a variety of aa^6^A nucleosides with canonical amino acids (aa=Asp, Gly, His, Phe, Thr,^[17]^ Ser, Val), their incorporation into DNA and RNA and an investigation of how they influence the physicochemical properties of oligonucleotides. We were particularly interested to study how they might affect the stability of RNA and DNA. The computer visualization shows that in A‐form RNA (Figure 1 b), the amino acid part of the aa^6^A base would need to reside inside the helix, shielded from the outside. In the B‐form DNA one could imagine a decoration of the major groove with the amino acid side chains as depicted in Figure 1 c.

In the Schemes 1 and 2 we show the synthesis of the different urea linked amino acid A‐derivatives (aa^6^A). We first prepared the amino acid components for the coupling to the A‐nucleoside (Scheme 1). Our starting points for Thr^6^A, Ser^6^A and Asp^6^A were the free amino acids **1**–**3**, in which we first transformed all carboxylic acids into the *p*‐nitrophenylethyl esters (npe, **4**–**6**).^[17]^ The hydroxy groups of the Thr and Ser compounds were finally protected as TBS‐ethers to give the final products **7** and **8** (Scheme 1 a). For Val, Gly and Phe we started with the Boc‐protected amino acids **9**–**11**, which we also converted into the npe‐esters **12**–**14** using Mitsunobu type chemistry^[18]^ followed by acidic (4 m HCl in dioxane) Boc‐deprotection to give the amino acid products **15**–**17** (Scheme 1 b).^[19]^ For His^6^A, we again started with the Boc‐protected amino acid **18** (Scheme 1 c) and used HBTU activation to generate the npe ester **19**. Protection of the imidazole *N* ^τ^ with POM‐chloride followed again by Boc‐deprotection furnished the ready to couple amino acid **21**.

**Scheme 1:**
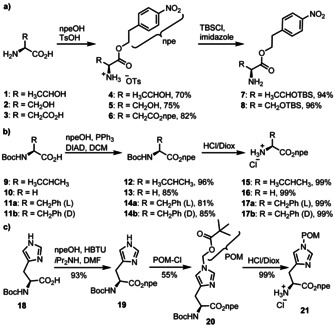
Synthesis of the amino acid building blocks as needed for the coupling to the nucleoside A to give Thr^6^A, Ser^6^A, Asp^6^A, Val^6^A, Gly^6^A, Phe^6^A and His^6^A.

**Scheme 2:**
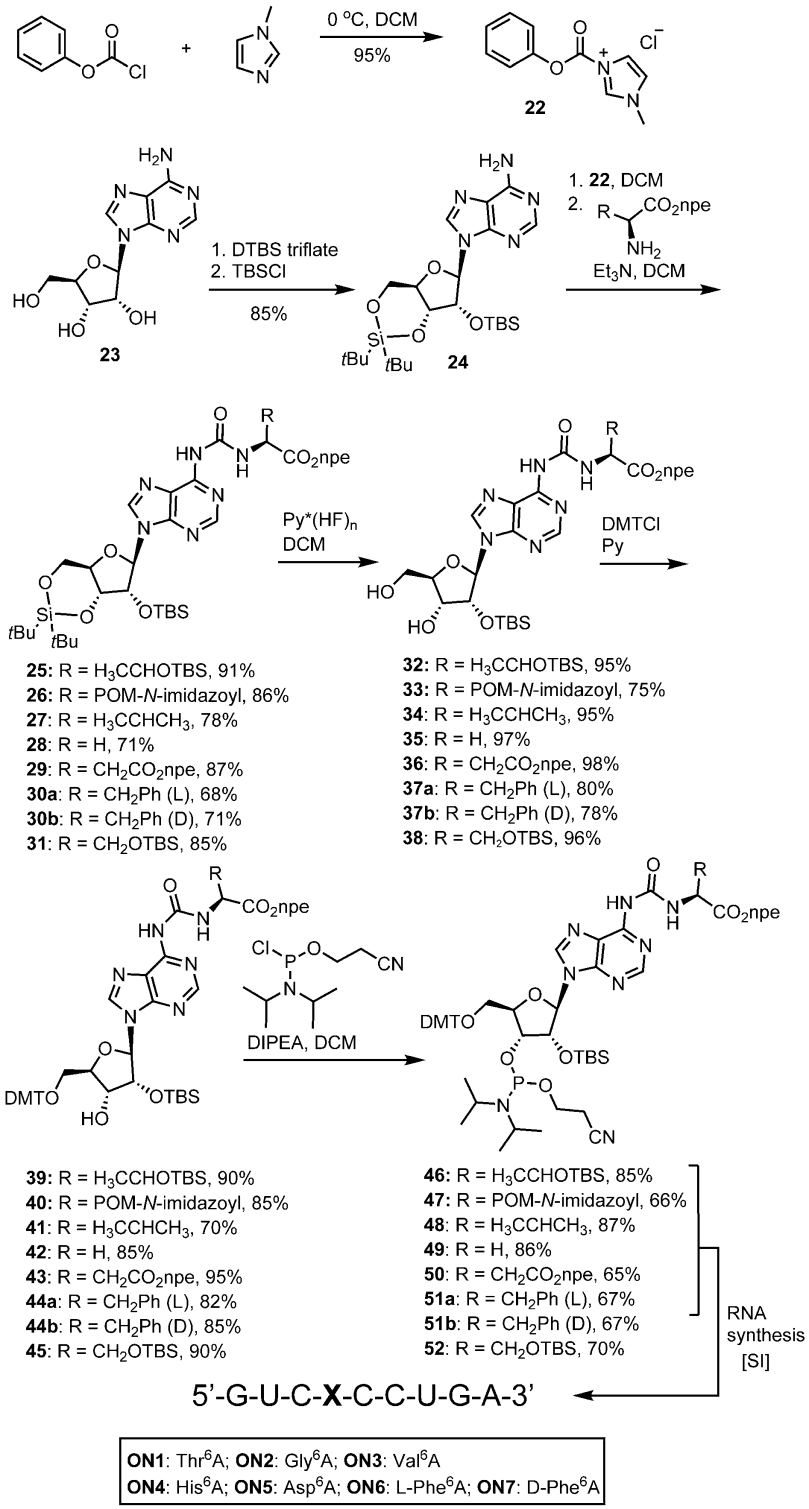
Synthesis of phosphoramidite building blocks of Thr^6^A, Ser^6^A, Asp^6^A, Val^6^A, Gly^6^A, Phe^6^A and His^6^A and their incorporation into RNA.

The connection of the amino acid with the A‐nucleoside via the urea moiety was next carried out as depicted in Scheme 2. We first treated phenyl chloroformate with *N*‐methylimidazole to obtain the 1‐*N*‐methyl‐3‐phenoxycarbonyl‐imidazolium chloride (**22**).^[20]^ Adenosine was converted in parallel into the cyclic 3′,5′‐silyl protected nucleoside, followed by conversion of the 2′‐OH group into the TBS‐ether.^[21]^ The reaction of compound **24** with the activated carbonate and the corresponding amino acid, provided in all cases the amino acid coupled products **25**–**31** in good to excellent yields. Subsequent cleavage of the cyclic silylether with HF⋅pyridine complex,[^22^, ^23^] protection of the 5′‐OH group with dimethoxytritylchloride (DMTCl)^[24]^ allowed the final conversion of the compounds into the corresponding phosphoramidites **46**–**52**. Standard solid phase RNA chemistry[^25^, ^26^, ^27^, ^28^, ^29^, ^30^, ^31^] was subsequently employed to prepare RNA strands containing the individual aa^6^A nucleosides stably embedded. The standard RNA synthesis protocol did not require any adjustment. In all cases we observed fair coupling of the aa^6^A phosphoramidites and no decomposition during deprotection. Deprotection required three steps. First, with DBU in THF at r.t. for 2 h we cleaved the npe‐protecting group. Second, we deprotected the bases and cleaved from the solid support with aqueous NH_3_/MeNH_2_. Finally, we removed the 2′‐silyl group with HF in NEt_3_.

In order to investigate how aa^6^A bases would affect the stability of DNA duplexes we also prepared as a representative molecule t^6^dA as depicted in Scheme 3. To this end we first acetyl‐protected dA **53**,^[32]^ performed the coupling of the protected threonine with the activated carbonate **22**, cleaved the acetyl groups and converted the nucleoside subsequently into the 5′‐DMT protected phosphoramidite **57**. The purification of compound **57** was quite difficult due to its high polarity. We needed to use rather polar mixture of EtOAc/Hex (2/1) as the mobile phase for the chromatographic separation. This provided the phosphoramidite **57**, however the material had a lower purity in comparison to the RNA phosphoramidites. Nevertheless, solid phase DNA synthesis and deprotection of the DNA strand **ODN1** proceeded again smoothly and in high yields.

**Scheme 3:**
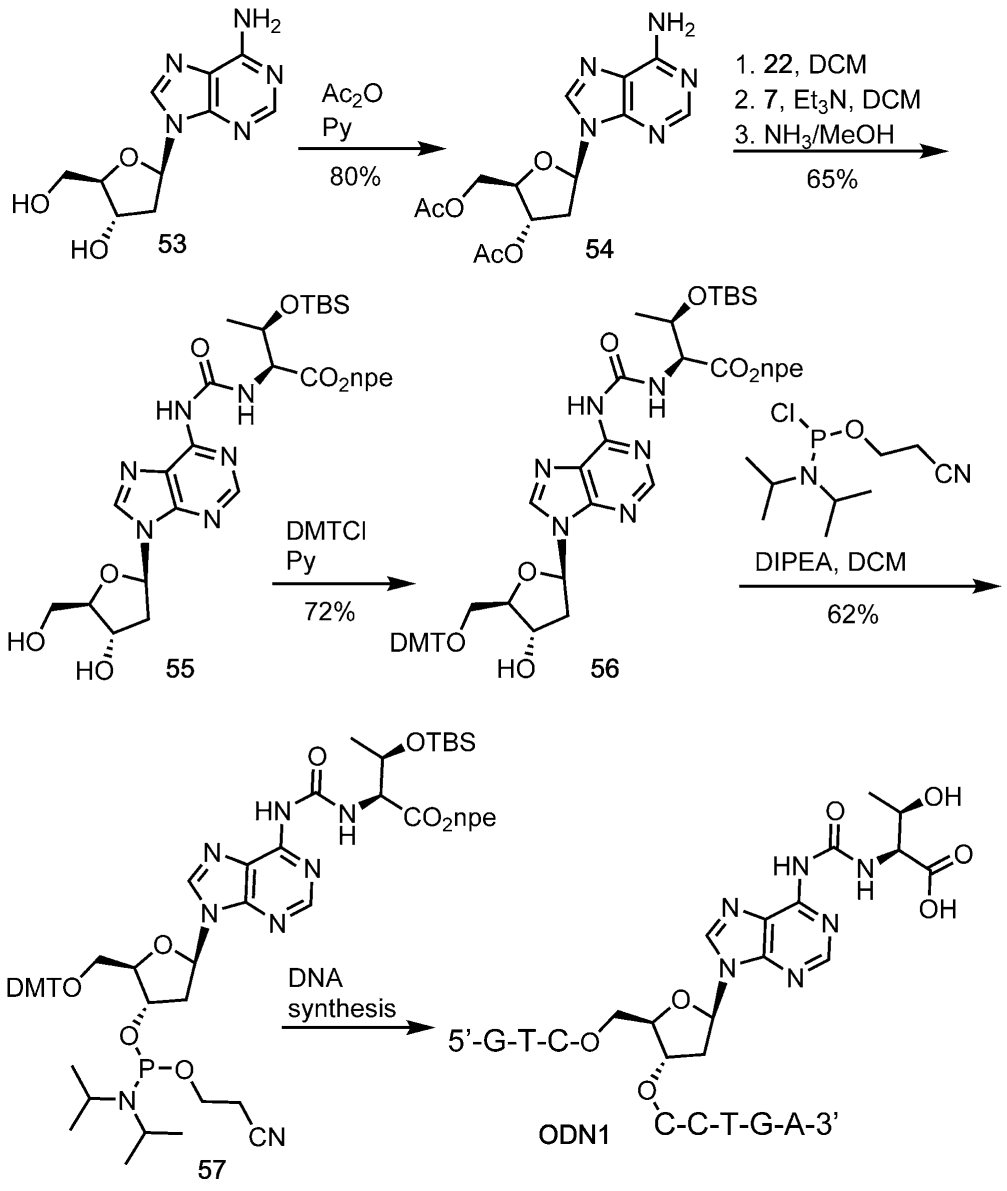
Synthesis of t^6^dA phosphoramidite and its incorporation into DNA.

Figure 2 a shows as an example the raw HPL‐chromatograms of **ON1** (RNA strand with embedded t^6^A) and the corresponding chromatogram after purification (inset) together with the obtained MALDI‐TOF mass spectrum (Figure 2 b). The chromatograms of the raw material show a good quality of the obtained RNA material. The analytical chromatogram after purification and the MALDI‐TOF data prove the purity of the finally obtained RNA oligonucleotide and the integrity of the t^6^A‐containing RNA strand.

**Figure 2:**
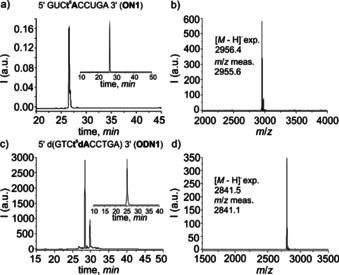
(a) Raw‐HPL chromatogram of **ON1**, with the inset showing the HPL‐chromatogram of purified **ON1**; (b) MALDI‐TOF mass spectrum of **ON1** after purification; (c) raw‐HPL chromatogram of **ODN1**, with the inset showing the HPL chromatogram of purified **ODN1**; (d) MALDI‐TOF mass spectrum of **ODN1** after purification.

Figure 2 c and 2 d show the same data set for the t^6^dA containing DNA oligonucleotide (**ODN1**), proving again the successful synthesis of t^6^dA containing oligonucleotide. The aa^6^(d)A nucleosides can exist in two different conformations.^[33]^ The first, s‐*trans*, maintains the Watson–Crick hydrogen bonding capabilities with the urea amino acid oriented towards the imidazole ring system (Figure 3 a). This allows formation of a Hoogsteen type 7‐membered ring H‐bond with the *N* ^7^. In the corresponding s‐*cis*‐conformation, the urea amino acid orients towards the Watson–Crick side thereby establishing a typically strong intramolecular 6‐membered H‐bond with *N* ^1^ (Figure 3 b). In order to investigate if the embedding of the amino acid would enforce s‐*trans*‐conformation and hence Watson–Crick H‐bonding, we measured melting points of all aa^6^A containing RNA strands and of the t^6^dA containing DNA strand hybridized to the corresponding counter strands (Figure 3). In the RNA:RNA situation we noted for all aa^6^A strands that we investigated, a single clear melting point, showing that only one conformer of the aa^6^A base likely exists in the RNA:RNA duplexes. In situation where the aa^6^A base exists in two different stable conformations, one would expect a more complex melting behaviour. In all cases we saw that the melting point is strongly reduced by 10–15 °C. When we embedded two aa^6^A building blocks into a short RNA strand no duplex formation was obtained. Even stronger reduction of the melting point was observed for the DNA duplex containing one t^6^dA. Here, we also saw just one sharp melting point and a reduction of the *T*_m_ by over 20 °C. These data show that the aa^6^A bases and among them t^6^A and g^6^A are unable to base pair. Although we have no direct proof of the structure the data argue for a preferred s‐*cis*‐conformation (Figure 3 b) in agreement with the literature.^[34]^

**Figure 3:**
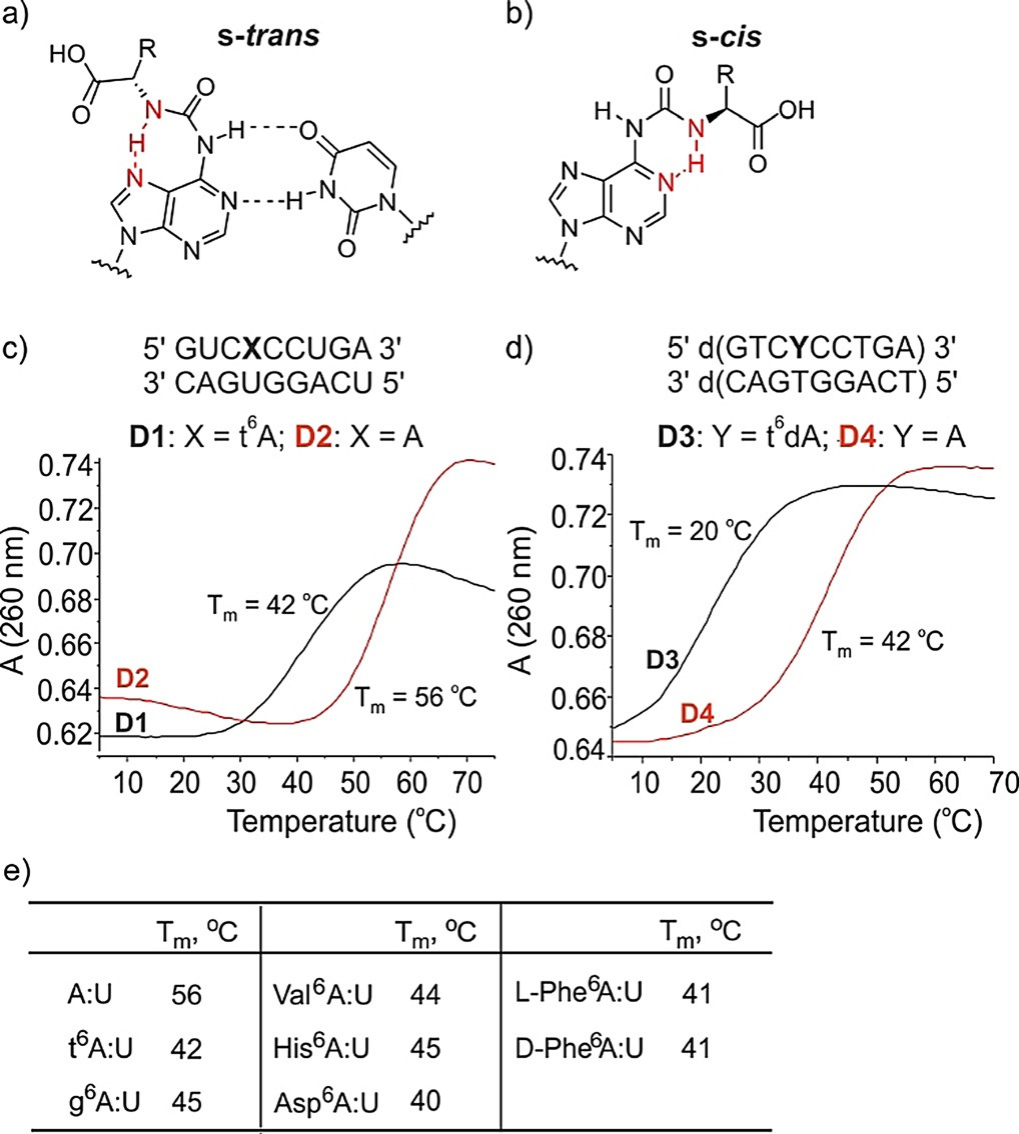
(a, b) Possible conformation, base pairing and intramolecular H‐bond of aa^6^A; (c, d) melting curves measured for t^6^A containing RNA:RNA duplexes and of a t^6^dA containing DNA:DNA duplex in comparison with the duplexes containing canonical (d)A:(d)T base pairs; (e) table of the determined melting points.

This conclusion is also supported by the observation that irrespective of the chirality of the attached amino acid (l‐ versus d‐Phe^6^A), we measured the same melting temperature. This would not be expected if the s‐*trans*‐conformation and base pairing would be possible. These data suggest that aa^6^A nucleosides within RNA position a given amino acid outside the A‐form helix in an unpaired situation and hence independent from the counterbase. As such, multiple aa^6^A containing RNA strands would be structures in which the RNA part is decorated by the amino acid side chains. In order to show that RNA‐structures containing multiple amino acids as representatives of an RNA‐peptide world can stably form, we prepared two RNA duplexes (Figure 4). In the first (**D5**), we placed three t^6^A bases as extra bases in an otherwise undisturbed RNA duplex. Indeed, now the stability of this duplex was indistinguishable from the same construct containing just canonical bases (**D6**). Finally, we prepared an RNA duplex **D7**, in which we placed the amino acids Ser‐Asp‐His directly next to each other to simulate what is known in the peptide world as the catalytic triad present in serine peptidases.^[35]^ Again in this case a stable duplex structure forms with the three aa^6^A bases creating a loop. Although we do not show any catalytic activity here, we believe that it is easily imaginable that if these amino acids are properly positioned in a stably folded RNA the structure could gain catalytic properties.

**Figure 4:**
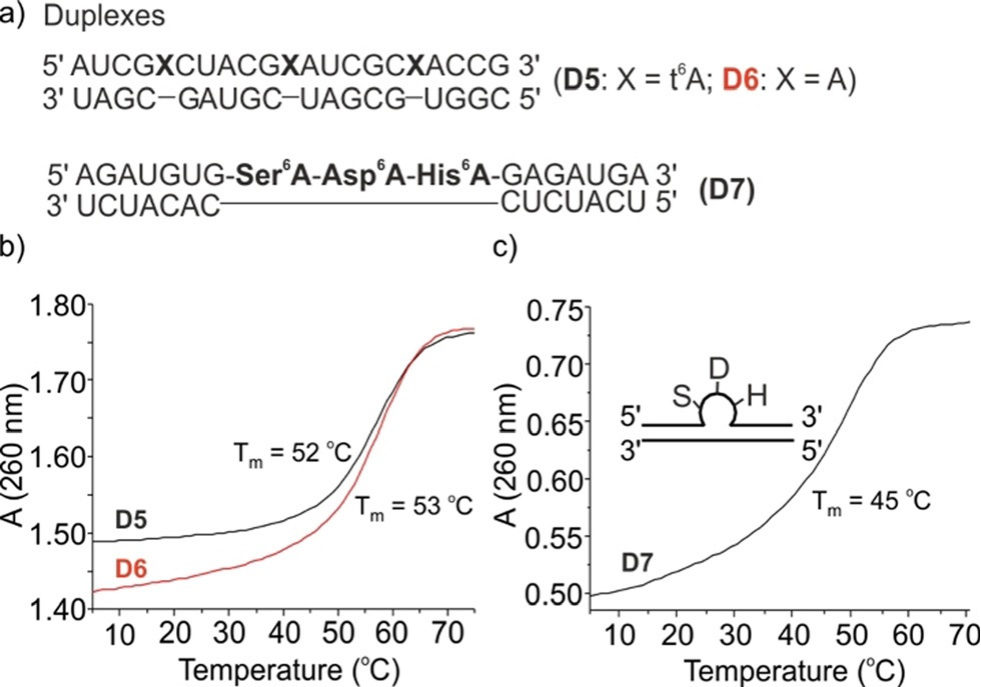
(a) Depiction of the RNA structures containing aa^6^A nucleobases in extrahelical positions forming either three little bulges or assembling a Ser‐Asp‐His triad known as the catalytic triad in serine proteases; (b, c) depiction of melting curves of duplexes **D5**, **D6**, **D7**; S: serine, D: aspartate, H: histidine.

The melting data show, that aa^6^A bases alone are unable to establish base pairing, which hinder them to encode sequence information. On the other side, these bases allow the incorporation of amino acids into RNA structures irrespective of the counterbase. Because RNAs are mostly stably folded structures in which many bases are not involved in any base pairing or establish no Watson–Crick interactions the amino acid adenosine nucleosides allow the stable incorporation of amino acid functionality into RNA.

In summary, here we investigated the synthesis and properties of aa^6^A nucleoside‐amino acid conjugates, some of which (t^6^A, g^6^A, hn^6^A) are today found as key components in the tRNAs of many species. In these tRNAs the aa^6^A nucleosides reside at the general purine position 37 adjacent to the anticodon loop. They are not involved in base pairing but fine tune the codon‐anticodon interaction to enable faithful translation of information into a peptide sequence.^[36]^ Here we show that these bases are indeed unable to base pair. They have to be placed outside the pairing regime that is needed for RNA folding. As such they function as anchors that allow the connection of amino acid to RNA structures independent of the counterbase. The side chains are then available to equip RNA with additional functions that might have been beneficial in an early RNA‐peptide world. The fact that aa^6^A nucleosides are stable structures and until today broadly found in today's RNA make them prime candidates to develop idea about the chemical constitution of the vanished RNA‐peptide world.

## Conflict of interest

The authors declare no conflict of interest.

## Supporting information

As a service to our authors and readers, this journal provides supporting information supplied by the authors. Such materials are peer reviewed and may be re‐organized for online delivery, but are not copy‐edited or typeset. Technical support issues arising from supporting information (other than missing files) should be addressed to the authors.

Supplementary
