# Supplementary material for: Amino Acid Modified RNA Bases as Building Blocks of an Early Earth RNA‐Peptide World
Source: Chemistry. 2020 Oct 14;26(65):14856–60. doi: 10.1002/chem.202002929 (PMC7756884; doi:10.1002/chem.202002929)
Supplement: Supplementary file 1 — Supplementary [file CHEM-26-14856-s001.pdf]

Chemistry–A European Journal

Supporting Information

Amino Acid Modified RNA Bases as Building Blocks of an Early Earth RNA-Peptide World

Milda Nainytė,[a] Felix Müller,[a] Giacomo Ganazzoli,[a] Chun-Yin Chan,[a] Antony Crisp,[a] Daniel Globisch,[b] and Thomas Carell*[a]

1. General Experimental Methods *2*
2. Synthesis of the Phosphoramidite Building-Blocks *3*
2.1. Synthesis of RNA building blocks 3
2.2. Synthesis of DNA building block 34
3. Synthesis and Purification of Oligonucleotides *38*
4. UV Melting Curve Measurements *42*
5. NMR spectra *43*
6. References *88*

1. General Experimental Methods

Chemicals were purchased from Sigma-Aldrich, TCI, Fluka, ABCR, Carbosynth or Acros Organics and used without further purification. Some of the strands were purchased from Metabion or Ella Biotech. Reagent-grade dry solvents (Sigma-Aldrich, Acros Organics) were stored over molecular sieves and handled under inert gas atmosphere. Reactions and chromatography fractions were monitored by qualitative thin-layer chromatography (TLC) on silica gel F254 TLC plates from Merck KGaA. Flash column chromatography was performed on Geduran® Si60 (40-63 µm) silica gel from Merck KGaA. NMR spectra were recorded on Bruker AVIIIHD 400 spectrometers (400 MHz). ^{1}H NMR shifts were calibrated to the residual solvent resonances: DMSO-d_6 (2.50 ppm), $CDCl_3$ (7.26 ppm), Acetone-d_6 (2.05 ppm), CD_2Cl_2 (5.32 ppm). ^{13}C NMR shifts were calibrated to the residual solvent: DMSO-d_6 (39.52 ppm), $CDCl_3$ (77.16 ppm), Acetone-d_6 (29.84 ppm), CD_2Cl_2 (53.84 ppm). All NMR spectra were analysed using the program MestreNova 10.0.1 from Mestrelab Research S. L. High resolution mass spectra were measured by the analytical section of the Department of Chemistry of the Ludwigs-Maximilians-Universität München on the spectrometer MAT 90 (ESI) from Thermo Finnigan GmbH. IR spectra were recorded on a PerkinElmer Spectrum BX II FT-IR system. All substances were directly applied as solids or on the ATR unit. Analytical RP-HPLC was performed on an analytical HPLC Waters Alliance (2695 Separation Module, 2996 Photodiode Array Detector) equipped with the column Nucleosil 120-2 C18 from Macherey Nagel using a flow of 0.5 ml/min, a gradient of 0-30% of buffer B in 45 min was applied. Preparative RP-HPLC was performed on a HPLC Waters Breeze (2487 Dual λ Array Detector, 1525 Binary HPLC Pump) equipped with the column VP 250/32 C18 from Macherey Nagel using a flow of 5 ml/min, a gradient of 0-25% of buffer B in 45 min was applied for the purifications. Oligonucleotides were purified using the following buffer system: buffer A: 100 mM NEt_3/HOAc (pH 7.0) in H_2O and buffer B: 100 mM NEt_3/HOAc in 80% (v/v) acetonitrile. The pH values of buffers were adjusted using a MP 220 pH-meter (Metter Toledo). Oligonucleotides were detected at wavelength: 260 nm. Melting profiles were measured on a JASCO V-650 spectrometer. Calculation of concentrations was assisted using the software OligoAnalyzer 3.0 (Integrated DNA Technologies: https://eu.idtdna.com/calc/analyzer). For strands containing artificial bases, the extinction coefficient of their corresponding canonical-only strand was employed without corrections. Matrix-assisted laser desorption/ionization-time-of-flight (MALDI-TOF) mass spectra were recorded on a Bruker Autoflex II. For MALDI-TOF

measurements, the samples were desalted on a 0.025 µm VSWP filter (Millipore) against ddH_2O and co-crystallized in a 3-hydroxypicolinic acid matrix (HPA).

2. Synthesis of the Phosphoramidite Building-Blocks

2.1. Synthesis of RNA building blocks

Npe-protection of carboxy group of amino acid

The reaction was performed according to the procedure published before. [1]

L-amino acid (1 eq.), 2-(4-nitrophenyl)ethanol (npe-OH, 3 eq.) and TsOH (3 eq.) were refluxed in toluene overnight in a *Dean-Stark* apparatus. The solution was cooled to room temperature and Et_2O was added. The oily residue was decanted, and the upper layer was removed to collect the oil. Precipitation of was induced by adding to the oil MeOH and Et_2O.

Compound 4

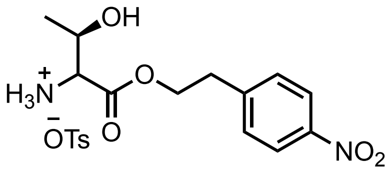

Yield: 70%; **IR**: $\tilde{\nu}$ = 3401 (w), 2930 (s), 2892 (s), 2858 (s), 1730 (s), 1510 (vs), 1465 (s), 1300 (vs), 1258 (s), 1167 (s), 1010 (s), 895 (w), 832 (s) cm^{-1}; **^{1}H NMR (400 MHz, DMSO-*d*$_6$) δ**: 8.2 – 8.1 (m, 5H), 7.58 (d, *J* = 8.8 Hz, 2H), 7.48 (d, *J* = 8.2 Hz, 2H), 7.11 (d, *J* = 8.2 Hz, 2H), 5.6 (br. s., 1H), 4.45 (t, *J* = 6.2 Hz, 2H), 4.1 – 4.0 (m, 1H), 3.89 (d, *J* = 4.0 Hz, 1H), 3.10 (t, *J* = 6.2 Hz, 2H), 2.29 (s, 3H), 1.14 (d, *J* = 6.6 Hz, 3H); **^{13}C NMR (101 MHz, DMSO-*d*$_6$) δ**: 168.2, 146.5, 146.3, 137.7, 130.4, 128.1, 125.6, 123.5, 65.4, 64.9, 57.9, 33.8, 20.7, 20.0; **HRMS (ESI)**: calculated for $C_{12}H_{17}N_2O_5^+$: m/z = 269.1137 $[M+H]^+$; found: m/z = 269.1140 $[M+H]^+$.

The analytical data is in agreement with the literature.[1]

Compound 5

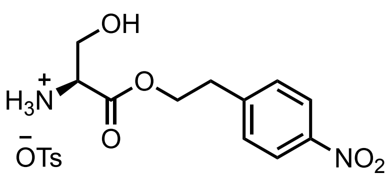

Yield: 75%; **IR**: $\tilde{\nu}$ = 3400 (w), 2931 (s), 2894 (s), 2858 (s), 1730 (s), 1510 (vs), 1465 (s), 1300 (vs), 1258 (s), 1167 (s), 1010 (s), 895 (w), 832 (s) cm^{-1}; **^{1}H NMR (400 MHz, DMSO-*d*$_6$) δ**: 8.34 (br. s, 3H), 8.16 (d, *J* = 8.9 Hz, 2H), 7.58 (d, *J* = 8.9 Hz, 2H), 7.50 (d, *J* = 8.0 Hz, 2H), 7.12 (d, *J* = 8.0 Hz, 2H), 4.49 – 4.36 (m, 2H), 4.11 (br. s, 1H), 3.74 – 3.73 (m, 2H), 3.08 (t, *J* = 6.4 Hz, 2H), 2.28 (s, 3H); **^{13}C NMR (101 MHz, DMSO-*d*$_6$) δ**: 168.0, 146.3, 138.0, 130.4, 128.2, 125.6, 123.5, 65.4, 59.5, 54.2, 33.9, 20.9; **HRMS (ESI)**: calculated for $C_{11}H_{15}N_2O_5^+$: m/z = 255.0981 $[M+H]^+$; found: m/z = 255.0977 $[M+H]^+$.

Compound 6

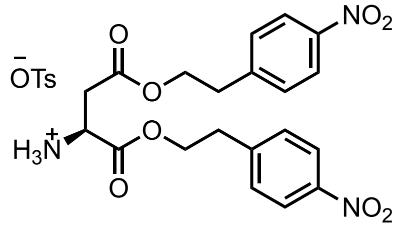

Yield: 82 %; **IR**: $\tilde{\nu}$ = 3402 (w), 2933 (s), 2894 (s), 2858 (s), 1730 (s), 1689 (s), 1514 (vs), 1469 (s), 1310 (vs), 1258 (s), 1167 (s), 1010 (s), 895 (w), 834 (s) cm^{-1}; **^{1}H NMR (400 MHz, DMSO-*d*$_6$) δ**: 8.42 (s, 3H), 8.16 – 8.11 (m, 4H), 7.55 – 7.48 (m, 6H), 7.12 (d, *J* = 7.9 Hz, 2H), 4.37 (t, *J* = 6.3 Hz, 2H), 4.34 – 4.30 (m, 1H), 4.26 (q, *J* = 6.3 Hz, 2H), 3.01 (q, *J* = 6.5 Hz, 4H), 2.85 (qd, *J* = 17.5, 5.1 Hz, 2H), 2.28 (s, 3H); **^{13}C NMR (101 MHz, DMSO-*d*$_6$) δ**: 169.0, 168.1, 146.4, 146.3, 145.3, 138.0, 130.3, 130.2, 128.2, 125.6, 123.5, 123.5, 65.7, 64.7, 48.4, 34.1, 33.8, 33.7, 20.8; **HRMS (ESI)**: calculated for $C_{20}H_{22}N_3O_8^+$: m/z = 432.1401 $[M+H]^+$; found: m/z = 432.1405 $[M+H]^+$.

General procedure of hydroxy group protection with TBSCl

The reaction was performed according to the procedure published before. [1]

Npe-protected ester (1 eq.) was dissolved in pyridine and treated with one half of TBSCl (3 eq.) and 1*H*-imidazole (3 eq.). After 10 min, the second half was added, and the reaction mixture was left to stir at room temperature overnight. The mixture was diluted with CH_2Cl_2 and washed successively with sat. $NaHCO_3$ solution and H_2O. The organic layer was dried, evaporated and purified by flash chromatography eluting with CH_2Cl_2/MeOH (10/1, v/v) to afford the target compound as an oil.

Compound 7

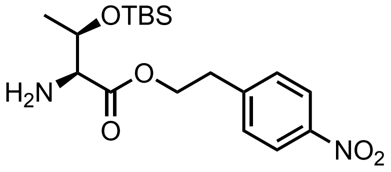

Yield: 94%; **IR**: $\tilde{\nu}$ = 3854 (w), 3745 (w), 2930 (w), 2856 (w), 1735 (vs), 1601 (s), 1518 (vs), 1472 (w), 1463 (w), 1374 (w), 1344 (vs), 1251 (s), 1155 (s), 1076 (s), 967 (s), 835 (s), 775 (s), 747 (s) cm^{-1}; **^{1}H NMR (400 MHz, $CDCl_3$) δ**: 8.16 (d, J = 8.6 Hz, 2H), 7.38 (d, J = 8.6 Hz, 2H), 4.41 (dt, J = 11.0, 6.8 Hz, 1H), 4.29 – 4.16 (m, 2H), 3.24 (d, J = 2.8 Hz, 1H), 3.06 (t, J = 6.8 Hz, 2H), 1.20 (d, J = 6.3 Hz, 3H), 0.80 (s, 9H), -0.01 (s, 3H), -0.10 (s, 3H); **^{13}C NMR (101 MHz, $CDCl_3$) δ**: 174.3, 147.0, 145.6, 129.9, 123.9, 69.6, 64.5, 60.9, 35.0, 25.7, 21.0, 17.9, -4.2, -5.2; **HRMS (ESI)**: calculated for $C_{18}H_{31}N_2O_5Si^+$: m/z = 383.2002 $[M+H]^+$; found: m/z = 383.1997 $[M+H]^+$.

The analytical data is in agreement with the literature.[1]

Compound 8

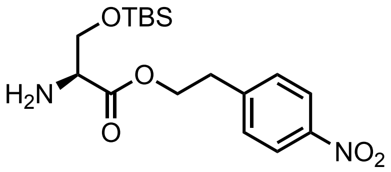

Yield: 96%; **IR**: $\tilde{\nu}$ = 3854 (w), 3745 (w), 2955 (w), 2930 (w), 2856 (w), 1735 (vs), 1601 (s), 1518 (vs), 1472 (w), 1374 (w), 1344 (vs), 1251 (s), 1155 (s), 1075 (s), 967 (s), 855 (w), 835 (s), 775 (s), 747 (s) cm^{-1}; **^{1}H NMR (400 MHz, $CDCl_3$) δ**: 8.13 (d, J = 8.6 Hz, 2H), 7.36 (d, J = 8.6 Hz, 2H), 4.34 (q, J = 6.7 Hz, 2H), 3.82 (dd, J = 9.8, 3.7 Hz, 1H), 3.73 (dd, J = 9.8, 3.0 Hz, 1H), 3.47 (s, 1H), 3.04 (t, J = 6.7 Hz, 2H), 0.81 (s, 9H); **^{13}C NMR (101 MHz, $CDCl_3$) δ**: 173.9, 146.9, 145.6, 129.8, 123.8, 65.4, 64.4, 56.5, 34.9, 25.7, 18.2, -5.5, -5.6; **HRMS (ESI)**: calculated for $C_{17}H_{29}N_2O_5Si^+$: m/z = 369.1846 $[M+H]^+$; found: m/z = 369.1842 $[M+H]^+$.

General procedure for npe-ester formation of Boc-protected amino acid

Boc-amino acid (1 eq.) was dissolved in CH_2Cl_2 under inert atmosphere and cooled to 0 °C. Then npeOH (1.3 eq.) and PPh_3 (1.3 eq.) were added followed by slow addition of DIAD (1.3 eq.). The reaction mixture was left to stir for 2 h at room temperature. Then the solution was washed with water, organic phase was dried over Na_2SO_4 and evaporated. The crude product was purified by flash chromatography to afford the target product.

Compound 12

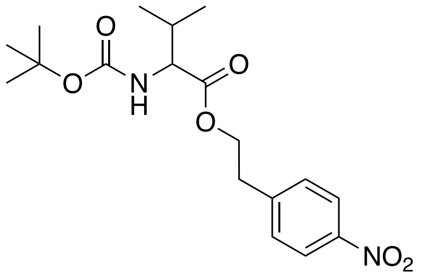

Eluent: Hex/EtOAc (4/1, v/v).

Yield: 96%; **IR**: $\tilde{\nu}$ = 3397 (w), 2975 (w), 1751 (s), 1709 (vs), 1519 (vs), 1391 (w), 1366 (w), 1345 (vs), 1159 (vs), 1056 (w), 905 (vs), 723 (vs) cm^{-1}; **^{1}H NMR (400 MHz, $CDCl_3$) δ**: 8.15 (d, J = 8.6 Hz, 2H), 7.38 (d, J = 8.6 Hz, 2H), 4.95 (d, J = 8.7 Hz, 1H), 4.38 (t, J = 6.6 Hz, 2H), 4.15 (dd, J = 9.0, 4.8 Hz, 1H), 3.06 (t, J = 6.6 Hz, 2H), 2.05 – 1.97 (m, 1H), 1.41 (s, 9H), 0.88 (d, J = 6.8 Hz, 3H), 0.78 (d, J = 6.8 Hz, 3H); **^{13}C NMR (101 MHz, $CDCl_3$) δ**: 172.4, 155.7, 146.9, 145.5, 129.8, 123.8, 79.9, 64.5, 58.6, 34.9, 31.2, 28.4, 19.0, 17.6; **HRMS (ESI)**: calculated for $C_{18}H_{27}N_2O_6^+$: m/z = 367.1869 [M+H]$^+$; found: m/z = 367.1874 [M+H]$^+$.

Compound 13

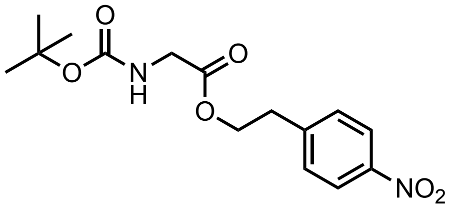

Eluent: Hex/EtOAc (3/1, v/v).

Yield: 85%; **IR**: $\tilde{\nu}$ = 3396 (w), 2978 (w), 2254 (w), 1750 (s), 1707 (vs), 1601 (w), 1518 (vs), 1391 (w), 1366 (w), 1345 (vs), 1250 (w), 1159 (vs), 1056 (w), 905 (vs), 727 (vs) cm^{-1}; **^{1}H NMR (400 MHz, $CDCl_3$) δ**: 8.10 (d, J = 8.6 Hz, 2H), 7.38 (d, J = 8.6 Hz, 2H), 4.97 (d, J = 9.2 Hz, 1H), 4.39 (t, J = 6.6 Hz, 2H), 3.87 (d, J = 5.8 Hz, 2H), 3.06 (t, J = 6.6 Hz, 2H), 1.43 (s, 9H); **^{13}C NMR (101 MHz, $CDCl_3$) δ**: 170.3, 155.8, 147.0, 145.4, 129.9, 123.9, 80.3, 64.7, 42.4, 34.9, 28.4; **HRMS (ESI)**: calculated for $C_{15}H_{21}N_2O_6^+$: m/z = 325.1400 [M+H]$^+$; found: m/z = 325.1398 [M+H]$^+$.

The analytical data is in agreement with the literature.[2]

Compound 14a

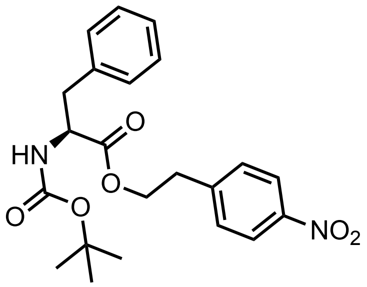

Eluent: Hex/EtOAc (4/1, v/v).

Yield: 81%; **IR**: $\tilde{\nu}$ = 3426 (w), 3356 (w), 1740 (w), 1709 (vs), 1602 (w), 1518 (vs), 1495 (s), 1344 (vs), 1249 (w), 1159 (vs), 1056 (w), 855 (s), 733 (s), 698 (vs) cm^{-1}; **^{1}H NMR (400 MHz, $CDCl_3$) δ**: 8.15 (d, *J* = 8.7 Hz, 2H), 7.31 (d, *J* = 8.7 Hz, 2H), 7.25 – 7.20 (m, 3H), 7.04 (d, *J* = 5.6 Hz, 2H), 4.90 (d, *J* = 8.4 Hz, 1H), 4.54 (q, *J* = 6.4 Hz, 1H), 4.39 – 4.25 (m, 2H), 3.07 – 2.93 (m, 4H), 1.41 (s, 9H); **^{13}C NMR (101 MHz, CD_2Cl_2) δ**: 171.8, 155.0, 146.9, 145.3, 135.8, 129.8, 129.2, 128.6, 127.1, 123.8, 80.1, 64.7, 54.5, 38.4, 34.7, 28.3; **HRMS (ESI)**: calculated for $C_{22}H_{26}N_2O_6Na^+$: m/z = 437.1683 $[M+Na]^+$; found: m/z = 437.1684 $[M+Na]^+$.

Compound 14b

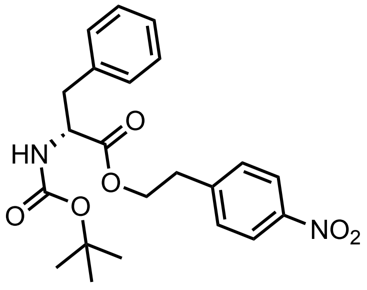

Eluent: Hex/EtOAc (4/1, v/v).

Yield: 85%; **IR**: $\tilde{\nu}$ = 3443 (w), 3375 (w), 2977 (w), 2929 (w), 1740 (w), 1709 (vs), 1602 (w), 1517 (vs), 1495 (s), 1343 (vs), 1248 (w), 1157 (vs), 1055 (w), 855 (s), 747 (s), 698 (vs) cm^{-1}; **^{1}H NMR (400 MHz, $CDCl_3$) δ**: 8.16 (d, *J* = 8.7 Hz, 2H), 7.33 (d, *J* = 8.7 Hz, 2H), 7.30 – 7.20 (m, 3H), 7.07 (d, *J* = 6.2 Hz, 2H), 4.97 (d, *J* = 8.4 Hz, 1H), 4.56 (q, *J* = 6.2 Hz, 1H), 4.40 – 4.27 (m, 2H), 3.08 – 2.93 (m, 4H), 1.43 (s, 9H); **^{13}C NMR (101 MHz, $CDCl_3$) δ**: 171.8, 155.1, 146.9, 145.4, 135.9, 129.8, 129.2, 128.6, 127.1, 123.8, 80.0, 64.7, 54.5, 38.4, 34.7, 28.3; **HRMS (ESI)**: calculated for $C_{22}H_{30}N_3O_6^+$: m/z = 432.2129 $[M+NH_4]^+$; found: m/z = 432.2131 $[M+NH_4]^+$.

Compound 19

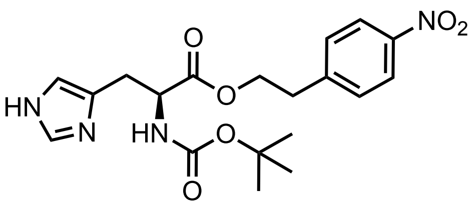

Boc-histidine (0.5 g, 1.96 mmol, 1 eq.), 2-(4-nitrophenyl)ethanol (0.655 g, 3.92 mmol, 2 eq.), DMAP (0.048 g, 0.39 mmol, 0.20 eq.) and HBTU (0.967 g, 2.55 mmol, 1.3 eq.) were dissolved in DMF (4 ml) under inert atmosphere. Diisopropylamine (686 µl, 4.90 mmol, 2.5 eq.) was added dropwise and the reaction mixture was stirred overnight at room temperature. The resulting solution was diluted with EtOAc (30 ml) and quenched with saturated NH_4Cl solution (15 ml). The organic layer was washed with water, dried and the solvents were removed *in vacuo*. The crude product was purified by flash chromatography on silica gel (2% to 5% MeOH in DCM) to obtain the target product as a pale-yellow foam.

Yield: 93%; **IR**: $\tilde{\nu}$ =2977 (w), 1699 (vs), 1600 (s), 1516 (vs), 1391 (w), 1365 (w), 1344 (vs), 1250 (w), 1160 (vs), 1108 (w), 1054 (w), 1016 (w), 855 (vs), 748 (w), 697 (w) cm^{-1}; **^{1}H NMR (400 MHz, $CDCl_3$) δ**: 8.18 – 8.07 (m, 2H), 7.58 (s, 1H), 7.41 – 7.30 (m, 2H), 6.72 (s, 1H), 5.77 (d, *J* = 8.2 Hz, 1H), 4.51 (q, *J* = 6.2 Hz, 1H), 4.34 (t, *J* = 6.6 Hz, 2H), 3.06 – 3.00 (m, 4H), 1.41 (s, 9H); **^{13}C NMR (101 MHz, $CDCl_3$) δ**: 172.1, 155.7, 147.1, 145.7, 135.2, 134.2, 130.0, 123.9, 115.9, 80.2, 64.9, 53.6, 34.9, 29.7, 28.5; **HRMS (ESI)**: calculated for $C_{19}H_{25}N_4O_6^+$: m/z = 405.1769 $[M+H]^+$; found: m/z = 405.1765 $[M+H]^+$.

General procedure for deprotection of Boc-protecting group

Npe-protected amino acid was dissolved in 4M HCl/Dioxane mixture at 0 °C. The reaction mixture was left to stir for 2 h and afterwards was evaporated to dryness. The resulting product was used for further steps without additional purification.

Compound 15

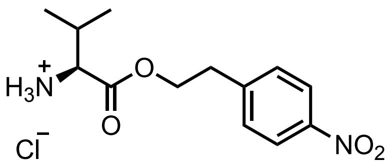

Yield: 99%; **IR**: $\tilde{\nu}$ = 3335 (w), 2964 (w), 2850 (w), 1741 (s), 1604 (w), 1516 (vs), 1464 (w), 1379 (vs), 1232 (vs), 1215 (s), 1170 (w), 1043 (w), 969 (w), 857 (s), 751 (s), 700 (s) cm^{-1}; **^{1}H**

NMR (400 MHz, DMSO-d_6) δ: 8.66 (br. s, 3H), 8.17 (d, *J* = 8.6 Hz, 2H), 7.62 (d, *J* = 8.6 Hz, 2H), 4.46 (dtd, *J* = 23.3, 11.1, 6.3 Hz, 2H), 3.76 (d, *J* = 4.5 Hz, 1H), 3.11 (t, *J* = 6.3 Hz, 2H), 2.10 (tt, *J* = 11.6, 5.8 Hz, 1H), 0.84 (d, *J* = 3.0 Hz, 3H), 0.82 (d, *J* = 3.0 Hz, 3H). **^{13}C NMR (101 MHz, DMSO-d_6) δ**: 168.8, 146.3, 130.4, 123.4, 65.3, 57.2, 33.8, 29.2, 18.3, 17.4 **HRMS (ESI)**: calculated for $C_{13}H_{19}N_2O_4^+$: m/z = 267.1339 [M+H]$^+$; found: m/z = 267.1139 [M+H]$^+$.

Compound 16

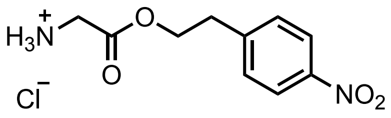

Yield: 99%; **IR**: $\tilde{\nu}$ = 2949 (w), 1746 (s), 1515 (vs), 1310 (vs), 1238 (vs), 1053 (w), 955 (s), 905 (s) 856 (s), 698 (s) cm^{-1}; **^{1}H NMR (400 MHz, DMSO-d_6) δ**: 8.30 (br. s, 3H), 8.18 (d, *J* = 8.7 Hz, 2H), 7.59 (d, *J* = 8.7 Hz, 2H), 4.44 (t, *J* = 6.4 Hz, 2H), 3.77 (s, 2H), 3.09 (t, *J* = 6.4 Hz, 2H); **^{13}C NMR (101 MHz, DMSO-d_6) δ**: 167.6, 156.2, 146.3, 130.4, 123.5, 67.9, 33.9, 28.2, 22.0; **HRMS (ESI)**: calculated for $C_{10}H_{13}N_2O_4^+$: m/z = 225.0870 [M+H]$^+$; found: m/z = 225.0868 [M+H]$^+$. The analytical data is in agreement with the literature.[2]

Compound 17a

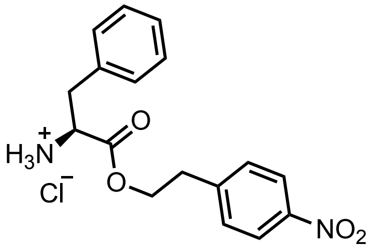

Yield: 99%; **IR**: $\tilde{\nu}$ = 3142 (w), 2988 (w), 2802 (w), 1740 (vs), 1601 (s), 1518 (vs), 1490 (vs), 1351 (vs), 1232 (vs), 1191 (s), 1102 (s), 981 (s), 856 (vs), 755 (vs), 736 (vs), 706 (vs) cm^{-1}; **^{1}H NMR (400 MHz, DMSO-d_6) δ**: 8.74 (br. s, 3H), 8.16 (d, *J* = 8.7 Hz, 2H), 7.49 (d, *J* = 8.7 Hz, 2H), 7.33 – 7.21 (m, 3H), 7.12 (dd, *J* = 7.8, 1.8 Hz, 2H), 4.33 (t, *J* = 6.3 Hz, 2H), 4.20 (dd, *J* = 7.8, 5.5 Hz, 1H), 3.16 (dd, *J* = 14.0, 5.5 Hz, 1H), 3.06 – 2.89 (m, 3H); **^{13}C NMR (101 MHz, DMSO-d_6) δ**: 168.9, 146.3, 134.9, 130.4, 129.4, 128.5, 127.2, 123.4, 65.4, 53.3, 35.8, 33.7; **HRMS (ESI)**: calculated for $C_{17}H_{19}N_2O_4^+$: m/z = 315.1339 [M+H]$^+$; found: m/z = 315.1332 [M+H]$^+$.

Compound 17b

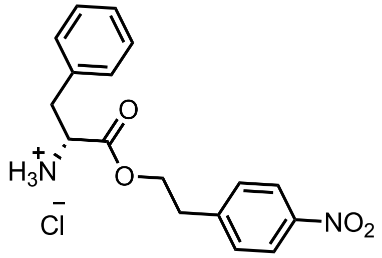

Yield: 99%; **IR**: $\tilde{\nu}$ = 3146 (w), 2988 (w), 2802 (w), 1740 (vs), 1602 (s), 1518 (vs), 1490 (vs), 1351 (vs), 1232 (vs), 1192 (s), 1102 (s), 856 (vs), 755 (vs), 736 (vs), 705 (vs) cm^{-1}; **^{1}H NMR (400 MHz, DMSO-*d*$_6$) δ**: 8.91 (br. s., 3H), 8.12 (d, *J* = 8.7 Hz, 2H), 7.46 (d, *J* = 8.7 Hz, 2H), 7.27 – 7.20 (m, 3H), 7.12 (d, *J* = 6.4 Hz, 2H), 4.29 (t, *J* = 6.4 Hz, 2H), 4.19 – 4.08 (m, 1H), 3.21 (dd, *J* = 14.1, 5.0, 1H), 3.10 – 2.88 (m, 3H); **^{13}C NMR (101 MHz, DMSO-*d*$_6$) δ**: 168.9, 146.3, 134.8, 130.4, 129.4, 128.5, 127.2, 123.4, 65.4, 53.3, 35.8, 33.7; **HRMS (ESI)**: calculated for $C_{17}H_{19}N_2O_4^+$: m/z = 315.1339 $[M+H]^+$; found: m/z = 315.1332 $[M+H]^+$.

Compound 21

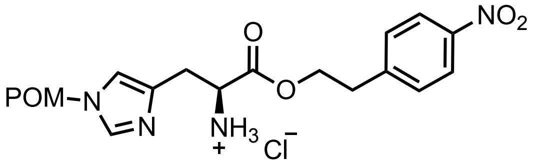

Yield: 98%; **IR**: $\tilde{\nu}$ = 2960 (w), 1737 (vs), 1624 (w), 1598 (w), 1516 (vs), 1454 (w), 1415 (w), 1351 (vs), 1282 (w), 1188 (w), 1124 (vs), 1045 (w), 1008 (w), 854 (s), 774 (w), 749 (w), 701 (w) cm^{-1}; **^{1}H NMR (400 MHz, DMSO-*d*$_6$) δ**: 8.69 (s, 3H), 8.21 – 8.15 (m, 2H), 7.59 – 7.55 (m, 2H), 7.51 (s, 1H), 6.04 (s, 2H), 4.39 (m, 3H), 3.56 (s, 1H), 3.17 (d, *J* = 6.9 Hz, 2H), 3.04 (td, *J* = 6.4, 3.9 Hz, 2H), 1.13 (s, 9H); **^{13}C NMR (101 MHz, DMSO-*d*$_6$) δ**: 176.7, 168.2, 146.4, 146.1, 137.4, 130.4, 123.5, 120.0, 69.2, 66.4, 65.6, 51.1, 38.2, 33.7, 26.5; **HRMS (ESI)**: calculated for $C_{20}H_{27}N_4O_6^+$: m/z = 419.1925 $[M+H]^+$; found: 419.1919 $[M+H]^+$.

Compound 20

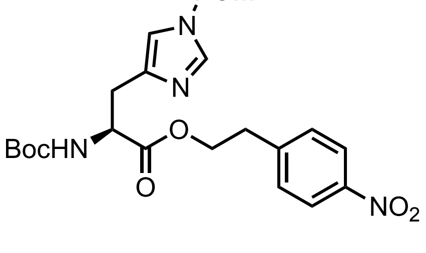

The histidine derivative **19** (0.745 g, 1.84 mmol, 1 eq.) was dissolved in DMF (12 mL) under N_2 atmosphere at 0 °C. Subsequently, K_2CO_3 (0.509 g, 3.68 mmol, 2 eq.) was added and the mixture was stirred for 40 min. Chloromethylpivalate (319 µl, 2.21 mmol, 1.2 eq.) was added dropwise at 0°C and the reaction mixture was left to warm to room temperature while stirring for 5 h. Catalytic amounts of KI were added and the mixture was stirred for another 1 h. The resulting suspension was diluted with EtOAc (75 ml) and quenched with saturated NH_4Cl solution (35 ml). The organic phase was washed with water, dried over Na_2SO_4 and concentrated *in vacuo*. The crude mixture was purified by flash chromatography on silica gel (40% isohexane in EtOAc to pure EtOAc). The pivalate protected histidine derivative was obtained as a yellow oil.

Yield: 55%; **IR**: 2850 (w), 2600 (w), 1738 (vs), 1624 (w), 1598 (w), 1573 (w), 1516 (vs), 1454 (w), 1414 (w), 1350 (vs), 1282 (w), 1191 (w), 1124 (vs), 1044 (w), 1008 (w), 854 (vs), 773 (w), 749 (w), 701 (w) cm^{-1}; **^{1}H NMR (400 MHz, $CDCl_3$) δ**: 8.19 – 8.14 (m, 2H), 7.54 (d, J = 1.4 Hz, 1H), 7.42 – 7.36 (m, 2H), 6.71 (s, 1H), 5.81 (d, J = 8.2 Hz, 1H), 5.73 (s, 2H), 4.51 (dt, J = 8.2, 5.3 Hz, 1H), 4.34 (t, J = 6.7 Hz, 2H), 3.04 (t, J = 6.7 Hz, 2H), 2.97 (t, J = 5.2 Hz, 2H), 1.42 (s, 9H), 1.14 (s, 9H); **^{13}C NMR (101 MHz, $CDCl_3$) δ**: 177.9, 172.0, 155.7, 147.0, 145.8, 138.3, 130.0, 123.9, 117.3, 79.9, 77.4, 67.7, 64.7, 53.5, 38.9, 35.0, 30.1, 28.5, 27.0; **HRMS (ESI)**: calculated for $C_{25}H_{35}N_4O_8^+$: m/z = 519.2449 $[M+H]^+$; found: m/z = 519.2441 $[M+H]^+$.

Compound 22

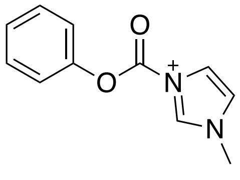

The reaction was conducted according to a published procedure.[3]

Phenyl chloroformate (4 ml, 31.9 mmol, 1 eq.) was dissolved in dry CH_2Cl_2 under nitrogen and cooled to 0 °C. Then *N*-methylimidazole (2.54 ml, 31.9 mmol, 1 eq.) was added dropwise. The mixture was allowed to stir at room temperature for 2 hours. Afterwards the reaction mixture was filtered, the precipitate was washed with CH_2Cl_2 and dried.

Yield: 95%; **IR**: $\tilde{\nu}$ = 2926 (w), 1783 (vs), 1588 (w), 1536 (w), 1372 (s), 1330 (s), 1232 (vs), 749 (vs), 689 (s) cm^{-1}; **^{1}H NMR (400 MHz, DMSO-*d*$_6$) δ**: 10.29 (s, 1H), 8.37 (s, 1H), 8.02 (s, 1H), 7.43 – 7.58 (m, 5H), 4.01 (s, 3H); **^{13}C NMR (101 MHz, DMSO-*d*$_6$) δ**: 157.5, 135.6, 129.3, 123.1, 121.3, 119.5, 118.6, 115.3, 35.4.

The analytical data is in agreement with the literature.[3]

Compound 24

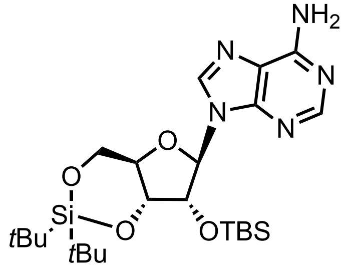

Compound was synthesized following the procedure published earlier.[4]

Adenosine **23** (1 g, 3.74 mmol, 1 eq.) was suspended in DMF and di-*tert*-butylsilyl ditriflate (1.46 ml, 4.49 mmol, 1.2 eq.) was added dropwise under stirring at 0 °C. The resulting solution was stirred at 0°C for 45 min. Then imidazole (1.27 g, 18.7 mmol, 5 eq.) was added and the reaction was warmed to room temperature over a period of 30 min. Then TBSCl (0.68 g, 4.49 mmol, 1.2 eq.) was added and the reaction was heated to 60 °C overnight. Subsequently, the reaction mixture was diluted with EtOAc and washed with water and brine. The organic layer was dried and evaporated. The residue was purified by flash chromatography (Hex/EtOAc, 1/1, v/v).

Yield: 76%; **IR**: $\tilde{\nu}$ = 3148 (w), 2933 (w), 2859 (w), 2361 (w), 1677 (s), 1604 (s), 1598 (w), 1576 (w), 1473 (w), 1426 (w), 1363 (w), 1329 (w), 1302 (w), 1258 (w), 1200 (w), 1166 (w), 1136 (w), 1105 (w), 1064 (vs), 1009 (s), 890 (w), 828 (vs), 786 (w), 754 (w), 729 (w) cm^{-1}; **^{1}H NMR (400 MHz, CDCl$_3$) δ**: 8.31 (s, 1H), 7.83 (s, 1H), 6.12 (br. s, 2H), 5.91 (s, 1H), 4.61 (d, *J* = 4.7 Hz, 1H), 4.50 (ddd, *J* = 16.5, 9.3, 4.7 Hz, 2H), 4.25 – 4.17 (m, 1H), 4.03 (dd, *J* = 10.5, 9.3 Hz, 1H), 1.07 (s, 9H), 1.04 (s, 9H), 0.92 (s, 9H), 0.16 (s, 3H), 0.14 (s, 3H); **^{13}C NMR (101 MHz, CDCl$_3$) δ**: 155.5, 152.8, 149.3, 138.9, 120.4, 92.6, 75.9, 75.6, 74.8, 67.9, 27.6, 27.1, 26.0, 22.9, 20.5, 18.4, -4.2, -4.8; **HRMS (ESI)**: calculated for $C_{24}H_{44}N_5O_4Si_2^+$: m/z = 522.2932 $[M+H]^+$; found: m/z = 522.2926 $[M+H]^+$.

The analytical data is in agreement with the literature.[4]

General procedure for amino acid attachment to the adenosine derivative

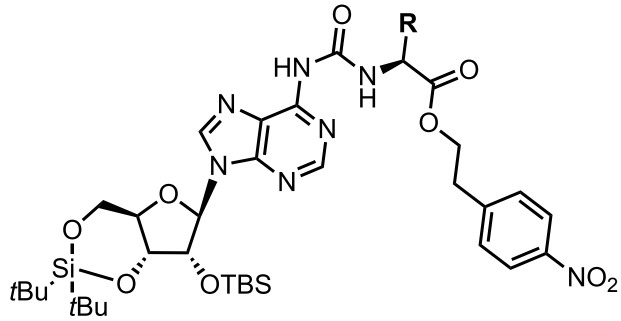

The silyl-protected adenosine derivative **24** (1 eq.) was dissolved in dry CH_2Cl_2 under nitrogen atmosphere. 1-*N*-methyl-3-phenoxycarbonyl-imidazolium chloride (**22**, 2 eq.) was added to the reaction mixture and the resulting suspension was stirred at room temperature for 2 hours (the solution in time becomes clear). Afterwards the protected amino acid (2 eq.) was added together with TEA (2 eq.) as a solution in CH_2Cl_2 and the resulting solution was stirred overnight at room temperature. The reaction was quenched by addition of saturated aqueous $NaHCO_3$ solution. The solution was extracted three times with CH_2Cl_2, and the organic phase was dried, filtered and concentrated *in vacuo*. The residue was purified by flash chromatography eluting with Hex/EtOAc to give product as white foam.

Compound 25

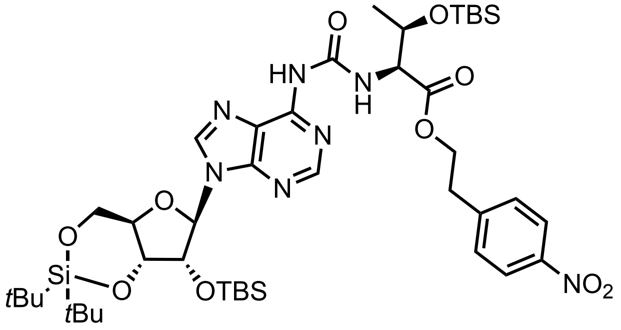

Eluent: Hex/EtOAc (4/3, v/v).

Yield: 91%; **IR**: $\tilde{\nu}$ = 3237 (w), 2931 (s), 2857 (s), 1737 (s), 1701 (vs), 1610 (s), 1520 (vs), 1465 (s), 1345 (s), 1250 (s), 1136 (w), 1057 (s), 998 (w), 894 (w), 840 (s), 777 (s) cm^{-1}; **^{1}H NMR (400 MHz, $CDCl_3$) δ**: 10.05 (d, *J* = 9.0 Hz, 1H), 8.56 (s, 1H), 8.41 (s, 1H), 8.16 (s, 1H), 7.94 (d, *J* = 8.6 Hz, 2H), 7.32 (d, *J* = 8.6 Hz, 2H), 5.99 (s, 1H), 4.64 (d, *J* = 4.6 Hz, 1H), 4.60-4.46 (m, 3H), 4.33-4.24 (m, 2H), 4.20-4.29 (m, 1H), 4.05 (dd, *J* = 10.5, 9.1 Hz, 1H), 3.03 (t, *J* = 6.5 Hz, 2H), 1.25 (d, *J* = 6.5 Hz, 3H), 1.08 (s, 9H), 1.05 (s, 9H), 0.95 (s, 9H), 0.90 (s, 9H), 0.19 (s, 3H), 0.16 (s, 3H), 0.07 (s, 3H), -0.04 (s, 3H); **^{13}C NMR (101 MHz, $CDCl_3$) δ**: 171.1, 154.6, 151.3, 150.3, 149.8, 146.8, 145.7, 141.6, 129.9, 123.7, 121.1, 92.6, 76.0, 75.7, 75.0, 68.8, 68.0, 64.8, 59.8, 35.0, 27.7, 27.2, 26.1, 25.7, 22.9, 21.3, 20.6, 18.5, 18.0, -4.1, -4.8, -5.2; **HRMS (ESI)**: calculated for $C_{43}H_{72}N_7O_{10}Si_3^+$: m/z = 930.4643 $[M+H]^+$; found: m/z = 930.4640 $[M+H]^+$.

Compound 26

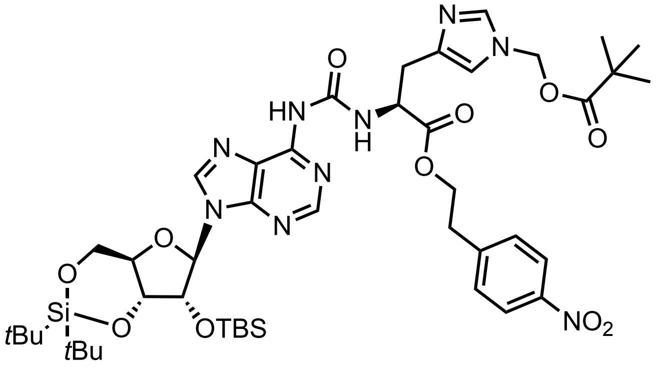

Eluent: 10% CH_2Cl_2 in EtOAc to pure EtOAc.

Yield: 86%; **IR**: $\tilde{\nu}$ = 3854 (w), 3745 (w), 3650 (w), 2932 (w), 2858 (w), 2361 (w), 2341 (w), 1735 (s), 1670 (s), 1654 (w), 1610 (w), 1587 (w), 1521 (vs), 1472 (s), 1395 (w), 1345 (vs), 1252 (w), 1166 (w), 1118 (vs), 1055 (vs), 999 (w), 894 (w), 826 (vs), 781 (s), 750 (w) cm^{-1}; **^{1}H NMR (400 MHz, $CDCl_3$) δ**: 10.05 (dd, *J* = 7.6, 3.7 Hz, 1H), 8.45 (s, 1H), 8.08 (s, 1H), 8.06 – 8.02 (m, 3H), 7.59 (d, *J* = 1.3 Hz, 1H), 7.41 – 7.35 (m, 2H), 6.86 (dd, *J* = 5.3, 1.3 Hz, 1H), 5.96 (d, *J* = 7.0 Hz, 1H), 5.74 (d, *J* = 2.6 Hz, 2H), 4.94 – 4.85 (m, 1H), 4.60 (dd, *J* = 6.0, 4.6 Hz, 1H), 4.53 – 4.46 (m, 2H), 4.42 (tq, *J* = 6.5, 1.7 Hz, 2H), 4.24 (tdd, *J* = 9.8, 5.0, 2.8 Hz, 1H), 4.08 – 4.00 (m, 1H), 3.23 – 3.12 (m, 2H), 3.07 (t, *J* = 6.5 Hz, 2H), 1.09 (s, 9H), 1.08 (s, 9H), 1.05 (s, 9H), 0.94 (s, 9H), 0.17 (s, 3H), 0.15 (s, 3H); **^{13}C NMR (101 MHz, $CDCl_3$) δ**: 177.8, 171.7, 153.6, 151.3, 150.2, 149.8, 146.9, 145.8, 141.2, 138.4, 138.1, 123.0, 123.8, 121.1, 117.3, 92.6, 76.0, 75.7, 74.9, 67.9, 67.7, 64.7, 55.5, 38.8, 35.0, 30.7, 27.7, 27.2, 26.9, 26.1, 22.9, 20.5, 18.5, 1.3, -4.2; **HRMS (ESI)**: calculated for $C_{45}H_{68}N_9O_{11}Si_2^+$: m/z = 966.4571 $[M+H]^+$; found: m/z = 966.4576 $[M+H]^+$.

Compound 27

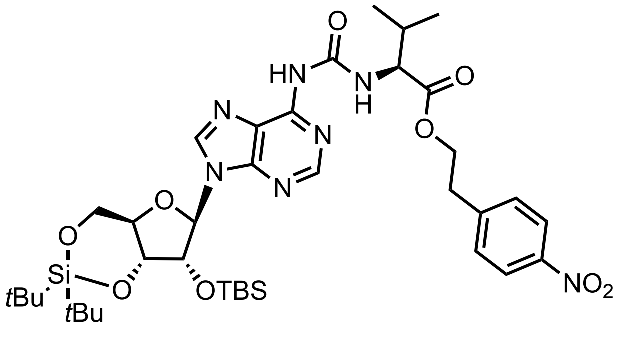

Eluent: Hex/EtOAc (4/3, v/v).

Yield: 78%; **IR**: $\tilde{\nu}$ = 3230 (w), 2960 (w), 2960 (w), 2858 (w), 1741 (s), 1702 (vs), 1611 (s), 1520 (vs), 1466 (s), 1345 (vs), 1250 (s), 1139 (vs), 1057 (vs), 999 (s), 894 (s), 810 (vs), 781 (s) cm^{-1}; **^{1}H NMR (400 MHz, $CDCl_3$) δ**: 10.01 (d, *J* = 8.4 Hz, 1H), 8.50 (s, 1H), 8.17 (s, 1H), 8.07 (d, *J* = 8.3 Hz, 2H), 7.28 (d, *J* = 8.3 Hz, 2H), 5.98 (s, 1H), 4.61 (d, *J* = 4.6 Hz, 1H), 4.56 – 4.38 (m, 5H), 4.20-4.29 (m, 1H), 4.07 (dd, *J* = 10.5, 9.1 Hz, 1H), 3.09 (t, *J* = 6.5 Hz, 2H), 2.28 – 2.21 (m, 1H), 1.08 (s, 9H), 1.05 (s, 9H), 1.00 (d, *J* = 6.5 Hz, 3H), 0.95 (s, 12H), 0.18 (s, 3H), 0.16 (s, 3H); **^{13}C NMR (101 MHz, $CDCl_3$) δ**: 171.1, 154.1, 152.2, 151.1, 149.8, 146.9, 145.6, 141.5, 129.9, 126.4, 123.7, 121.1, 92.6, 75.9, 75.7, 74.9, 67.9, 64.8, 58.8, 35.0, 30.9, 27.7, 27.2, 26.1, 22.9, 20.4, 19.5, 18.4, 18.0, -4.2, -4.9; **HRMS (ESI)**: calculated for $C_{38}H_{60}N_7O_9Si_2^+$: m/z = 814.3991 $[M+H]^+$; found: m/z = 814.3976.

Compound 28

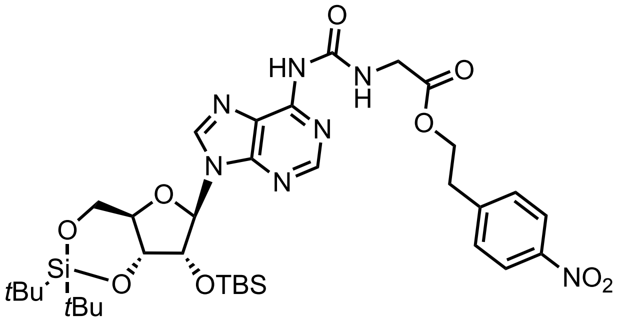

Eluent: Hex/EtOAc (4/3, v/v).

Yield: 72%; **IR**: $\tilde{\nu}$ = 3239 (w), 2932 (s), 2858 (s), 1749 (s), 1703 (vs), 1611 (s), 1520 (vs), 1468 (s), 1345 (s), 1252 (s), 1141 (w), 1055 (s), 990 (w), 894 (w), 826 (s), 750 (s) cm^{-1}; **^{1}H NMR (400 MHz, $CDCl_3$) δ**: 9.95 (br. s, 1H), 8.50 (s, 1H), 8.21 – 8.03 (m, 3H), 7.38 (d, *J* = 8.6 Hz, 2H), 5.98 (s, 1H), 4.60 (d, *J* = 4.7 Hz, 1H), 4.57 – 4.40 (m, 4H), 4.30 – 4.17 (m, 3H), 4.10 – 4.03 (m, 1H), 3.09 (t, *J* = 6.6 Hz, 2H), 1.08 (s, 9H), 1.05 (s, 9H) 0.94 (s, 9H), 0.17 (s, 3H), 0.16 (s, 3H); **^{13}C NMR (101 MHz, $CDCl_3$) δ**: 170.0, 154.2, 151.2, 150.2, 149.9, 147.0, 145.5, 141.4, 129.9, 123.8, 121.1, 92.5, 76.0, 75.7, 74.9, 67.9, 42.2, 35.0, 27.6, 27.2, 26.0, 22.9, 20.5, 18.5, -4.1, -4.9; **HRMS (ESI)**: calculated for $C_{35}H_{54}N_7O_9Si_2^+$: m/z = 772.3522 $[M+H]^+$; found: m/z = 772.3504 $[M+H]^+$.

Compound 29

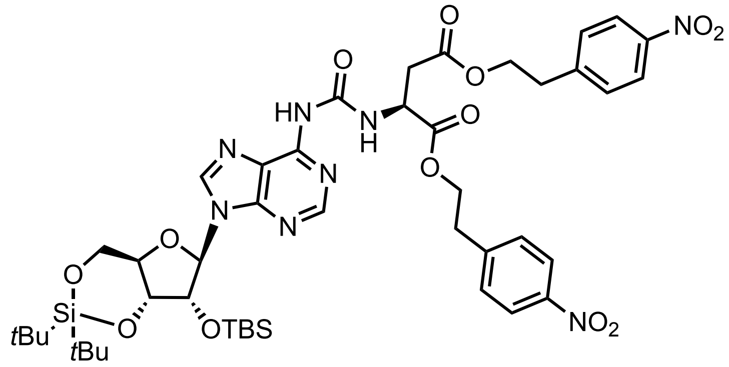

Eluent: Hex/EtOAc (2/1, v/v).

Yield: 87%; **IR**: $\tilde{\nu}$ = 3229 (w), 2953 (w), 2929 (w), 2857 (w), 1735 (s), 1693 (s), 1607 (s), 1589 (s), 1517 (vs), 1469 (s), 1391 (w), 1310 (vs), 1292 (w), 1251 (w), 1210 (w), 835 (s) cm^{-1}; **^{1}H NMR (400 MHz, CDCl$_3$) δ**: 10.28 (s, 1H), 8.42 (s, 1H), 8.22 – 8.12 (m, 1H), 8.07 (d, *J* = 8.6 Hz, 2H), 7.96 (d, *J* = 8.6 Hz, 2H), 7.37 – 7.30 (m, 4H), 6.00 (s, 1H), 4.93 – 4.89 (m, 1H), 4.64 (d, *J* = 4.6 Hz, 1H), 4.51 (ddd, *J* = 9.2, 4.9, 2.7 Hz, 2H), 4.46 – 4.40 (m, 2H), 4.39 – 4.22 (m, 3H), 4.09 – 4.05 (m, 1H), 3.08 – 2.94 (m, 6H), 1.07 (s, 9H), 1.05 (s, 9H), 0.95 (s, 9H), 0.19 (s, 3H), 0.17 (s, 3H); **^{13}C NMR (101 MHz, CDCl$_3$) δ**: 179.8, 170.7, 153.7, 149.8, 146.9, 146.8, 145.5, 145.4, 129.8, 123.8, 123.6, 120.9, 92.6, 75.9, 75.7, 74.9, 67.9, 65.0, 64.5, 49.7, 36.5, 34.9, 34.8, 27.6, 27.1, 26.0, 22.8, 20.5, 18.4, -4.2, -4.9; **HRMS (ESI)**: calculated for $C_{45}H_{63}N_8O_{13}Si_2^+$: m/z = 979.4053 [M+H]$^+$; found: m/z = 979.4056.

Compound 30a

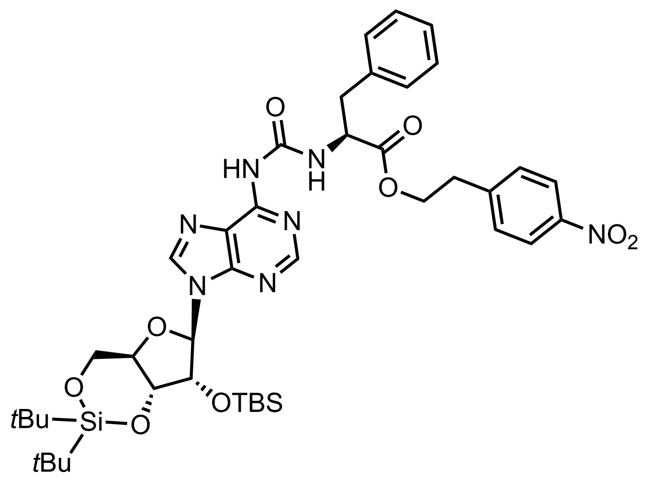

Yield: 68%; **IR**: $\tilde{\nu}$ = 3190 (w), 2933 (w), 2858 (w), 1742 (w), 1702 (vs), 1612 (s), 1587 (w), 1521 (vs), 1469 (vs), 1345 (vs), 1253 (s), 1057 (s), 1000 (w), 828 (vs) cm^{-1}; **^{1}H NMR (400 MHz, CD$_2$Cl$_2$) δ**: 10.00 (d, *J* = 7.5 Hz, 1H), 8.94 (s, 1H), 8.37 (d, *J* = 1.2 Hz, 2H), 8.00 (d, *J* =

8.7 Hz, 2H), 7.39 – 7.26 (m, 5H), 7.21 – 7.17 (m, 2H), 6.02 (s, 1H), 4.89 – 4.76 (m, 1H), 4.60 (d, *J* = 4.6 Hz, 1H), 4.54 – 4.46 (m, 2H), 4.40 (td, *J* = 6.5 Hz, 1.7 Hz, 2H), 4.31 – 4.22 (m, 1H), 4.16 – 4.06 (m, 1H), 3.18 (d, *J* = 6.8 Hz, 2H), 3.03 (t, *J* = 6.5 Hz, 2H), 1.10 (s, 9H), 1.06 (s, 9H), 0.96 (s, 9H), 0.20 (s, 3H), 0.18 (s, 3H); **^{13}C NMR (101 MHz, CD_2Cl_2) δ**: 171.5, 153.8, 150.7, 150.2, 149.8, 146.7, 145.9, 142.2, 136.4, 129.8, 129.5, 128.5, 127.1, 123.4, 120.8, 92.2, 75.8, 75.7, 74.8, 67.7, 64.6, 54.9, 37.8, 34.7, 27.3, 26.9, 25.7, 22.6, 20.2, 18.2, -4.6, -5.2; **HRMS (ESI)**: calculated for $C_{42}H_{60}N_7O_9Si_2^+$: m/z = 862.3986 [M+H]$^+$; found: m/z = 862.3995 [M+H]$^+$.

Compound 30b

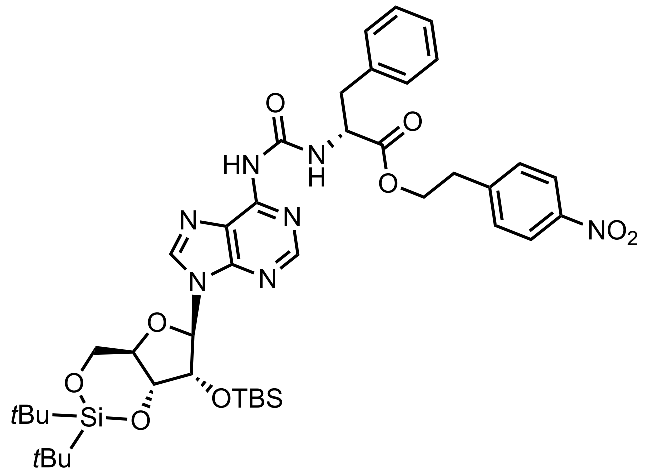

Eluent: Hex/EtOAc (4/1, v/v).

Yield: 71%; **IR**: $\tilde{\nu}$ = 3192 (w), 2933 (w), 2858 (w), 1740 (w), 1700 (vs), 1611 (s), 1520 (vs), 1466 (vs), 1345 (vs), 1252 (s), 1166 (w), 1139 (w), 1054 (vs), 998 (s), 893 (s), 826 (vs), 736 (vs) cm^{-1}; **^{1}H NMR (400 MHz, CD_2Cl_2) δ**: 10.01 (d, *J* = 7.6 Hz, 1H), 8.96 (s, 1H), 8.37 (s, 2H), 8.00 (d, *J* = 8.7 Hz, 1H), 7.37 – 7.22 (m, 5H), 7.22 – 7.14 (m, 2H), 6.03 (s, 1H), 4.90 – 4.81 (m, 1H), 4.61 (d, *J* = 4.5 Hz, 1H), 4.54 – 4.45 (m, 2H), 4.39 (t, *J* = 6.4 Hz, 2H), 4.32 – 4.21 (m, 1H), 4.16 – 4.05 (m, 1H), 3.19 (d, *J* = 6.2 Hz, 2H), 3.02 (t, *J* = 6.4 Hz, 2H), 1.09 (s, 9H), 1.06 (s, 9H), 0.96 (s, 9H), 0.20 (s, 3H), 0.18 (s, 3H); **^{13}C NMR (101 MHz, CD_2Cl_2) δ**: 171.4, 153.6, 150.8, 150.2, 149.8, 146.7, 145.9, 141.8, 136.4, 129.8, 129.5, 128.5, 127.1, 123.4, 120.8, 92.2, 75.9, 75.7, 74.8, 67.7, 64.6, 54.9, 37.9, 34.7, 27.3, 26.8, 25.7, 22.6, 20.2, 18.2, -4.6, -5.3.; **HRMS (ESI)**: calculated for $C_{42}H_{60}N_7O_9Si_2^+$: m/z = 862.3986 [M+H]$^+$; found: m/z = 862.3996 [M+H]$^+$.

Compound 31

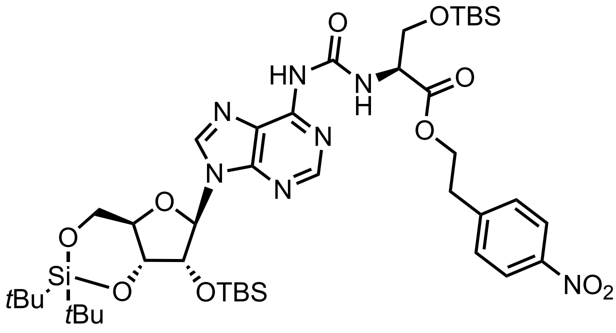

Eluent: Hex/EtOAc (1/1, v/v).

Yield: 85%; **IR**: $\tilde{\nu}$ = 3238 (w), 2931 (s), 2859 (s), 1737 (s), 1701 (vs), 1610 (s), 1520 (vs), 1470 (s), 1345 (s), 1251 (s), 1136 (w), 1057 (s), 998 (w), 899 (w), 840 (s), 778 (s) cm^{-1}; **^{1}H NMR (400 MHz, $CDCl_3$) δ**: 10.18 (d, *J* = 8.3 Hz, 1H), 8.61 (s, 1H), 8.44 (s, 1H), 8.19 (s, 1H), 8.02 (d, *J* = 8.6 Hz, 2H), 7.35 (d, *J* = 8.7 Hz, 2H), 5.99 (s, 1H), 4.72 (dt, *J* = 8.4, 2.8 Hz, 1H), 4.61 (d, *J* = 4.6 Hz, 1H), 4.50 (td, *J* = 9.8, 4.9 Hz, 2H), 4.43 (t, *J* = 6.5 Hz, 2H), 4.24 (td, *J* = 10.0, 5.0 Hz, 1H), 4.14 (dd, *J* = 10.1, 2.7 Hz, 1H), 4.07 (dd, *J* = 10.5, 9.1 Hz, 1H), 3.91 (dd, *J* = 10.1, 3.1 Hz, 1H), 3.06 (t, *J* = 6.5 Hz, 2H), 1.08 (s, 9H), 1.05 (s, 9H), 0.94 (s, 9H), 0.88 (s, 10H), 0.18 (s, 3H), 0.16 (s, 3H); **^{13}C NMR (101 MHz, $CDCl_3$) δ**: 170.6, 153.9, 151.2, 150.3, 149.8, 146.9, 145.6, 141.6, 129.8, 123.7, 121.1, 92.5, 75.9, 75.7, 74.9, 67.9, 64.8, 64.6, 55.7, 34.9, 27.6, 27.1, 26.0, 25.7, 22.8, 20.5, 18.4, 18.2, -4.2, -4.8, -5.3, -5.6; **HRMS (ESI)**: calculated for $C_{42}H_{70}N_7O_{10}Si_3^+$: m/z = 916.4492 $[M+H]^+$; found: m/z = 916.4501 $[M+H]^+$.

General procedure for deprotection 3'-5'-silyl protecting group:

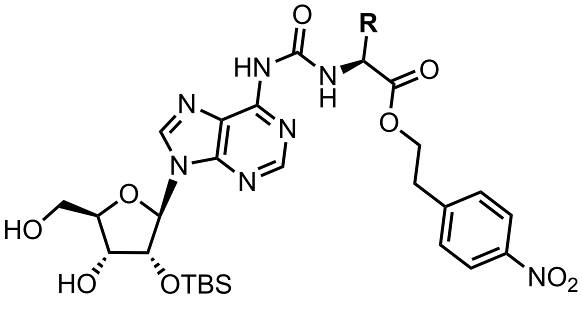

The modified adenosine (0.86 mmol) was dissolved in CH_2Cl_2 under N_2 atmosphere and transferred into a plastic flask. Pyridine (1 ml) was added and the solution was cooled in an ice-bath. Then Py*$(HF)_n$ (140 µl) was added and the mixture was stirred at 0 °C for 2 h. The reaction was quenched with sat. $NaHCO_3$ and extracted with CH_2Cl_2. Organic phase was washed with

water and dried over Na_2SO_4. The solvents were removed in vacuo. The crude product was purified by flash chromatography eluting with CH_2Cl_2/MeOH (9/1, v/v) to afford the product as a colourless foam.

Compound 32

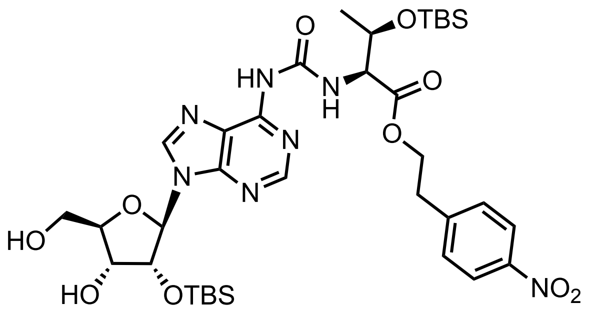

Yield: 95%; **IR**: $\tilde{\nu}$ = 3244 (w), 2952 (w), 2929 (w), 2856 (w), 1736 (w), 1695 (s), 1610 (s), 1588 (s), 1520 (vs), 1469 (s), 1345 (vs), 1313 (w), 1250 (vs), 1129 (w), 1093 (s), 835 (vs), 760 (vs) cm^{-1}; **^{1}H NMR (400 MHz, $CDCl_3$) δ**: 9.90 (d, *J* = 9.1 Hz, 1H), 8.50 (s, 1H), 8.46 (s, 1H), 8.10 – 8.08 (m, 3H), 7.37 (d, *J* = 8.6 Hz, 2H), 5.85 (d, *J* = 7.0 Hz, 1H), 5.10 (dd, *J* = 7.0, 4.7 Hz, 1H), 4.59 (dd, *J* = 9.1, 1.5 Hz, 1H), 4.53 – 4.41 (m, 2H), 4.41 – 4.34 (m, 2H), 4.34 – 4.26 (m, 1H), 3.98 (dd, *J* = 13.0, 1.5 Hz, 1H), 3.78 (dd, *J* = 13.0, 1.5 Hz, 1H), 3.07 (t, *J* = 6.7 Hz, 2H), 1.24 (d, *J* = 6.7 Hz, 3H), 0.89 (s, 9H), 0.81 (s, 9H), 0.05 (s, 3H), -0.06 (s, 3H), -0.14 (s, 3H), -0.34 (s, 3H); **^{13}C NMR (101 MHz, $CDCl_3$) δ**: 170.9, 154.0, 151.3, 150.9, 149.3, 147.1, 145.6, 143.0, 130.0, 123.9, 91.5, 87.7, 74.9, 72.7, 68.8, 65.13, 63.3, 59.8, 35.0, 25.7, 21.3, 18.1, 18.0, -4.0, -5.0, -5.1, -5.2; **HRMS (ESI)**: calculated for $C_{35}H_{56}N_7O_{10}Si_2^+$: m/z = 790.3622 $[M+H]^+$; found: m/z = 790.3612 $[M+H]^+$.

Compound 33

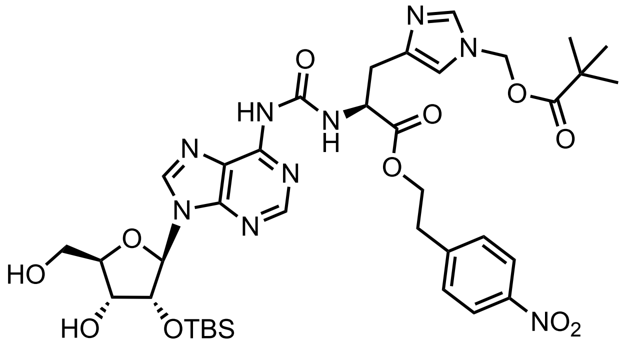

Yield: 75%; **IR**: $\tilde{\nu}$ = 3854 (w), 3745 (w), 3650 (w), 3230 (w), 2930 (w), 2857 (w), 2361 (w), 2341 (w), 1740 (s), 1699 (vs), 1611 (w), 1587 (w), 1520 (vs), 1472 (s), 1395 (w), 1345 (vs), 1253 (w), 1119 (vs), 1091 (w), 1058 (w), 1030 (w), 983 (w), 914 (w), 856 (vs), 779 (vs), 747(w), 670 (w) cm^{-1}; **^{1}H NMR (400 MHz, $CDCl_3$) δ**: 10.05 – 9.98 (m, 1H), 8.50 (d, *J* = 3.1 Hz, 1H), 8.18 – 8.10 (m, 3H), 8.01 (d, *J* = 9.1 Hz, 1H), 7.60 (dd, *J* = 8.2, 1.3 Hz, 1H), 7.44 – 7.37 (m, 2H), 6.84 (d, *J* = 1.4 Hz, 1H), 5.99 – 5.93 (m, 1H), 5.81 (dd, *J* = 7.3, 5.4 Hz, 1H), 5.77 – 5.71 (m, 2H), 5.08 (dt, *J* = 7.3, 5.1 Hz, 1H), 4.90 (dt, *J* = 7.2, 5.4 Hz, 1H), 4.42 (q, *J* = 6.3 Hz, 2H), 4.39 – 4.34 (m, 2H), 3.98 – 3.92 (m, 1H), 3.82 – 3.72 (m, 1H), 3.23 – 3.13 (m, 2H), 3.08 (dt, *J* = 13.8, 6.6 Hz, 2H), 2.83 (s, 1H), 1.09 (s, 9H), 0.80 (s, 9H), -0.18 (s, 3H), -0.40 (s, 3H); **^{13}C NMR (101 MHz, $CDCl_3$) δ**: 177.8, 171.8, 153.2, 151.1, 150.9, 149.3, 147.0, 145.8, 143.0, 138.2, 138.1, 130.0, 123.9, 123.1, 117.4, 91.4, 87.8, 74.7, 73.0, 67.7, 65.0, 63.5, 53.5, 38.8, 35.0, 30.6, 26.9, 25.7, 18.0, -5.1, -5.2; **HRMS (ESI)**: calculated for $C_{37}H_{52}N_9O_{11}Si^+$: m/z = 826.3550 $[M+H]^+$; found: m/z = 826.3559 $[M+H]^+$.

Compound 34

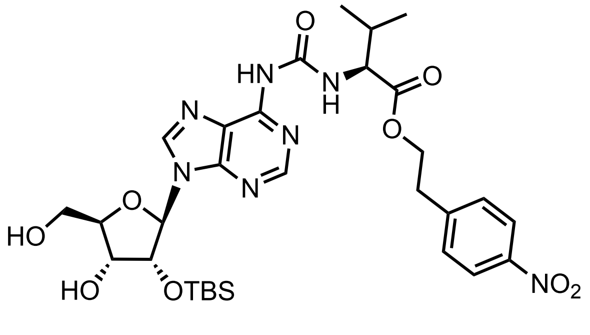

Yield: 95%; **IR**: $\tilde{\nu}$ = 3245 (w), 2930 (w), 2857 (w), 1743 (s), 1695 (vs), 1590 (s), 1518 (vs), 1471 (s), 1344 (vs), 1252 (s), 1212 (w), 1188 (s), 1130 (w), 1085 (s), 836 (vs) cm^{-1}; **^{1}H NMR (400 MHz, Acetone-*d*$_6$) δ**: 10.01 (d, *J* = 8.4 Hz, 1H), 8.50 (s, 1H), 8.17 (s, 1H), 8.07 (d, *J* = 8.3 Hz, 2H), 7.28 (d, *J* = 8.3 Hz, 2H), 5.98 (s, 1H), 4.61 (d, *J* = 4.6 Hz, 1H), 4.56 – 4.38 (m, 6H), 4.20-4.29 (m, 1H), 4.07 (dd, *J* = 10.5, 9.1 Hz, 1H), 3.09 (t, *J* = 6.5 Hz, 2H), 1.00 (d, *J* = 6.5 Hz, 3H), 0.96 (d, *J* = 6.5 Hz, 3H), 0.95 (s, 9H), 0.18 (s, 3H), 0.16 (s, 3H); **^{13}C NMR (101 MHz, $CDCl_3$) δ**: 172.2, 154.4, 151.6, 149.5, 147.0, 145.5, 143.3, 136.5, 129.9, 123.7, 122.0, 91.2, 87.6, 74.7, 72.8, 64.7, 63.4, 58.7, 35.0, 30.9, 25.6, 19.4, 17.9, -5.2, -5.3; **HRMS (ESI)**: calculated for $C_{30}H_{44}N_7O_9Si^+$: m/z = 674.2970 $[M+H]^+$; found: m/z = 674.2974 $[M+H]^+$.

Compound 35

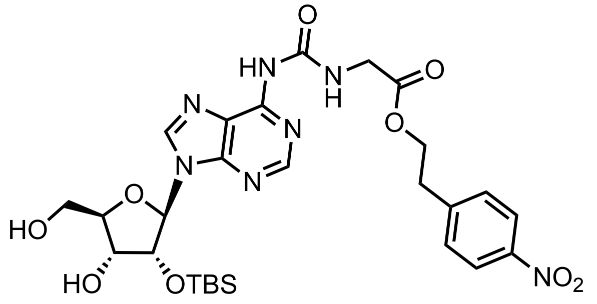

Yield: 97%; **IR**: $\tilde{\nu}$ = 3250 (w), 2932 (w), 2859 (w), 1740 (s), 1680 (vs), 1595 (s), 1520 (vs), 1470 (s), 1340 (vs), 1254 (s), 1213 (w), 1168 (s), 1130 (w), 1085 (s), 836 (vs) cm^{-1}; **^{1}H NMR (400 MHz, $CDCl_3$) δ**: 9.92 (t, *J* = 5.7 Hz, 1H), 8.92 (br. s, 1H), 8.54 (s, 1H), 8.25 (s, 1H), 8.13 (d, *J* = 8.7 Hz, 2H), 7.39 (d, *J* = 8.7 Hz, 2H), 5.87 (d, *J* = 7.2 Hz, 1H), 5.07 (dd, *J* = 7.2, *J* = 4.7 Hz, 1H), 4.44 (t, *J* = 6.7 Hz, 2H), 4.37 (m, 2H), 4.20 (t, *J* = 5.2 Hz, 2H), 3.97 (d, *J* = 13.0 Hz, 1H), 3.77 (d, *J* = 13.0 Hz, 1H), 3.09 (t, *J* = 6.7 Hz, 2H), 2.94 (br. s, 1H), 0.78 (s, 9H), -0.19 (s, 3H), -0.38 (s, 3H); **^{13}C NMR (101 MHz, $CDCl_3$) δ**: 169.9, 154.0, 150.8, 149.5, 147.0, 145.4, 143.6, 129.9, 123.9, 122.0, 91.2, 87.6, 74.8, 72.8, 64.9, 63.3, 42.2, 34.9, 25.6, 17.9, -5.2; **HRMS (ESI)**: calculated for $C_{27}H_{37}N_7O_9Si^+$: m/z = 632.2495 $[M + H]^+$; found m/z = 632.2492.

Compound 36

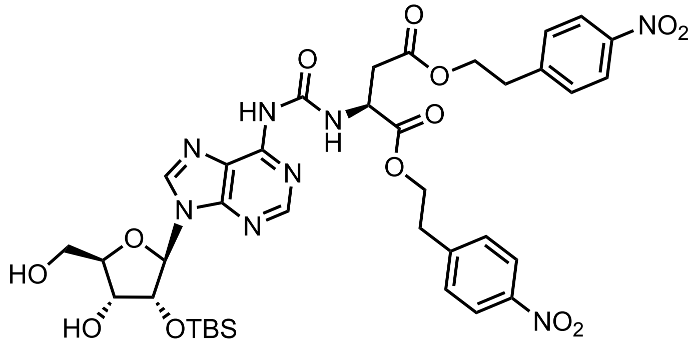

Yield: 98%; **IR**: $\tilde{\nu}$ = 3229 (w), 2953 (w), 2929 (w), 2857 (w), 1735 (s), 1693 (s), 1607 (s), 1589 (s), 1517 (vs), 1469 (s), 1391 (w), 1310 (vs), 1292 (w), 1251 (w), 1210 (w), 835 (s) cm^{-1}; **^{1}H NMR (400 MHz, Acetone-*d*$_6$) δ**: 10.30 (d, *J* = 7.9 Hz, 1H), 9.41 (s, 1H), 8.77 (s, 1H), 8.50 (s, 1H), 8.10 (d, *J* = 8.8 Hz, 2H), 8.06 (d, *J* = 8.8 Hz, 2H), 7.54 (dd, *J* = 8.7, 7.4 Hz, 4H), 6.12 (d, *J* = 5.8 Hz, 1H), 5.07 (s, 1H), 5.00 – 4.95 (m, 1H), 4.90 (dt, *J* = 7.9, 5.3 Hz, 1H), 4.49 – 4.31 (m, 5H), 4.23 (q, *J* = 2.4 Hz, 1H), 4.06 (s, 1H), 3.92 (dd, *J* = 12.4, 2.1 Hz, 1H), 3.79 (d, *J* = 12.2 Hz, 1H), 3.10 (dt, *J* = 12.6, 6.4 Hz, 4H), 3.02 (d, *J* = 5.5 Hz, 2H), 0.79 (s, 9H), -0.08 (s, 3H), -0.21 (s,

3H); **^{13}C NMR (101 MHz, Acetone-*d*$_6$) δ**: 171.3, 171.2, 154.2, 151.4, 150.9, 150.6, 147.6, 147.4, 147.2, 144.3, 136.6, 131.0, 124.1, 121.9, 90.6, 87.6, 76.8, 72.4, 65.8, 65.1, 62.9, 50.6, 37.1, 35.2, 27.2, 26.0, 18.6, -4.9, -5.1; **HRMS (ESI)**: calculated for $C_{37}H_{47}N_8O_{13}Si^+$: m/z = 839.3032 $[M+H]^+$; found: m/z = 839.3041 $[M+H]^+$.

Compound 37a

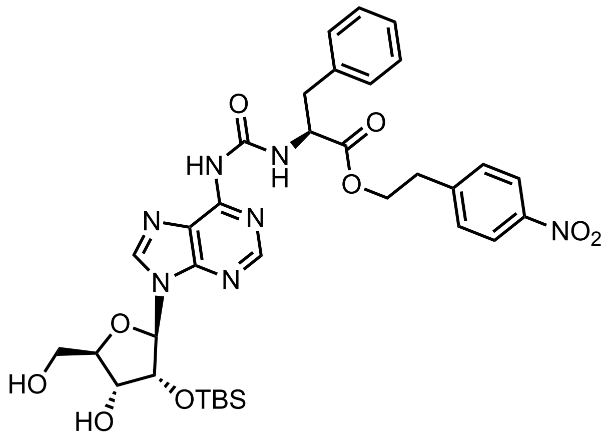

Yield: 80%; **IR**: $\tilde{\nu}$ = 3240 (w), 2929 (w), 2857 (w), 1742 (w), 1699 (vs), 1613 (s), 1520 (vs), 1471 (s), 1345 (vs), 1255 (w), 839 (s) cm^{-1}; **^{1}H NMR (400 MHz, CD_2Cl_2) δ**: 9.83 (d, *J* = 7.4 Hz, 1H), 8.63 (s, 1H), 8.40 (s, 1H), 8.18 (s, 1H), 8.10 (d, *J* = 8.7 Hz, 2H), 7.38 (d, *J* = 8.7 Hz, 2H), 7.33 – 7.21 (m, 2H), 7.19 – 7.14 (m, 2H), 5.87 (d, *J* = 7.2 Hz, 1H), 5.64 (dd, *J* = 11.6 Hz, *J* = 2.3 Hz, 1H), 5.07 (dd, *J* = 7.2 Hz, *J* = 4.7 Hz, 1H), 4.87 – 4.80 (m, 1H), 4.45 – 4.32 (m, 4H), 3.93 (d, *J* = 12.9 Hz, 1H), 3.79 – 3.69 (m, 1H), 3.17 (d, *J* = 6.2 Hz, 2H), 3.05 (t, *J* = 6.6 Hz, 2H), 2.93 (s, 1H), 0.79 (s, 9H), -0.19 (s, 3H), -0.38 (s, 3H); **^{13}C NMR (101 MHz, CD_2Cl_2) δ**: 171.4, 153.1, 150.7, 150.5, 149.8, 149.3, 146.8, 145.8, 143.3, 136.3, 129.8, 129.4, 128.3, 127.2, 123.5, 91.0, 87.6, 74.8, 72.7, 64.7, 63.1, 54.9, 37.8, 34.7, 25.3, 17.7, -5.6, -5.7; **HRMS (ESI)**: calculated for $C_{34}H_{44}N_7O_9Si^+$: m/z = 722.2965 $[M+H]^+$; found: m/z = 722.2971 $[M+H]^+$.

Compound 37b

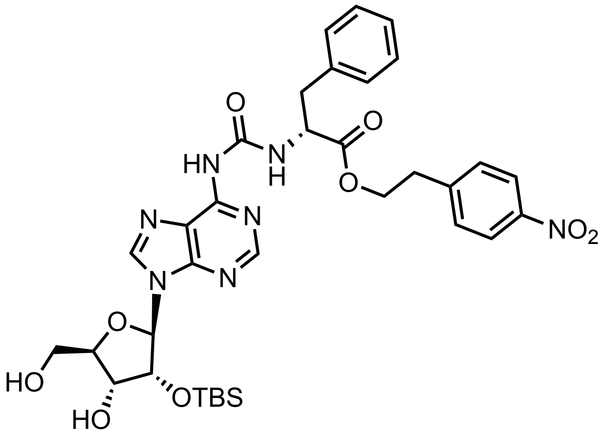

Yield: 78%; **IR**: $\tilde{\nu}$ = 3229 (w), 2930 (w), 2858 (w), 1740 w), 1697 (vs), 1612 (s), 1519 (vs), 1470 (s), 1345 (vs), 1254 (s), 1216 (w), 838 (vs), 781 (s), 736 (vs), 700 (s) cm^{-1}; **^{1}H NMR (400 MHz, CD_2Cl_2) δ**: 9.79 (br. s, 1H), 8.58 (br. s, 1H), 8.39 (s, 1H), 8.17 (t, *J* = 4.0 Hz, 1H), 8.05 (d, *J* = 8.7 Hz, 2H), 7.35 (d, *J* = 8.7 Hz, 2H), 7.32 – 7.23 (m, 2H), 7.21-7.15 (m, 2H), 5.87 (d, *J* = 7.1 Hz, 1H), 5.61 (dd, *J* = 11.6 Hz, 2.4 Hz, 1H), 5.05 (dd, *J* = 7.2 Hz, 4.7 Hz, 1H), 4.82 (q, *J* = 6.2 Hz, 1H), 4.46 – 4.30 (m, 4H), 3.93 (d, *J* = 12.9 Hz, 1H), 3.79 – 3.68 (m, 1H), 3.17 (d, *J* = 6.2 Hz, 2H), 3.03 (t, *J* = 6.5 Hz, 3H), 2.88 (s, 1H), 0.80 (s, 9H), -0.17 (s, 3H), -0.36 (s, 3H); **^{13}C NMR (101 MHz, CD_2Cl_2) δ**: 171.4, 153.1, 150.7, 150.5, 149.8, 149.3, 146.8, 145.8, 143.3, 136.3, 129.8, 129.4, 128.5, 128.3, 127.2, 123.5, 91.0, 87.6, 74.8, 72.7, 64.7, 63.1, 54.9, 37.8, 34.7, 25.3, 17.7, -5.6, -5.7; **HRMS (ESI)**: calculated for $C_{34}H_{43}N_7O_9SiNa^+$: m/z = 744.2784 $[M+Na]^+$; found: m/z = 744.2776 $[M+Na]^+$.

Compound 38

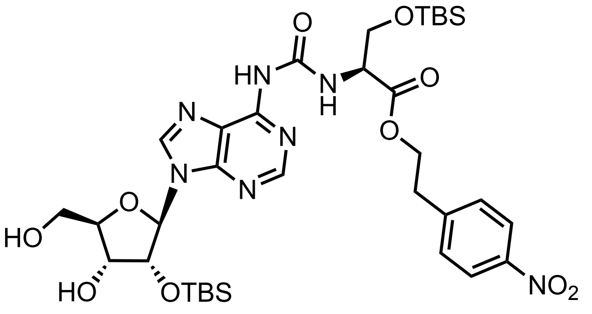

Yield: 96%; **IR**: $\tilde{\nu}$ = 3240 (w), 2950 (w), 2927 (w), 2856 (w), 1737 (w), 1695 (s), 1611 (s), 1589 (s), 1520 (vs), 1469 (s), 1346 (vs), 1313 (w), 1250 (vs), 1130 (w), 1093 (s), 835 (vs), 780 (vs) cm^{-1}; **^{1}H NMR (400 MHz, Acetone-*d_6*) δ**: 10.11 (d, *J* = 8.2 Hz, 1H), 9.13 (s, 1H), 8.72 (s, 1H), 8.52 (s, 1H), 8.11 (d, *J* = 8.7 Hz, 2H), 7.59 (d, *J* = 8.7 Hz, 2H), 6.13 (d, *J* = 6.1 Hz, 1H), 5.07 (dd, *J* = 8.7, 3.8 Hz, 1H), 4.99 (dd, *J* = 6.1, 4.7 Hz, 1H), 4.66 (dt, *J* = 8.3, 3.0 Hz, 1H), 4.45 (td, *J*

= 6.5, 1.2 Hz, 2H), 4.39 (td, *J* = 4.3, 2.5 Hz, 1H), 4.22 (p, *J* = 2.8 Hz, 1H), 4.15 (dd, *J* = 10.3, 2.9 Hz, 1H), 4.04 (d, *J* = 3.8 Hz, 1H), 3.98 (dd, *J* = 10.2, 3.2 Hz, 1H), 3.94 – 3.87 (m, 1H), 3.78 (ddd, *J* = 12.4, 8.5, 2.5 Hz, 1H), 3.16 (t, *J* = 6.3 Hz, 2H), 0.88 (s, 9H), 0.79 (s, 9H), 0.07 (s, 3H), 0.02 (s, 3H), -0.08 (s, 3H), -0.23 (s, 3H); **^{13}C NMR (101 MHz, Acetone-*d*$_6$) δ**: 171.0, 154.1, 151.4, 151.0, 147.4, 144.1, 131.1, 124.2, 122.1, 90.5, 87.8, 76.8, 72.6, 65.7, 64.3, 63.0, 56.4, 35.3, 27.2, 26.1, 26.0, 18.7, 18.6, -4.9, -5.2, -5.3, -5.6; **HRMS (ESI)**: calculated for $C_{34}H_{54}N_7O_{10}Si_2Na^+$: m/z = 798.3290 [M+Na]$^+$; found: m/z = 798.3288 [M+Na]$^+$.

General procedure for DMT protection:

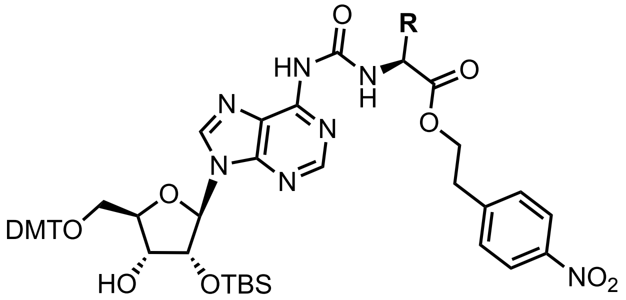

The 3'-5'-deprotected adenosine derivative (1 eq.) was dissolved in pyridine under N_2 atmosphere. DMT chloride (1.5 eq.) was added in two portions and the mixture was stirred at room temperature overnight. Then the volatiles were evaporated, and crude product was purified by flash chromatography eluting with CH_2Cl_2/MeOH (10/1/, v/v) with an addition of 0.1% of pyridine, unless otherwise specified, to afford the DMT protected derivative as white foam.

Compound 39

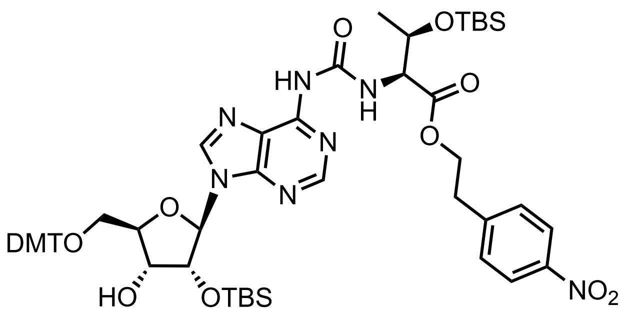

Yield: 90%; **IR**: $\tilde{\nu}$ = 3350 (w), 2930 (w), 2856 (w), 1729 (w), 1684 (s), 1608 (s), 1521 (vs), 1464 (s), 1345 (vs), 1248 (vs), 1174 (w), 1094 (w), 10033 (s), 827 (vs), 777 (vs) cm^{-1}; **^{1}H NMR (400 MHz, Acetone-*d*$_6$) δ**: 10.00 (d, *J* = 9.0 Hz, 1H), 8.95 (s, 1H), 8.55 (s, 1H), 8.40 (s, 1H), 7.98 (d, *J* = 8.7 Hz, 2H), 7.59 – 7.46 (m, 4H), 7.35 (dd, *J* = 9.0, 3.0 Hz, 4H), 7.28 – 7.13 (m, 4H), 6.83

(dd, J = 9.0, 3.0 Hz, 4H), 6.16 (d, J = 4.6 Hz, 1H), 5.16 (t, J = 4.6 Hz, 1H), 4.61 – 4.48 (m, 3H), 4.45 – 4.35 (m, 2H), 4.33 – 4.26 (m, 1H), 3.99 (d, J = 5.8 Hz, 1H), 3.75 (s, 6H), 3.51 – 3.44 (m, 2H), 3.12 (t, J = 6.2 Hz, 2H), 1.27 (d, J = 6.2 Hz, 3H), 0.91 (s, 9H), 0.85 (s, 9H), 0.09 (s, 3H), 0.06 (s, 3H), -0.05 (s, 6H); **^{13}C NMR (101 MHz, Acetone-*d*$_6$) δ**: 171.6, 159.6, 154.8, 151.5, 151.3, 147.5, 146.1, 136.7, 131.1, 130.9, 129.7, 129.0, 128.5, 127.5, 126.1, 124.1, 121.9, 113.8, 90.2, 87.1, 84.8, 76.3, 71.9, 69.6, 65.6, 64.4, 60.3, 55.5, 35.3, 26.1, 25.9, 21.5, 18.7, 18.5, -4.1, -4.6, -4.8, -5.2; **HRMS (ESI)**: calculated for $C_{56}H_{74}N_7O_{12}Si_2^+$: m/z = 1092.4929 [M+H]$^+$; found: m/z = 1092.4937 [M+H]$^+$.

Compound 40

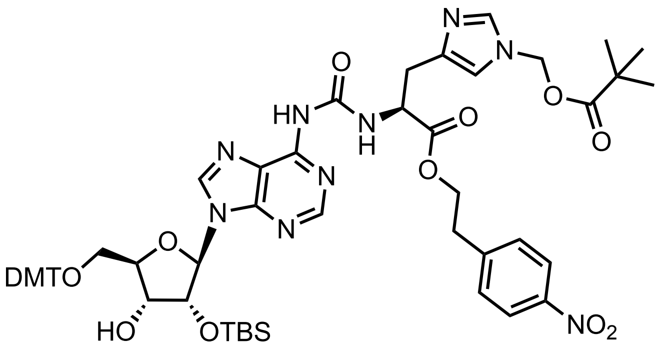

Eluent: 10% CH_2Cl_2 in EtOAc to pure EtOAc containing 0.1% of pyridine.

Yield: 85%; **IR**: $\tilde{\nu}$ = 3854 (w), 3746 (w), 3650 (w), 3630 (w), 2931 (w), 2361 (w), 2341 (w), 1740 (s), 1700 (vs), 1654 (w), 1609 (s), 1587 (w), 1559 (w), 1508 (vs), 1472 (s), 1396 (w), 1345 (s), 1300 (w), 1250 (vs), 1176 (s), 1118 (vs), 1032 (vs), 986 (w), 912 (w), 835 (vs), 781 (s), 751 (w), 700 (w) cm^{-1}; **^{1}H NMR (400 MHz, Acetone-*d*$_6$) δ**: 10.11 – 10.06 (m, 1H), 8.61 – 8.55 (m, 1H), 8.47 (s, 1H), 8.45 (s, 1H), 8.13 – 8.05 (m, 2H), 7.71 (d, J = 1.4 Hz, 1H), 7.63 – 7.55 (m, 2H), 7.53 – 7.46 (m, 2H), 7.40 – 7.32 (m, 4H), 7.28 (td, J = 8.2, 7.7, 2.0 Hz, 2H), 7.25 – 7.18 (m, 1H), 7.06 (s, 1H), 6.88 – 6.81 (m, 4H), 6.13 (d, J = 4.4 Hz, 1H), 5.88 (s, 2H), 5.10 (t, J = 4.6 Hz, 1H), 4.75 (dq, J = 8.0, 4.2, 3.3 Hz, 1H), 4.56 – 4.51 (m, 1H), 4.47 – 4.36 (m, 2H), 4.30 – 4.25 (m, 1H), 4.03 – 3.93 (m, 1H), 3.77 (s, 3H), 3.76 (s, 3H), 3.50 – 3.40 (m, 2H), 3.16 – 3.11 (m, 2H), 3.12 – 3.04 (m, 2H), 1.05 (s, 9H), 0.86 (s, 9H), 0.06 (s, 3H), -0.04 (s, 3H); **^{13}C NMR (101 MHz, Acetone-*d*$_6$) δ**: 177.9, 172.1, 159.6, 153.9, 151.7, 151.3, 147.7, 147.6, 146.1, 143.3, 139.2, 138.8, 136.7, 131.1, 131.0, 130.0, 129.0, 128.6, 127.6, 124.6, 124.1, 118.2, 113.9, 90.1, 87.1, 84.7, 76.4, 71.9, 71.8, 68.8, 66.2, 64.4, 55.5, 39.2, 35.3, 31.0, 27.0, 26.1, 18.7, -4.6, -4.8; **HRMS (ESI)**: calculated for $C_{58}H_{70}N_9O_{13}Si^+$: m/z = 1128.4857 [M+H]$^+$; found: m/z = 1128.4885 [M+H]$^+$.

Compound 41

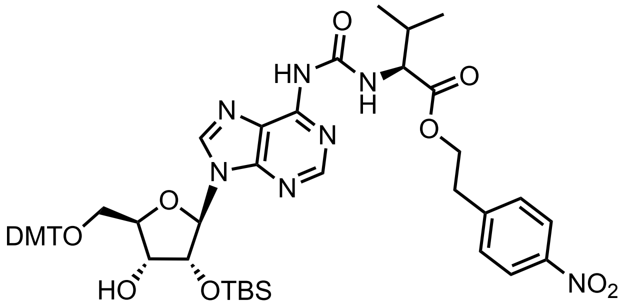

Yield: 70%; **IR**: $\tilde{\nu}$ = 2929 (w), 1735 (w), 1684 (w), 1569 (s), 1508 (vs), 1464 (s), 1344 (s), 1249 (vs), 1176 (w), 1015 (s), 800 (vs) 749 (s) cm^{-1}; **^{1}H NMR (400 MHz, Acetone-*d_6*) δ**: 10.02 (d, *J* = 9.0 Hz, 1H), 8.55 (s, 1H), 8.51 (s, 1H), 8.11 (d, *J* = 8.7 Hz, 2H), 7.59 (d, *J* = 8.7 Hz, 2H), 7.52 – 7.47 (m, 2H), 7.37 (dd, *J* = 9.0, 2.2 Hz, 4H), 7.30 – 7.13 (m, 4H), 6.86 – 6.82 (m, 4H), 6.16 (d, *J* = 4.6 Hz, 1H), 5.16 (t, *J* = 4.6 Hz, 1H), 4.57 – 4.39 (m, 5H), 4.30 (q, *J* = 4.6 Hz, 1H), 4.00 (d, *J* = 5.8 Hz, 1H), 3.76 (s, 6H), 3.47 (d, *J* = 4.2 Hz, 2H), 3.16 (t, *J* = 6.5 Hz, 2H), 2.31 – 2.30 (m, 1H), 0.99 (d, *J* = 6.5 Hz, 3H), 0.95 (d, *J* = 6.5 Hz, 3H), 0.85 (s, 9H), 0.06 (s, 3H), -0.04 (s, 3H); **^{13}C NMR (101 MHz, Acetone-*d_6*) δ**: 172.2, 159.5, 154.3, 151.6, 151.3, 147.4, 146.1, 143.5, 136.6, 131.0, 130.0, 128.9, 128.9, 127.5, 126.1, 124.2, 121.7, 113.8, 90.3, 87.0, 84.7, 76.4, 71.9, 65.2, 64.3, 59.5, 55.4, 35.3, 31.5, 26.1, 19.6, 18.7, 18.2, -4.6, -4.8; **HRMS (ESI)**: calculated for $C_{51}H_{62}N_{7}O_{11}Si^{+}$: m/z = 976.4277 $[M+H]^{+}$; found: m/z = 976.4287 $[M+H]^{+}$.

Compound 42

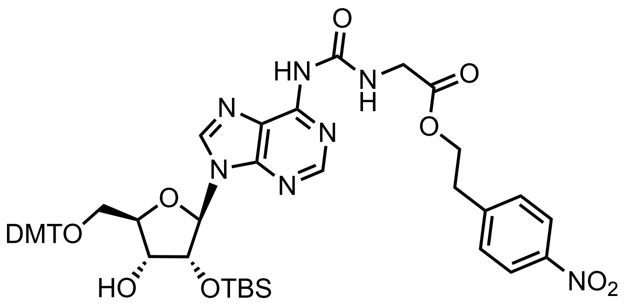

Yield: 85%; **IR**: $\tilde{\nu}$ = 2931 (w), 1748 (w), 1703 (s), 1609 (s), 1588 (w), 1509 (s), 1469 (s), 1345 (vs), 1250 (vs), 1177 (vs), 1035 (w), 835 (s) cm^{-1}; **^{1}H NMR (400 MHz, Acetone-*d_6*) δ**: 9.93 (t, *J* = 5.5 Hz, 1H), 9.05 (br. s, 1H), 8.58 (s, 1H), 8.46 (s, 1H), 8.11 (d, *J* = 8.7 Hz, 2H), 7.58 (d, *J* = 8.7 Hz, 2H), 7.54 – 7.47 (m, 2H), 7.41 – 7.33 (m, 4H), 7.31 – 7.23 (m, 2H), 7.23 – 7.16 (m, 1H), 6.88 – 6.78 (m, 4H), 6.16 (d, *J* = 4.4 Hz, 1H), 5.10 (dd, *J* = 4.7 Hz, 1H), 4.53 (t, *J* = 5.1 Hz, 1H), 4.44 (t, *J* = 6.4 Hz, 2H), 4.30 (dt, *J* = 4.4 Hz, 1H), 4.13 (d, *J* = 5.5 Hz, 2H), 4.00 (d, *J* = 5.7 Hz,

1H), 3.75 (s, 6H), 3.53 – 3.42 (m, 2H), 3.13 (t, *J* = 6.4 Hz, 2H), 0.85 (s, 9H), 0.05 (s, 3H), -0.05 (s, 3H); **^{13}C NMR (101 MHz, Acetone-*d₆*) δ**: 170.6, 159.6, 154.6, 151.7, 151.3, 151.2, 147.6, 147.4, 146.1, 143.6, 136.7, 131.1, 130.9, 129.1, 129.0, 128.6, 127.5, 124.2, 121.7, 113.8, 90.0, 87.1, 84.8, 76.4, 72.0, 65.2, 64.4, 55.5, 42.6, 35.3, 26.1, 18.7, -4.7, -4.8; **HRMS (ESI)**: calculated for $C_{48}H_{55}N_7O_{11}Si^+$: m/z = 934.3802 [M + H]$^+$; found: m/z = 934.3812 [M + H]$^+$.

Compound 43

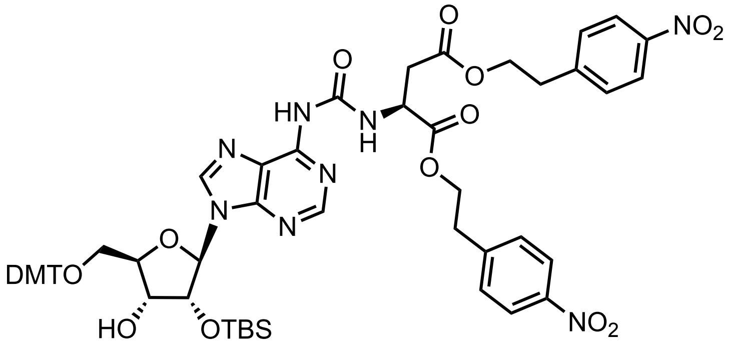

Yield: 95%; **IR**: $\tilde{\nu}$ = 2930 (w), 1734 (s), 1698 (s), 1607 (s), 1509 (vs), 1466 (s), 1370 (vs), 1248 (s), 1174 (s), 1032 (s), 829 (vs), 699 (s) cm^{-1}; **^{1}H NMR (400 MHz, Acetone-*d₆*) δ**: 10.29 (d, *J* = 7.9 Hz, 1H), 9.18 (s, 1H), 8.60 (s, 1H), 8.42 (s, 1H), 8.10 – 8.04 (m, 4H), 7.58 – 7.51 (m, 4H), 7.39 – 7.36 (m, 4H), 7.30 – 7.25 (m, 2H), 7.21 – 7.18 (m, 2H), 6.86 – 6.82 (m, 5H), 6.17 (d, *J* = 4.2 Hz, 1H), 5.10 (t, *J* = 4.5 Hz, 1H), 4.85 (dt, *J* = 7.9, 5.2 Hz, 1H), 4.56 (q, *J* = 5.2 Hz, 1H), 4.46 – 4.28 (m, 5H), 4.02 (d, *J* = 5.9 Hz, 1H), 3.75 (d, *J* = 2.1 Hz, 6H), 3.53 – 3.44 (m, 2H), 3.13 – 3.06 (m, 4H), 3.00 – 2.96 (m, 2H), 0.86 (s, 9H), 0.07 (s, 3H), 0.03 (s, 3H); **^{13}C NMR (101 MHz, Acetone-*d₆*) δ**: 171.3, 171.2, 159.5, 154.2, 151.5, 150.6, 147.4, 147.2, 146.1, 143.6, 136.7, 131.0, 130.0, 129.0, 128.6, 127.5, 124.2, 113.8, 90.1, 87.0, 84.6, 76.4, 71.8, 65.7, 65.1, 64.3, 55.4, 50.6, 37.1, 35.2, 26.1, 18.6, -4.6, -4.8; **HRMS (ESI)**: calculated for $C_{58}H_{64}N_8O_{15}SiNa^+$: m/z = 1163.4158 [M+Na]$^+$; found: m/z = 1163.4159 [M+Na]$^+$.

Compound 44a

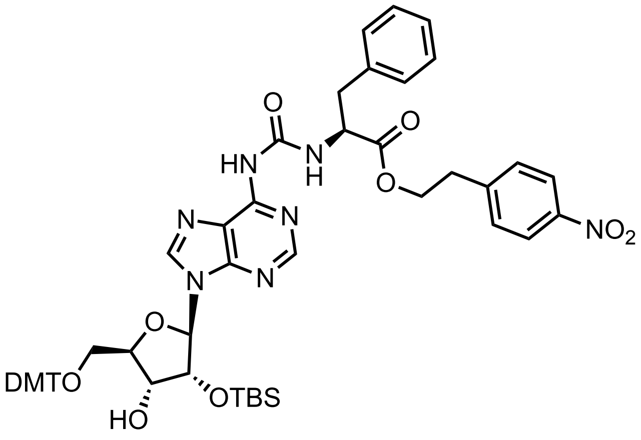

Eluent: CH_2Cl_2/MeOH (20/1, v/v).

Yield: 82%; **IR**: $\tilde{\nu}$ = 3245 (w), 2952 (w), 2931 (w), 1741 (w), 1699 (s), 1608 (s), 1587 (w), 1519 (s), 1509 (s), 1469 (w), 1345 (s), 1249 (s), 1176 (s), 1034 (w), 834 (s), 783 (w), 735 (vs), 701 (vs) cm^{-1}; **^{1}H NMR (400 MHz, CD_2Cl_2) δ**: 9.83 (d, *J* = 7.5 Hz, 1H), 8.29 (s, 1H), 8.17 (s, 1H), 8.06 (d, *J* = 8.7 Hz, 2H), 8.00 (s, 1H), 7.46 (d, *J* = 6.8 Hz, 2H), 7.38 – 7.32 (m, 7H), 7.31 – 7.20 (m, 7H), 7.18 – 7.14 (m, 2H), 6.82 (d, *J* = 8.9 Hz, 4H), 6.02 (d, *J* = 5.0 Hz, 1H), 5.02 (t, *J* = 5.0 Hz, 1H), 4.80 (q, *J* = 6.3 Hz, 1H), 4.44 – 4.32 (m, 3H), 4.24 (q, *J* = 3.8 Hz, 1H), 3.77 (s, 6H), 3.48 (dd, *J* = 10.7 Hz, *J* = 3.2 Hz, 1H), 3.39 (dd, *J* = 10.7 Hz, *J* = 4.3 Hz, 1H), 3.16 (d, *J* = 6.3 Hz, 2H), 3.03 (t, *J* = 6.5 Hz, 2H), 2.67 (d, *J* = 4.7 Hz, 1H), 0.85 (s, 9H), 0.01 (s, 3H), -0.12 (s, 3H); **^{13}C NMR (101 MHz, CD_2Cl_2) δ**: 171.5, 153.3, 150.8, 150.6, 149.8, 149.4, 146.8, 145.8, 143.6, 136.2, 135.9, 129.9, 129.4, 128.5, 128.3, 127.2, 123.7, 123.6, 121.8, 91.0, 87.5, 74.8, 72.7, 64.8, 63.1, 54.8, 37.8, 34.7, 25.3, 17.7, -5.7; **HRMS (ESI)**: calculated for $C_{55}H_{62}N_7O_{11}Si^+$: m/z = 1024.4271 $[M+H]^+$; found: m/z = 1024.429 $[M+H]^+$.

Compound 44b

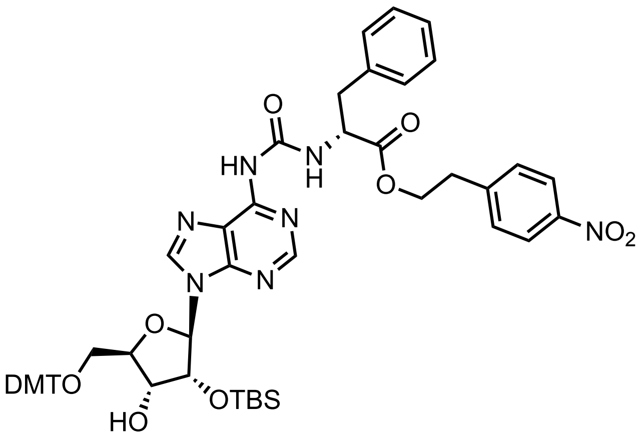

Eluent: CH_2Cl_2/MeOH (20/1, v/v).

Yield: 85%; **IR**: $\tilde{\nu}$ = 3231 (w), 2930 (w), 2858 (w), 1741 (w), 1697 (s), 1612 (s), 1587 (w), 1518 (vs), 1470 (s), 1344 (vs), 1253 (w), 1180 (w), 1134 (w), 1085 (w), 836 (s), 780 (s), 734 (vs), 698 (vs) cm^{-1}; **^{1}H NMR (400 MHz, CD_2Cl_2) δ**: 9.83 (d, *J* = 7.5 Hz, 1H), 8.58 (br. s, 1H), 8.32 (s, 1H), 8.19 (s, 1H), 8.06 (d, *J* = 8.7 Hz, 2H), 7.99 (s, 1H), 7.46 (d, *J* = 6.9 Hz, 2H), 7.38 – 7.31 (m, 7H), 7.31 – 7.21 (m, 7H), 7.20 – 7.16 (m, 2H), 6.82 (d, *J* = 8.9 Hz, 4H), 6.05 (d, *J* = 4.7 Hz, 1H), 4.94 (t, *J* = 5.0 Hz, 1H), 4.81 (q, *J* = 6.3 Hz, 1H), 4.44 – 4.33 (m, 3H), 4.24 (q, *J* = 3.8 Hz, 1H), 3.77 (s, 6H), 3.48 (dd, *J* = 10.7 Hz, 3.1 Hz, 1H), 3.41 (dd, *J* = 10.7 Hz, 4.2 Hz, 1H), 3.16 (d, *J* = 6.9 Hz, 2H), 3.03 (t, *J* = 6.5 Hz, 2H), 2.66 (d, *J* = 5.0 Hz, 1H), 0.86 (s, 9H), 0.02 (s, 3H), -0.09 (s, 3H); **^{13}C NMR (101 MHz, CD_2Cl_2) δ**: 171.4, 153.4, 150.7, 150.5, 149.8, 149.4, 146.8, 145.9, 143.7, 136.3, 129.9, 129.5, 128.5, 128.3, 127.2, 123.5, 123.5, 121.8, 91.0, 87.5, 74.9, 72.7, 64.8, 63.1, 54.9, 37.8, 34.7, 25.3, 17.8, -5.6; **HRMS (ESI)**: calculated for $C_{55}H_{62}N_7O_{11}Si^+$: m/z = 1024.4271 $[M+H]^+$; found: m/z = 1024.4291 $[M+H]^+$.

Compound 45

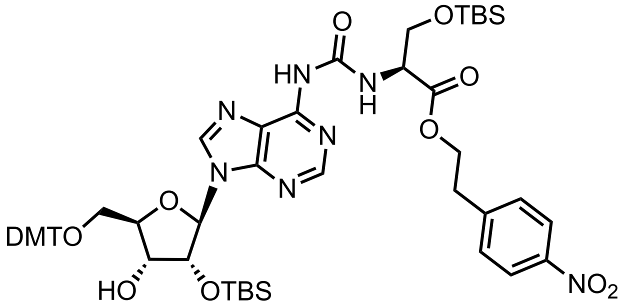

Yield: 90%; **IR**: $\tilde{\nu}$ = 2930 (w), 1740 (s), 1694 (s), 1607 (w), 1582 (w), 1519 (vs), 1465 (w), 1345 (s), 1249 (s), 1176 (w), 1110 (w), 1034 (w), 820 (vs), 778 (s), 699 (w) cm^{-1}; **^{1}H NMR (400 MHz, Acetone-*d*$_6$) δ**: 10.13 (d, *J* = 8.2 Hz, 1H), 8.99 (s, 1H), 8.58 (s, 1H), 8.44 (s, 1H), 8.09 (d, *J* = 8.7 Hz, 2H), 7.58 (d, *J* = 8.7 Hz, 2H), 7.51 (dd, *J* = 8.4, 1.3 Hz, 2H), 7.39 (d, *J* = 2.5 Hz, 2H), 7.37 (d, *J* = 2.4 Hz, 2H), 7.32 – 7.18 (m, 4H), 6.87 – 6.83 (m, 4H), 6.18 (d, *J* = 4.7 Hz, 1H), 5.13 (t, *J* = 5.0 Hz, 1H), 4.62 (dt, *J* = 8.2, 2.8 Hz, 1H), 4.53 (q, *J* = 5.0 Hz, 1H), 4.45 (t, *J* = 6.3 Hz, 2H), 4.30 (td, *J* = 4.6, 3.4 Hz, 1H), 4.14 (dd, *J* = 10.2, 2.8 Hz, 1H), 3.96 (dd, *J* = 10.2, 2.8 Hz, 1H), 3.77 (s, 6H), 3.48 (qd, *J* = 10.5, 4.1 Hz, 2H), 3.16 (t, *J* = 6.3 Hz, 2H), 0.89 (s, 9H), 0.85 (s, 9H), 0.07 (s, 3H), 0.06 (s, 3H), 0.03 (s, 3H), -0.06 (s, 3H); **^{13}C NMR (101 MHz, Acetone-*d*$_6$) δ**: 170.0, 159.5, 154.1, 151.6, 150.6, 147.4, 146.1, 143.6, 136.7, 131.1, 130.9, 129.0, 128.6, 127.6, 124.6, 124.2, 121.8, 113.8, 89.9, 87.1, 84.9, 76.5, 72.0, 65.8, 64.3, 56.4, 55.4, 35.3, 27.2, 26.1, 18.7, 18.4, -4.6, -4.9, -5.3, -5.5; **HRMS (ESI)**: calculated for $C_{55}H_{71}N_7O_{12}Si_2Na^+$: m/z = 1100.4597 $[M+Na]^+$; found: m/z = 1100.4620 $[M+Na]^+$.

General synthesis of RNA phosphoramidites

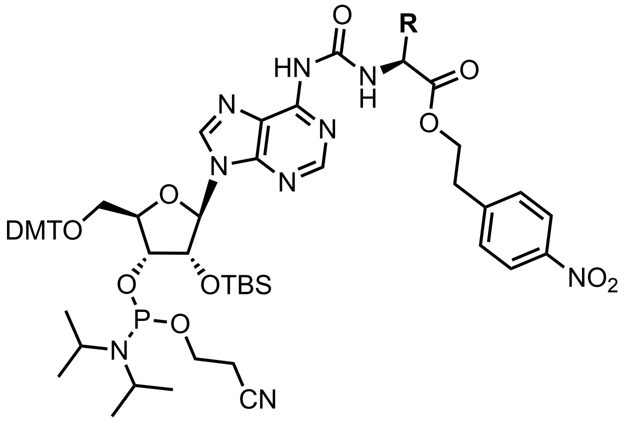

In an oven-dried flask under argon atmosphere, 5'-DMT protected compound (1 eq.) was dissolved in CH_2Cl_2 and cooled to 0 °C. Hünig's base (4 eq.) was added dropwise followed by the addition of 2-cyanoethyl *N,N*-diisopropylchlorophosphoramidite (2.5 eq.). The solution was stirred at room temperature for 2 h. The reaction was quenched by addition of sat. $NaHCO_3$ solution, and then extracted three times with CH_2Cl_2. The organic phase was dried over Na_2SO_4, filtered and concentrated in vacuo. The residue was purified by flash chromatography, eluting with Hex/EtOAc (1/1, v/v) containing 0.1% of pyridine, unless otherwise specified. The phosphoramidite was obtained as a mixture of diastereomers as white foam.

Compound 46

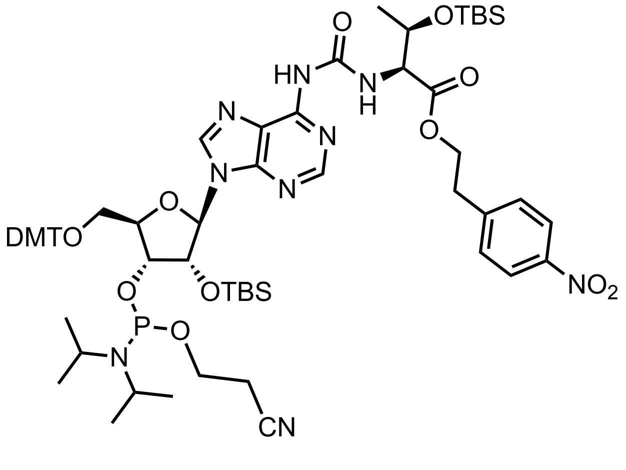

Yield: 85%; **^{31}P NMR (162 MHz, Acetone-*d*$_6$) δ**: 150.16, 148.45; **HRMS (ESI)**: calculated for $C_{65}H_{91}N_9O_{13}PSi_2^+$: m/z = 1292.6007 [M+H]$^+$; found: m/z = 1292.6033 [M+H]$^+$.

Compound 47

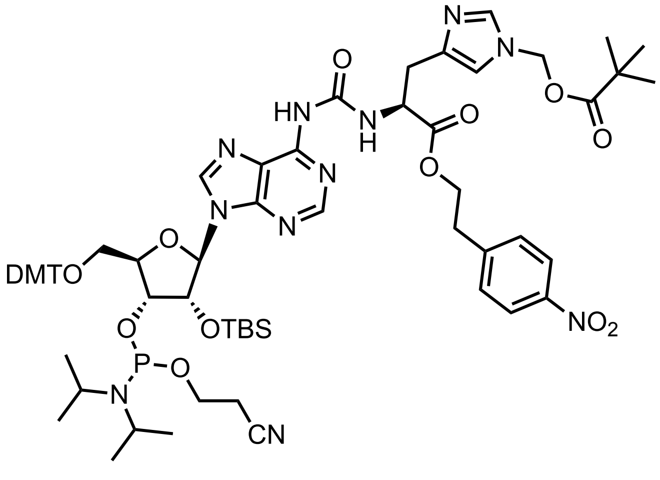

Eluent: 30% CH_2Cl_2 in EtOAc containing 0.1% of pyridine.

Yield: 66%; **^{31}P NMR (162 MHz, Acetone-*d*$_6$) δ**: 150.26, 148.61; **HRMS (ESI)**: calculated for $C_{67}H_{87}N_{11}O_{14}PSi^+$: m/z = 1328.5935 [M+H]$^+$; found: m/z = 1328.5944 [M+H]$^+$.

Compound 48

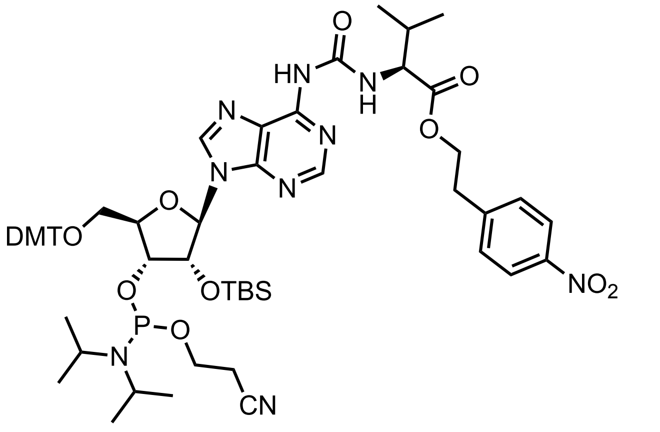

Yield: 87%; **^{31}P NMR (162 MHz, Acetone-*d*$_6$) δ**: 150.22, 148.65; **HRMS (ESI)**: calculated for $C_{60}H_{79}N_9O_{12}PSi^+$: m/z = 1176.5355 [M+H]$^+$; found: m/z = 1176.5359 [M+H]$^+$.

Compound 49

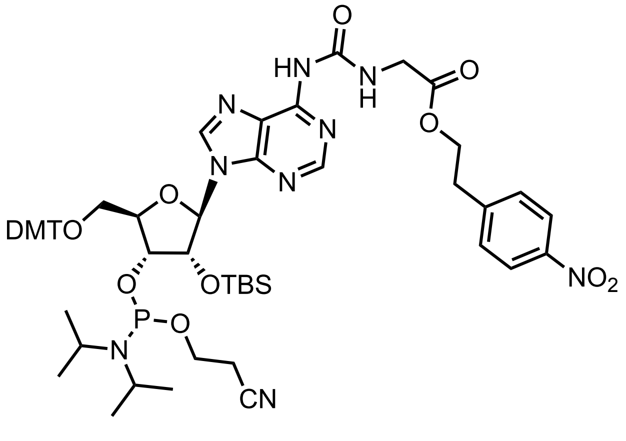

Yield: 86%; **^{31}P NMR (162 MHz, Acetone-*d$_6$*) δ**: 150.32, 148.60; **HRMS (ESI)**: calculated for $C_{57}H_{72}N_9O_{12}SiPSi^+$: m/z = 1134.4880 [M+H]$^+$; found: m/z = 1134.4894 [M + H]$^+$.

Compound 50

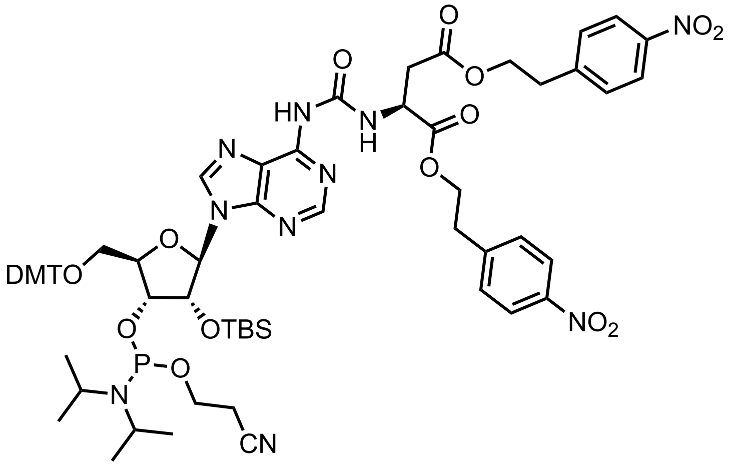

Yield: 65%; **^{31}P NMR (162 MHz, Acetone-*d$_6$*) δ**: 150.15, 148.67; **HRMS (ESI)**: calculated for $C_{67}H_{82}N_{10}O_{16}PSi^+$: m/z = 1341.5417 [M+H]$^+$; found: m/z = 1341.5437 [M+H]$^+$.

Compound 51a

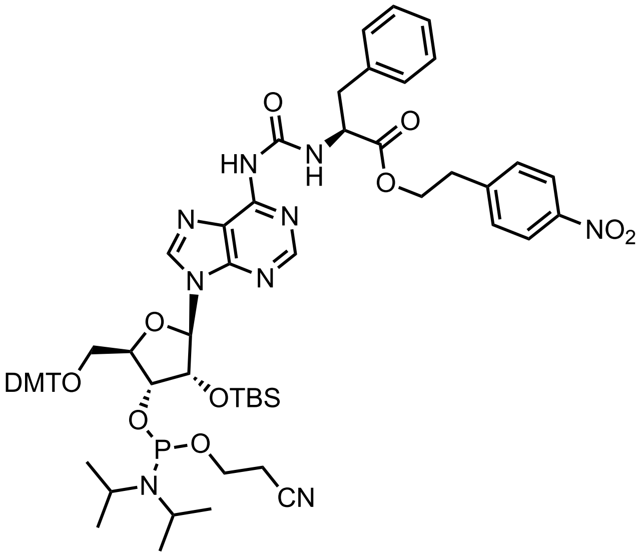

Yield: 67%; **^{31}P NMR (162 MHz, CD_2Cl_2) δ**: 150.7, 149.1; **HRMS (ESI)**: calculated for $C_{64}H_{79}N_9O_{12}PSi^+$: m/z = 1224.5350 $[M+H]^+$; found: m/z = 1224.5374 $[M+H]^+$.

Compound 51b

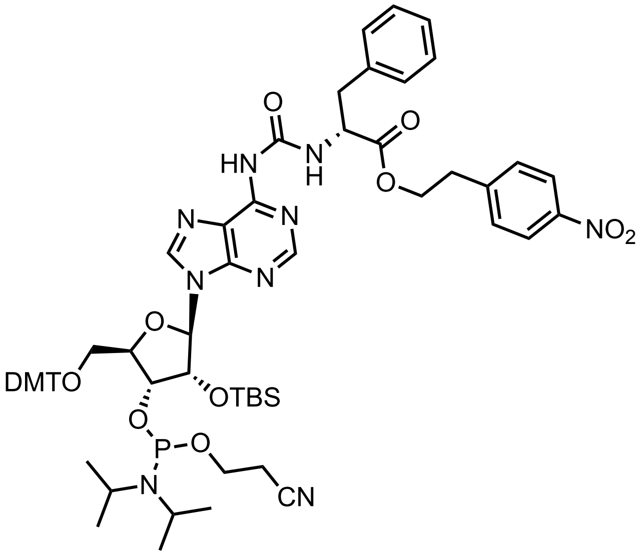

Yield: 67%; **^{31}P NMR (162 MHz, CD_2Cl_2) δ**: 150.6, 148.9; **HRMS (ESI)**: calculated for $C_{64}H_{79}N_9O_{12}PSi^+$: m/z = 1224.5350 $[M+H]^+$; found: m/z = 1224.5383 $[M+H]^+$.

Compound 52

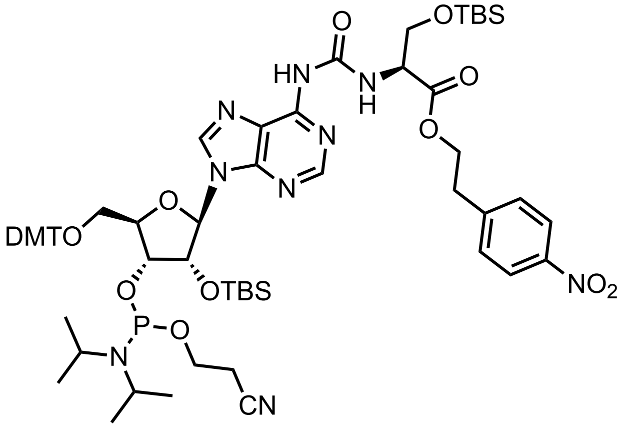

Yield: 70%; **^{31}P NMR (162 MHz, Acetone-*d*$_6$) δ**: 150.34, 148.45; **HRMS (ESI)**: calculated for $C_{64}H_{89}N_9O_{13}PSi_2^+$: m/z = 1278.5856 [M+H]$^+$; found: m/z = 1278.5877 [M+H]$^+$.

2.2. Synthesis of DNA building block

Compound 54

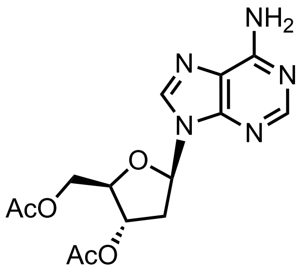

The compound was synthesized according to the published procedure.[5]

Acetic anhydride (1.1 ml, 11.5 mmol, 6.2 eq.) was added to a mixture of deoxyadenosine monohydrate **53** (0.5 g, 1.85 mmol, 1 eq.), pyridine (7 ml) and 4-(dimethylamino)pyridine (25 mg, 0.4 mmol, 0.1 eq.). The reaction mixture was stirred at room temperature for 4 hours. Subsequently, iced water was added, and the mixture was concentrated and co-evaporated with toluene. The compound was used for further steps without additional purification.

Yield: 99%; **^{1}H NMR (400 MHz, CDCl$_3$) δ**: 8.23 (s, 1H), 7.92 (s, 1H), 7.26 (s, 2H), 6.34 (dd, *J* = 7.9, 6.0 Hz, 1H), 5.34 – 5.32 (m, 1H), 4.32 – 4.24 (m, 3H), 2.87 – 2.80 (m, 1H), 2.57 – 2.52 (m, 1H), 2.04 (s, 3H), 2.00 (s, 3H). **HRMS (ESI)**: calculated for $C_{14}H_{18}N_5O_5^+$: m/z = 336.1302 [M+H]$^+$; found: m/z = 336.1305 [M+H]$^+$.

The analytical data is in agreement with the literature.[5]

Compound 55

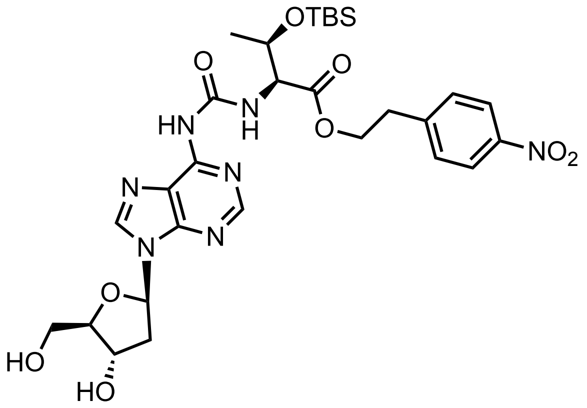

The acetyl-protected deoxyadenosine derivative **54** (0.5 g, 1.5 mmol, 1 eq.) was dissolved in dry CH_2Cl_2 under nitrogen atmosphere. 1-*N*-methyl-3-phenoxycarbonyl-imidazolium chloride (**22**; 0.71 g, 3 mmol, 2 eq.) was added to the reaction mixture and the resulting suspension was stirred at room temperature for 2 hours (the solution in time becomes clear). Afterwards the protected threonine derivative **7** (1.1 g, 3 mmol, 2 eq.) was added together with TEA (415 µl, 3 mmol, 2 eq.) as a solution in CH_2Cl_2 and the resulting solution was stirred overnight at room temperature. The reaction was quenched by addition of saturated aqueous $NaHCO_3$ solution. The solution was extracted three times with CH_2Cl_2, and the organic phase was dried, filtered and concentrated *in vacuo*. The acetyl groups were immediately deprotected with 7N NH_3/MeOH (2 ml). After stirring 2 hours at room temperature, the reaction mixture was evaporated. The residue was purified via flash chromatography eluting with CH_2Cl_2/MeOH (10/1, v/v).

Yield: 65%; **IR**: $\tilde{\nu}$ = 3227 (w), 2929 (w), 2855 (w), 1734 (w), 1686 (vs), 1610 (s), 1587 (s), 1518 (vs), 1465 (vs), 1344 (vs), 1312 (w), 1248 (s), 1213 (w), 1094 (vs), 939 (s), 827 (vs) cm^{-1}; **^1H NMR (400 MHz, Acetone-d_6) δ**: 10.02 (d, *J* = 8.6 Hz, 1H), 9.40 (s, 1H), 8.70 (s, 1H), 8.39 (s, 1H), 7.91 (d, *J* = 8.2 Hz, 2H), 7.47 (d, *J* = 8.2 Hz, 2H), 6.54 (t, *J* = 6.7 Hz, 1H), 4.67 (s, 1H), 4.51 (d, *J* = 9.3 Hz, 2H), 4.38 (t, *J* = 5.6 Hz, 2H), 4.09 (s, 1H), 3.86 – 3.67 (m, 2H), 3.08 (t, *J* = 5.6 Hz, 2H), 2.90 (dt, *J* = 13.0, 6.7 Hz, 1H), 2.52 – 2.41 (m, 1H), 2.01 (s, 1H), 1.93 (s, 1H), 1.23 (d, *J* = 5.9 Hz, 3H), 0.88 (s, 9H), 0.05 (s, 3H), -0.06 (s, 3H); **^{13}C NMR (101 MHz, Acetone-d_6) δ**: 171.5, 155.1, 151.1, 150.9, 147.4, 144.2, 130.9, 124.0, 122.0, 89.8, 86.8, 72.6, 69.5, 65.5, 63.3, 41.4, 35.2, 30.6, 26.0, 21.5, 20.5, 18.4, -4.2, -5.2; **HRMS (ESI)**: calculated for $C_{29}H_{42}N_7O_9Si^+$: m/z = 660.2813 $[M+H]^+$; found: m/z = 660.2812 $[M+H]^+$.

Compound 56

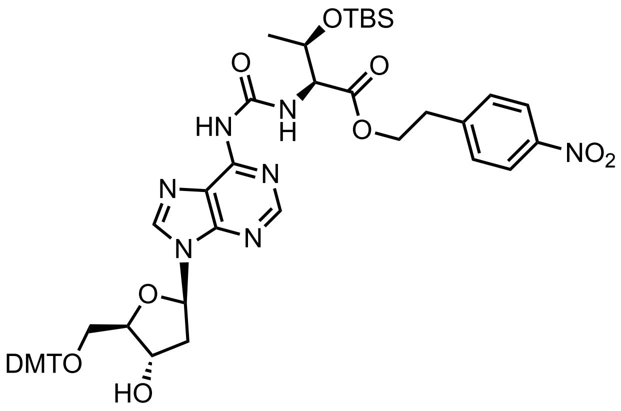

The 3'-5'-deprotected adenosine derivative **55** (0.34 g, 0.52 mmol, 1 eq.) was dissolved in pyridine under N_2 atmosphere. DMT chloride (0.26 g, 0.77 mmol, 1.5 eq.) was added in two portions and the mixture was stirred at room temperature overnight. Then the volatiles were evaporated, and crude product was purified by flash chromatography eluting with CH_2Cl_2/MeOH (10/1/, v/v) with an addition of 0.1% of pyridine to afford the DMT protected derivative.

Yield: 72%; **IR**: $\tilde{\nu}$ = 2960 (w), 2930 (w), 1734 (w), 1696 (s), 1609 (s), 1586 (w), 1520 (vs), 1509 (vs), 1467 (s), 1345 (vs), 1304 (w), 1250 (vs), 1175 (s), 1095 (w), 1034 (s), 940 (w), 828 (vs), 777 (s), 699 (w) cm^{-1}; **^{1}H NMR (400 MHz, Acetone-*d*$_6$) δ**: 10.02 (d, *J* = 9.1 Hz, 1H), 8.98 (s, 1H), 8.52 (s, 1H), 8.34 (s, 1H), 7.96 (d, *J* = 8.7 Hz, 2H), 7.51 (d, *J* = 8.7 Hz, 2H), 7.42 (d, *J* = 7.1 Hz, 2H), 7.31 – 7.28 (m, 4H), 7.24 – 7.12 (m, 3H), 6.79 – 6.74 (m, 4H), 6.55 (t, *J* = 6.4 Hz, 1H), 4.76 (q, *J* = 4.2 Hz, 1H), 4.64 – 4.58 (m, 1H), 4.55 (ddd, *J* = 10.6, 7.7, 1.5 Hz, 2H), 4.41 (t, *J* = 6.2 Hz, 2H), 4.25 – 4.18 (m, 1H), 3.74 (d, *J* = 3.3 Hz, 6H), 3.43 (dd, *J* = 10.2, 5.9 Hz, 1H), 3.35 (dd, *J* = 10.2, 4.0 Hz, 1H), 3.17 – 3.10 (m, 3H), 2.58 – 2.52 (m, 1H), 1.29 (d, *J* = 6.3 Hz, 3H), 0.93 (s, 9H), 0.11 (s, 3H), -0.01 (s, 3H); **^{13}C NMR (101 MHz, Acetone-*d*$_6$) δ**: 171.6, 159.4, 154.9, 151.2, 151.0, 147.4, 146.1, 143.9, 136.8, 131.0, 130.9, 130.8, 128.9, 128.4, 127.4, 124.0, 122.0, 113.7, 87.7, 86.8, 86.1, 72.6, 69.6, 65.4, 65.1, 60.3, 55.4, 40.0, 35.3, 26.0, 21.5, 18.4, -4.1, -5.2; **HRMS (ESI)**: calculated for $C_{50}H_{60}N_7O_{11}Si^+$: m/z = 962.4120 $[M+H]^+$; found: m/z = 962.4136 $[M+H]^+$.

Compound 57

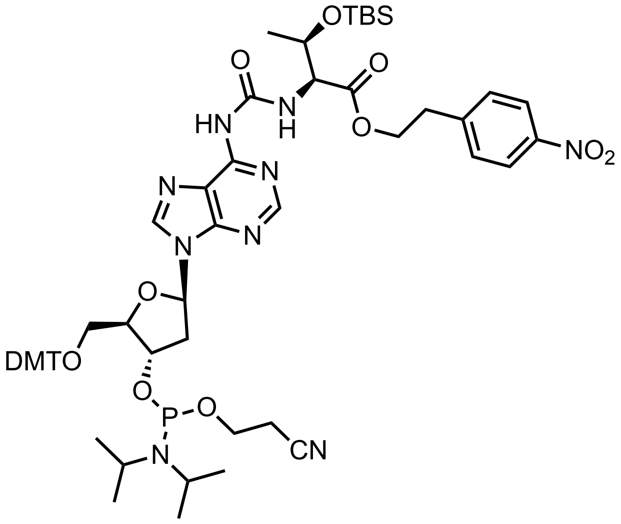

In an oven-dried flask under argon atmosphere, 5'-DMT protected compound **56** (0.1 g, 0.1 mmol, 1 eq.) was dissolved in CH_2Cl_2 and cooled to 0 °C. Hünig's base (72 µl, 0.4 mmol, 4 eq.) was added dropwise followed by the addition of 2-cyanoethyl *N,N*-diisopropylchlorophosphoramidite (60 µl, 0.25 mmol, 2.5 eq.). The solution was stirred at room temperature for 2 h. The reaction was quenched by addition of sat. $NaHCO_3$ solution, and then extracted three times with CH_2Cl_2. The organic phase was dried over Na_2SO_4, filtered and concentrated in vacuo. The residue was purified by flash chromatography, eluting with Hex/EtOAc (1/2, v/v) containing 0.1% of pyridine. The phosphoramidite was obtained as a mixture of diastereomers as white foam.

Yield: 62%; **^{31}P NMR (162 MHz, Acetone-*d*$_6$) δ**: 148.00, 146.59; **HRMS (ESI)**: calculated for $C_{59}H_{76}N_9NaO_{12}PSi^+$: m/z = 1184.5018 $[M+Na]^+$; found: m/z = 1184.5021 $[M+Na]^+$.

3. Synthesis and Purification of Oligonucleotides

All of the oligonucleotides used in this study were synthesized on a 1 µmol scale using a DNA automated synthesizer (Applied Biosystems 394 DNA/RNA Synthesizer) with standard phosphoramidite chemistry. The phosphoramidites of canonical ribonucleosides were purchased from Glen Research and Sigma-Aldrich. Oligonucleotides containing non-canonical nucleosides were synthesized in DMT-OFF mode using phosphoramidites (Bz-A, Dmf-G, Ac-C, U) with BTT in CH_3CN as an activator, DCA in CH_2Cl_2 as a deblocking solution and Ac_2O in pyridine/THF as a capping reagent. For deprotection of npe-group the solid support was treated with DBU solution (10% in THF, 1 ml) for 2 h at room temperature. Subsequently, the supernatant was removed, and the beads were washed with THF and dried under vacuum. The solid support was suspended in a mixture of aqueous ammonia and methylamine (1:1, 1 ml) at room temperature for 1 h. The supernatant was removed, and the beads were washed with water. The supernatant and washings were combined, and the solvents were evaporated under reduced pressure. The residue was subsequently heated with a solution of triethylamine trihydrofluoride (125 µl) in DMSO (50 µl) at 65 °C for 1.5 h. The RNA was precipitated by addition of aqueous NaOAc solution (3M, 25 µl) and *n*-butanol (1 ml). To ensure complete precipitation, the sample was incubated at -80 °C for 1 h. After centrifugation (4 °C, 4000 rpm, 15 min), the supernatant was removed, and the precipitated RNA was lyophilized. The oligonucleotides were further purified by reverse-phase HPLC using a Waters Breeze (2487 Dual λ Array Detector, 1525 Binary HPLC Pump) equipped with the column VP 250/32 C18 from Macherey Nagel. Oligonucleotides were purified using the following buffer system: buffer A: 100 mM NEt_3/HOAc, pH 7.0 in H_2O and buffer B: 100 mM NEt_3/HOAc in 80 % (v/v) acetonitrile. A flow rate of 5 ml/min with a gradient of 0-25 % of buffer B in 30 min was applied for the purifications. Analytical RP-HPLC was performed on an analytical HPLC Waters Alliance (2695 Separation Module, 2996 Photodiode Array Detector) equipped with the column Nucleosil 120-2 C18 from Macherey Nagel using a flow of 0.5 mL/min, a gradient of 0-30% of buffer B in 45 min was applied. Calculation of concentrations was assisted using the software OligoAnalyzer 3.0 (Integrated DNA Technologies: https://eu.idtdna.com/calc/analyzer). For strands containing non-canonical base, the extinction coefficient of their corresponding canonical-only strand was employed without corrections. The structural integrity of the synthesized oligonucleotides was analyzed by MALDI-TOF mass measurements.

Sequences of synthesized strands:

ON1: 5' GUCt^{6}ACCUGA 3'

ON2: 5' GUCg^{6}ACCUGA 3'

ON3: 5' GUCval^{6}ACCUGA 3'

ON4: 5' GUChis^{6}ACCUGA 3'

ON5: 5' GUCasp^{6}ACCUGA 3'

ON6: 5' GUC-L-phe^{6}ACCUGA 3'

ON7: 5' GUC-D-phe^{6}ACCUGA 3'

ON8: 5' AUCGt^{6}ACUACGt^{6}AAUCGCt^{6}AACCG 3'

ON9: 5' AGAUGUG-ser^{6}A-asp^{6}A-his^{6}A-GAGAUGA 3'

ODN1: 5' d(GTCt^{6}dACCTGA) 3'

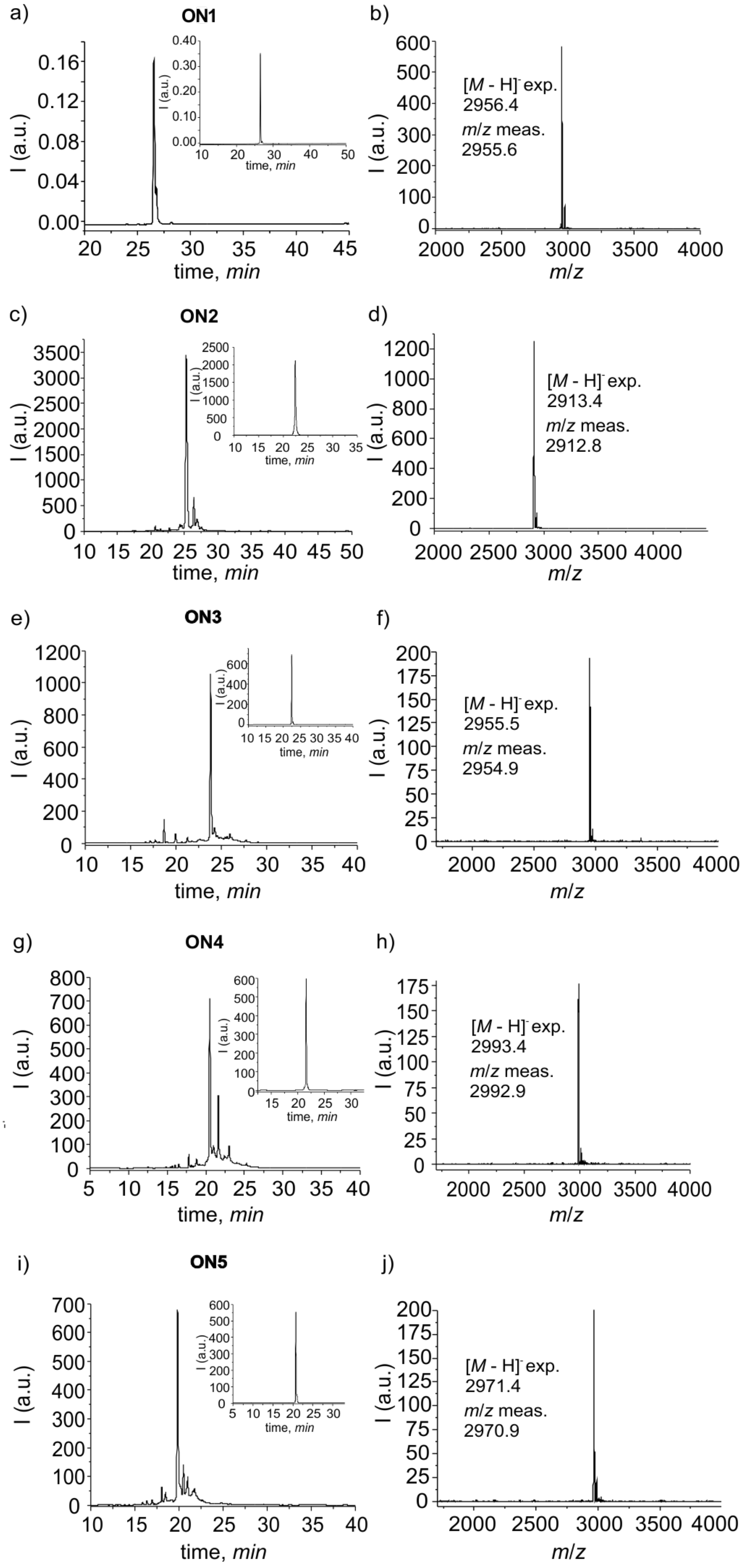

Figure S1. HPLC and MALDI data of oligonucleotides **ON1**-**ON5**.

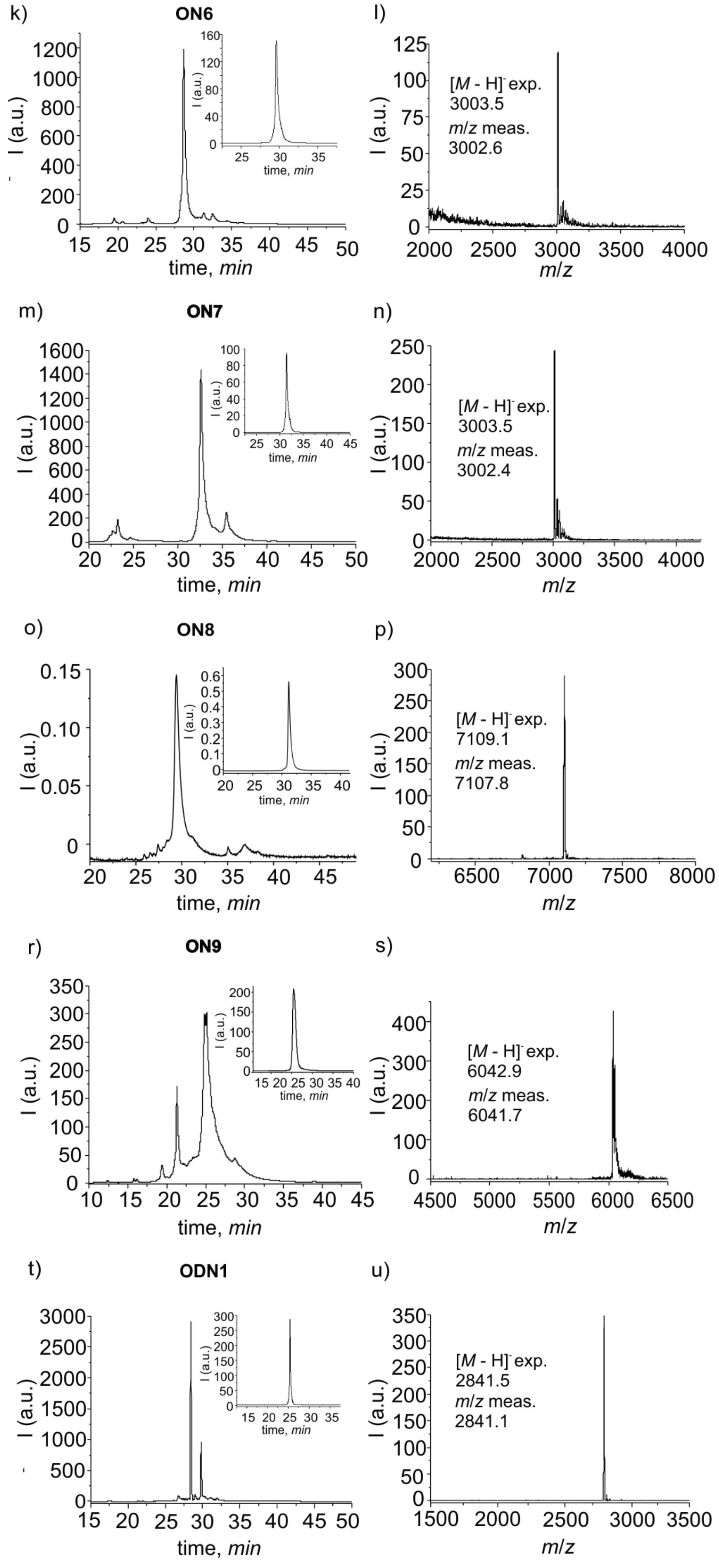

Figure S2. HPLC and MALDI data of oligonucleotides **ON6**-**ODN1**.

4. UV Melting Curve Measurements

The UV melting curves were measured on JASCO V-650 spectrometer using 10 mm QS cuvettes, purchased from Hellma Analytics. A solution (80 μL) of equimolar amounts of oligonucleotides (4 μM each) in the buffer solution containing 10 mM sodium phosphate buffer (pH 7.0) and 150 mM NaCl was heated at 90 °C for 2 min and gradually cooled to 4 °C prior to the measurement. Melting profiles were recorded at temperatures between 5 and 75 °C with a ramping and scanning rate of 1 °C/min at 260 nm. All samples were measured at least three times. T_m values from each measurement were calculated using the "fitting curve" method and presented as an average of three independent measurements.

5. NMR spectra

Compound 5

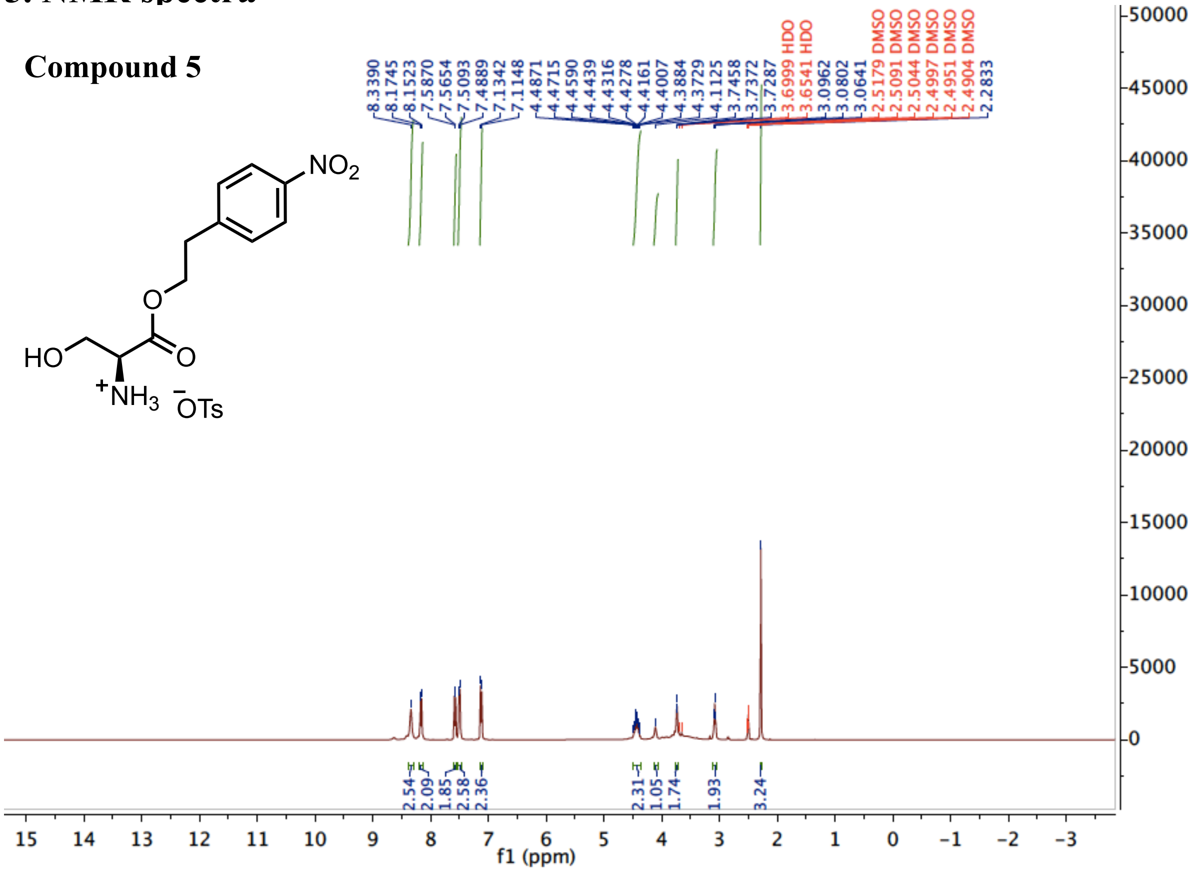

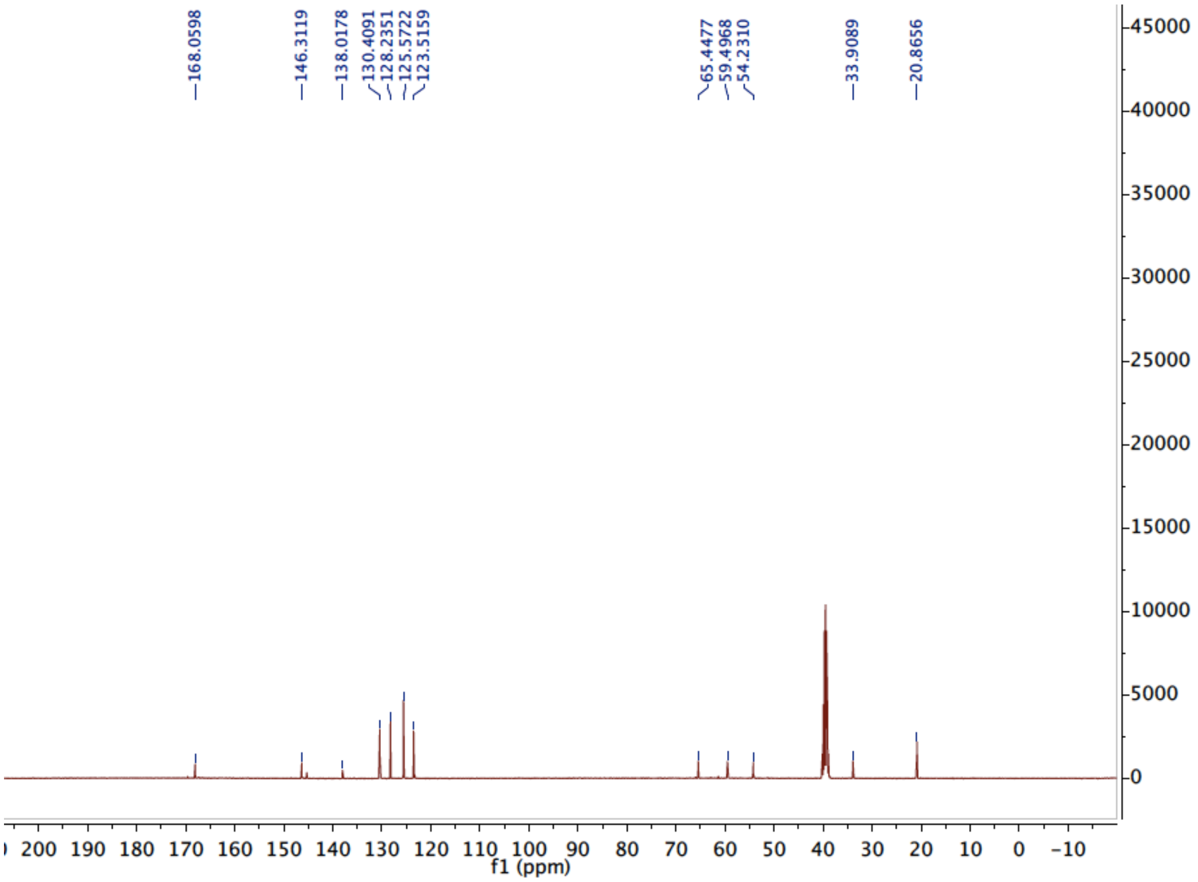

Compound 6

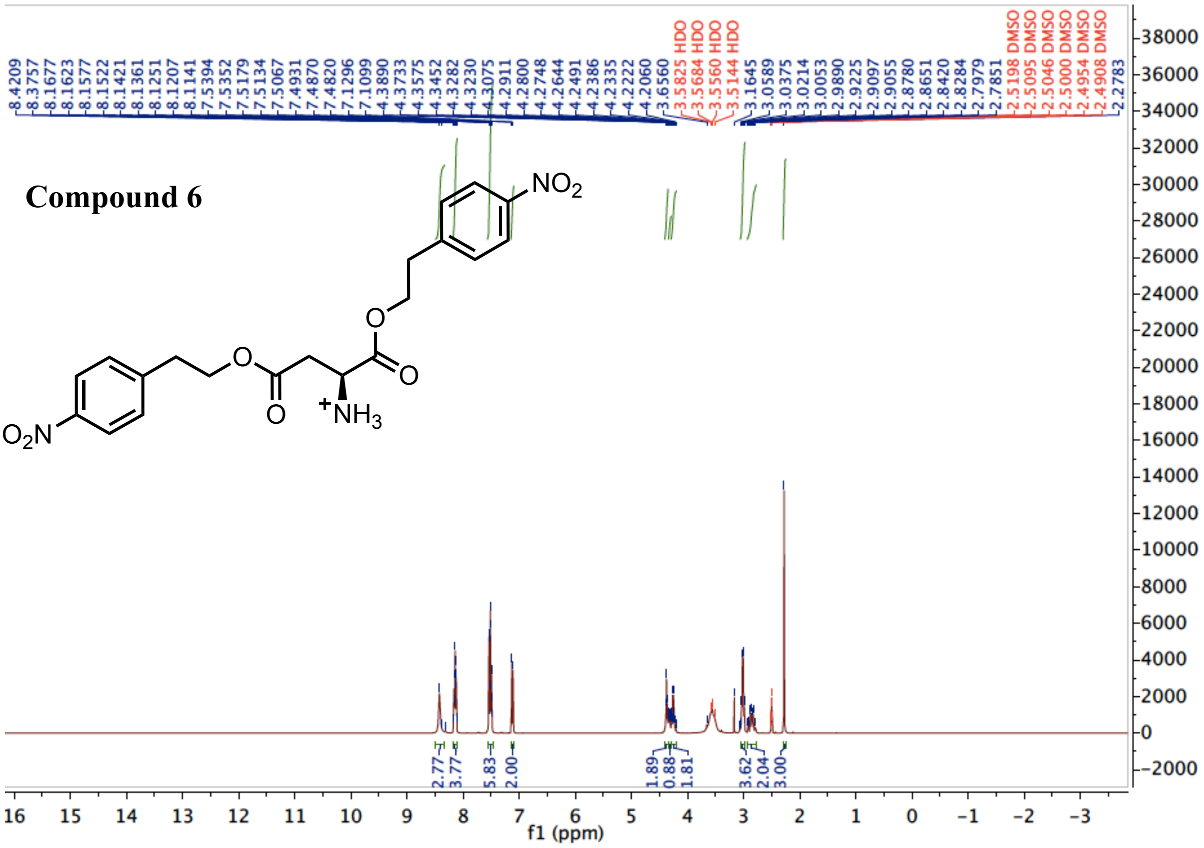
8.4209
8.3757
8.1677
8.1623
8.1577
8.1522
8.1421
8.1361
8.1251
8.1207
8.1141
7.5394
7.5352
7.5179
7.5134
7.5067
7.4931
7.4870
7.4820
7.1296
7.1099
4.3890
4.3733
4.3575
4.3452
4.3282
4.3230
4.3075
4.2911
4.2800
4.2748
4.2644
4.2491
4.2386
4.2335
4.2222
4.2060
3.6560
3.5825 HDO
3.5684 HDO
3.5560 HDO
3.5144 HDO
3.1645
3.0589
3.0375
3.0214
3.0053
2.9890
2.9225
2.9097
2.9055
2.8780
2.8651
2.8420
2.8284
2.7979
2.7851
2.5198 DMSO
2.5095 DMSO
2.5046 DMSO
2.5000 DMSO
2.4954 DMSO
2.4908 DMSO
2.2783
NO_2
O
O
O
O
O
O_2N
$^+NH_3$
2.77
3.77
5.83
2.00
1.89
0.88
1.81
3.62
2.04
3.00
f1 (ppm)

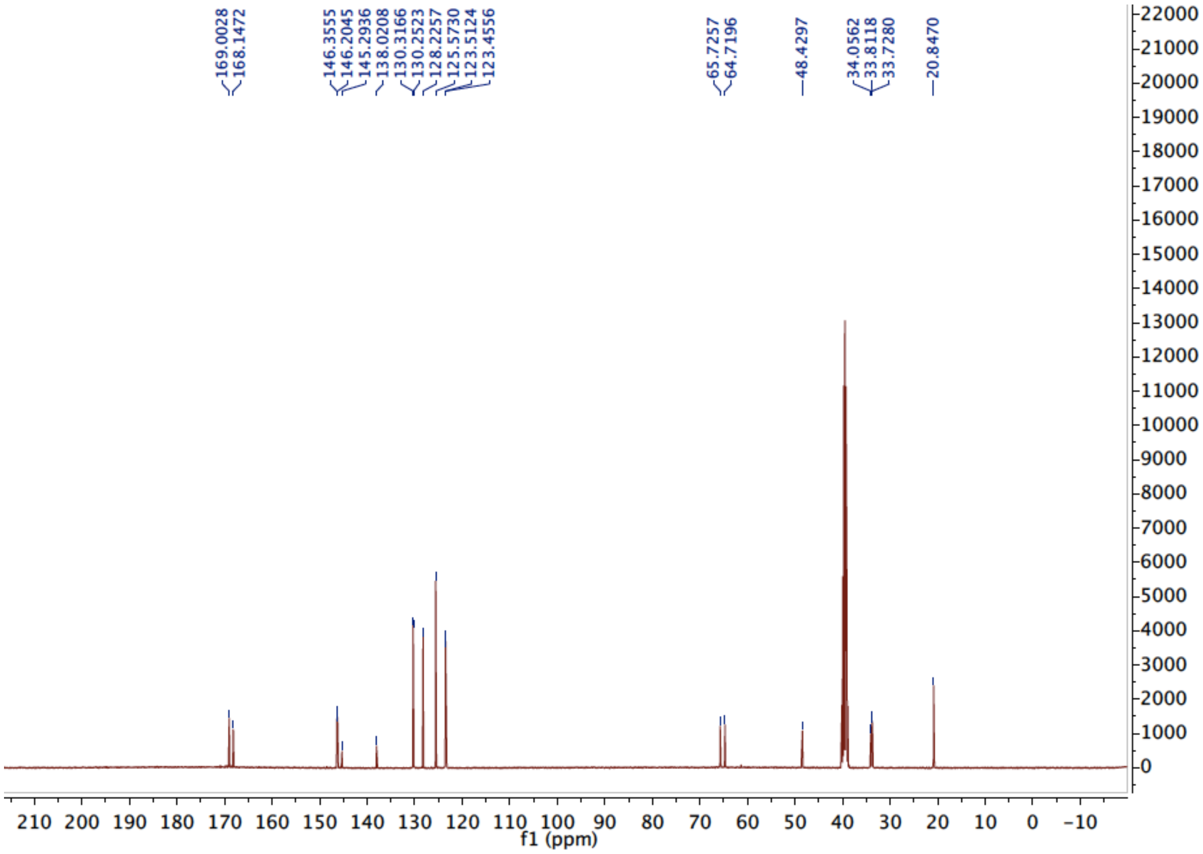
169.0028
168.1472
146.3555
146.2045
145.2936
138.0208
130.3166
130.2523
128.2257
125.5730
123.5124
123.4556
65.7257
64.7196
48.4297
34.0562
33.8118
33.7280
20.8470
f1 (ppm)

Compound 8

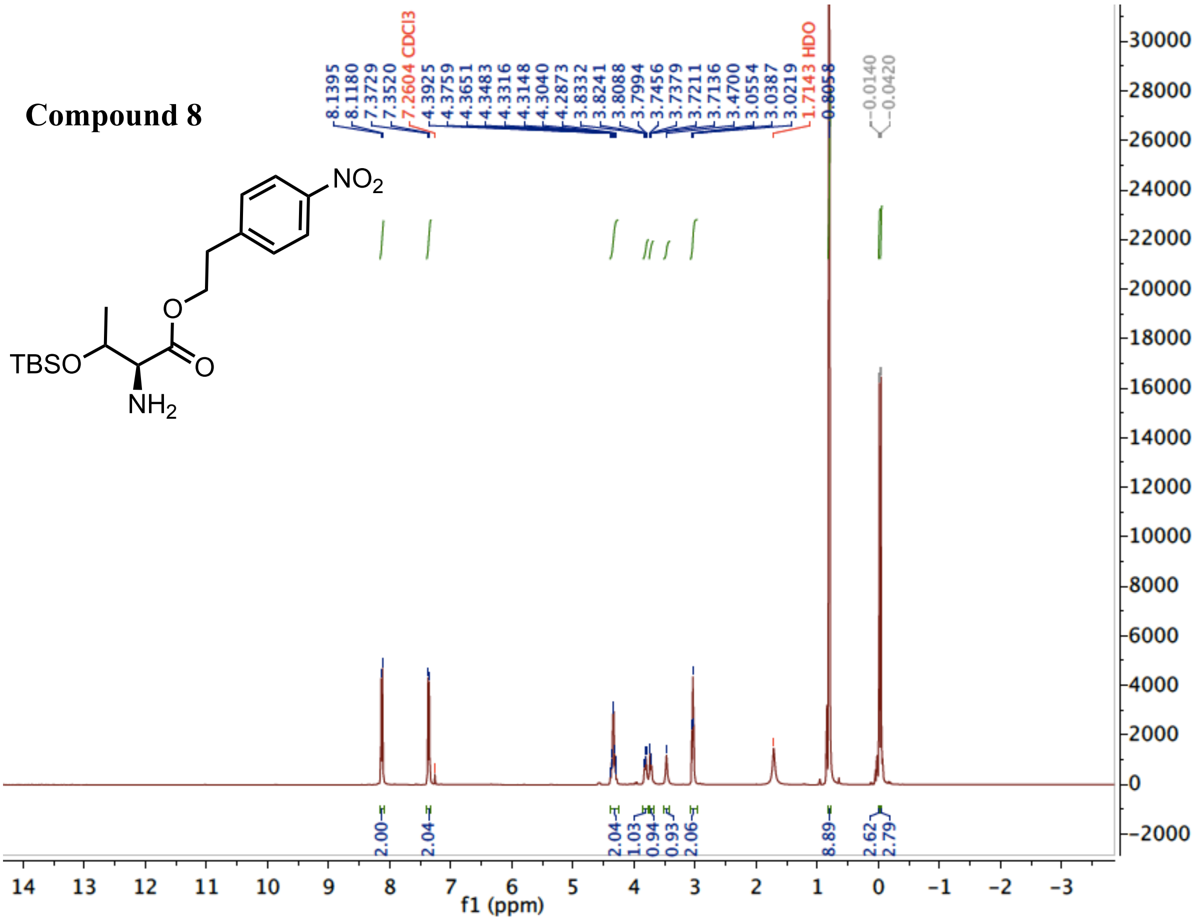

NO2
O
TBSO
O
NH2
8.1395
8.1180
7.3729
7.3520
7.2604 CDCl3
4.3925
4.3759
4.3651
4.3483
4.3316
4.3148
4.3040
4.2873
3.8332
3.8241
3.8088
3.7994
3.7456
3.7379
3.7211
3.7136
3.4700
3.0554
3.0387
3.0219
1.7143 HDO
0.8058
0.0140
-0.0420
2.00
2.04
2.04
1.03
0.94
0.93
2.06
8.89
2.62
2.79
f1 (ppm)

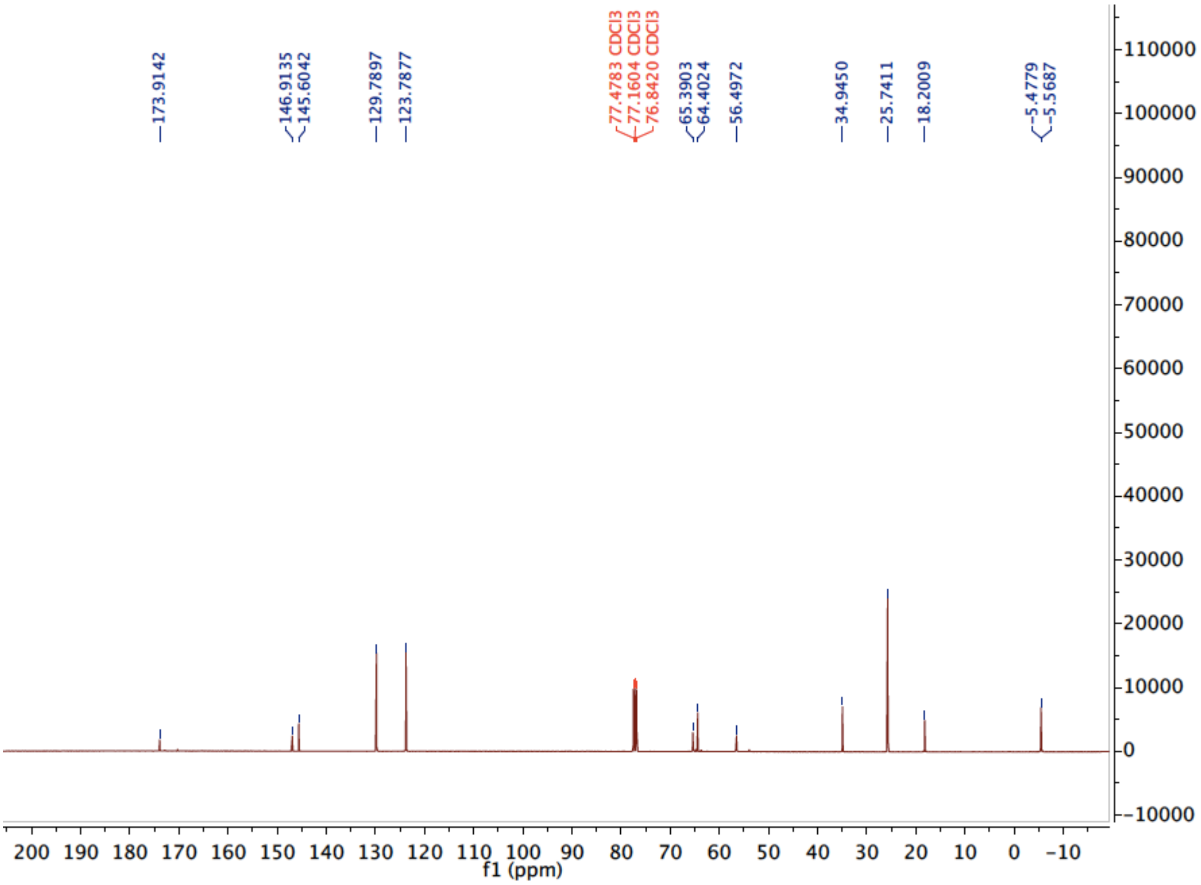

173.9142
146.9135
145.6042
129.7897
123.7877
77.4783 CDCl3
77.1604 CDCl3
76.8420 CDCl3
65.3903
64.4024
56.4972
34.9450
25.7411
18.2009
-5.4779
-5.5687
f1 (ppm)

Compound 12

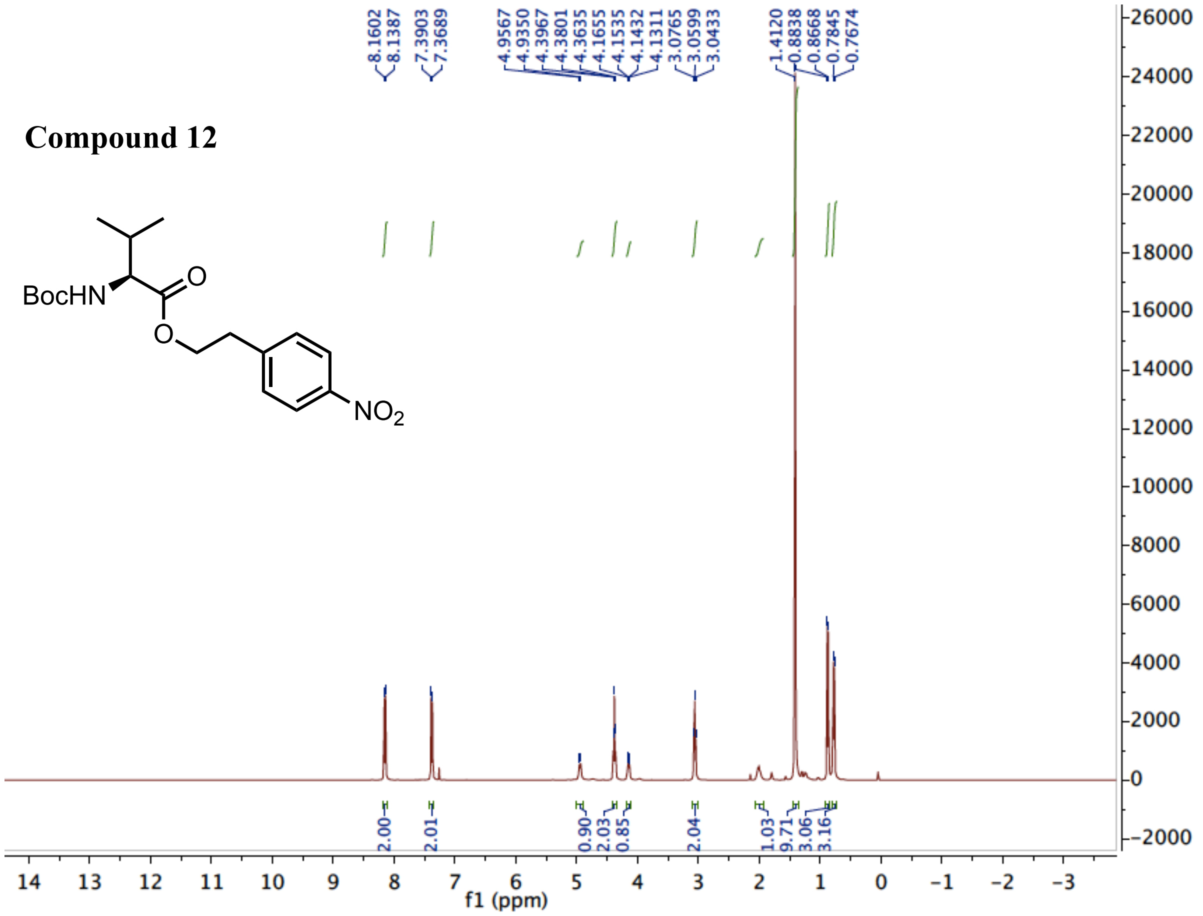

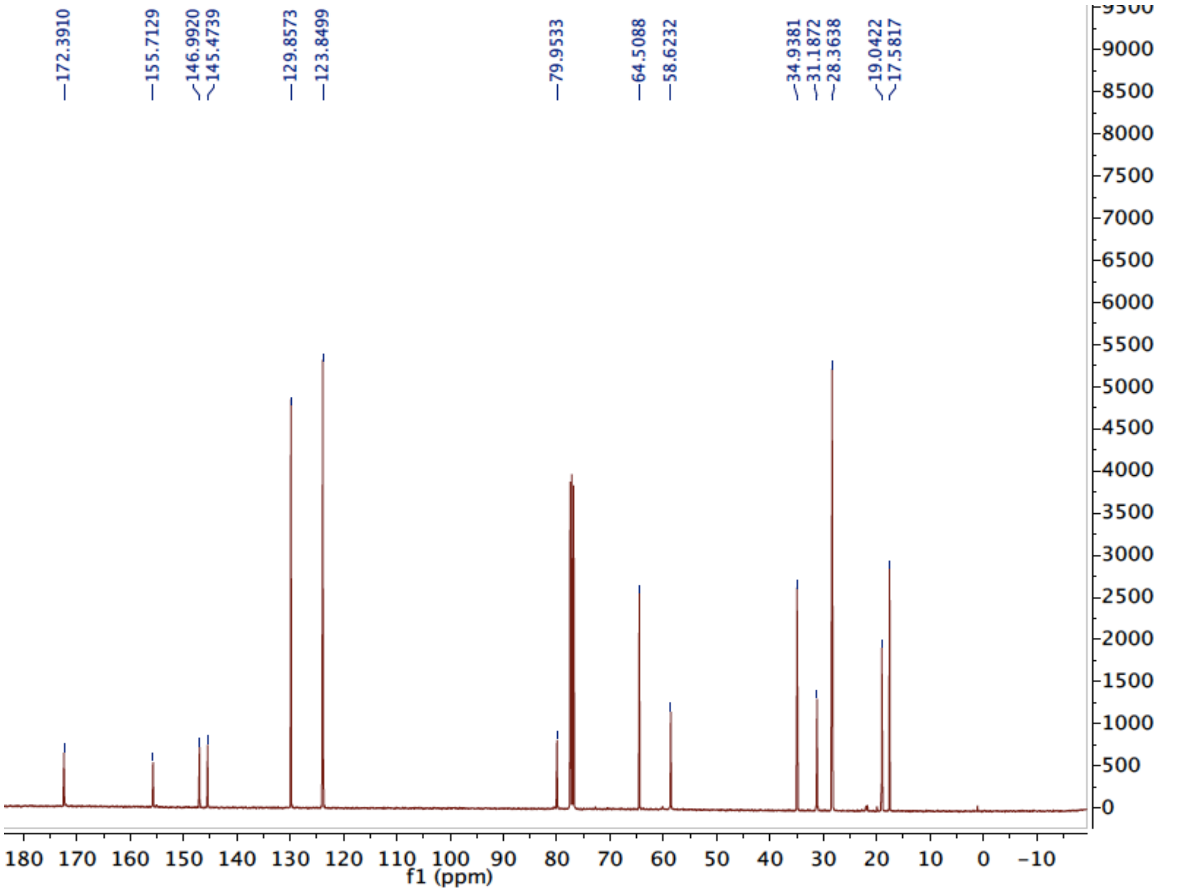

Compound 13

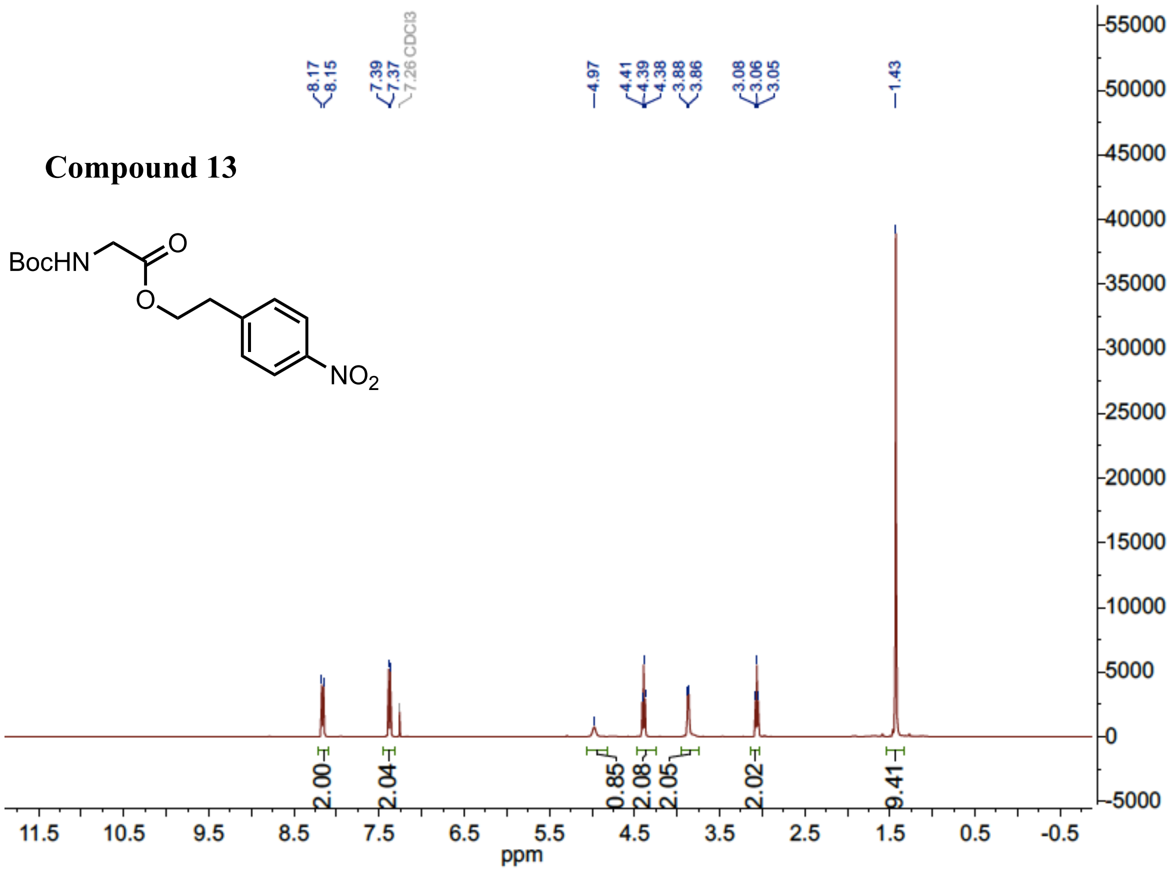

BocHN
O
O
NO2
8.17
8.15
7.39
7.37
7.26 CDCl3
4.97
4.41
4.39
4.38
3.88
3.86
3.08
3.06
3.05
1.43
2.00
2.04
0.85
2.08
2.05
2.02
9.41
ppm

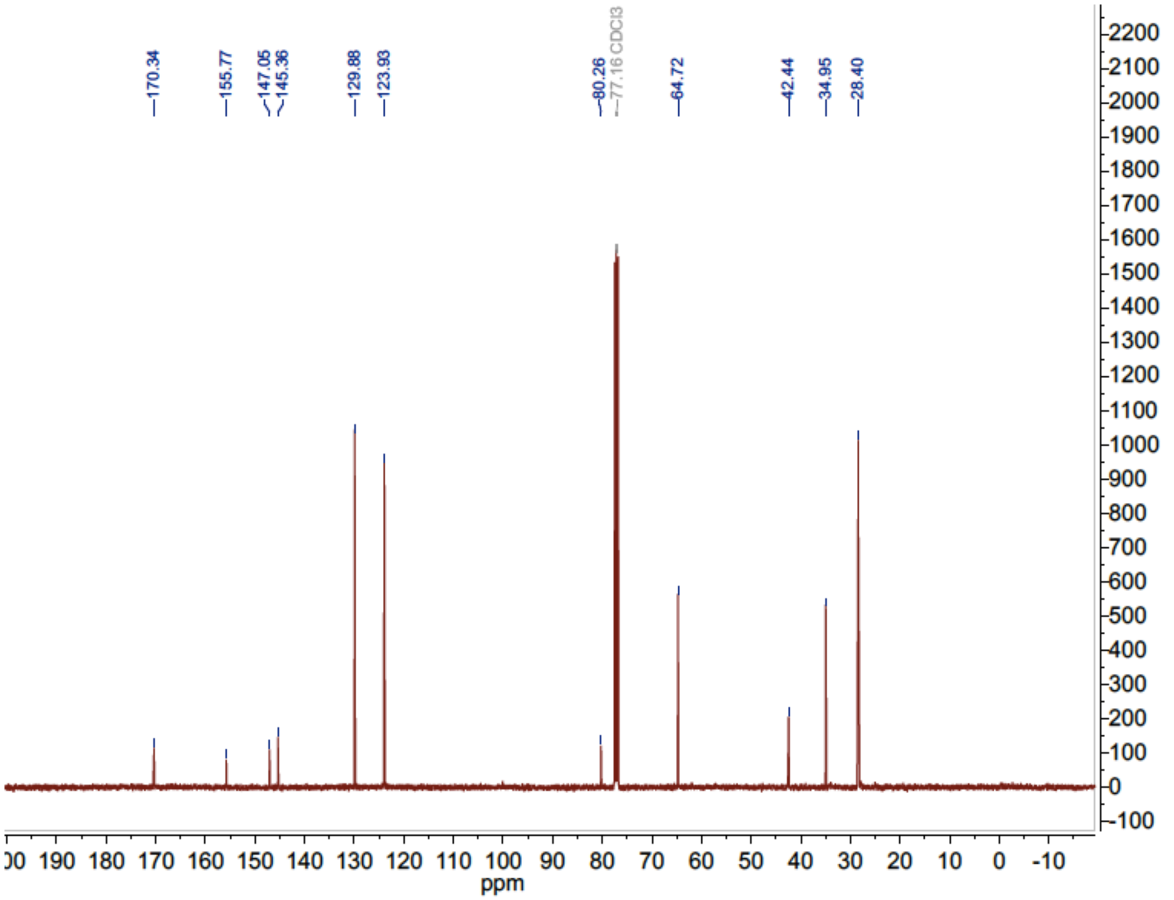

170.34
155.77
147.05
145.36
129.88
123.93
80.26
77.16 CDCl3
64.72
42.44
34.95
28.40
ppm

Compound 14a

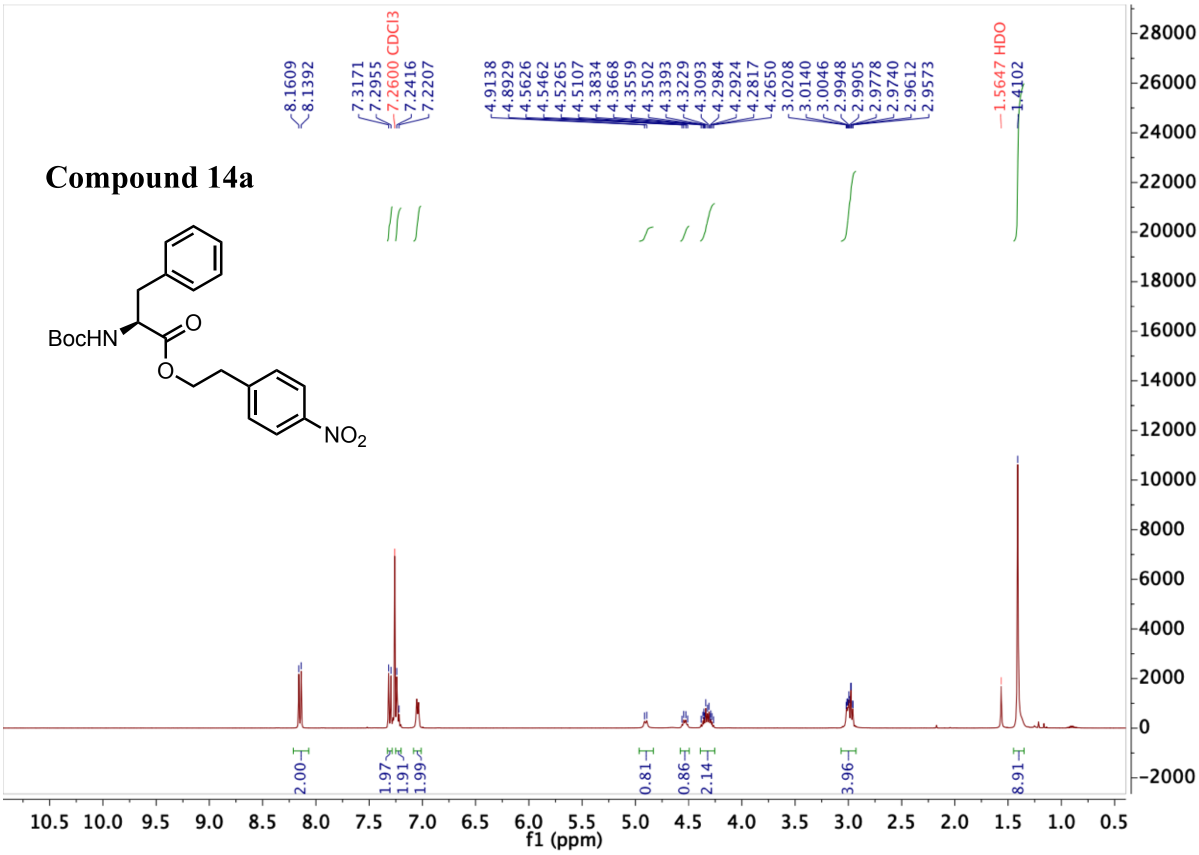

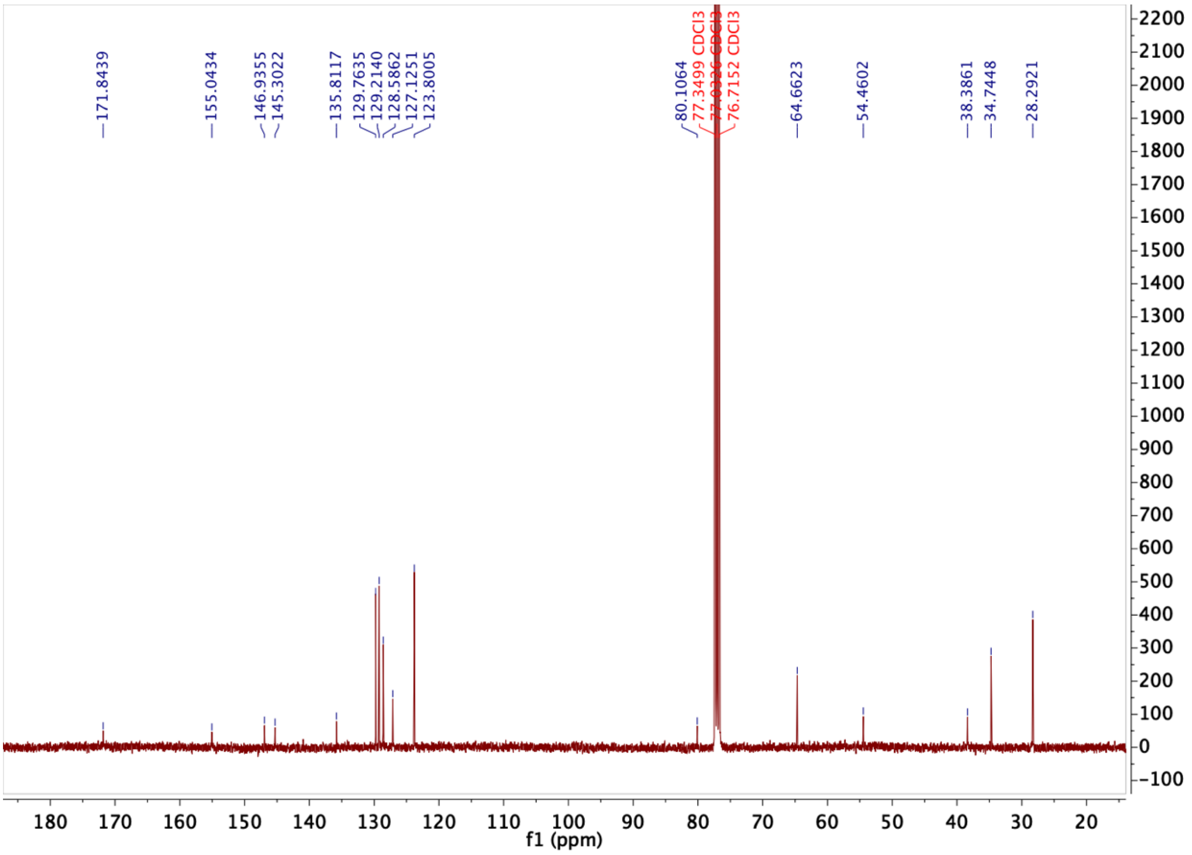

Compound 14b

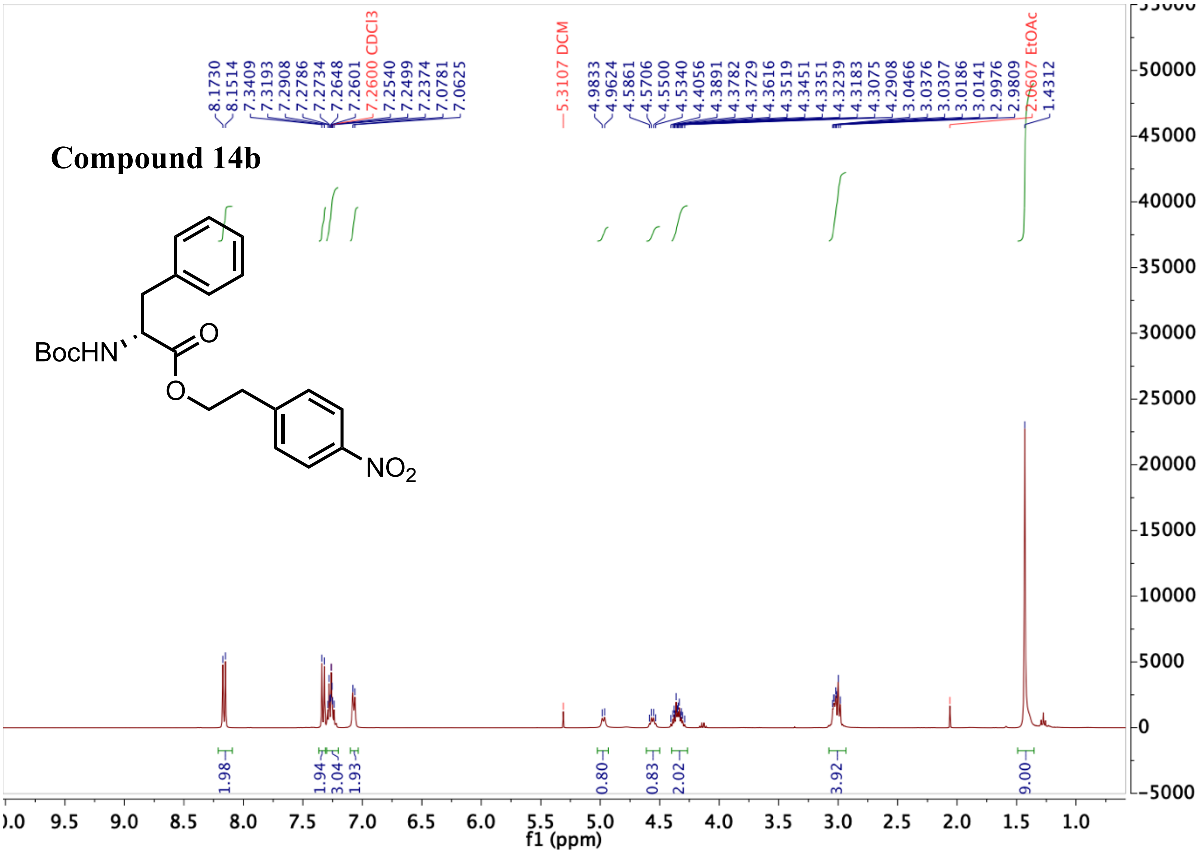

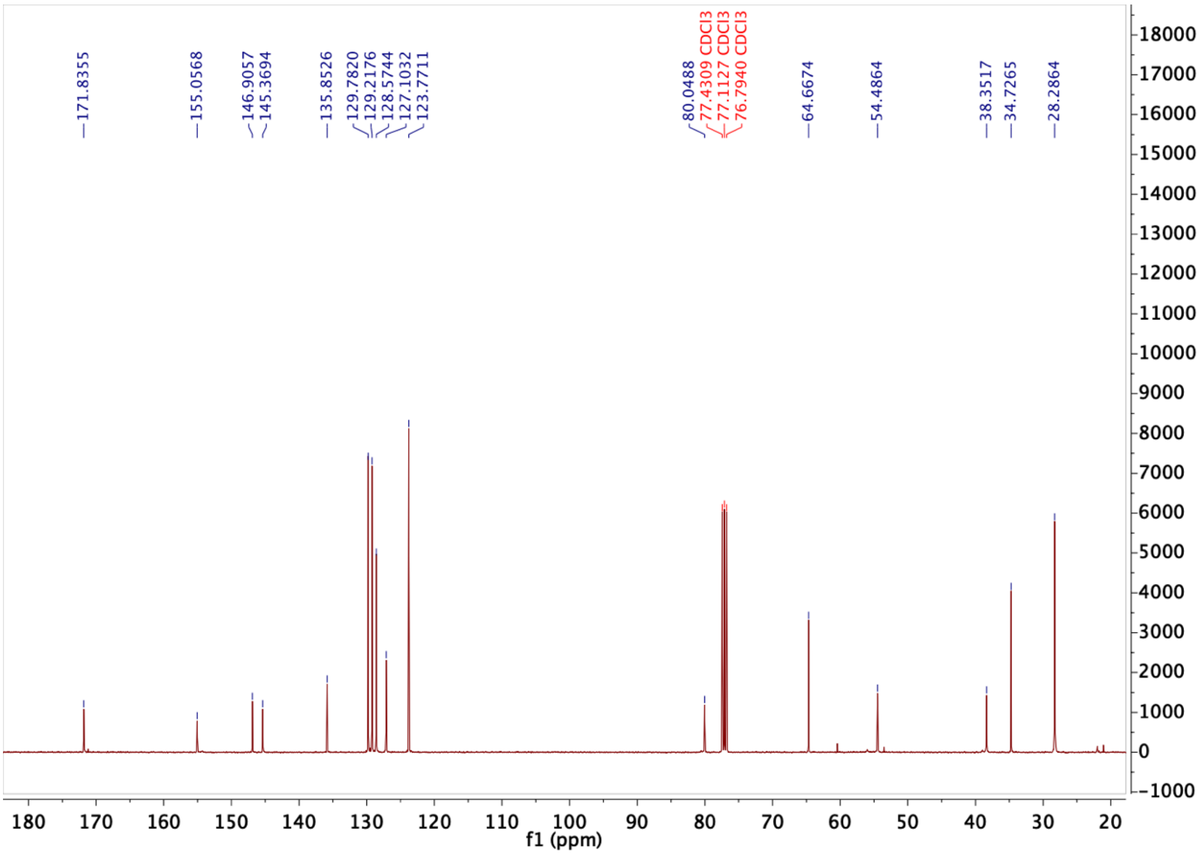

Compound 15

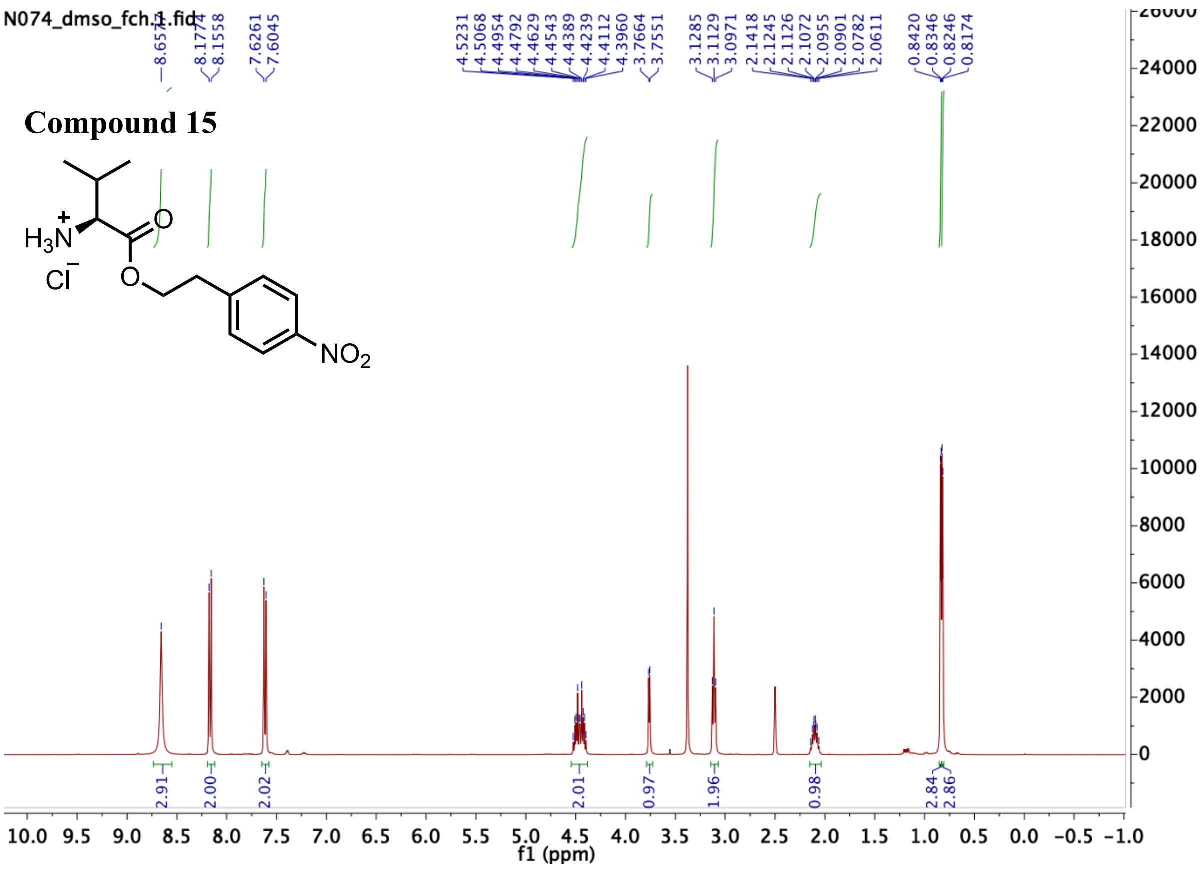

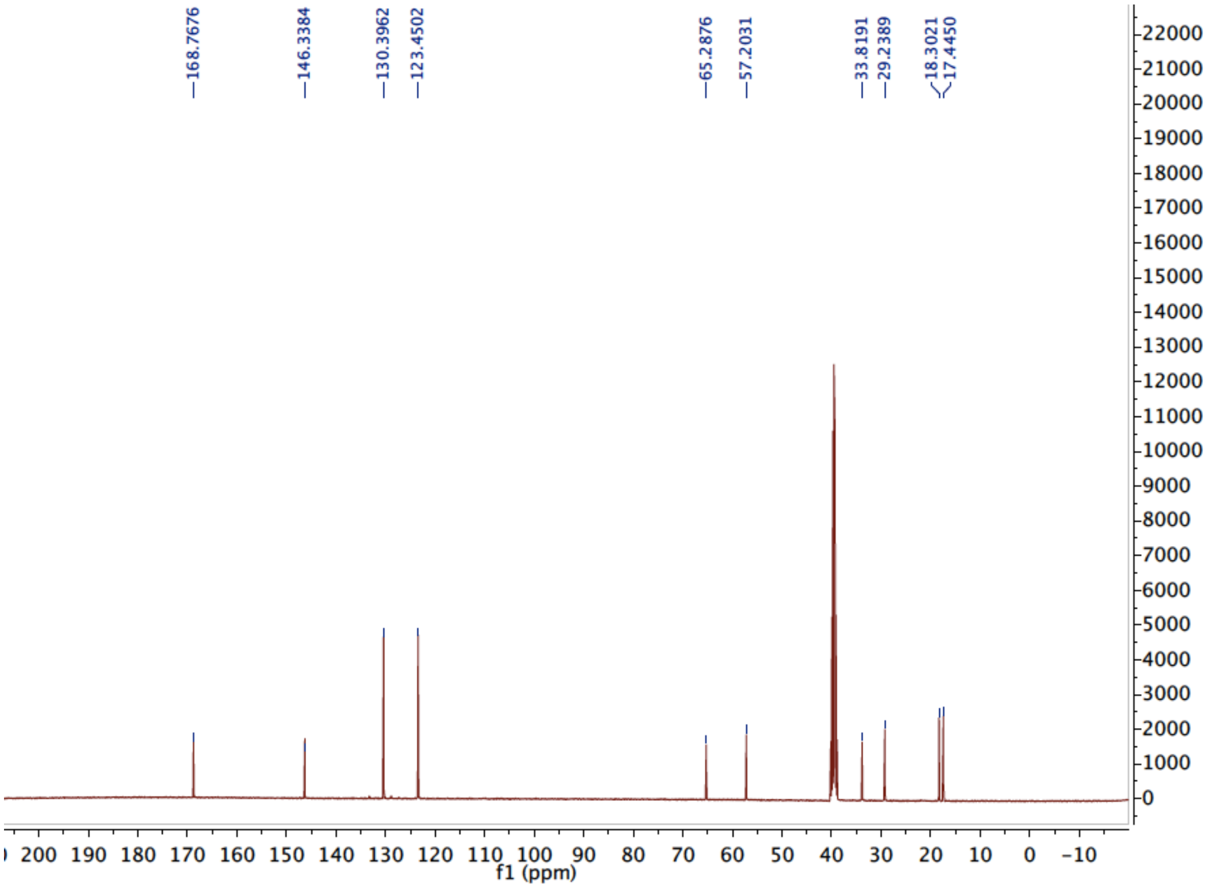

Compound 16

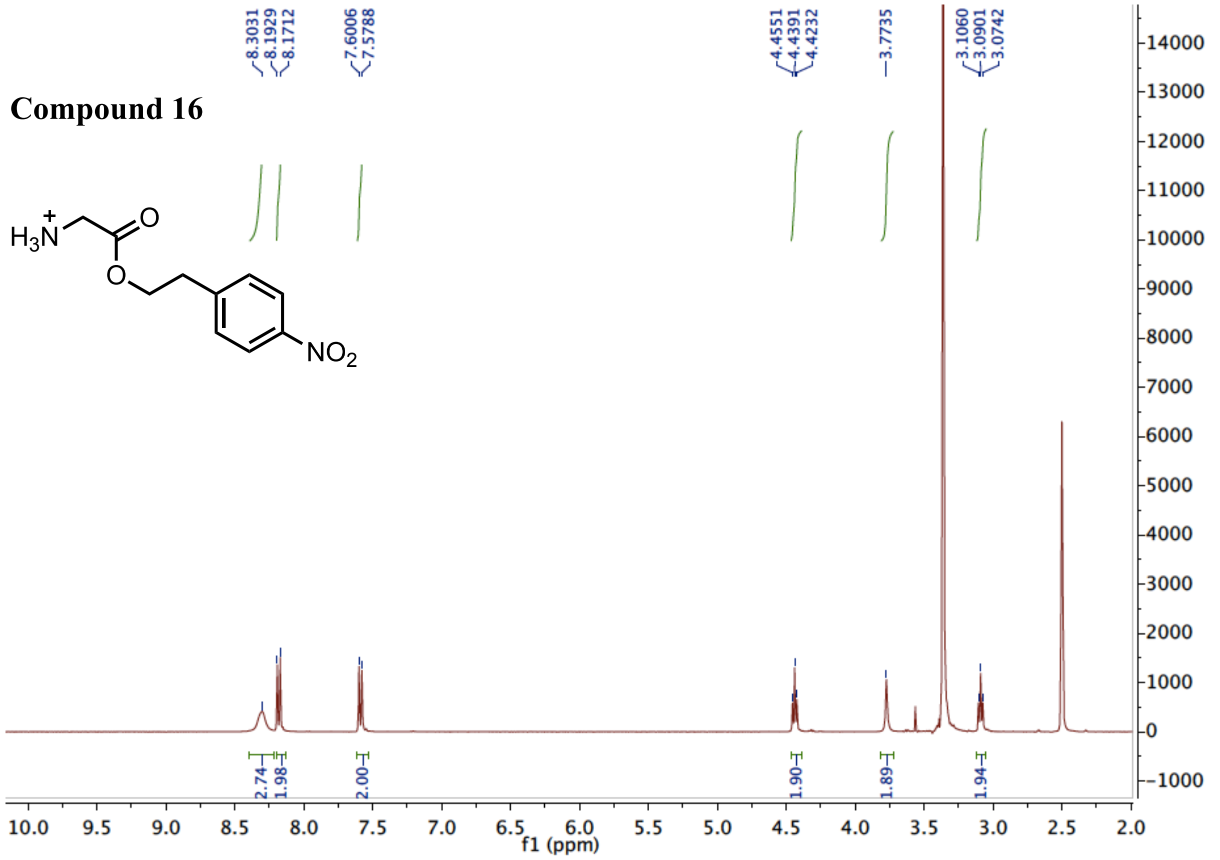
H3N+
O
O
NO2
8.3031
8.1929
8.1712
7.6006
7.5788
4.4551
4.4391
4.4232
3.7735
3.1060
3.0901
3.0742
2.74
1.98
2.00
1.90
1.89
1.94
f1 (ppm)

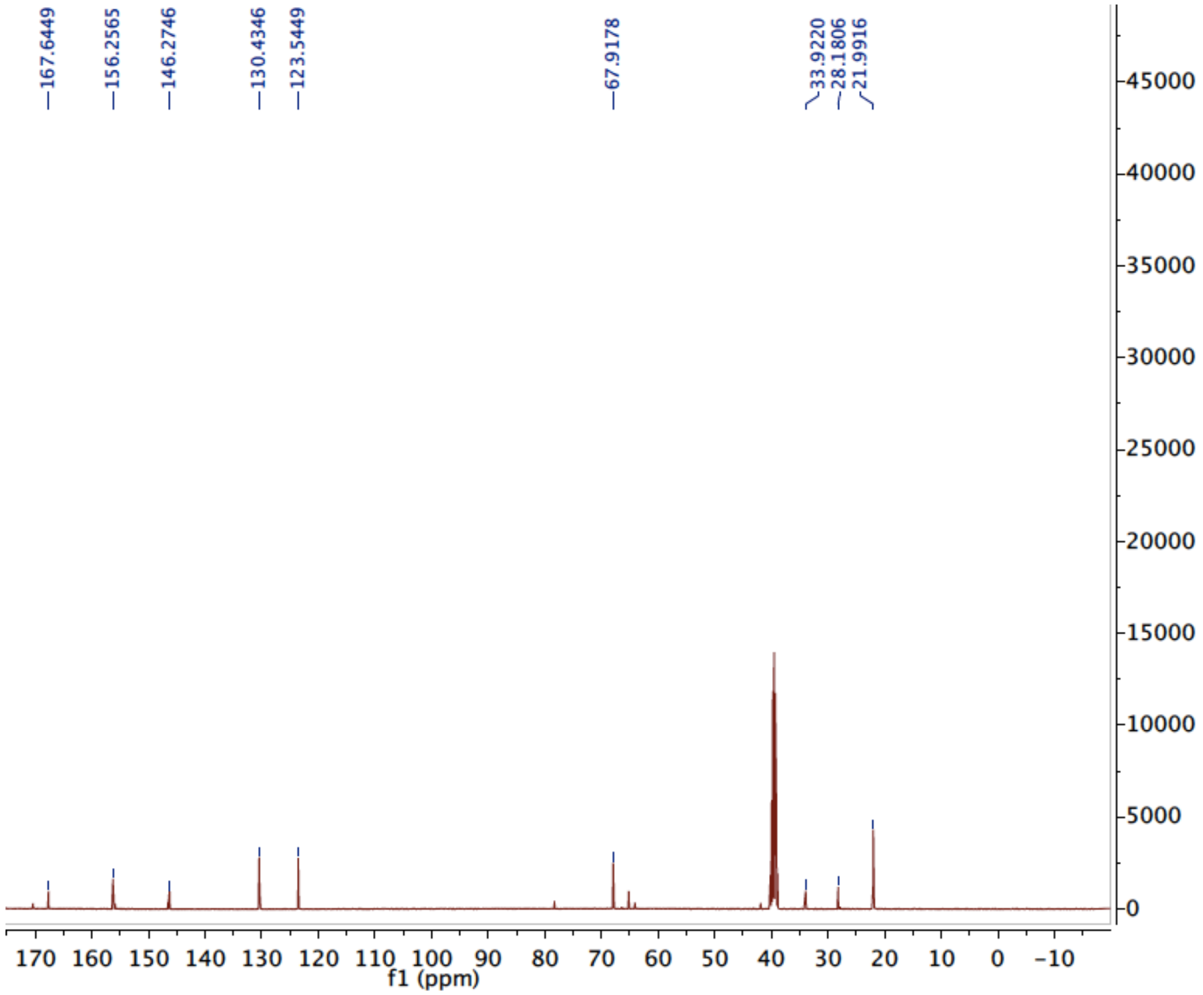
167.6449
156.2565
146.2746
130.4346
123.5449
67.9178
33.9220
28.1806
21.9916
f1 (ppm)

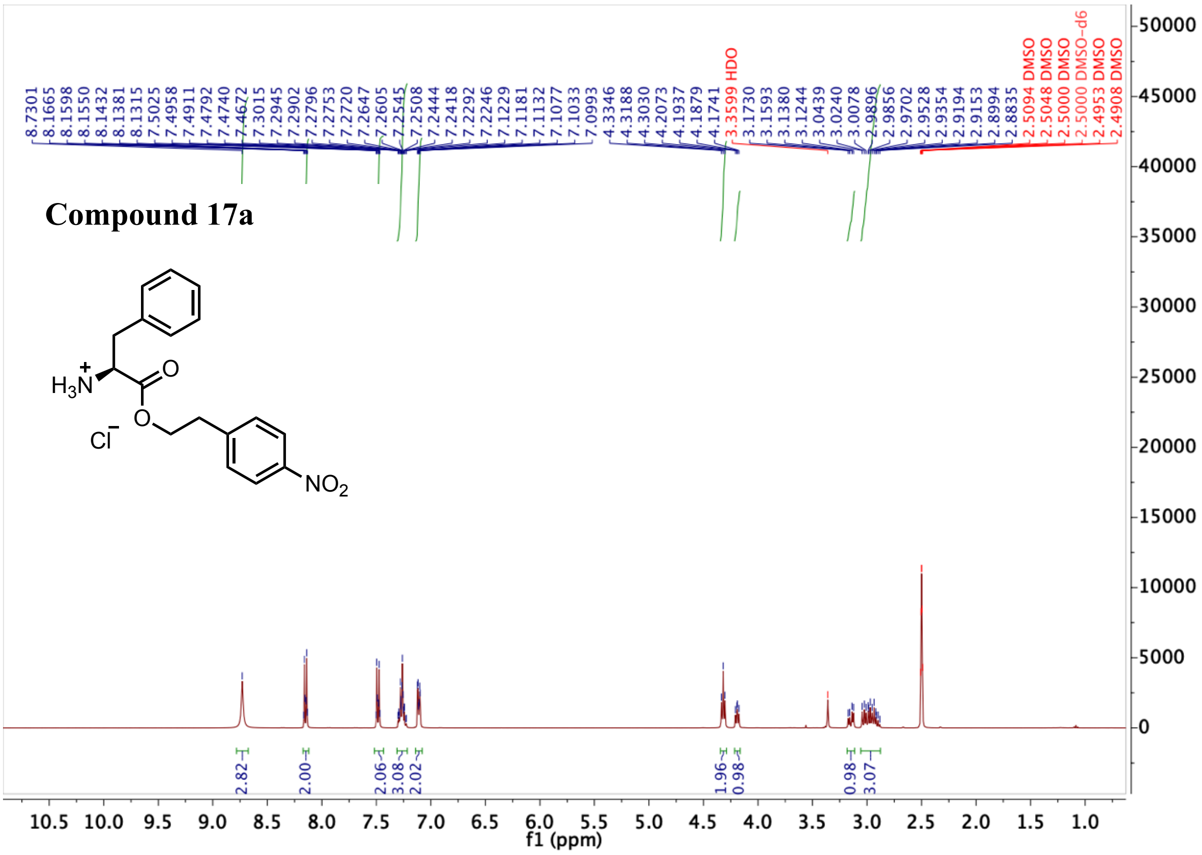
Compound 17a
NO2
Cl
f1 (ppm)

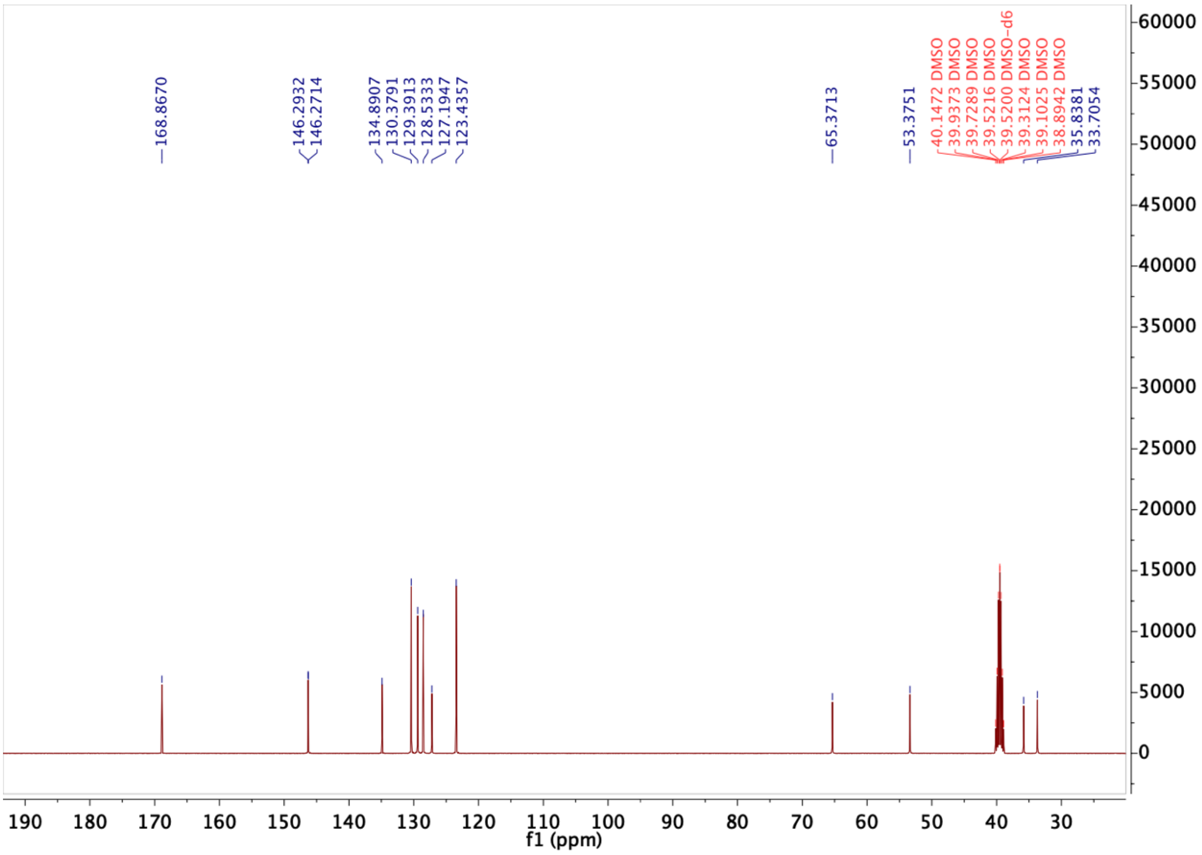
f1 (ppm)

Compound 17b

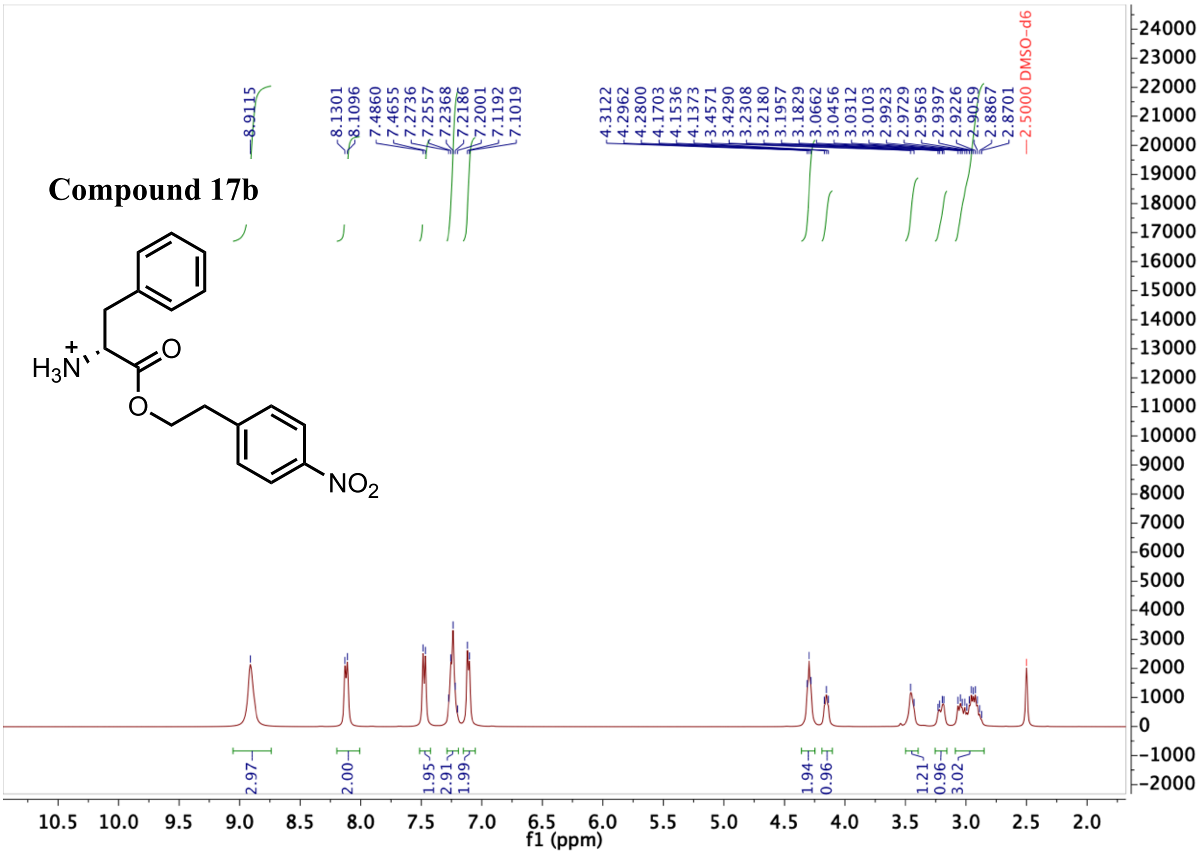

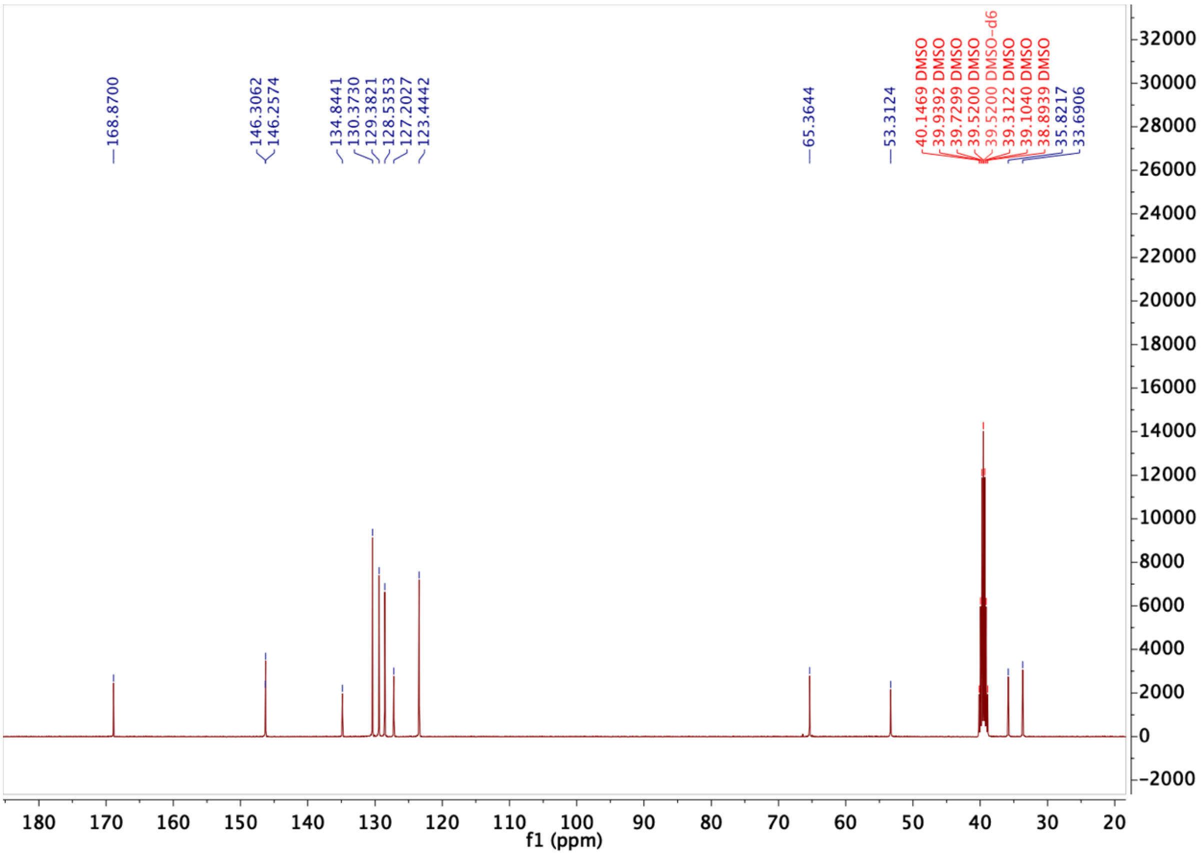

Compound 19

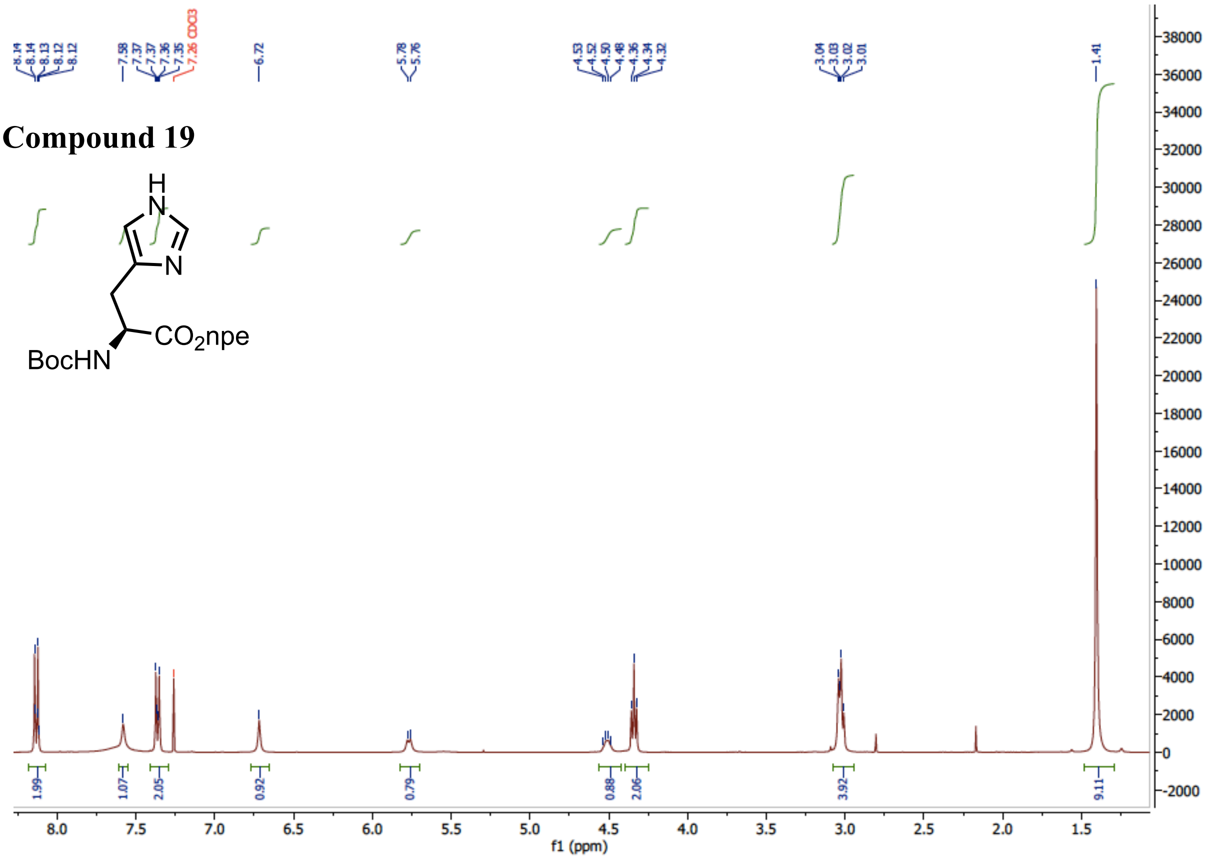

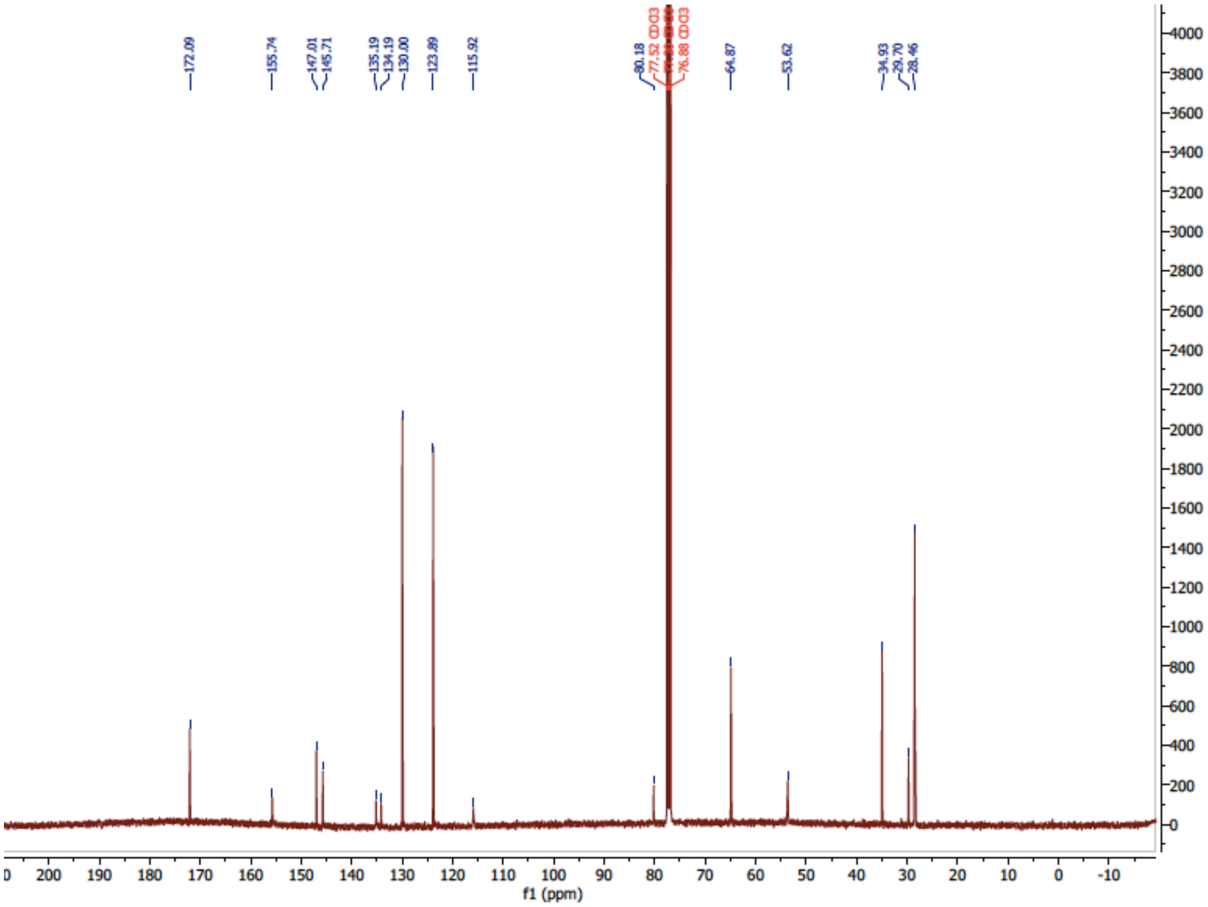

Compound 20

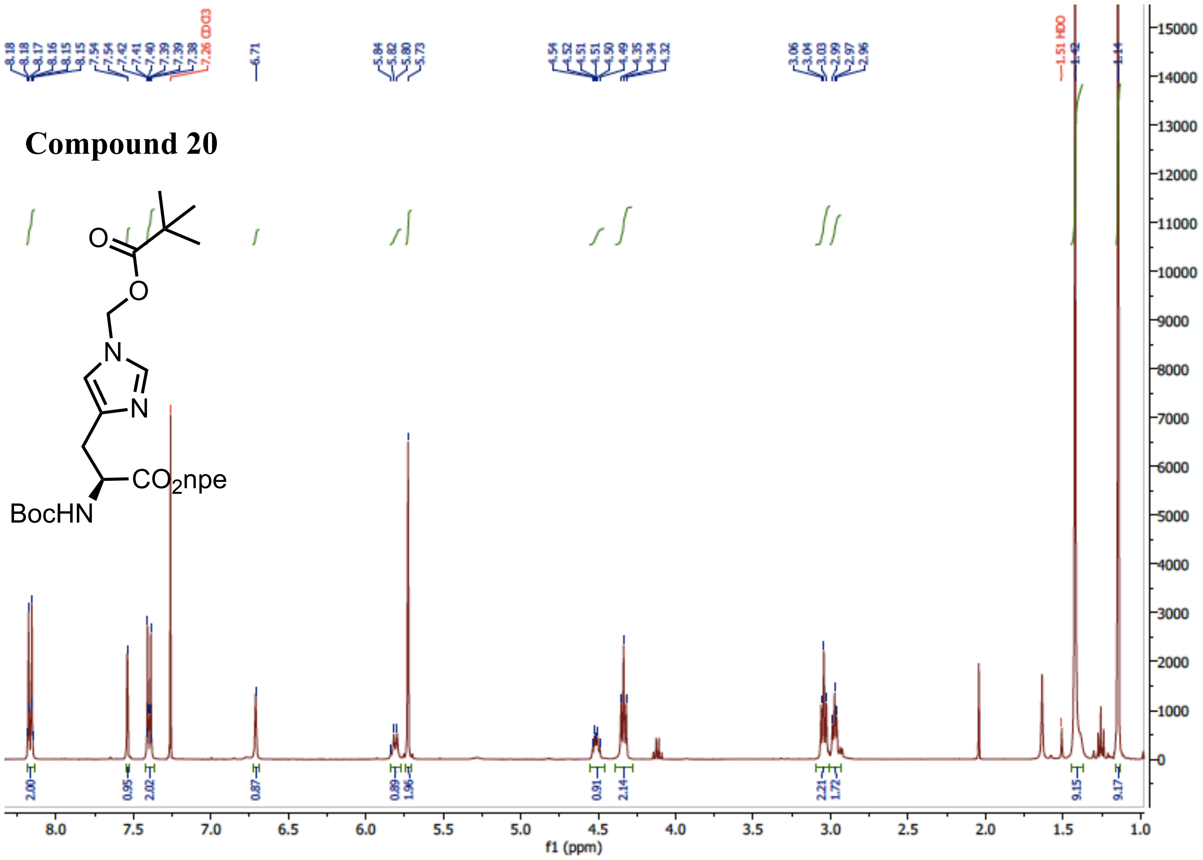

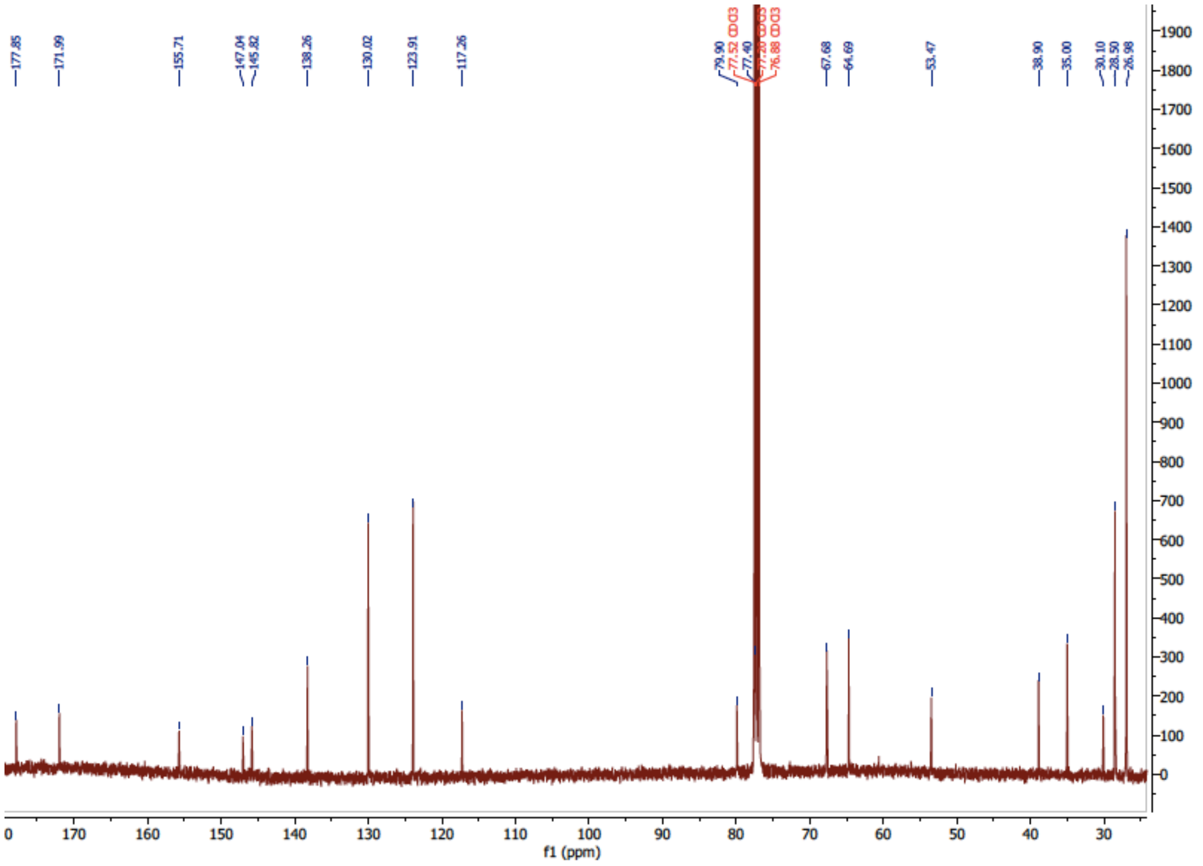

Compound 21

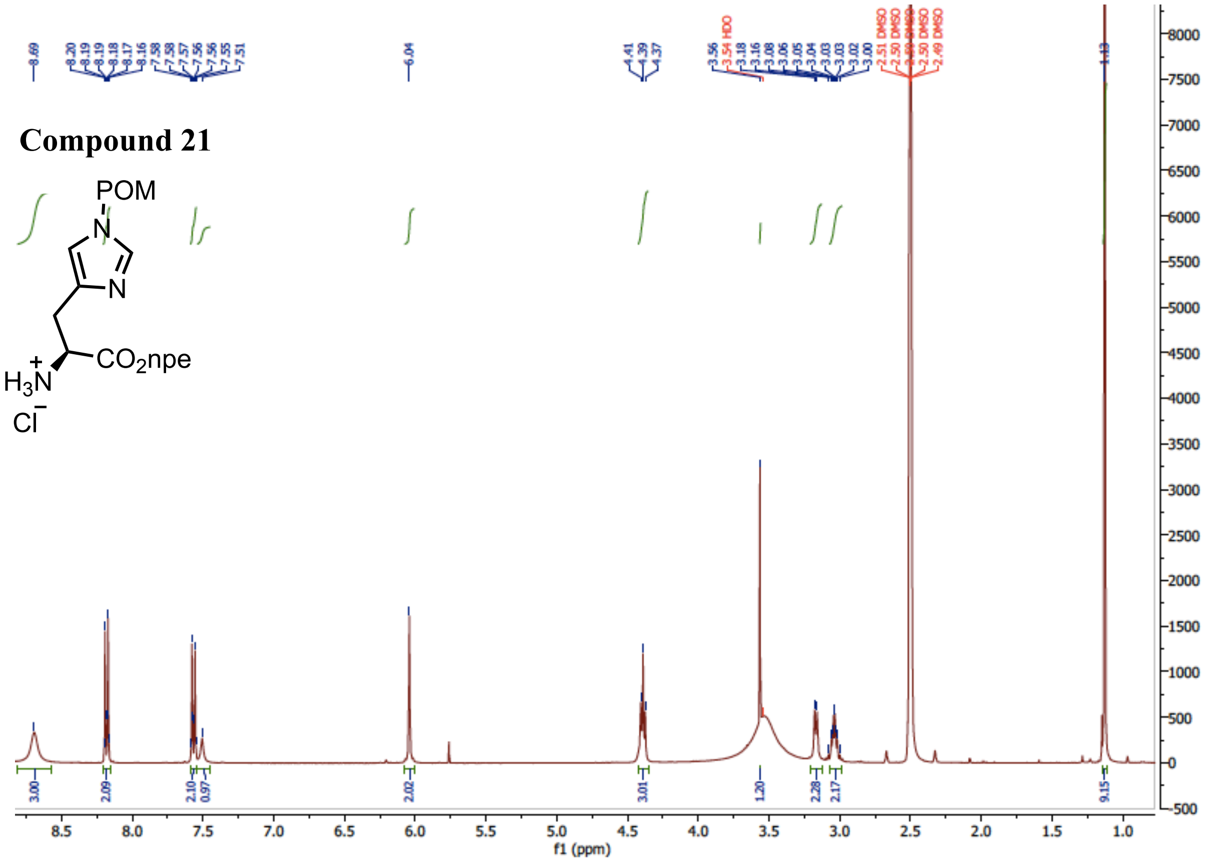

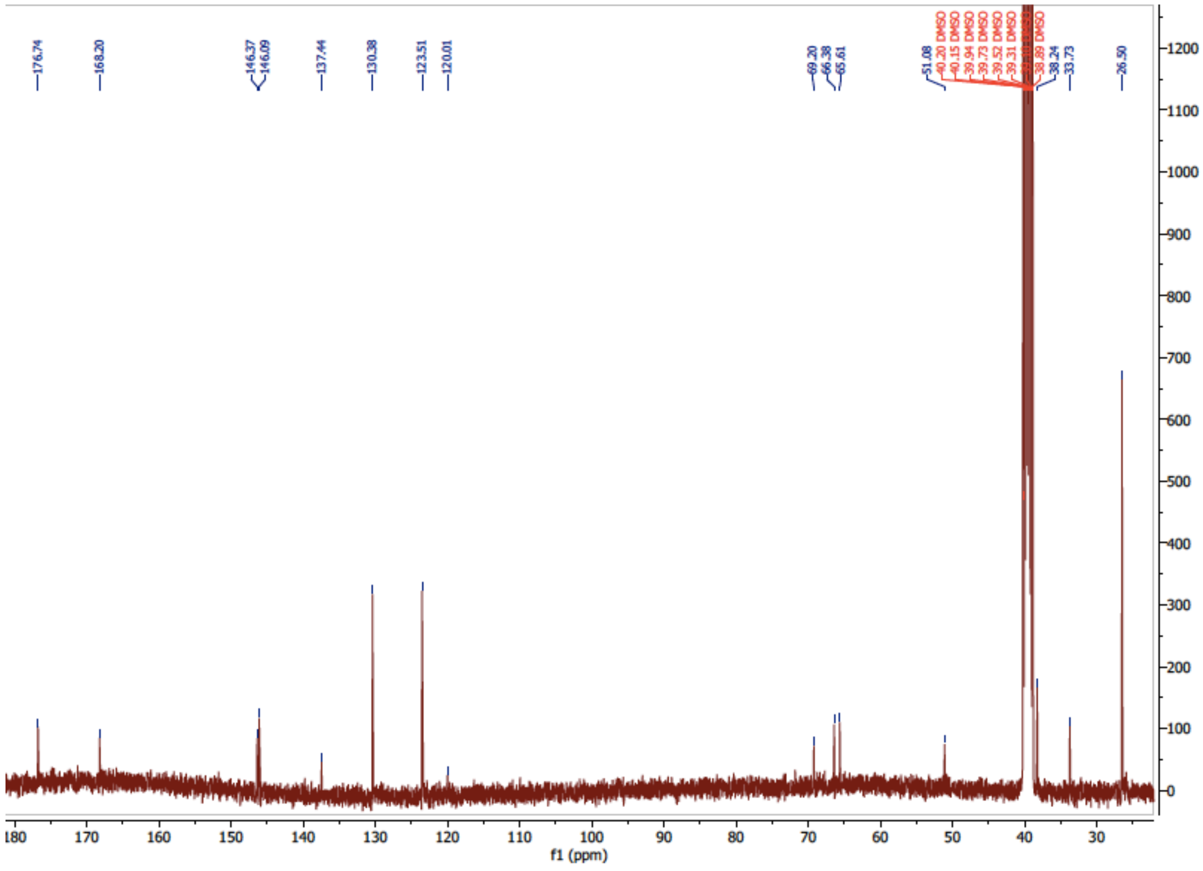

Compound 25

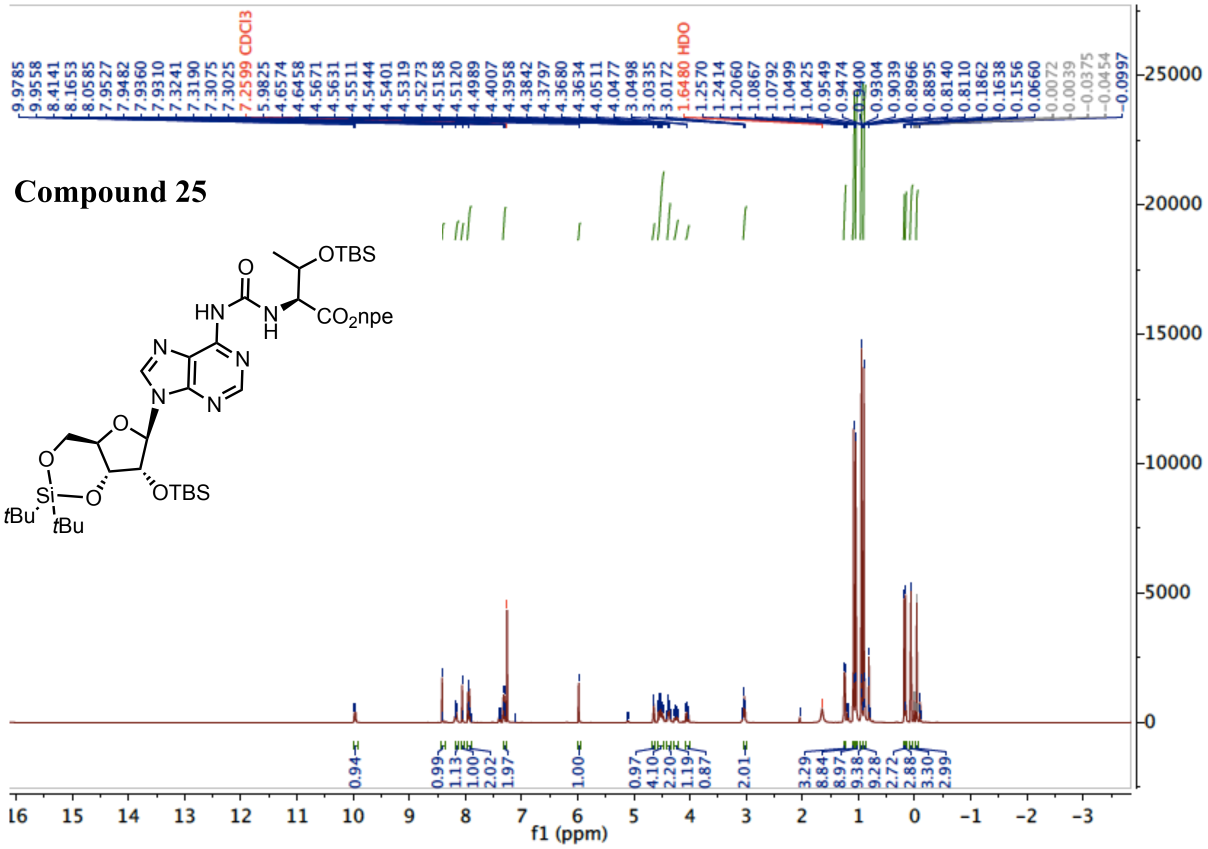

9.9785
9.9558
8.4141
8.1653
8.0585
7.9527
7.9482
7.9360
7.9310
7.3241
7.3190
7.3075
7.3025
7.2599 CDCl3
5.9825
4.6574
4.6458
4.5671
4.5631
4.5511
4.5444
4.5401
4.5319
4.5273
4.5158
4.5120
4.4989
4.4007
4.3958
4.3842
4.3797
4.3680
4.3634
4.0511
4.0477
3.0498
3.0335
3.0172
1.6480 HDO
1.2570
1.2414
1.2060
1.0867
1.0792
1.0499
1.0425
0.9549
0.9474
0.9400
0.9304
0.9039
0.8966
0.8895
0.8140
0.8110
0.1862
0.1638
0.1556
0.0660
0.0072
0.0039
-0.0375
-0.0454
-0.0997
OTBS
O
HN
N
H
CO2npe
N
N
N
N
O
O
Si
O
OTBS
tBu
tBu
0.94
0.99
1.13
1.00
2.02
1.97
1.00
0.97
4.10
2.20
1.19
0.87
2.01
3.29
8.84
8.97
9.38
9.28
2.72
2.88
3.30
2.99
25000
20000
15000
10000
5000
0
16 15 14 13 12 11 10 9 8 7 6 5 4 3 2 1 0 -1 -2 -3
f1 (ppm)

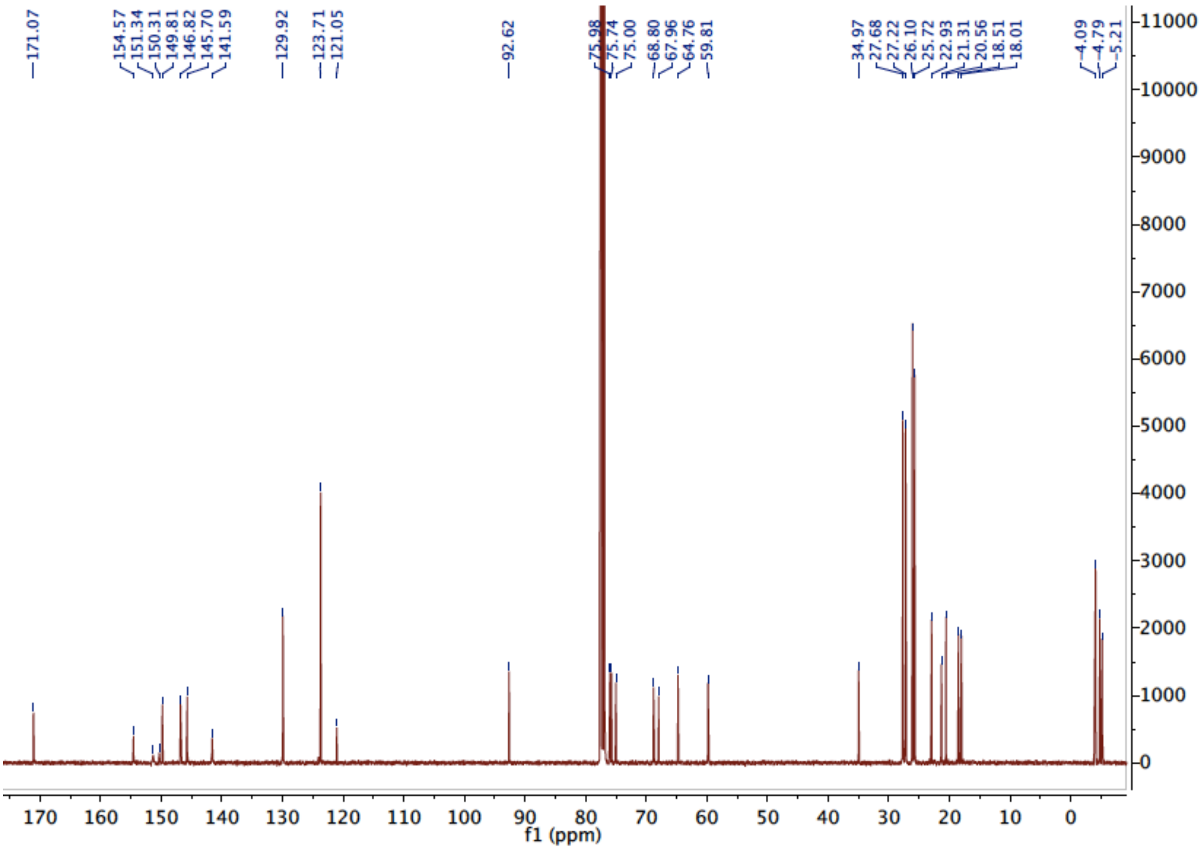

171.07
154.57
151.34
150.31
149.81
146.82
145.70
141.59
129.92
123.71
121.05
92.62
75.98
75.74
75.00
68.80
67.96
64.76
59.81
34.97
27.68
27.22
26.10
25.72
22.93
21.31
20.56
18.51
18.01
-4.09
-4.79
-5.21
11000
10000
9000
8000
7000
6000
5000
4000
3000
2000
1000
0
170 160 150 140 130 120 110 100 90 80 70 60 50 40 30 20 10 0
f1 (ppm)

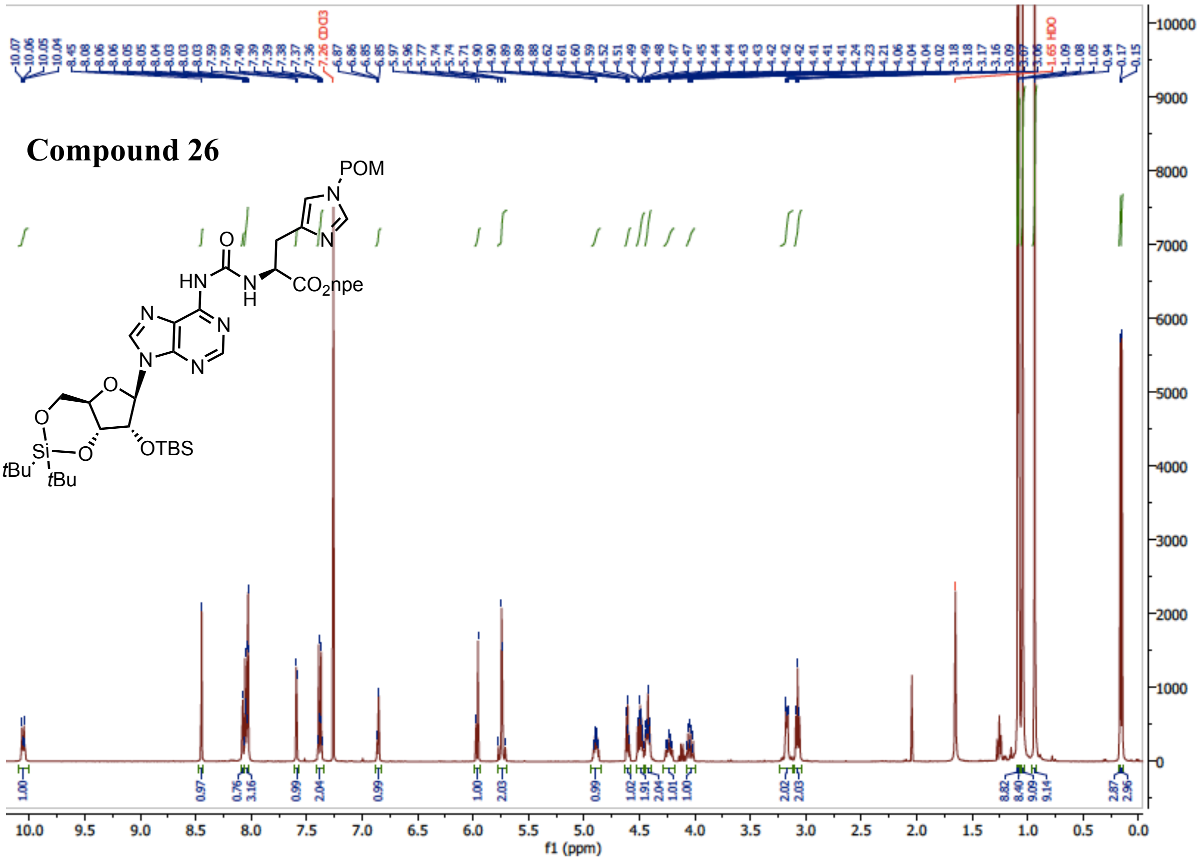
Compound 26
POM
N
N
O
HN
N
H
CO2npe
N
N
N
N
O
O
Si
O
tBu
tBu
OTBS
f1 (ppm)

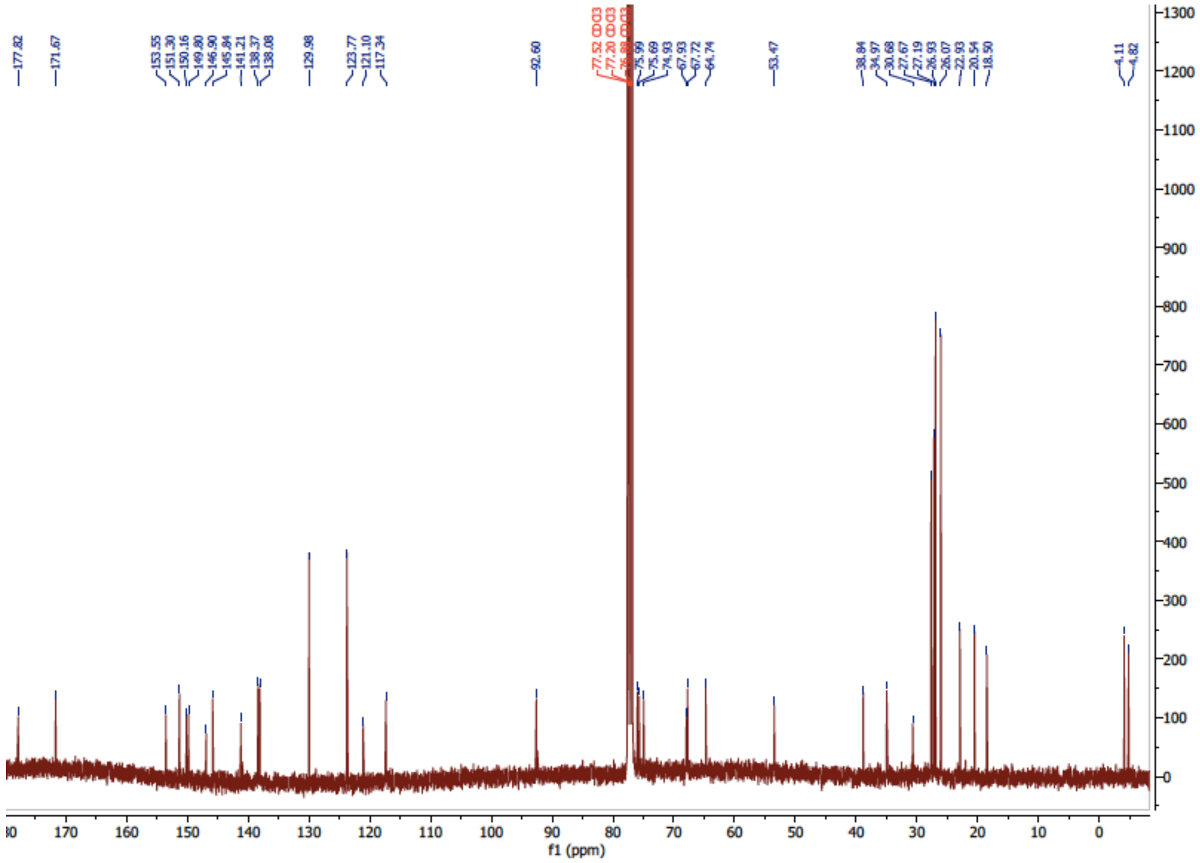
f1 (ppm)

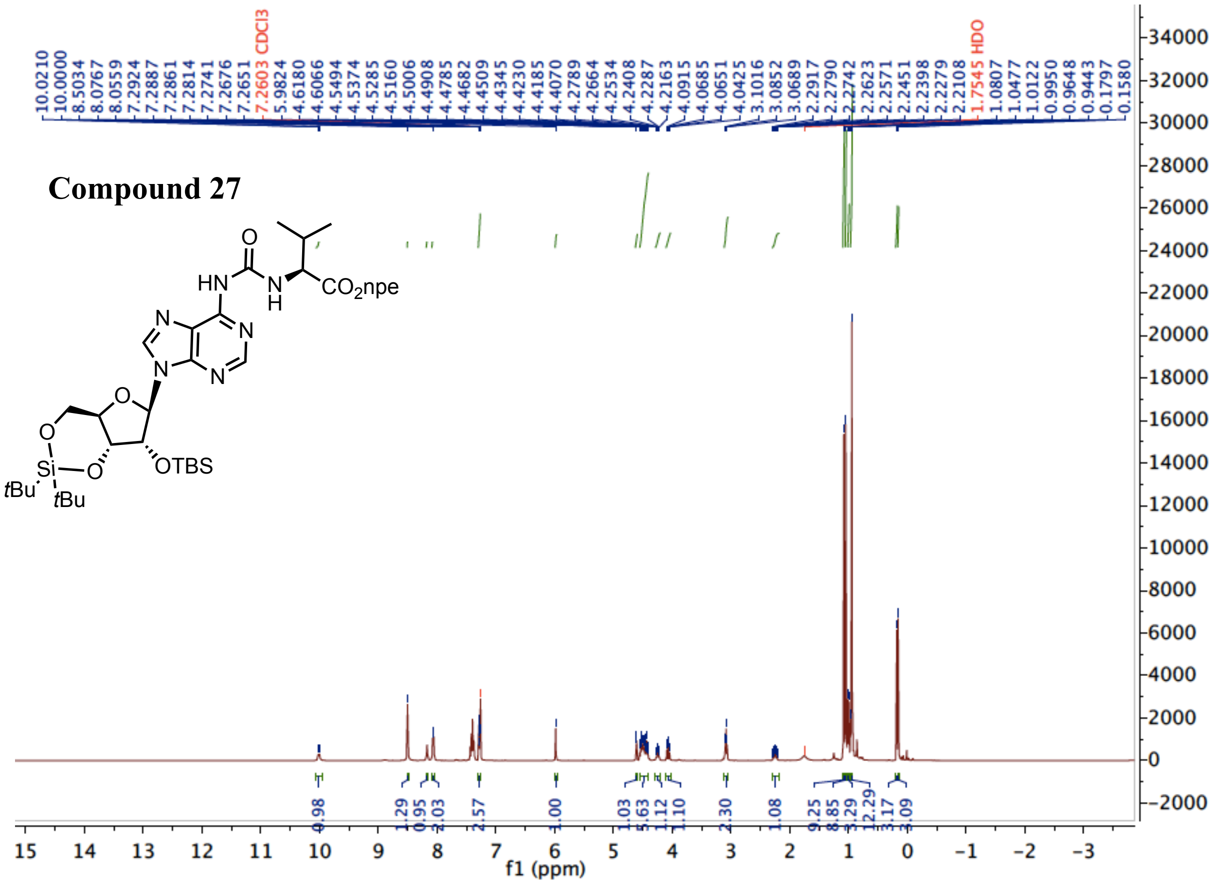
Compound 27
10.0210 10.0000 8.5034 8.0767 8.0559 7.2924 7.2887 7.2861 7.2814 7.2741 7.2676 7.2651 7.2603 CDCl3 5.9824 4.6180 4.6066 4.5494 4.5374 4.5285 4.5160 4.5006 4.4908 4.4785 4.4682 4.4509 4.4345 4.4230 4.4185 4.4070 4.2789 4.2664 4.2534 4.2408 4.2287 4.2163 4.0915 4.0685 4.0651 4.0425 3.1016 3.0852 3.0689 2.2917 2.2790 2.2742 2.2623 2.2571 2.2451 2.2398 2.2279 2.2108 1.7545 HDO 1.0807 1.0477 1.0122 0.9950 0.9648 0.9443 0.1797 0.1580
HN
O
N
H
CO2npe
N
N
N
N
O
O
Si
O
OTBS
tBu
tBu
0.98 1.29 0.95 2.03 2.57 1.00 1.03 5.63 1.12 1.10 2.30 1.08 9.25 8.85 3.29 12.29 3.17 3.09
f1 (ppm)

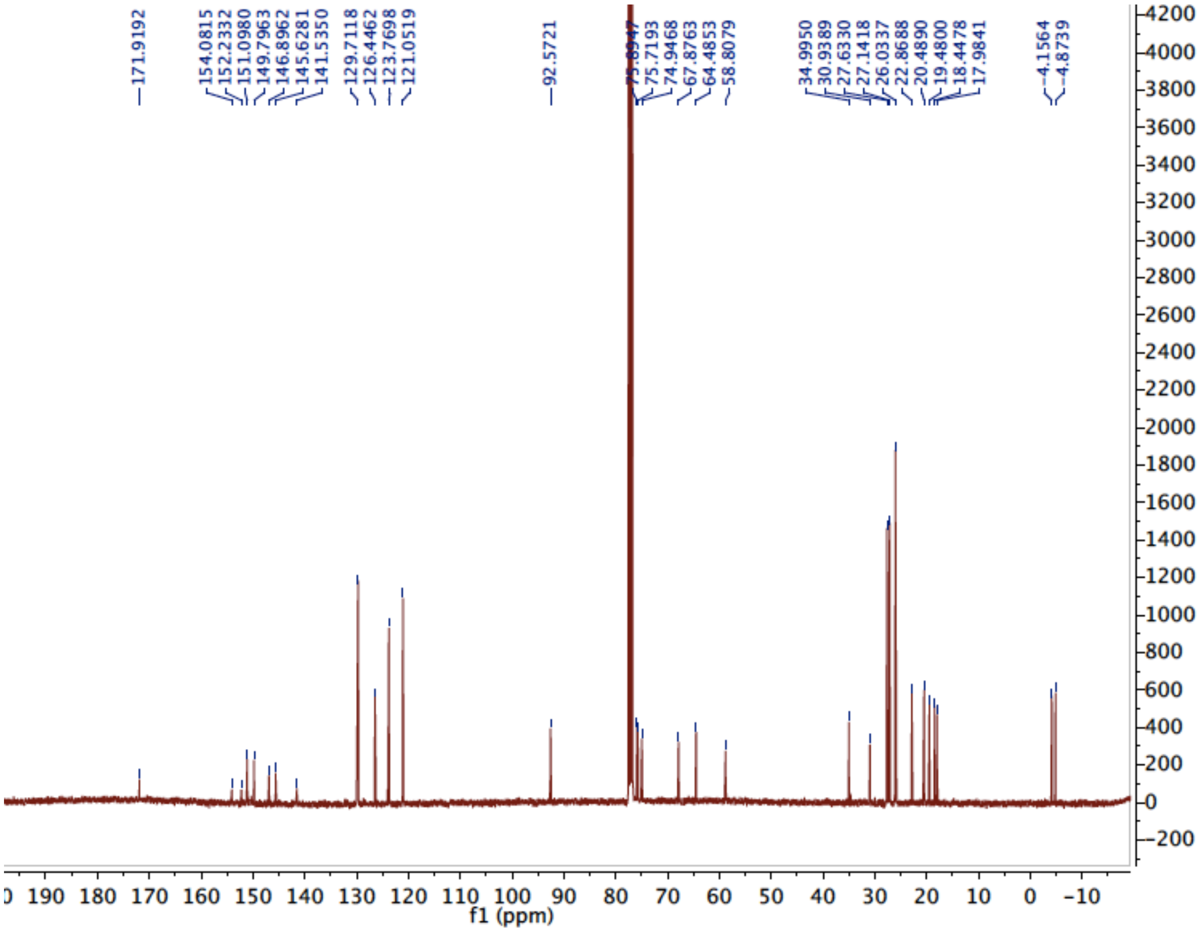
171.9192 154.0815 152.2332 151.0980 149.7963 146.8962 145.6281 141.5350 129.7118 126.4462 123.7698 121.0519 92.5721 75.7193 74.9468 67.8763 64.4853 58.8079 34.9950 30.9389 27.6330 27.1418 26.0337 22.8688 20.4890 19.4800 18.4478 17.9841 -4.1564 -4.8739
f1 (ppm)

Compound 28

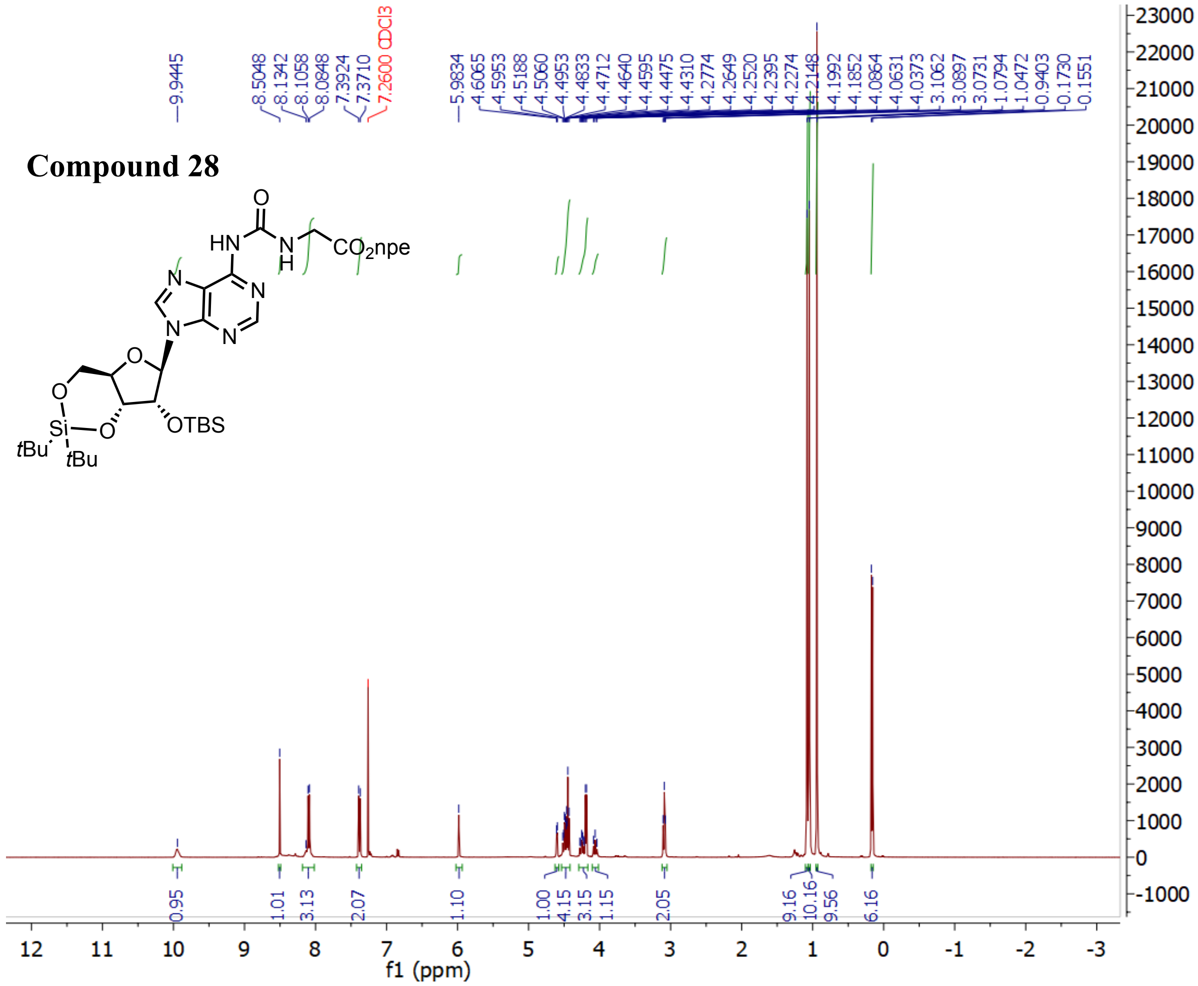
O
HN
N
H
CO2npe
N
N
N
N
O
O
Si
O
OTBS
tBu
tBu
9.9445
8.5048
8.1342
8.1058
8.0848
7.3924
7.3710
7.2600 CDCl3
5.9834
4.6065
4.5953
4.5188
4.5060
4.4953
4.4833
4.4712
4.4640
4.4595
4.4475
4.4310
4.2774
4.2649
4.2520
4.2395
4.2274
4.2148
4.1992
4.1852
4.0864
4.0631
4.0373
3.1062
3.0897
3.0731
1.0794
1.0472
0.9403
0.1730
0.1551
0.95
1.01
3.13
2.07
1.10
1.00
4.15
3.15
1.15
2.05
9.16
10.16
9.56
6.16
12
11
10
9
8
7
6
5
4
3
2
1
0
-1
-2
-3
f1 (ppm)
23000
22000
21000
20000
19000
18000
17000
16000
15000
14000
13000
12000
11000
10000
9000
8000
7000
6000
5000
4000
3000
2000
1000
0
-1000

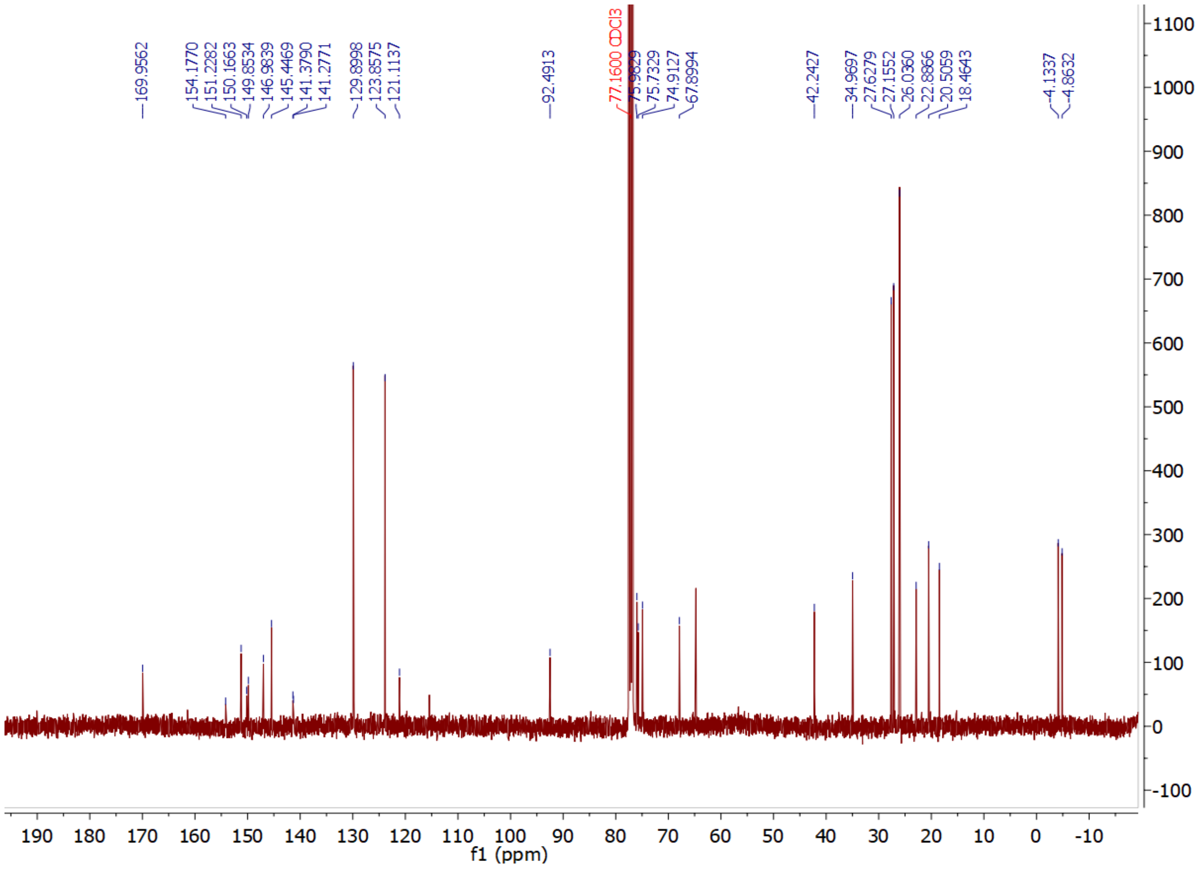
169.9562
154.1770
151.2282
150.1663
149.8534
146.9839
145.4469
141.3790
141.2771
129.8998
123.8575
121.1137
92.4913
77.1600 CDCl3
75.9829
75.7329
74.9127
67.8994
42.2427
34.9697
27.6279
27.1552
26.0360
22.8866
20.5059
18.4643
-4.1337
-4.8632
190
180
170
160
150
140
130
120
110
100
90
80
70
60
50
40
30
20
10
0
-10
f1 (ppm)
1100
1000
900
800
700
600
500
400
300
200
100
0
-100

Compound 29

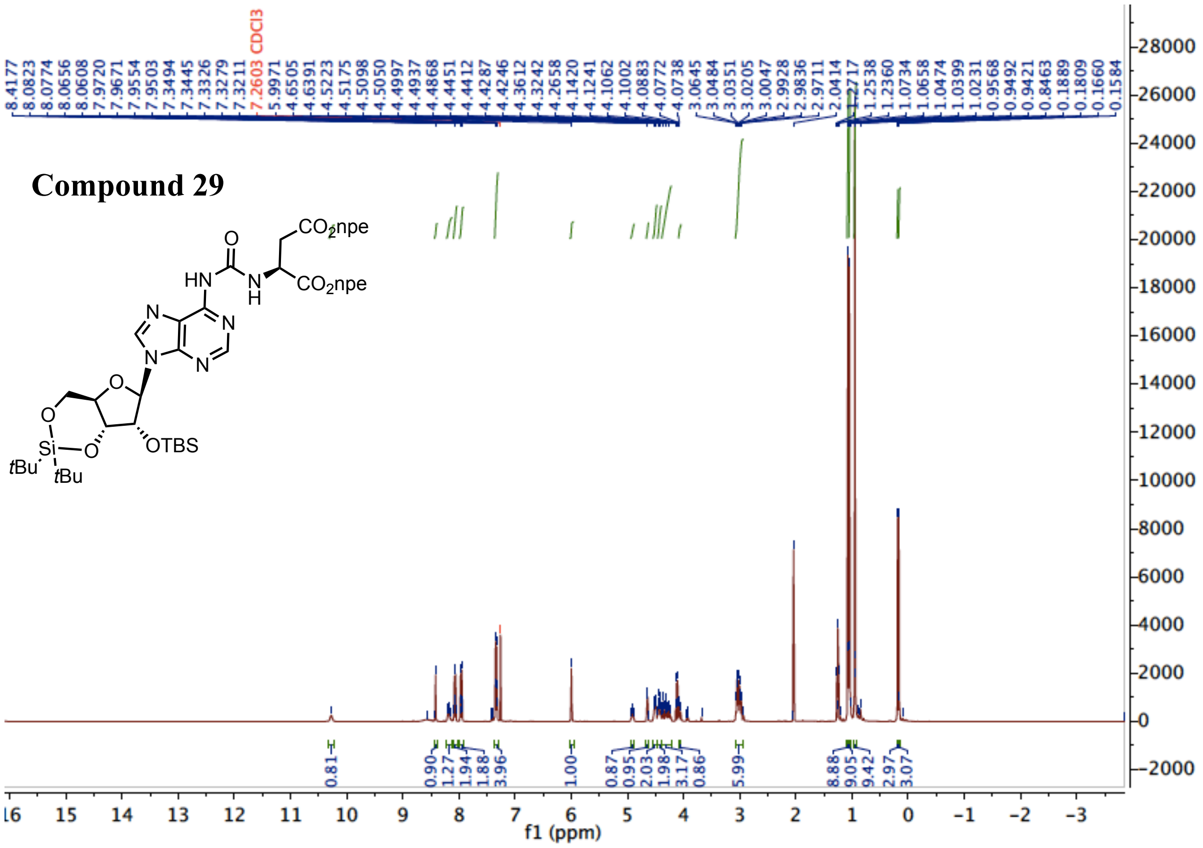

CO2npe
O
HN
N
H
CO2npe
N
N
N
N
O
O
Si
O
OTBS
tBu
tBu
8.4177
8.0823
8.0774
8.0656
8.0608
7.9720
7.9671
7.9554
7.9503
7.3494
7.3445
7.3326
7.3279
7.3211
7.2603 CDCl3
5.9971
4.6505
4.6391
4.5223
4.5175
4.5098
4.5050
4.4997
4.4937
4.4868
4.4451
4.4412
4.4287
4.4246
4.3612
4.3242
4.2658
4.1420
4.1241
4.1062
4.1002
4.0883
4.0772
4.0738
3.0645
3.0484
3.0351
3.0205
3.0047
2.9928
2.9836
2.9711
2.0414
1.2717
1.2538
1.2360
1.0734
1.0658
1.0474
1.0399
1.0231
0.9568
0.9492
0.9421
0.8463
0.1889
0.1809
0.1660
0.1584
0.81
0.90
1.27
1.94
1.88
3.96
1.00
0.87
0.95
2.03
1.98
3.17
0.86
5.99
8.88
9.05
9.42
2.97
3.07
f1 (ppm)

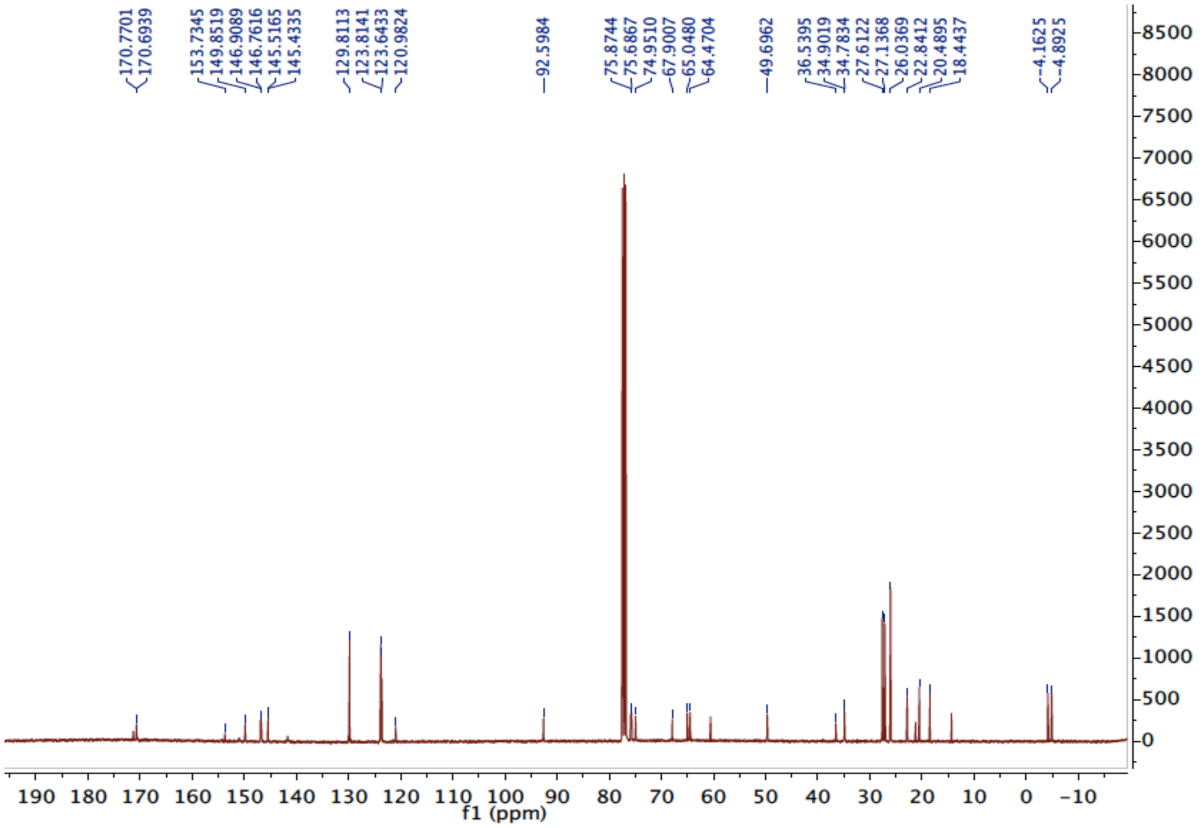

170.7701
170.6939
153.7345
149.8519
146.9089
146.7616
145.5165
145.4335
129.8113
123.8141
123.6433
120.9824
92.5984
75.8744
75.6867
74.9510
67.9007
65.0480
64.4704
49.6962
36.5395
34.9019
34.7834
27.6122
27.1368
26.0369
22.8412
20.4895
18.4437
-4.1625
-4.8925
f1 (ppm)

Compound 30a

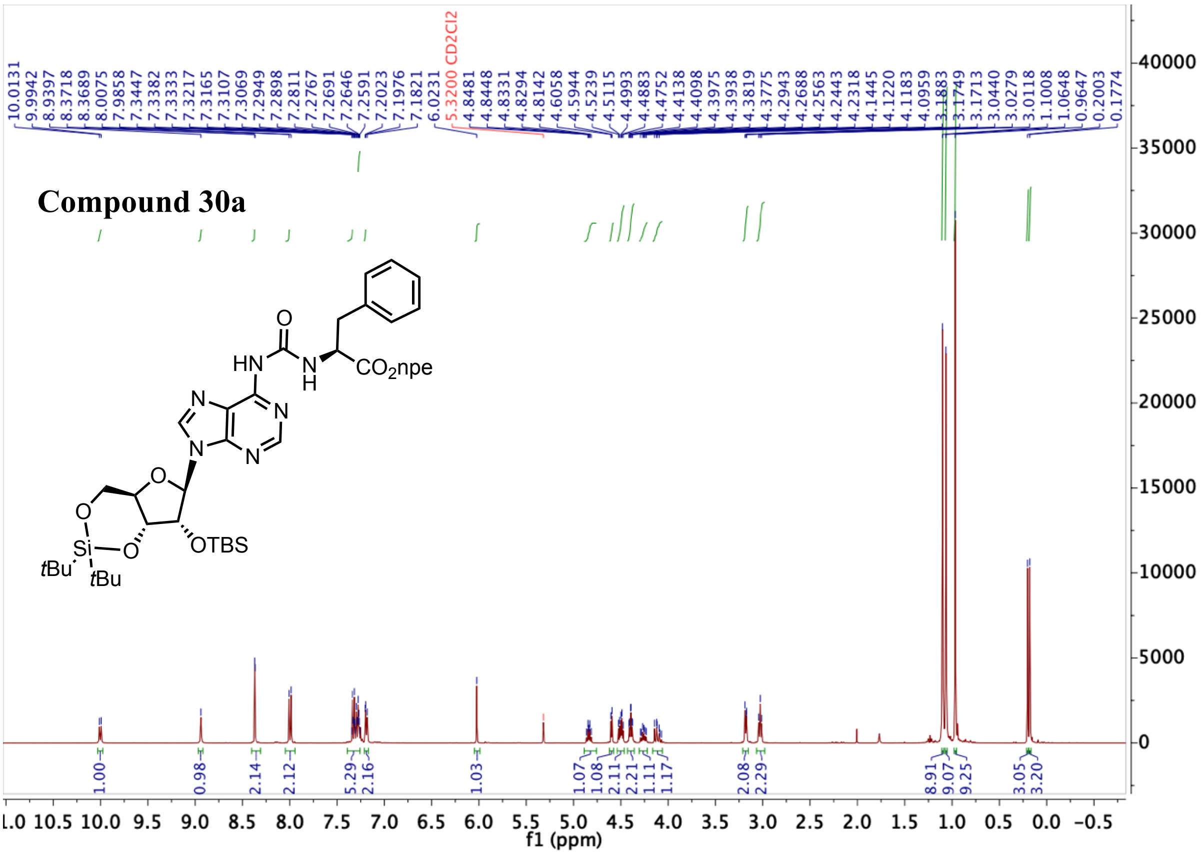

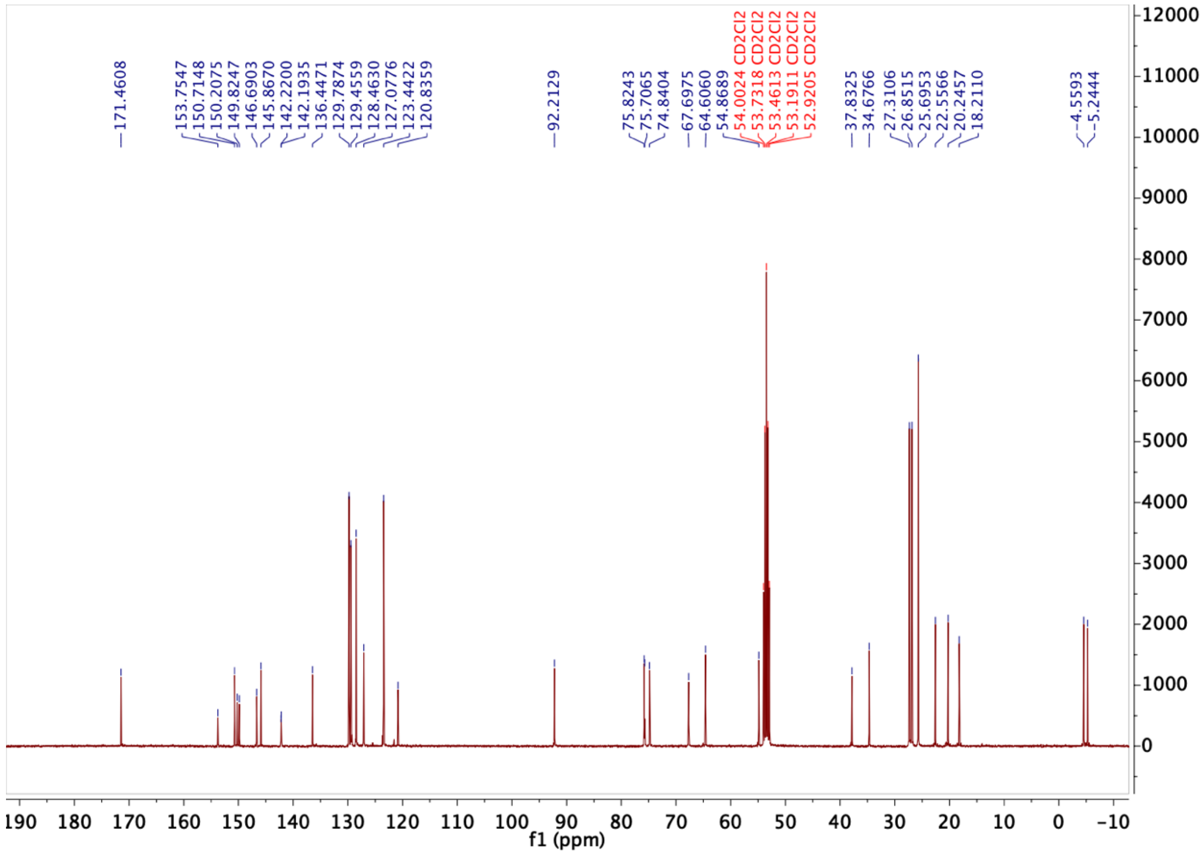

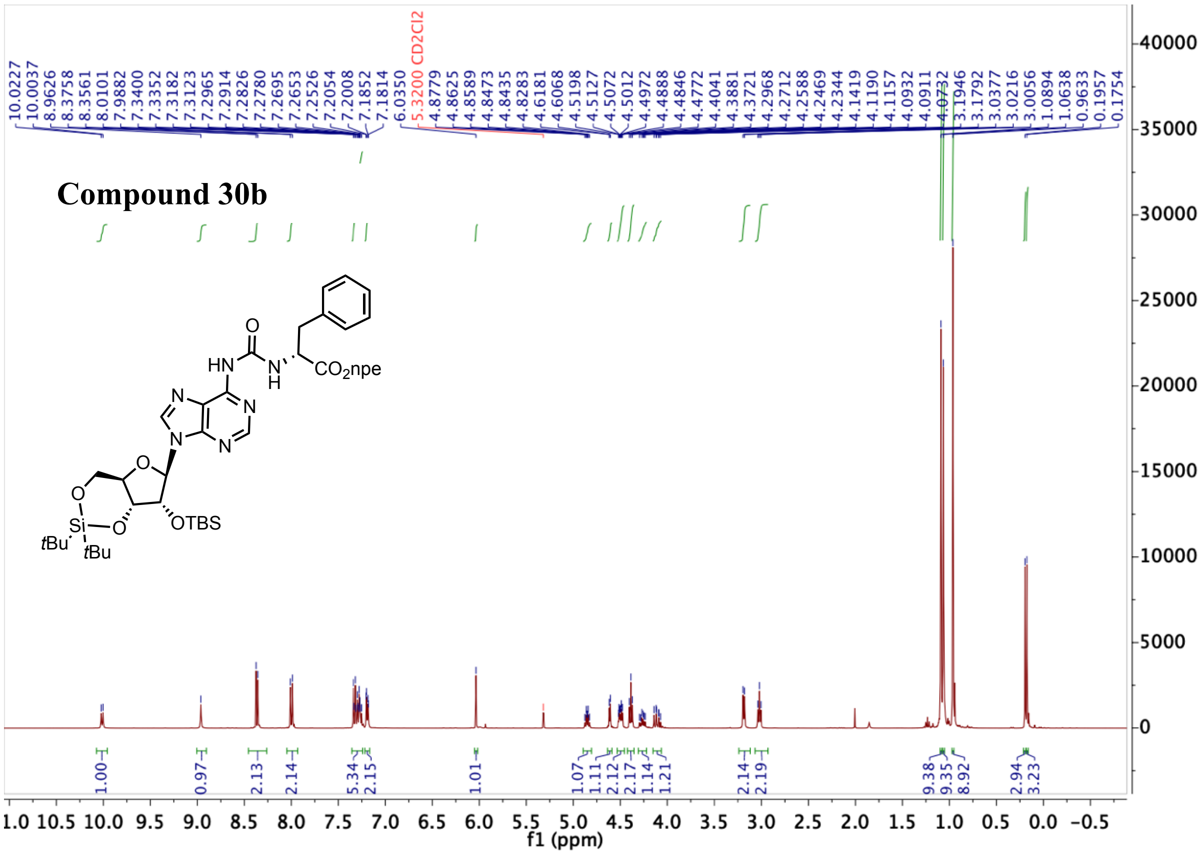
Compound 30b
10.0227
10.0037
8.9626
8.3758
8.3561
8.0101
7.9882
7.3400
7.3352
7.3182
7.3123
7.2965
7.2914
7.2826
7.2780
7.2695
7.2653
7.2526
7.2054
7.2008
7.1852
7.1814
6.0350
5.3200 CD2Cl2
4.8779
4.8625
4.8589
4.8473
4.8435
4.8283
4.6181
4.6068
4.5198
4.5127
4.5072
4.5012
4.4972
4.4888
4.4846
4.4772
4.4041
4.3881
4.3721
4.2968
4.2712
4.2588
4.2469
4.2344
4.1419
4.1190
4.1157
4.0932
4.0911
4.0732
3.1946
3.1792
3.0377
3.0216
3.0056
1.0894
1.0638
0.9633
0.1957
0.1754
HN
CO2npe
OTBS
tBu
tBu
1.00
0.97
2.13
2.14
5.34
2.15
1.01
1.07
1.11
2.12
2.17
1.14
1.21
2.14
2.19
9.38
9.35
8.92
2.94
3.23
f1 (ppm)

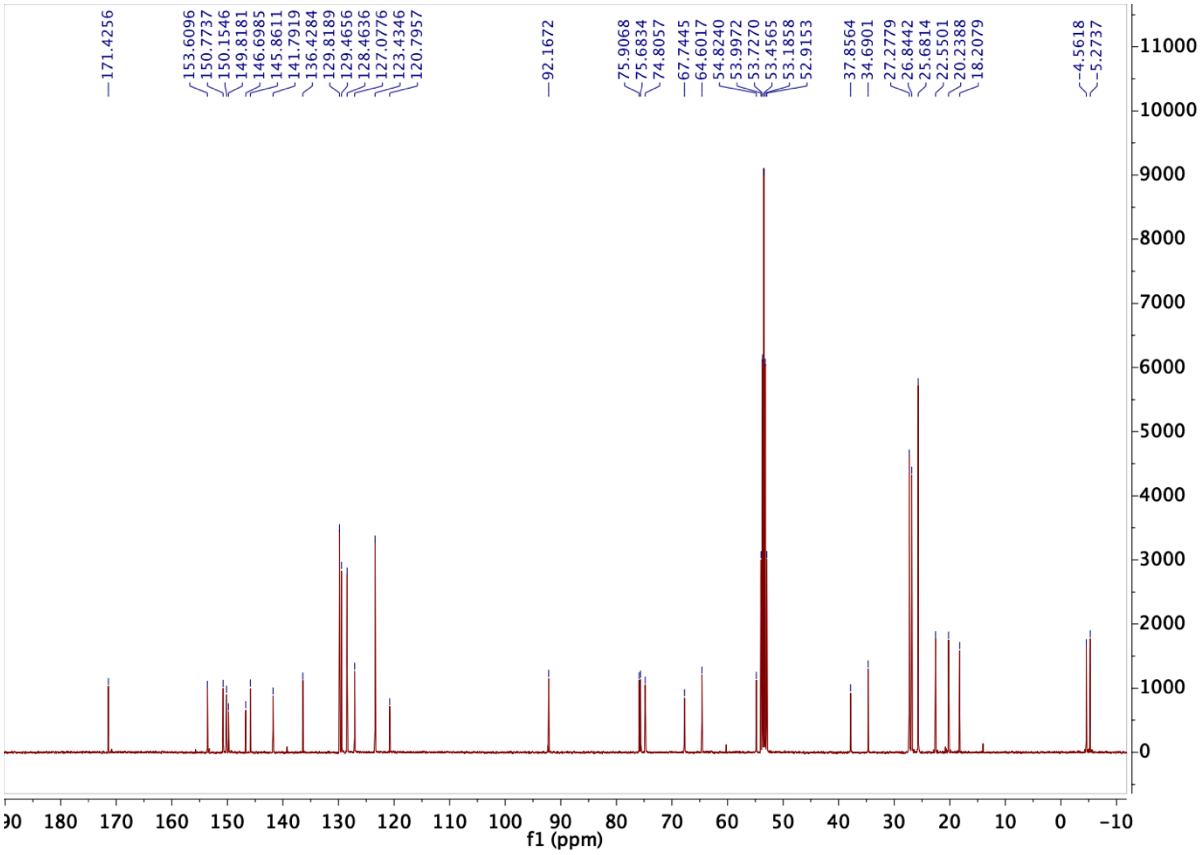
171.4256
153.6096
150.7737
150.1546
149.8181
146.6985
145.8611
141.7919
136.4284
129.8189
129.4656
128.4636
127.0776
123.4346
120.7957
92.1672
75.9068
75.6834
74.8057
67.7445
64.6017
54.8240
53.9972
53.7270
53.4565
53.1858
52.9153
37.8564
34.6901
27.2779
26.8442
25.6814
22.5501
20.2388
18.2079
-4.5618
-5.2737
f1 (ppm)

Compound 31

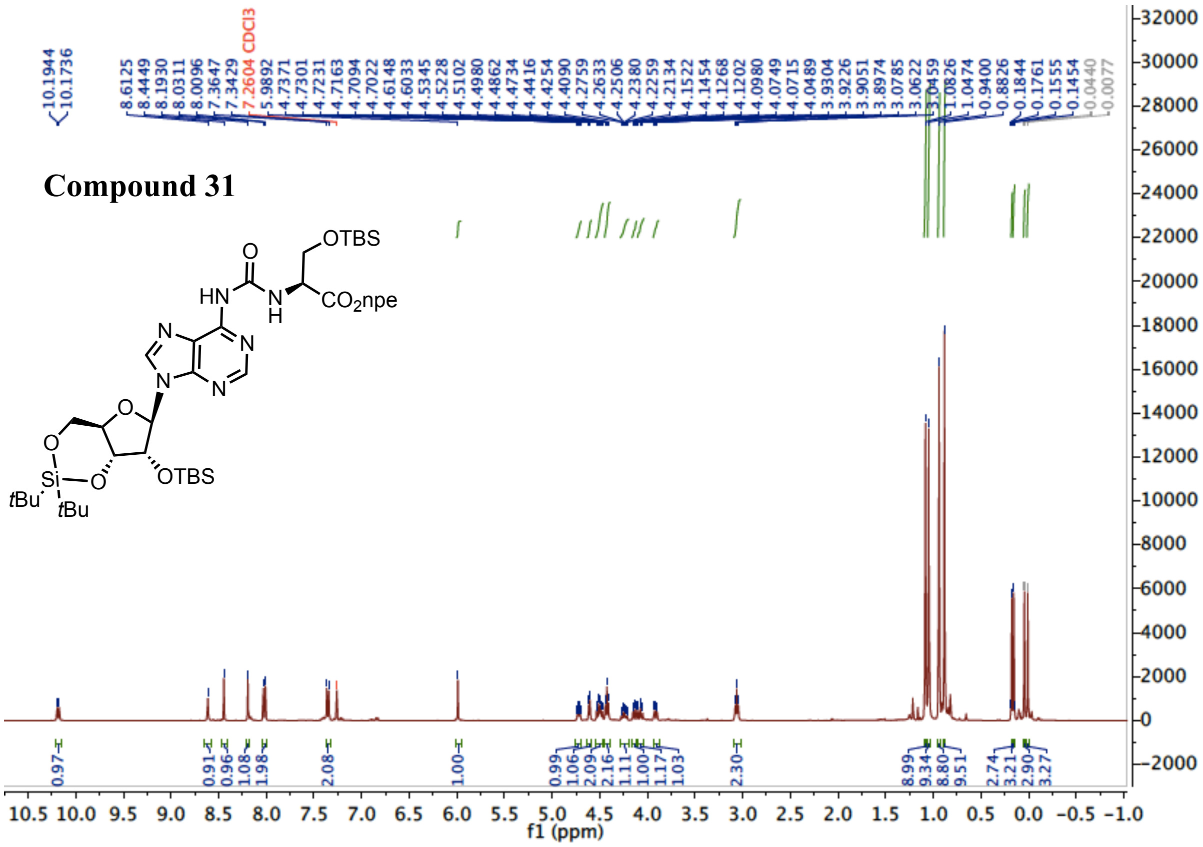

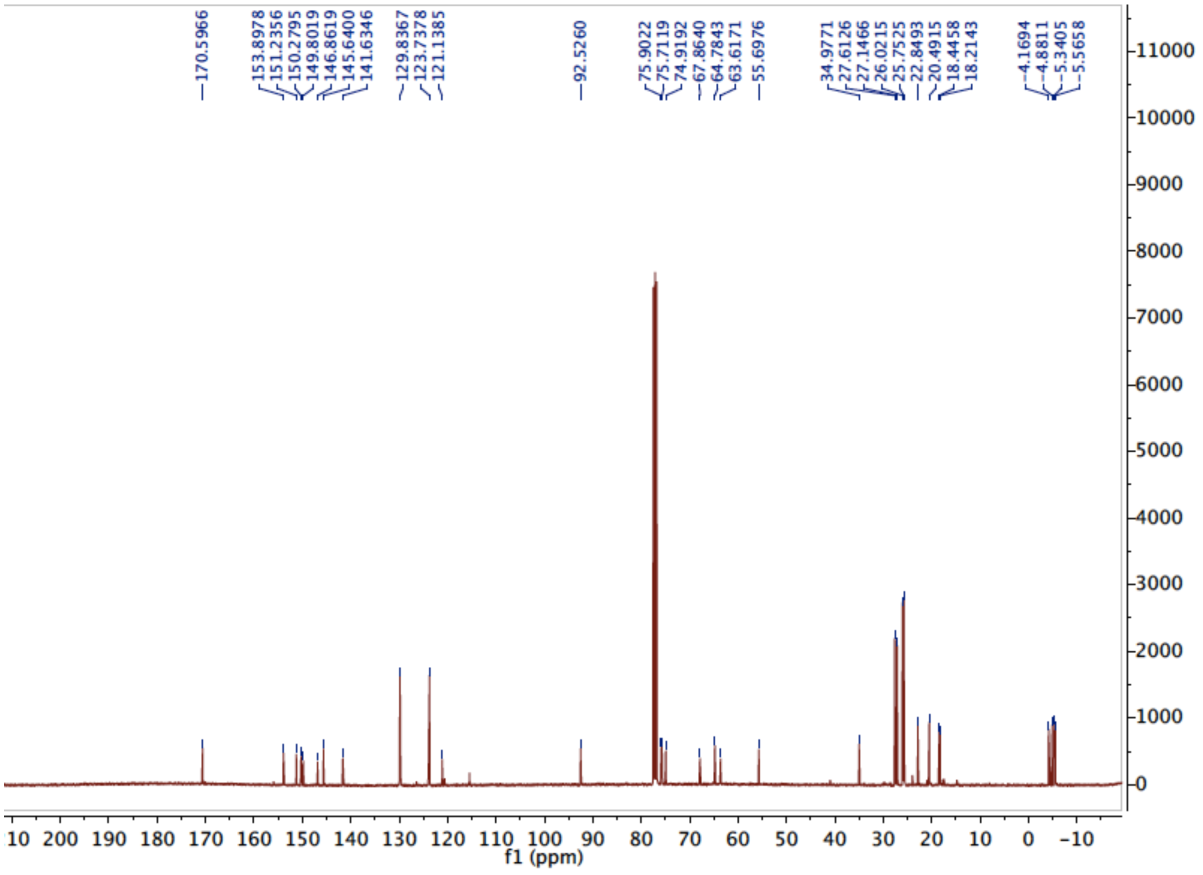

Compound 32

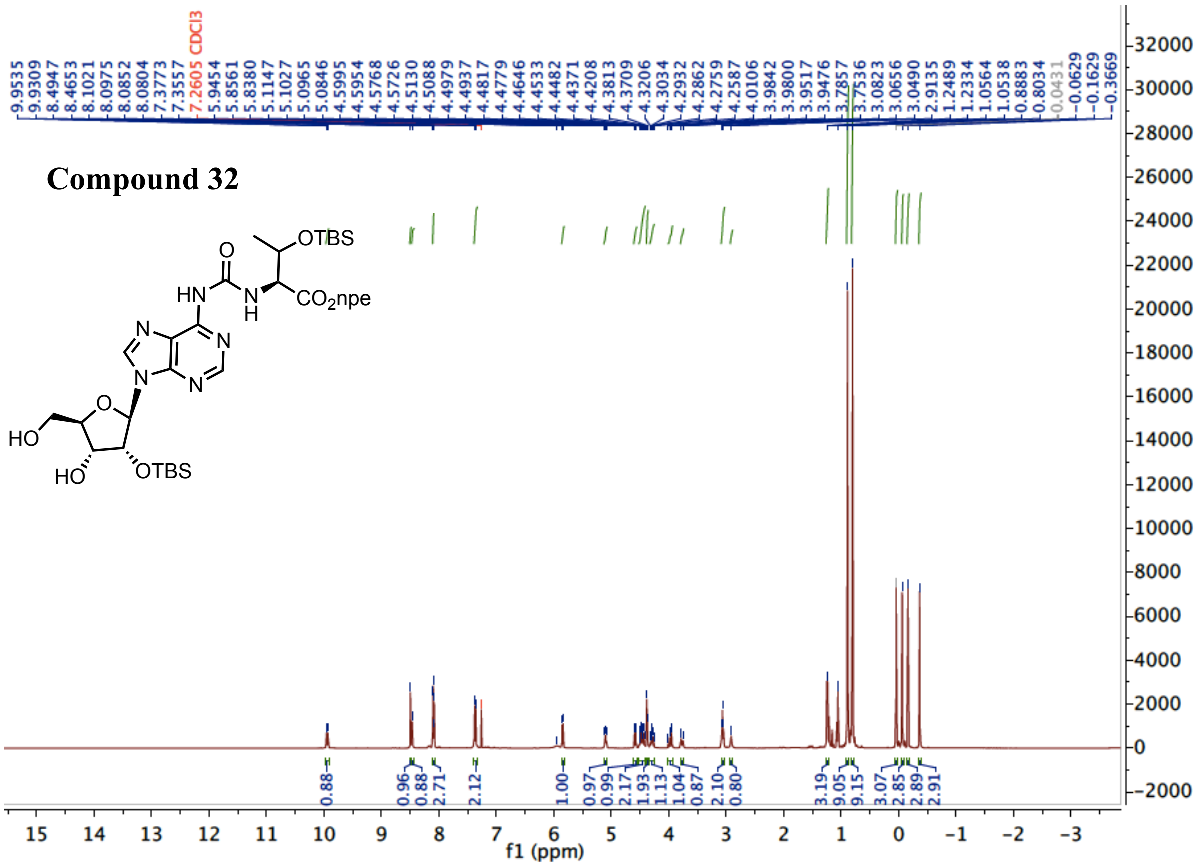

OTBS
O
HN
N
H
CO2npe
N
N
N
N
O
HO
HO
OTBS
9.9535
9.9309
8.4947
8.4653
8.1021
8.0975
8.0852
8.0804
7.3773
7.3557
7.2605 CDCl3
5.9454
5.8561
5.8380
5.1147
5.1027
5.0965
5.0846
4.5995
4.5954
4.5768
4.5726
4.5130
4.5088
4.4979
4.4937
4.4817
4.4779
4.4646
4.4533
4.4482
4.4371
4.4208
4.3813
4.3709
4.3206
4.3034
4.2932
4.2862
4.2759
4.2587
4.0106
3.9842
3.9800
3.9517
3.9476
3.7857
3.7536
3.0823
3.0656
3.0490
2.9135
1.2489
1.2334
1.0564
1.0538
0.8883
0.8034
0.0431
-0.0629
-0.1629
-0.3669
0.88
0.96
0.88
2.71
2.12
1.00
0.97
0.99
2.17
1.93
1.13
1.04
0.87
2.10
0.80
3.19
9.05
9.15
3.07
2.85
2.89
2.91
f1 (ppm)

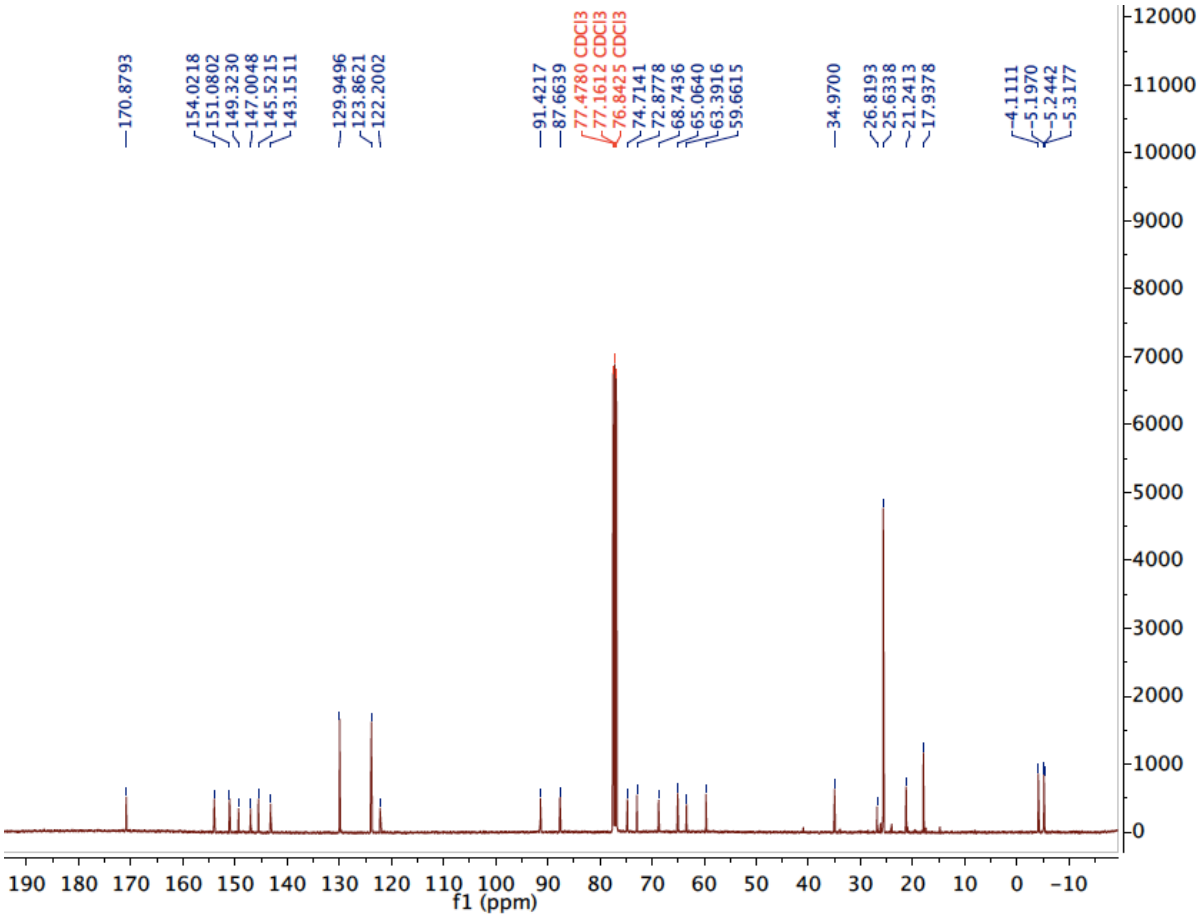

170.8793
154.0218
151.0802
149.3230
147.0048
145.5215
143.1511
129.9496
123.8621
122.2002
91.4217
87.6639
77.4780 CDCl3
77.1612 CDCl3
76.8425 CDCl3
74.7141
72.8778
68.7436
65.0640
63.3916
59.6615
34.9700
26.8193
25.6338
21.2413
17.9378
-4.1111
-5.1970
-5.2442
-5.3177
f1 (ppm)

Compound 33

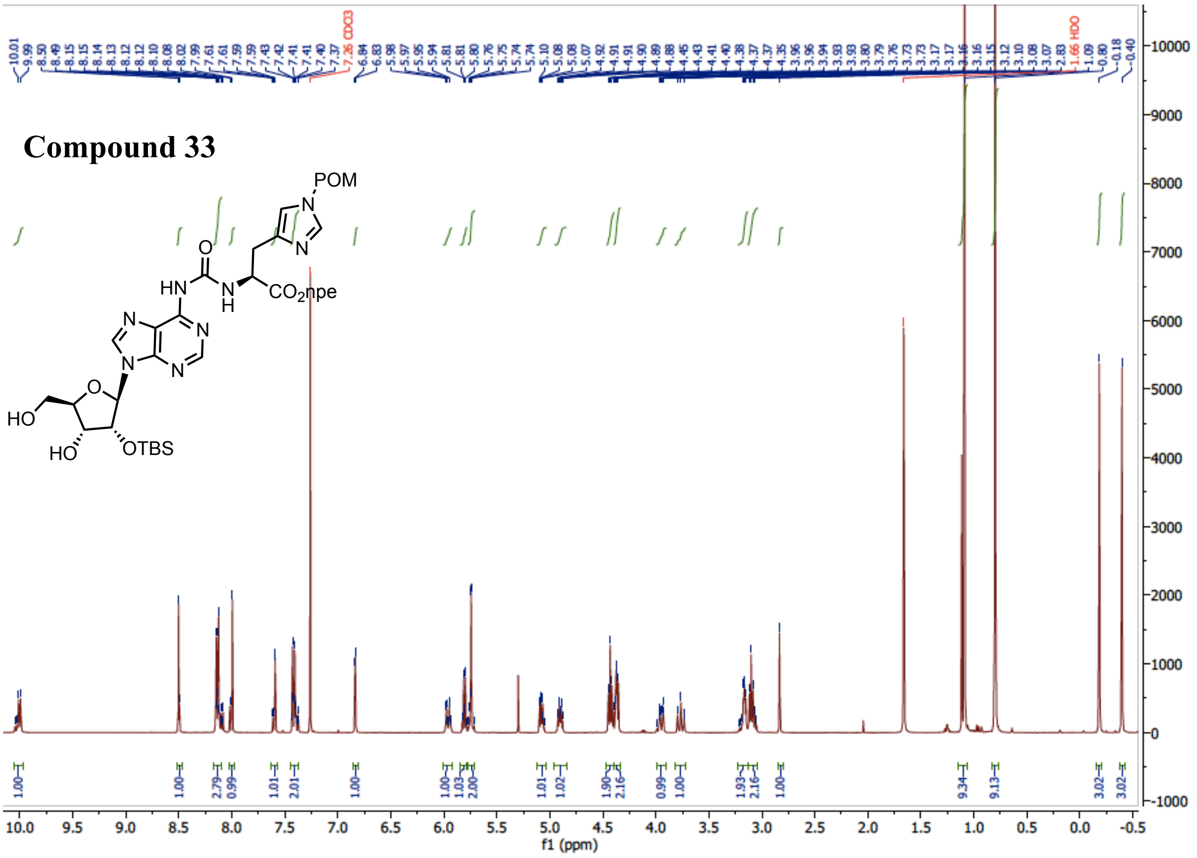

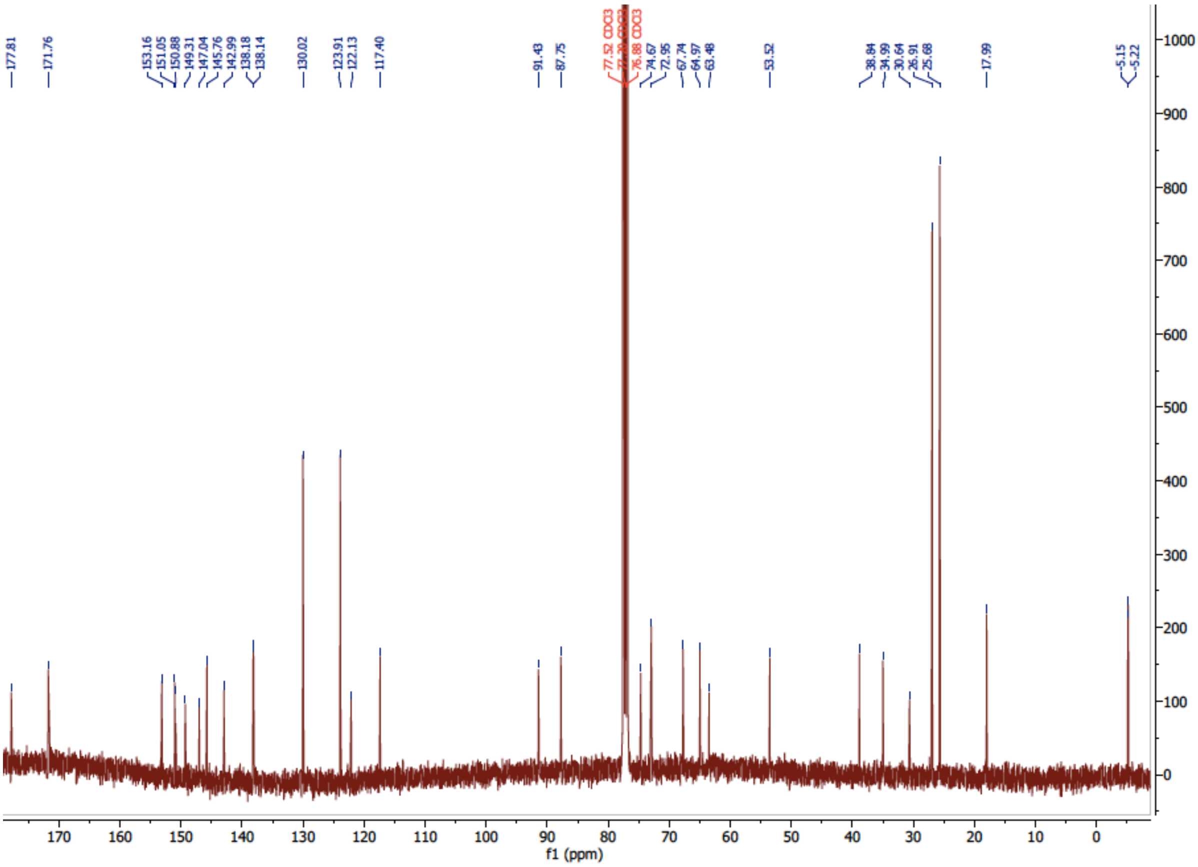

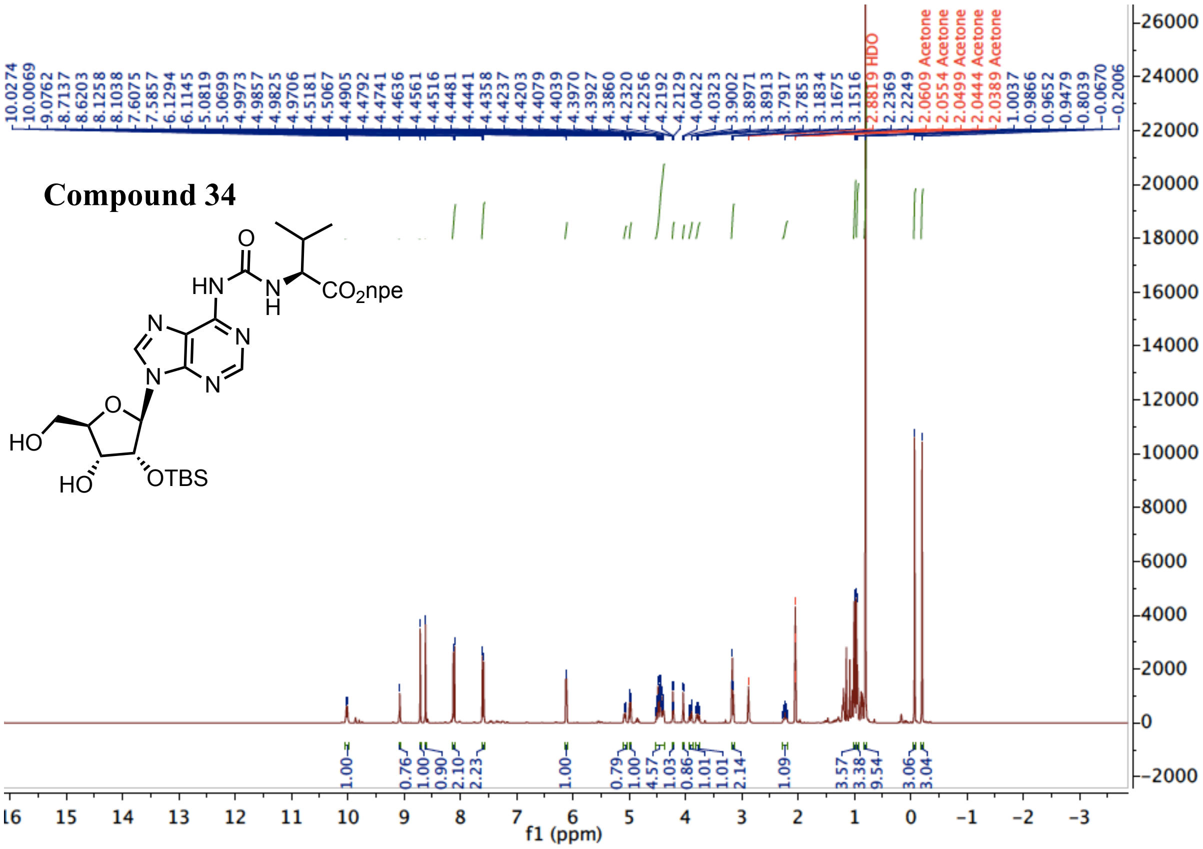

Compound 34
HN
N
H
O
CO2npe
N
N
N
N
O
HO
HO
OTBS
2.8819 HDO
2.0609 Acetone
2.0554 Acetone
2.0499 Acetone
2.0444 Acetone
2.0389 Acetone
f1 (ppm)

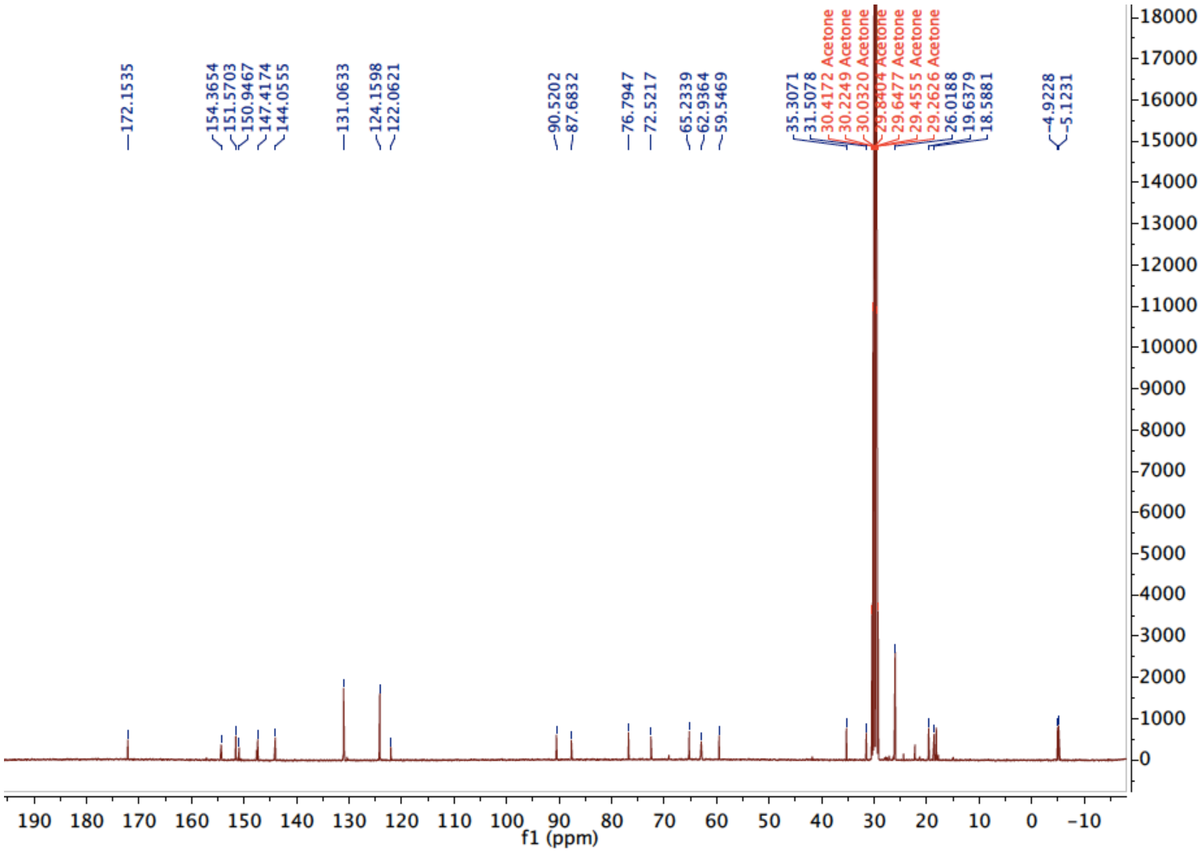

172.1535
154.3654
151.5703
150.9467
147.4174
144.0555
131.0633
124.1598
122.0621
90.5202
87.6832
76.7947
72.5217
65.2339
62.9364
59.5469
35.3071
31.5078
30.4172 Acetone
30.2249 Acetone
30.0320 Acetone
29.8404 Acetone
29.6477 Acetone
29.4555 Acetone
29.2626 Acetone
26.0188
19.6379
18.5881
-4.9228
-5.1231
f1 (ppm)

Compound 35

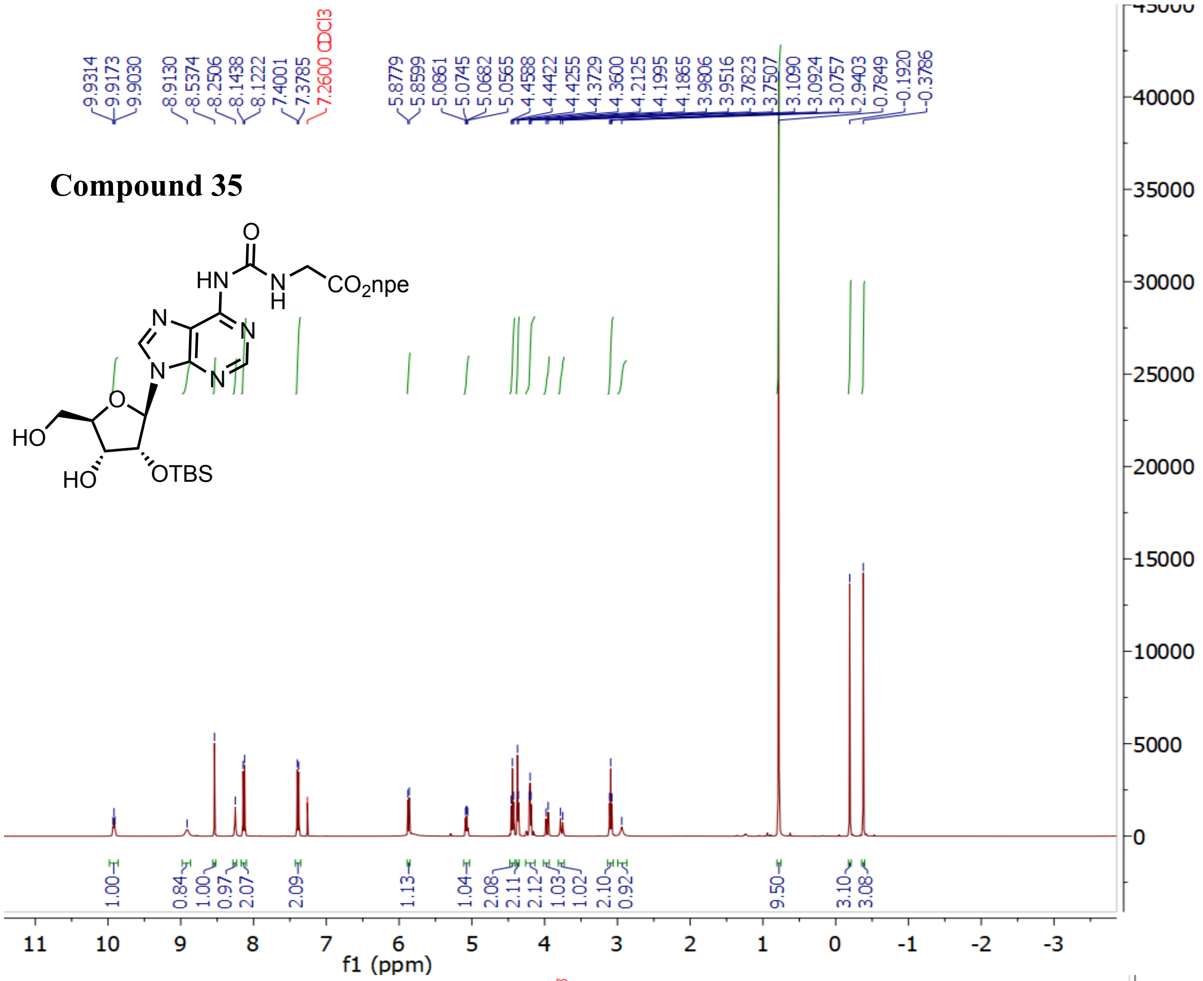
9.9314
9.9173
9.9030
8.9130
8.5374
8.2506
8.1438
8.1222
7.4001
7.3785
7.2600 CDCl3
5.8779
5.8599
5.0861
5.0745
5.0682
5.0565
4.4588
4.4422
4.4255
4.3729
4.3600
4.2125
4.1995
4.1865
3.9806
3.9516
3.7823
3.7507
3.1090
3.0924
3.0757
2.9403
0.7849
-0.1920
-0.3786
O
HN
N
H
CO2npe
N
N
N
N
O
HO
HO
OTBS
1.00
0.84
1.00
0.97
2.07
2.09
1.13
1.04
2.08
2.11
2.12
1.03
1.02
2.10
0.92
9.50
3.10
3.08
f1 (ppm)

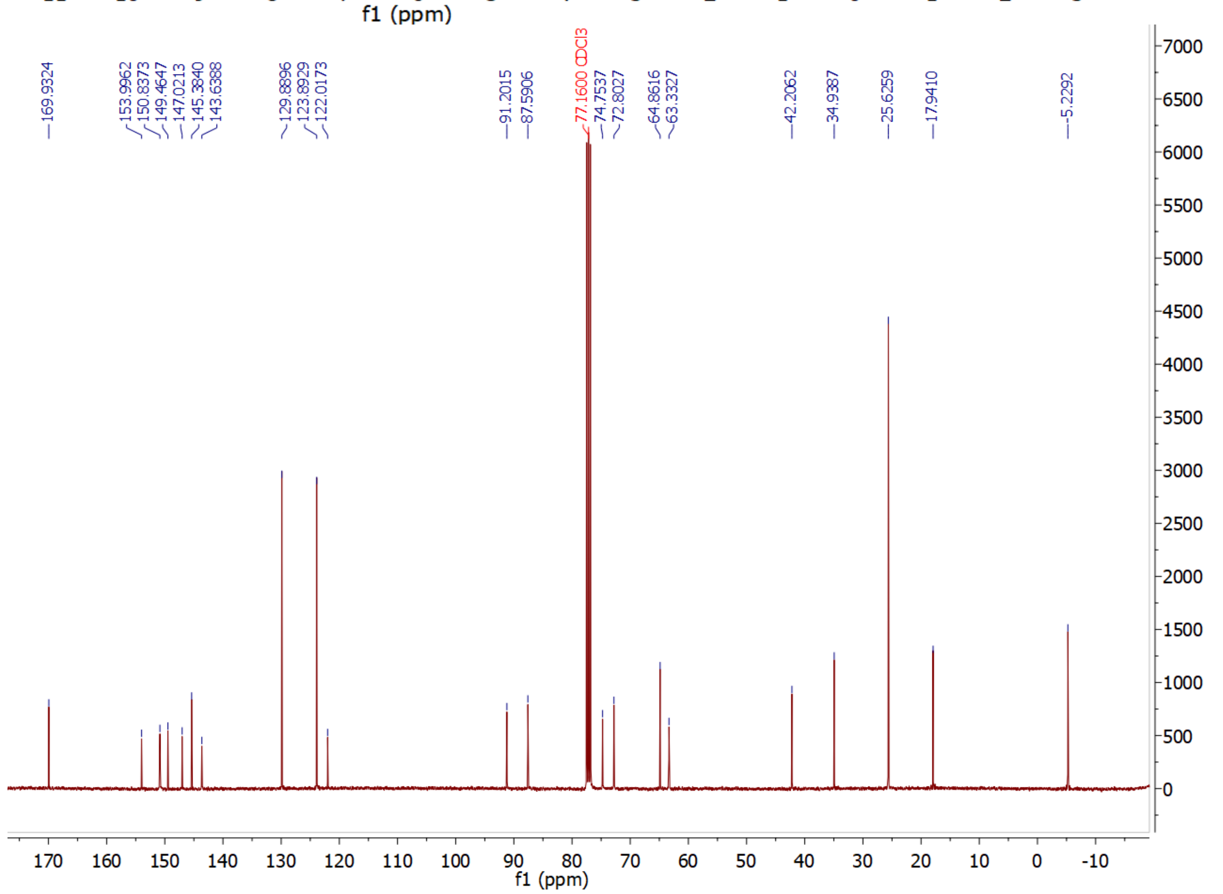
169.9324
153.9962
150.8373
149.4647
147.0213
145.3840
143.6388
129.8896
123.8929
122.0173
91.2015
87.5906
77.1600 CDCl3
74.7537
72.8027
64.8616
63.3327
42.2062
34.9387
25.6259
17.9410
-5.2292
f1 (ppm)

Compound 36

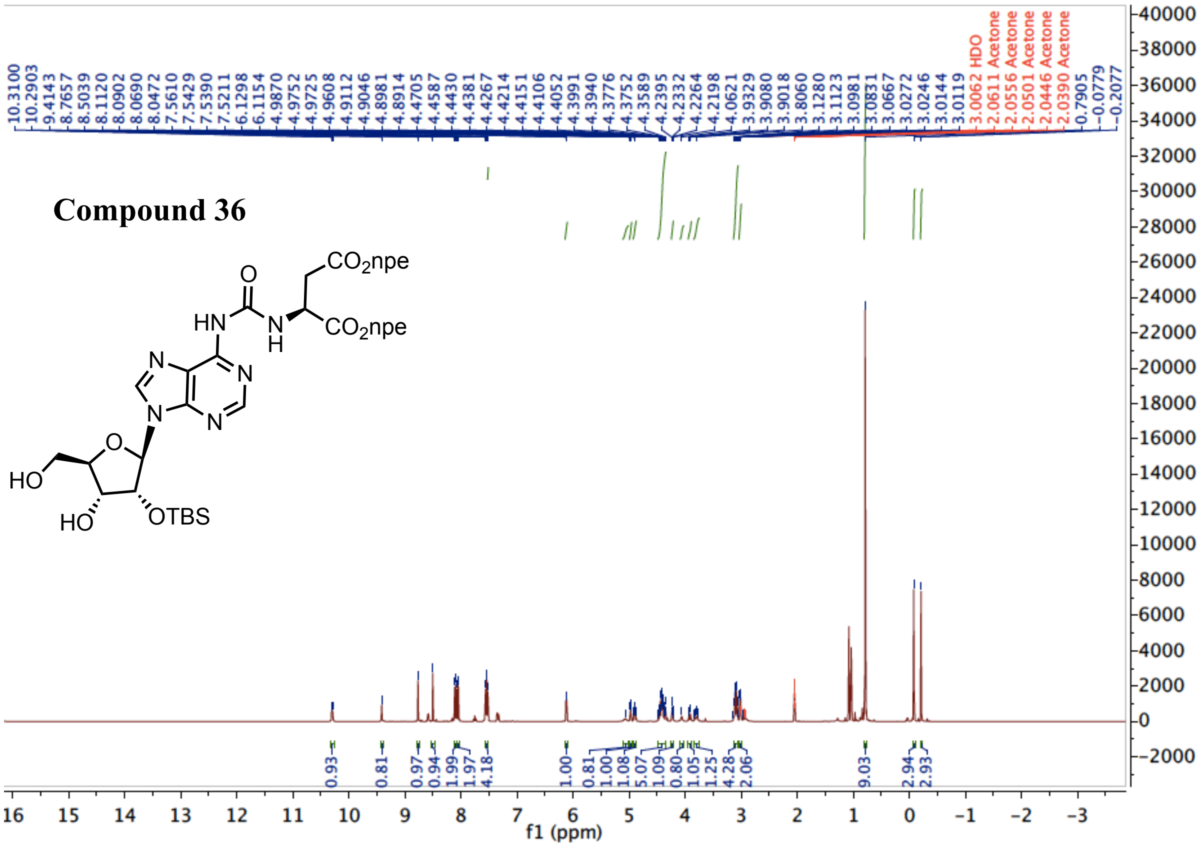
10.3100
10.2903
9.4143
8.7657
8.5039
8.1120
8.0902
8.0690
8.0472
7.5610
7.5429
7.5390
7.5211
6.1298
6.1154
4.9870
4.9752
4.9725
4.9608
4.9112
4.9046
4.8981
4.8914
4.4705
4.4587
4.4430
4.4381
4.4267
4.4214
4.4151
4.4106
4.4052
4.3991
4.3940
4.3776
4.3752
4.3589
4.2395
4.2332
4.2264
4.2198
4.0621
3.9329
3.9080
3.9018
3.8060
3.1280
3.1123
3.0981
3.0831
3.0667
3.0272
3.0246
3.0144
3.0119
3.0062 HDO
2.0611 Acetone
2.0556 Acetone
2.0501 Acetone
2.0446 Acetone
2.0390 Acetone
0.7905
-0.0779
-0.2077
CO2npe
O
HN
N
H
CO2npe
N
N
N
N
O
HO
HO
OTBS
0.93
0.81
0.97
0.94
1.99
1.97
4.18
1.00
0.81
1.00
1.08
5.07
1.09
0.80
1.05
1.25
4.28
2.06
9.03
2.94
2.93
f1 (ppm)

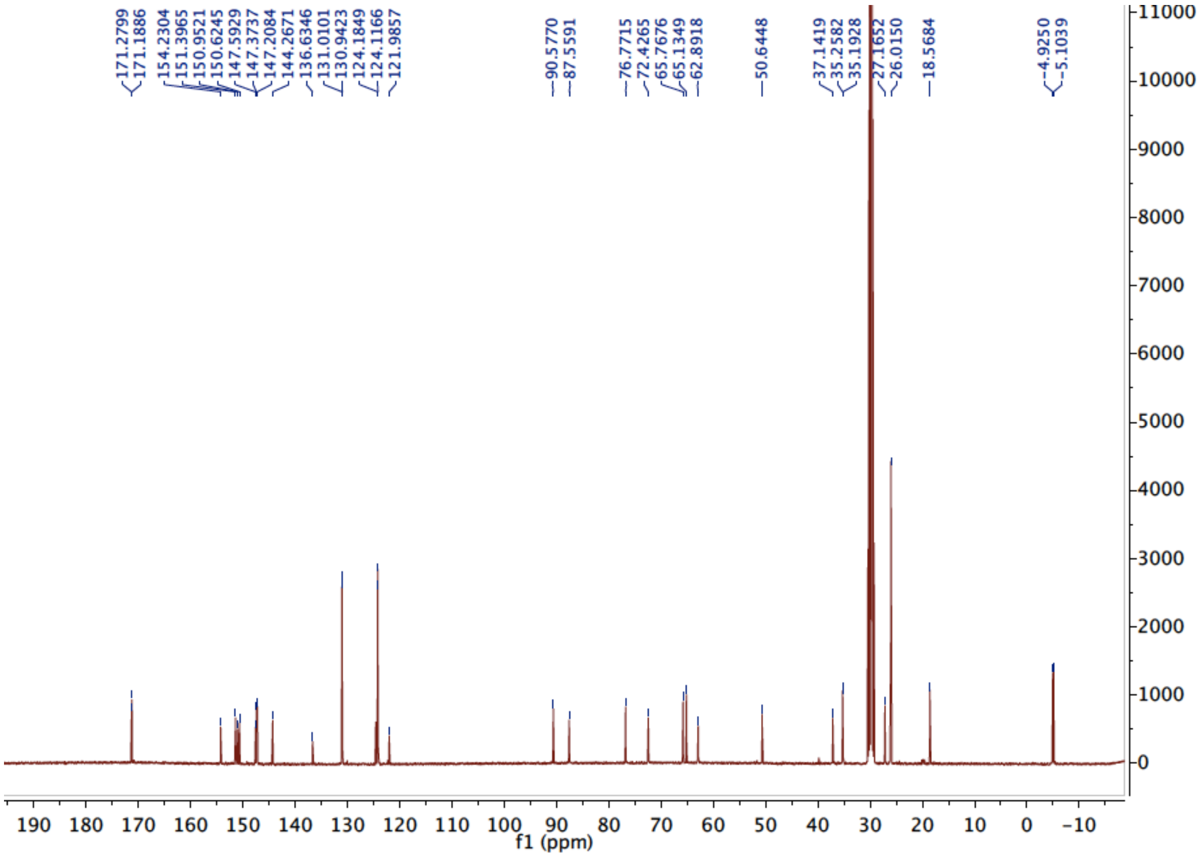
171.2799
171.1886
154.2304
151.3965
150.9521
150.6245
147.5929
147.3737
147.2084
144.2671
136.6346
131.0101
130.9423
124.1849
124.1166
121.9857
90.5770
87.5591
76.7715
72.4265
65.7676
65.1349
62.8918
50.6448
37.1419
35.2582
35.1928
27.1652
26.0150
18.5684
-4.9250
-5.1039
f1 (ppm)

Compound 37a

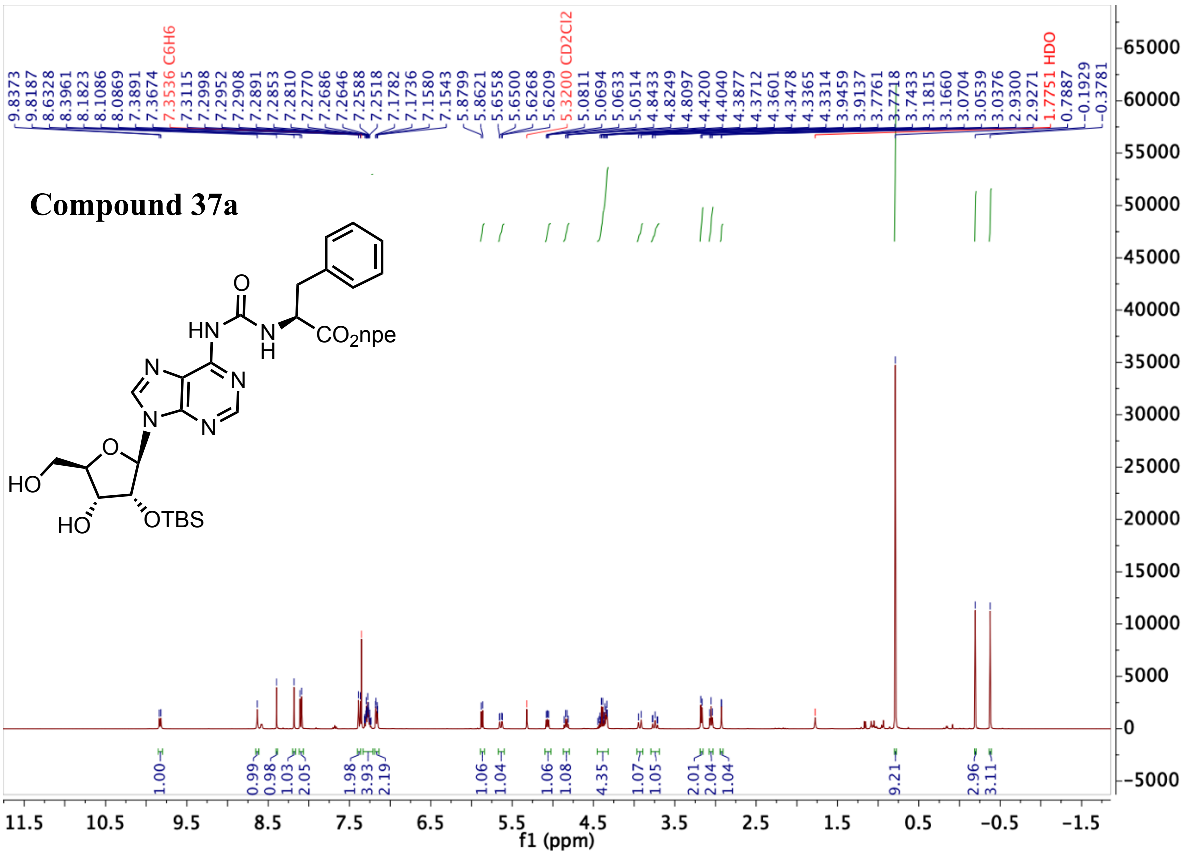

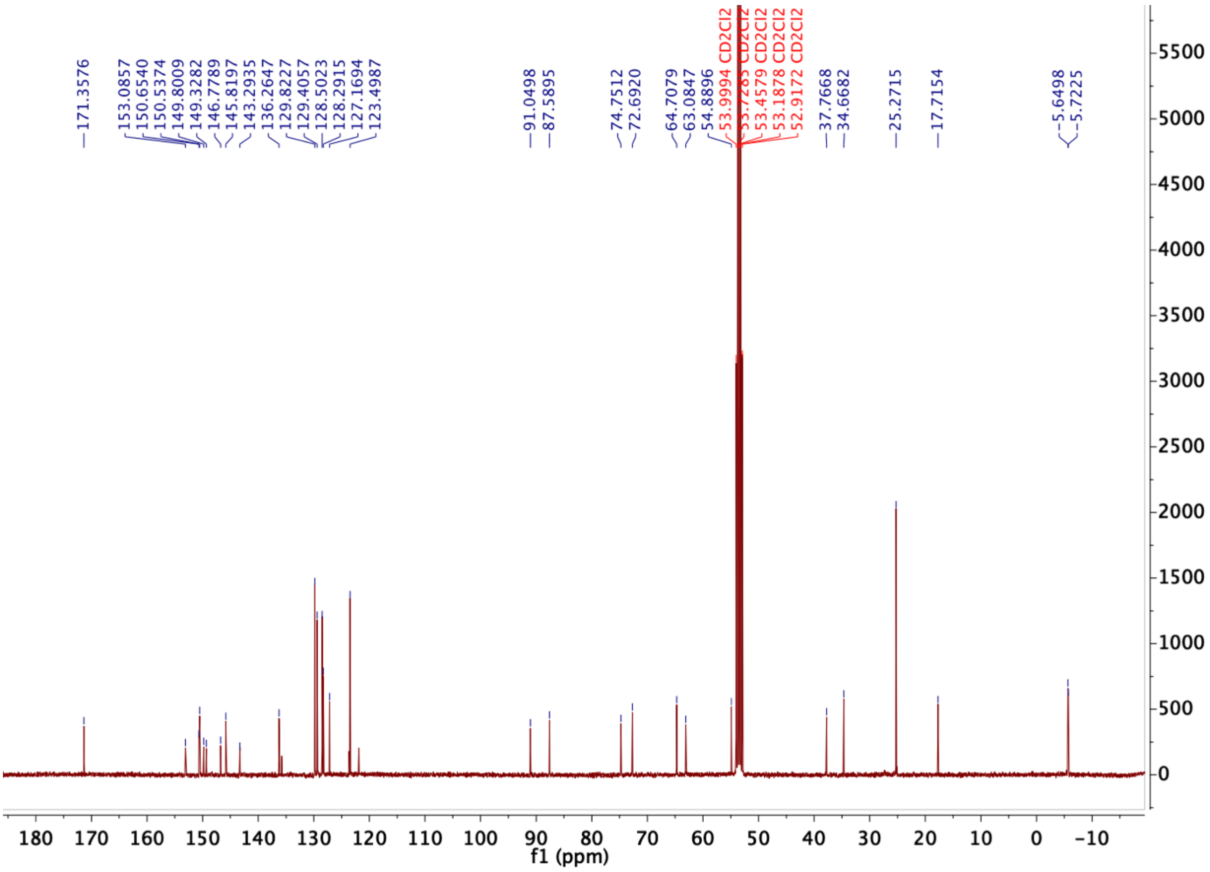

Compound 37b

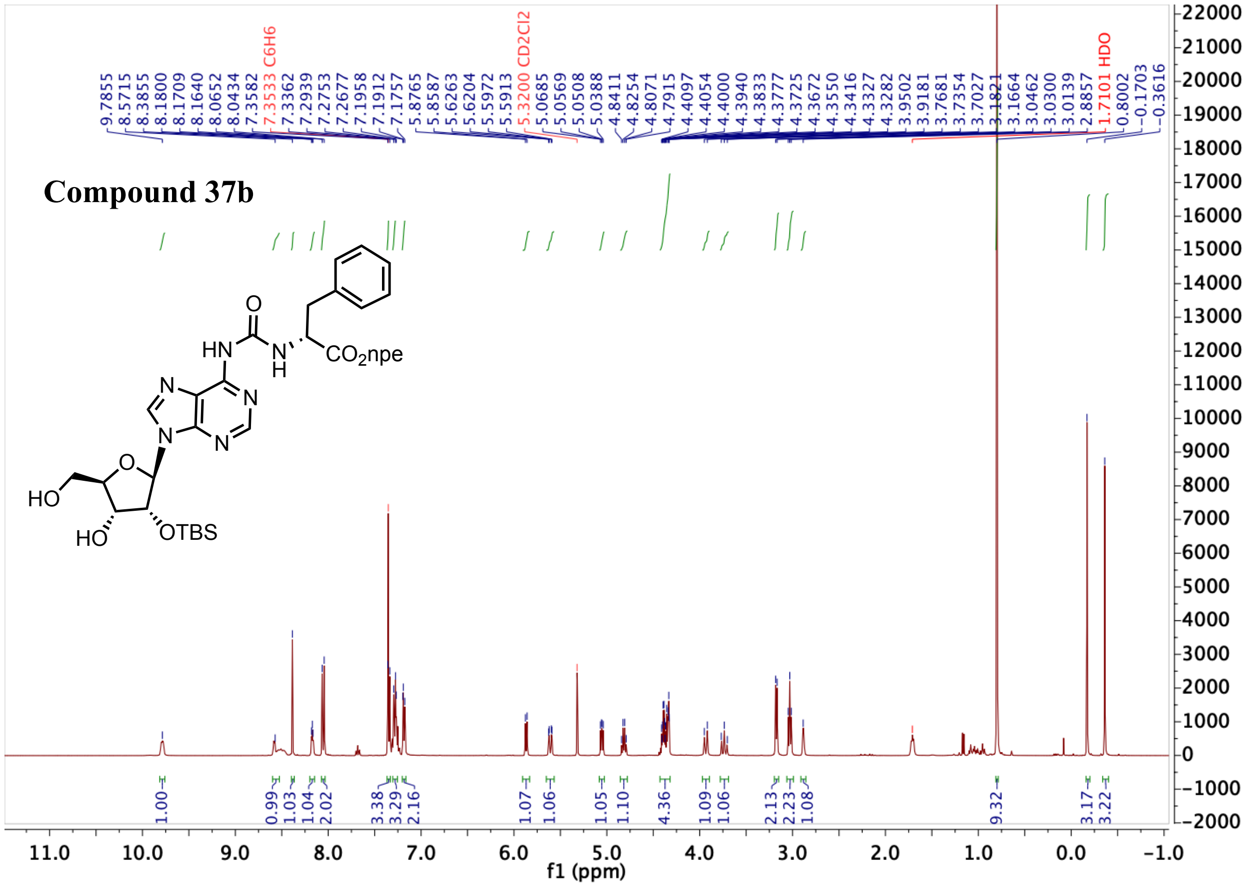

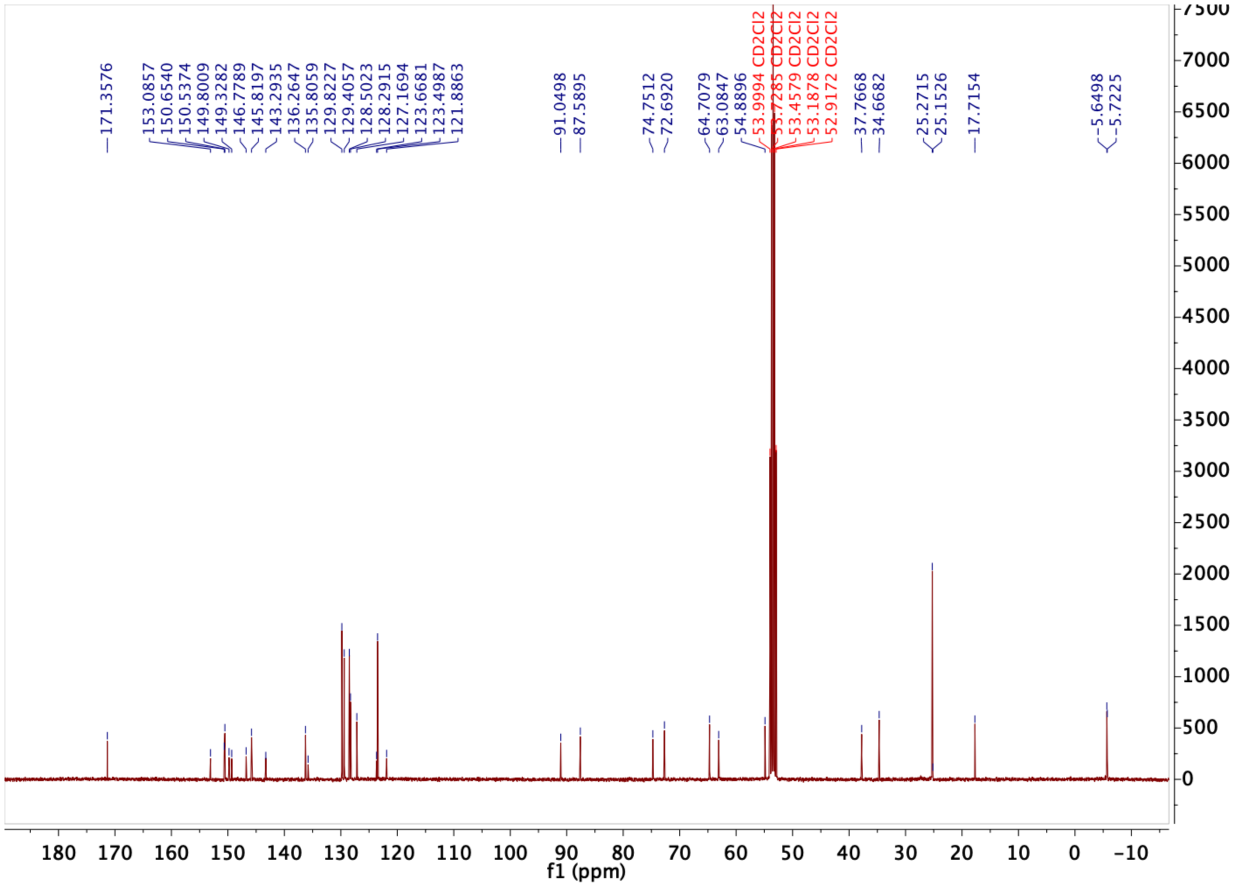

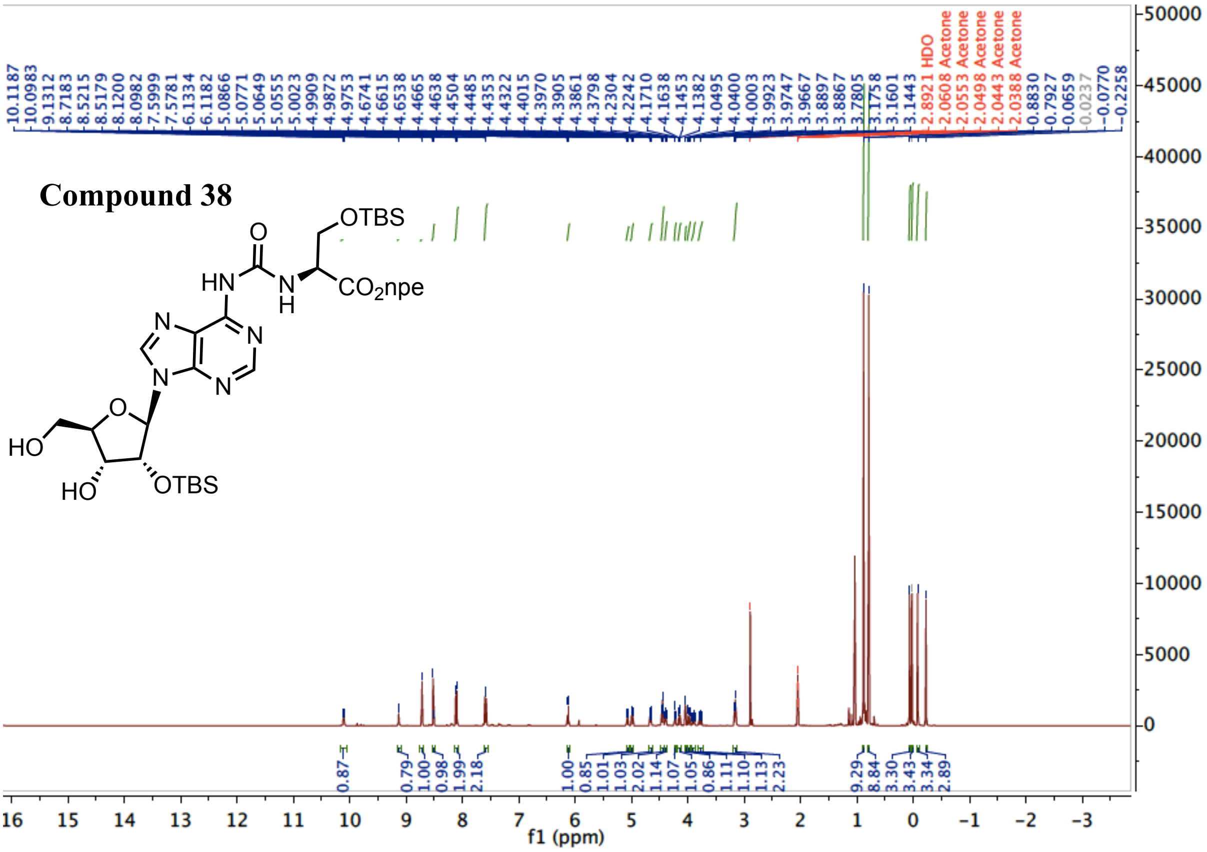
Compound 38
OTBS
O
HN
N
H
CO2npe
N
N
N
N
O
HO
HO
OTBS
f1 (ppm)

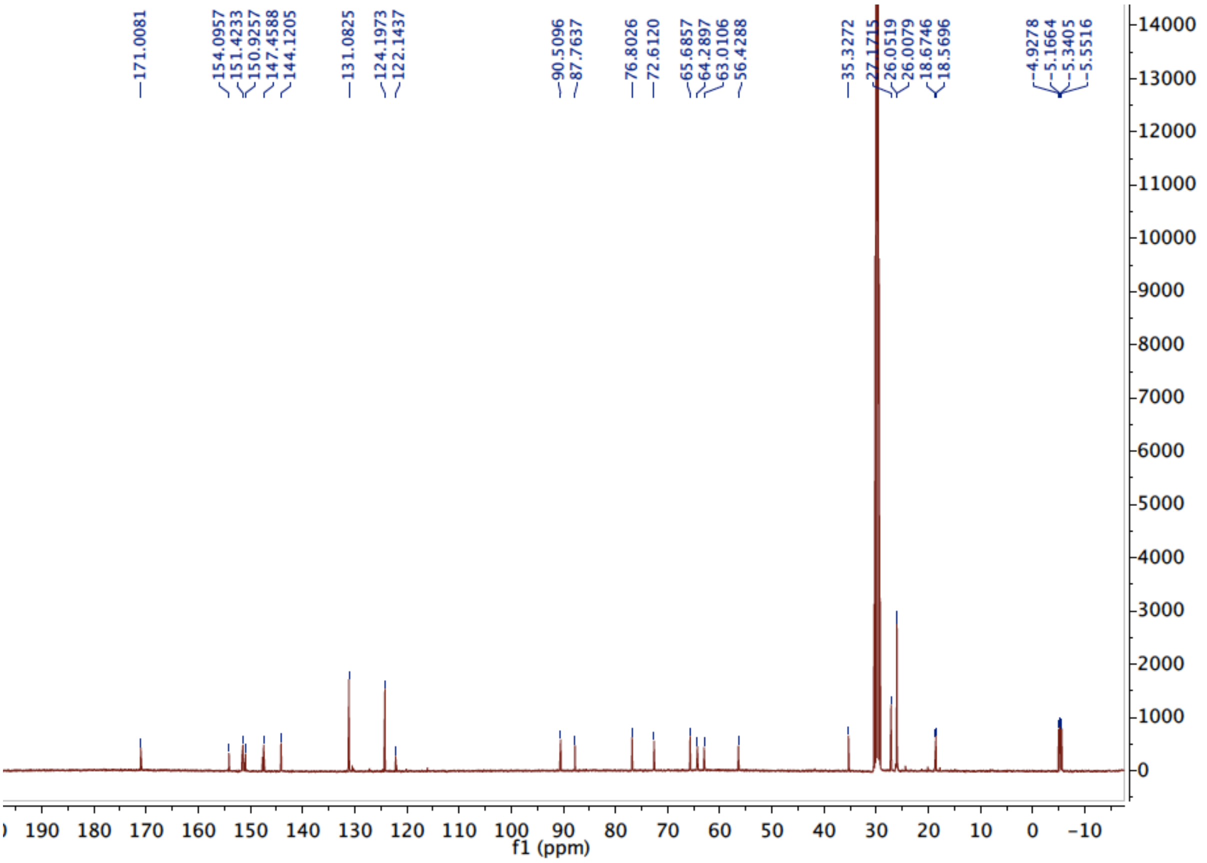
f1 (ppm)

Compound 39

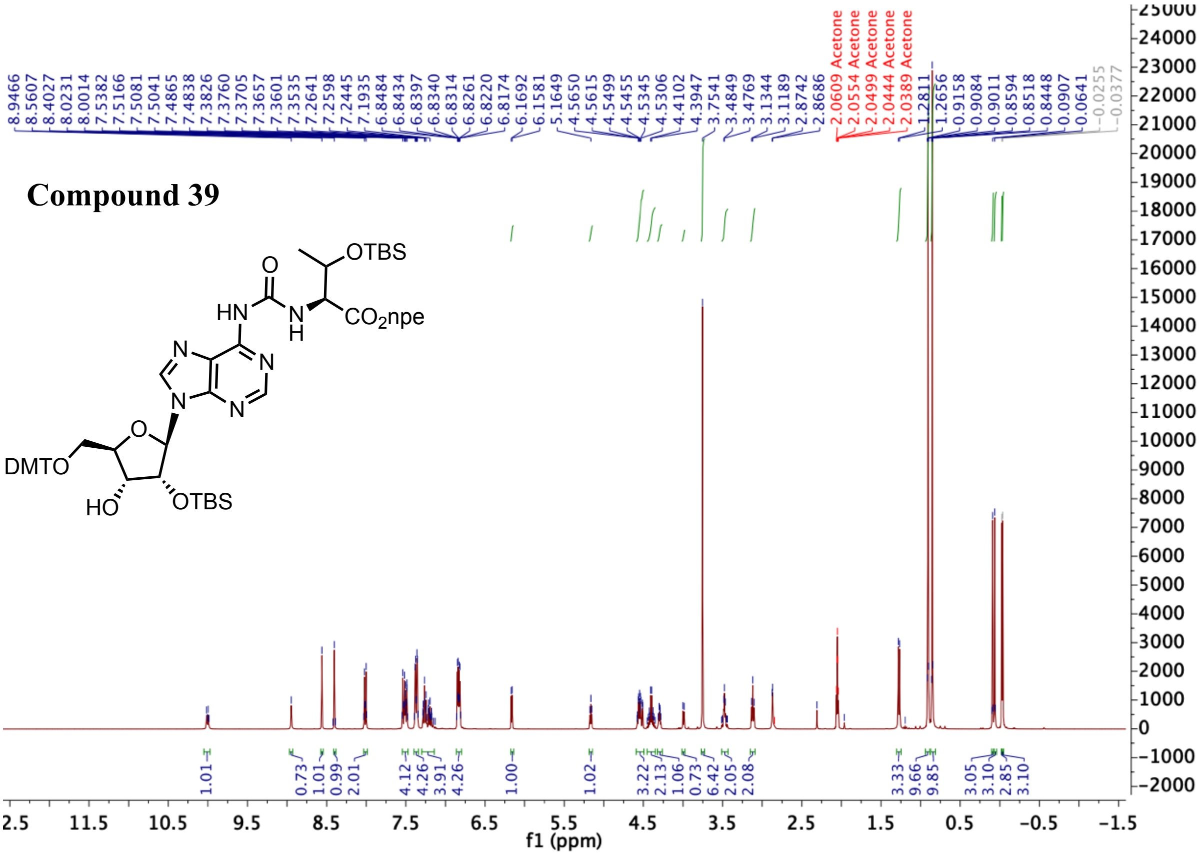

OTBS
O
HN
N
H
CO2npe
N
N
N
N
O
DMTO
HO
OTBS
8.9466 8.5607 8.4027 8.0231 8.0014 7.5382 7.5166 7.5081 7.5041 7.4865 7.4838 7.3826 7.3760 7.3705 7.3657 7.3601 7.3535 7.2641 7.2598 7.2445 7.1935 6.8484 6.8434 6.8397 6.8340 6.8314 6.8261 6.8220 6.8174 6.1692 6.1581 5.1649 4.5650 4.5615 4.5499 4.5455 4.5345 4.5306 4.4102 4.3947 3.7541 3.4849 3.4769 3.1344 3.1189 2.8742 2.8686
2.0609 Acetone
2.0554 Acetone
2.0499 Acetone
2.0444 Acetone
2.0389 Acetone
1.2811 1.2656 0.9158 0.9084 0.9011 0.8594 0.8518 0.8448 0.0907 0.0641 -0.0255 -0.0377
1.01 0.73 1.01 0.99 2.01 4.12 4.26 3.91 4.26 1.00 1.02 3.22 2.13 1.06 0.73 6.42 2.05 2.08 3.33 9.66 9.85 3.05 3.10 2.85 3.10
2.5 11.5 10.5 9.5 8.5 7.5 6.5 5.5 4.5 3.5 2.5 1.5 0.5 -0.5 -1.5
f1 (ppm)

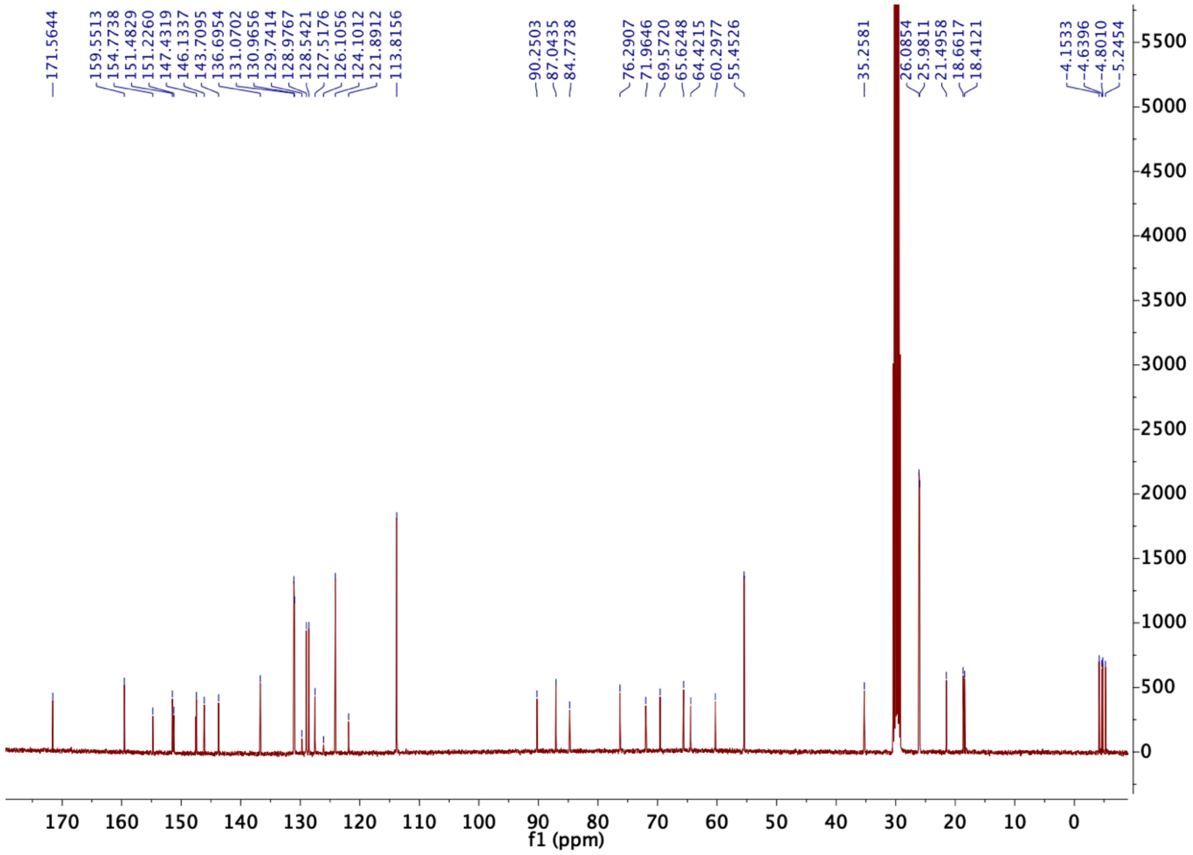

171.5644 159.5513 154.7738 151.4829 151.2260 147.4319 146.1337 143.7095 136.6954 131.0702 130.9656 129.7414 128.9767 128.5421 127.5176 126.1055 124.1012 121.8912 113.8156 90.2503 87.0435 84.7738 76.2907 71.9646 69.5720 65.6248 64.4215 60.2977 55.4526 35.2581 26.0854 25.9811 21.4958 18.6617 18.4121 -4.1533 -4.6396 -4.8010 -5.2454
170 160 150 140 130 120 110 100 90 80 70 60 50 40 30 20 10 0
f1 (ppm)

Compound 40

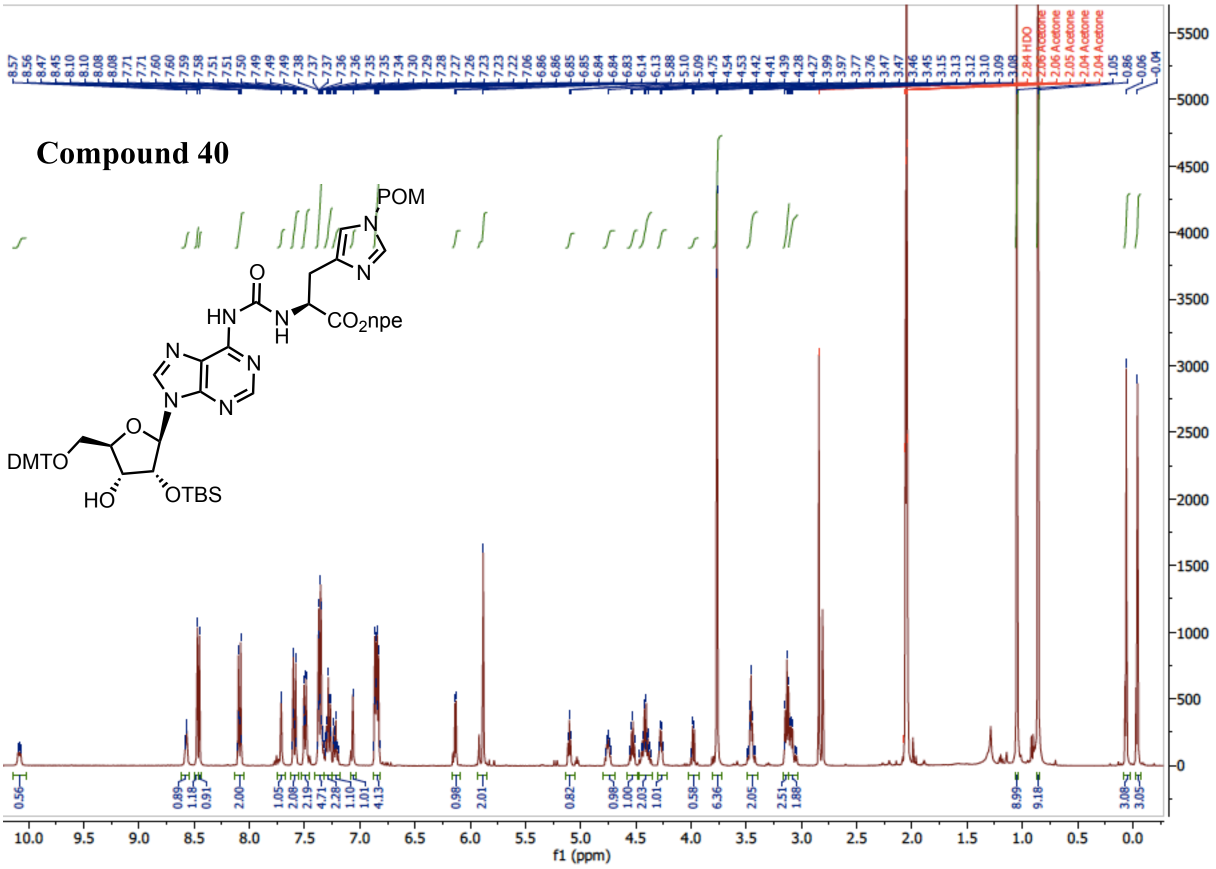

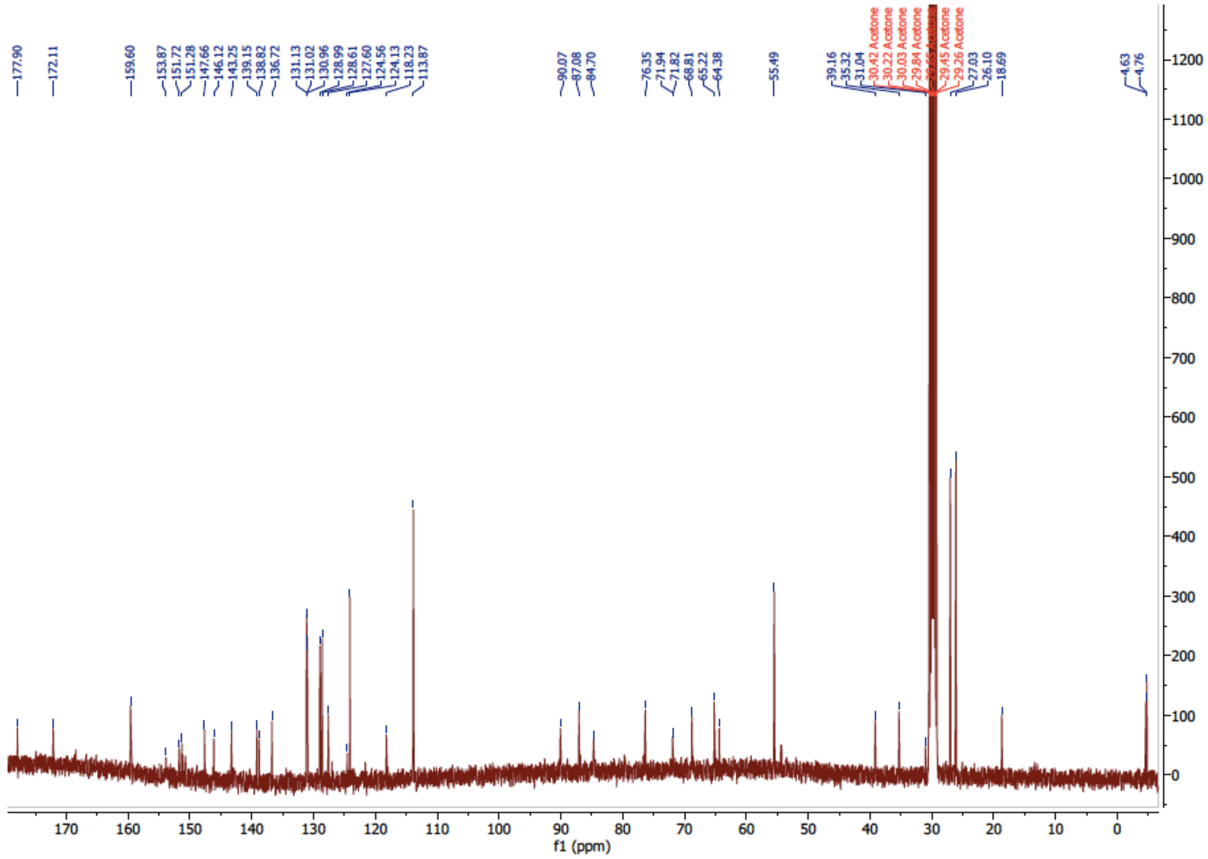

Compound 41

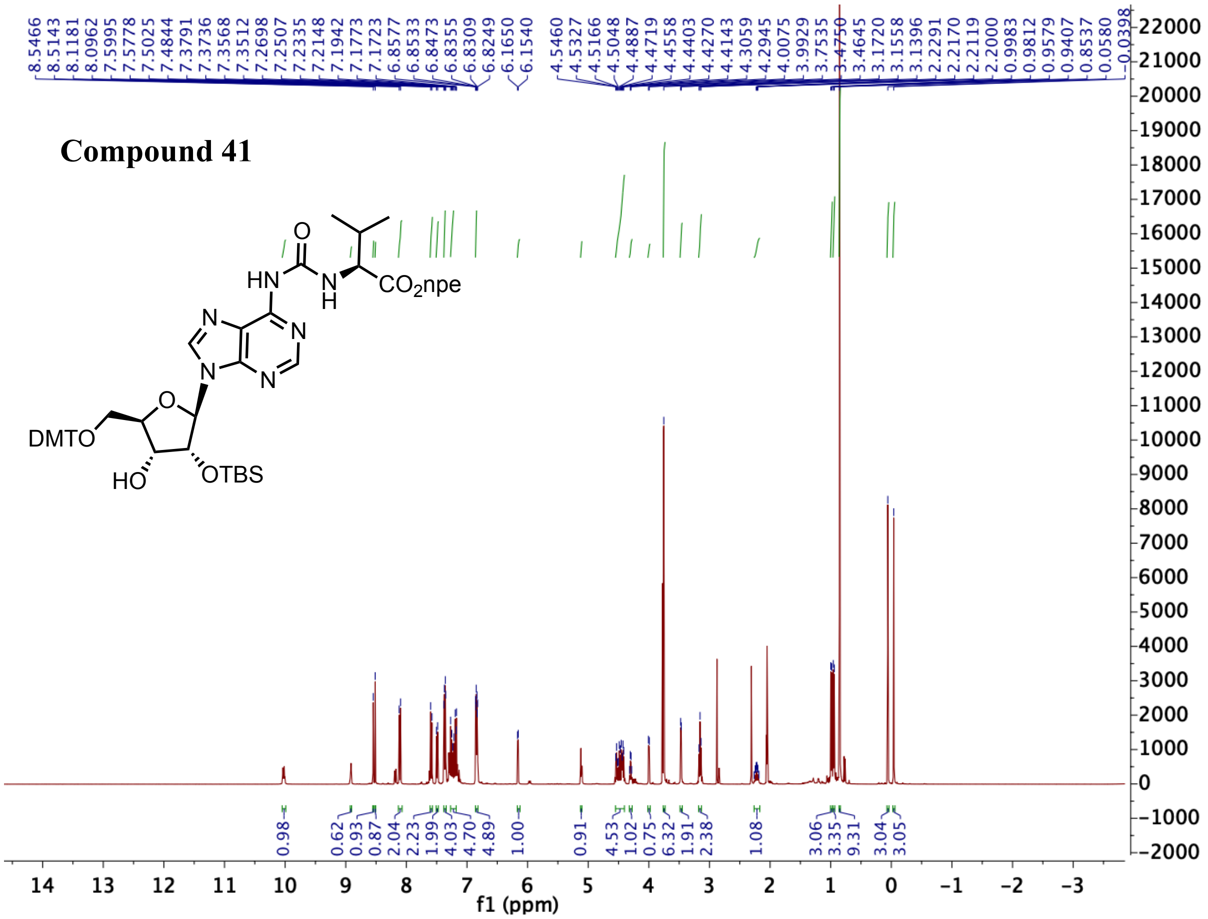
8.5466
8.5143
8.1181
8.0962
7.5995
7.5778
7.5025
7.4844
7.3791
7.3736
7.3568
7.3512
7.2698
7.2507
7.2335
7.2148
7.1942
7.1773
7.1723
6.8577
6.8533
6.8473
6.8355
6.8309
6.8249
6.1650
6.1540
4.5460
4.5327
4.5166
4.5048
4.4887
4.4719
4.4558
4.4403
4.4270
4.4143
4.3059
4.2945
4.0075
3.9929
3.7535
3.4750
3.4645
3.1720
3.1558
3.1396
2.2291
2.2170
2.2119
2.2000
0.9983
0.9812
0.9579
0.9407
0.8537
0.0580
-0.0398
HN
N
H
O
CO_2npe
N
N
N
N
N
O
DMTO
HO
OTBS
0.98
0.62
0.93
0.87
2.04
2.23
1.99
4.03
4.70
4.89
1.00
0.91
4.53
1.02
0.75
6.32
1.91
2.38
1.08
3.06
3.35
9.31
3.04
3.05
f1 (ppm)

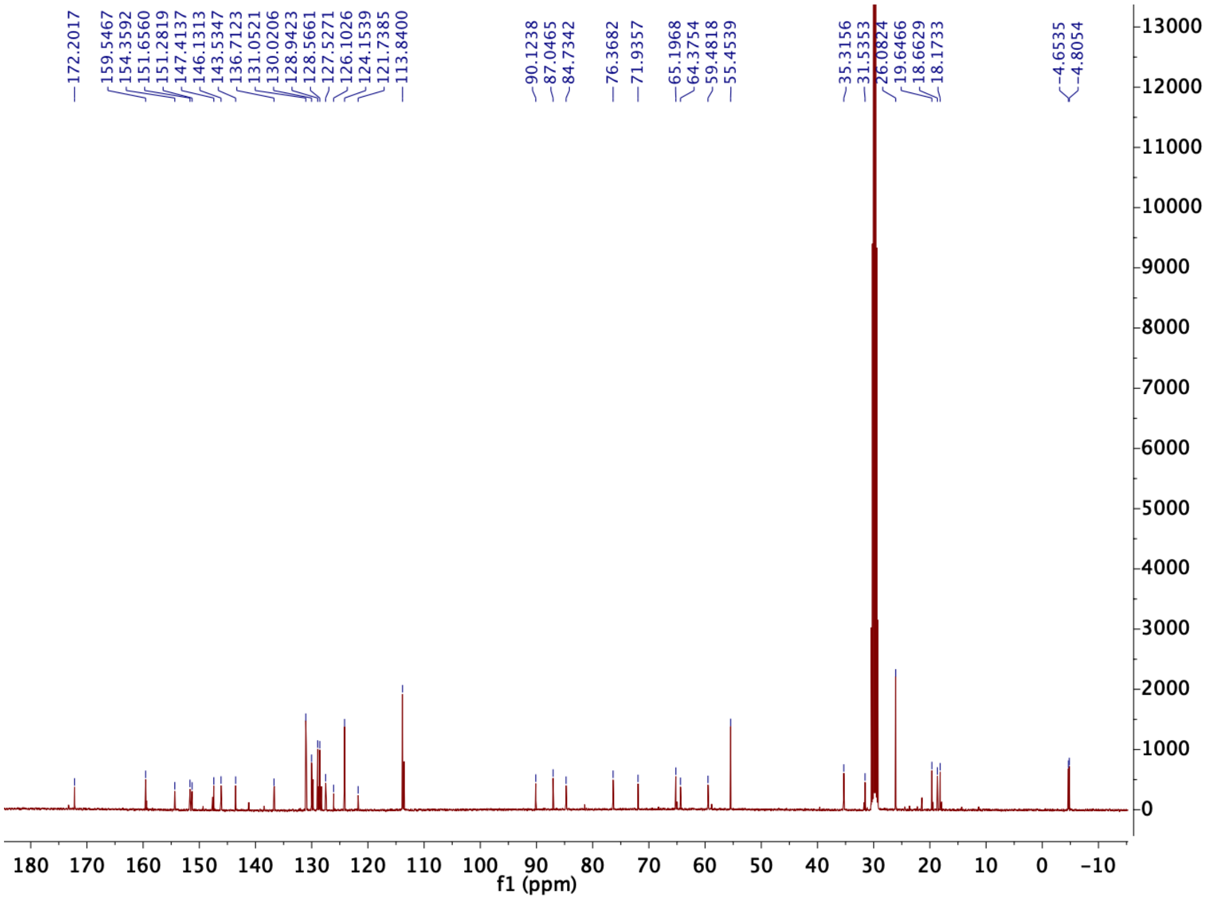
172.2017
159.5467
154.3592
151.6560
151.2819
147.4137
146.1313
143.5347
136.7123
131.0521
130.0206
128.9423
128.5661
127.5271
126.1026
124.1539
121.7385
113.8400
90.1238
87.0465
84.7342
76.3682
71.9357
65.1968
64.3754
59.4818
55.4539
35.3156
31.5353
26.0824
19.6466
18.6629
18.1733
-4.6535
-4.8054
f1 (ppm)

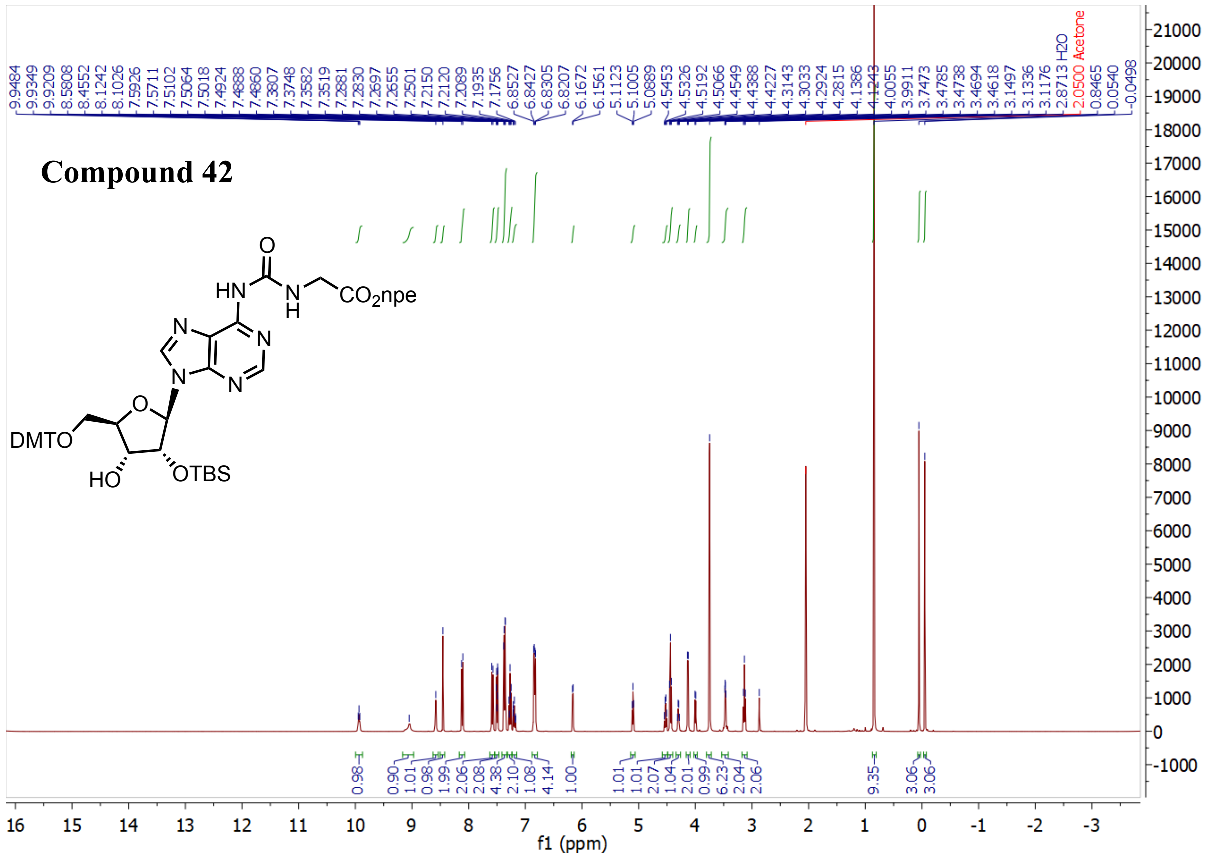
Compound 42
HN
N
H
O
CO2npe
N
N
N
N
O
DMTO
HO
OTBS
9.9484 9.9349 9.9209 8.5808 8.4552 8.1242 8.1026 7.5926 7.5711 7.5102 7.5064 7.5018 7.4924 7.4888 7.4860 7.3807 7.3748 7.3582 7.3519 7.2881 7.2830 7.2697 7.2655 7.2501 7.2150 7.2120 7.2089 7.1935 7.1756 6.8527 6.8427 6.8305 6.8207 6.1672 6.1561 5.1123 5.1005 5.0889 4.5453 4.5326 4.5192 4.5066 4.4549 4.4388 4.4227 4.3143 4.3033 4.2924 4.2815 4.1386 4.1243 4.0055 3.9911 3.7473 3.4785 3.4738 3.4694 3.4618 3.1497 3.1336 3.1176 2.8713 H2O 2.0500 Acetone 0.8465 0.0540 -0.0498
0.98 0.90 1.01 0.98 1.99 2.06 2.08 4.38 2.10 1.08 4.14 1.00 1.01 1.01 2.07 1.04 2.01 0.99 6.23 2.04 2.06 9.35 3.06 3.06
f1 (ppm)

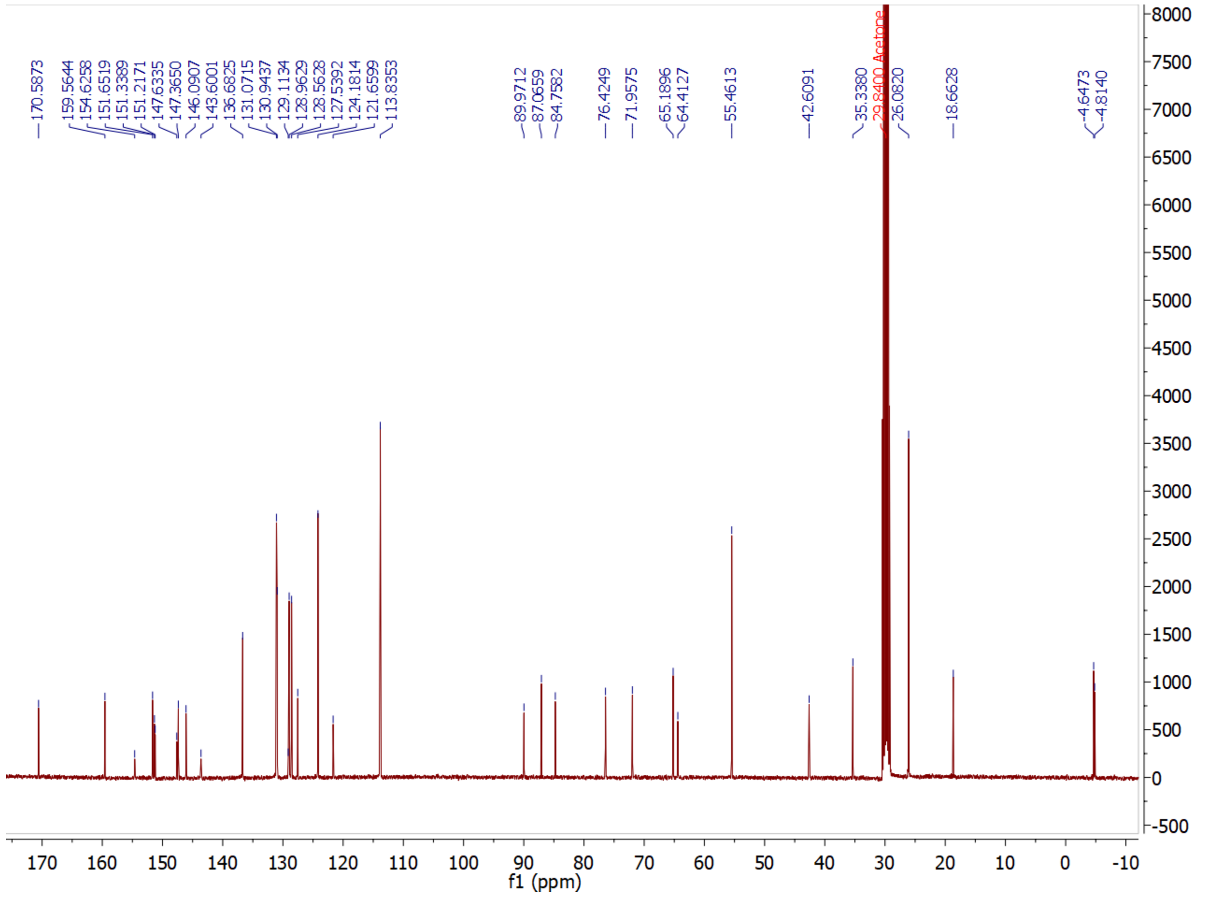
170.5873 159.5644 154.6258 151.6519 151.3389 151.2171 147.6335 147.3650 146.0907 143.6001 136.6825 131.0715 130.9437 129.1134 128.9629 128.5628 127.5392 124.1814 121.6599 113.8353 89.9712 87.0659 84.7582 76.4249 71.9575 65.1896 64.4127 55.4613 42.6091 35.3380 29.8400 Acetone 26.0820 18.6628 -4.6473 -4.8140
f1 (ppm)

Compound 43

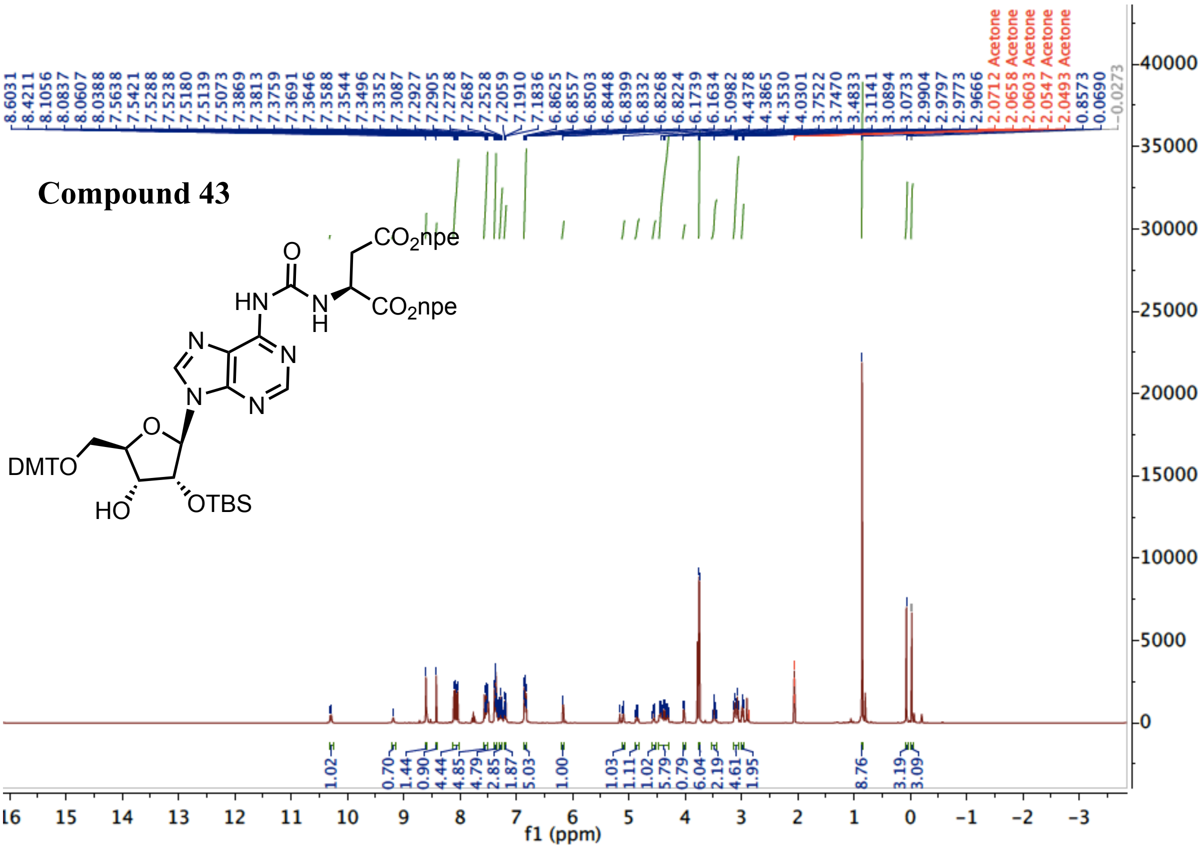

8.6031
8.4211
8.1056
8.0837
8.0607
8.0388
7.5638
7.5421
7.5288
7.5238
7.5180
7.5139
7.5073
7.3869
7.3813
7.3759
7.3691
7.3646
7.3588
7.3544
7.3496
7.3352
7.3087
7.2927
7.2905
7.2728
7.2687
7.2528
7.2059
7.1910
7.1836
6.8625
6.8557
6.8503
6.8448
6.8399
6.8332
6.8268
6.8224
6.1739
6.1634
5.0982
4.4378
4.3865
4.3530
4.0301
3.7522
3.7470
3.4833
3.1141
3.0894
3.0733
2.9904
2.9797
2.9773
2.9666
2.0712 Acetone
2.0658 Acetone
2.0603 Acetone
2.0547 Acetone
2.0493 Acetone
0.8573
0.0690
-0.0273
CO2npe
O
HN
N
H
CO2npe
N
N
N
N
O
DMTO
HO
OTBS
f1 (ppm)

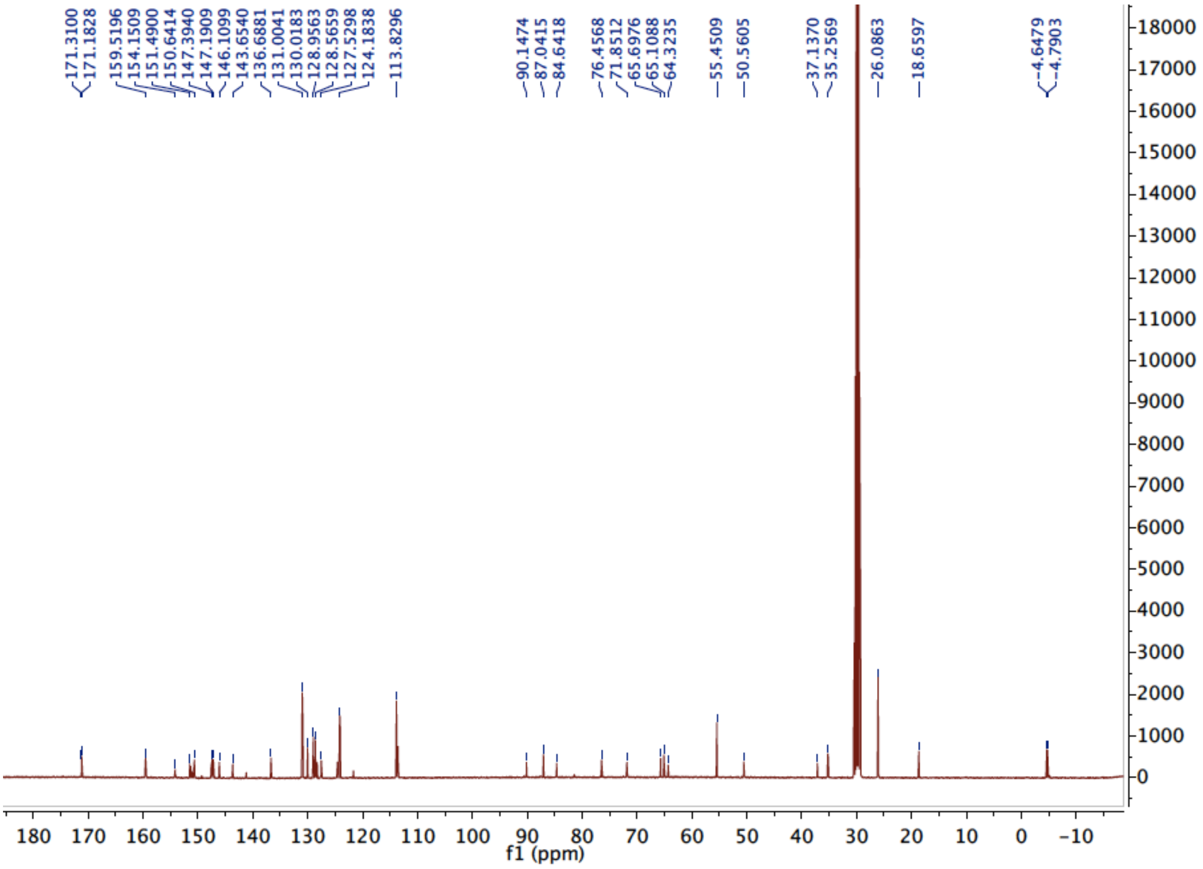

171.3100
171.1828
159.5196
154.1509
151.4900
150.6414
147.3940
147.1909
146.1099
143.6540
136.6881
131.0041
130.0183
128.9563
128.5659
127.5298
124.1838
113.8296
90.1474
87.0415
84.6418
76.4568
71.8512
65.6976
65.1088
64.3235
55.4509
50.5605
37.1370
35.2569
26.0863
18.6597
-4.6479
-4.7903
f1 (ppm)

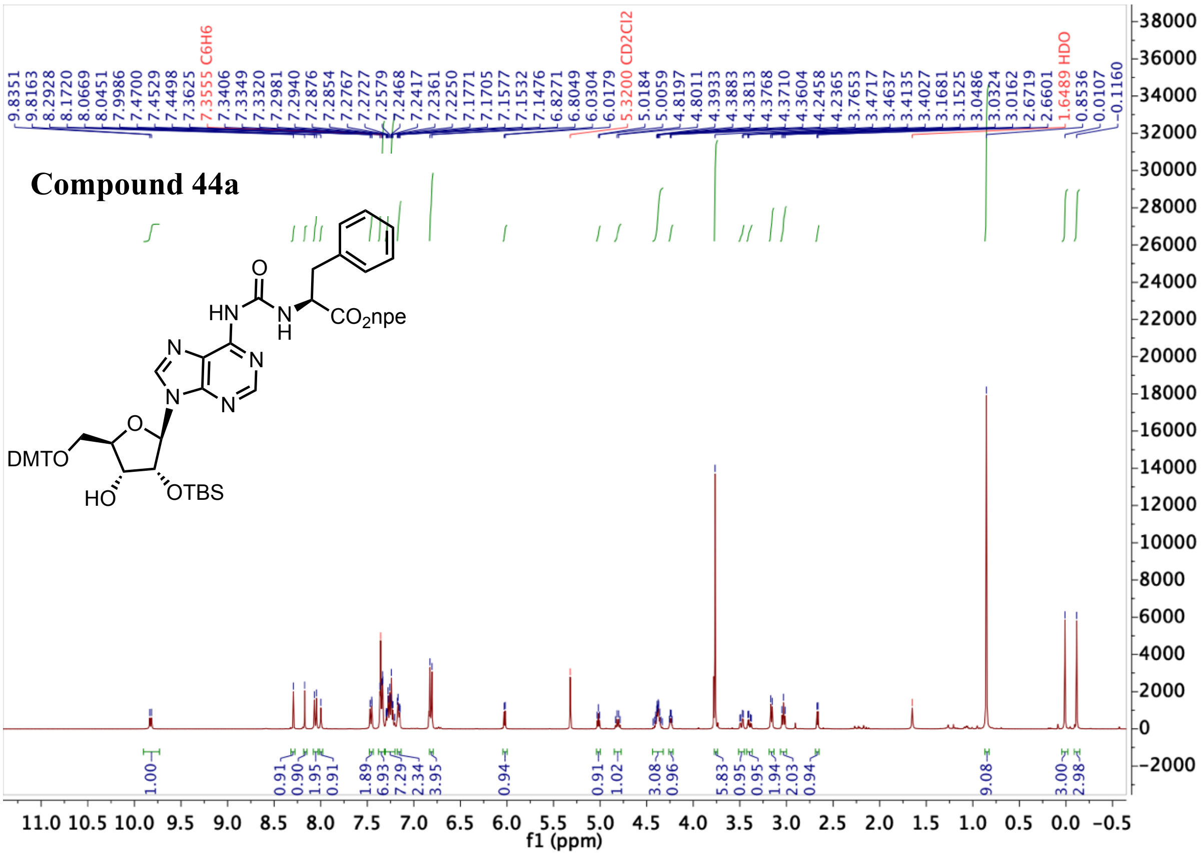

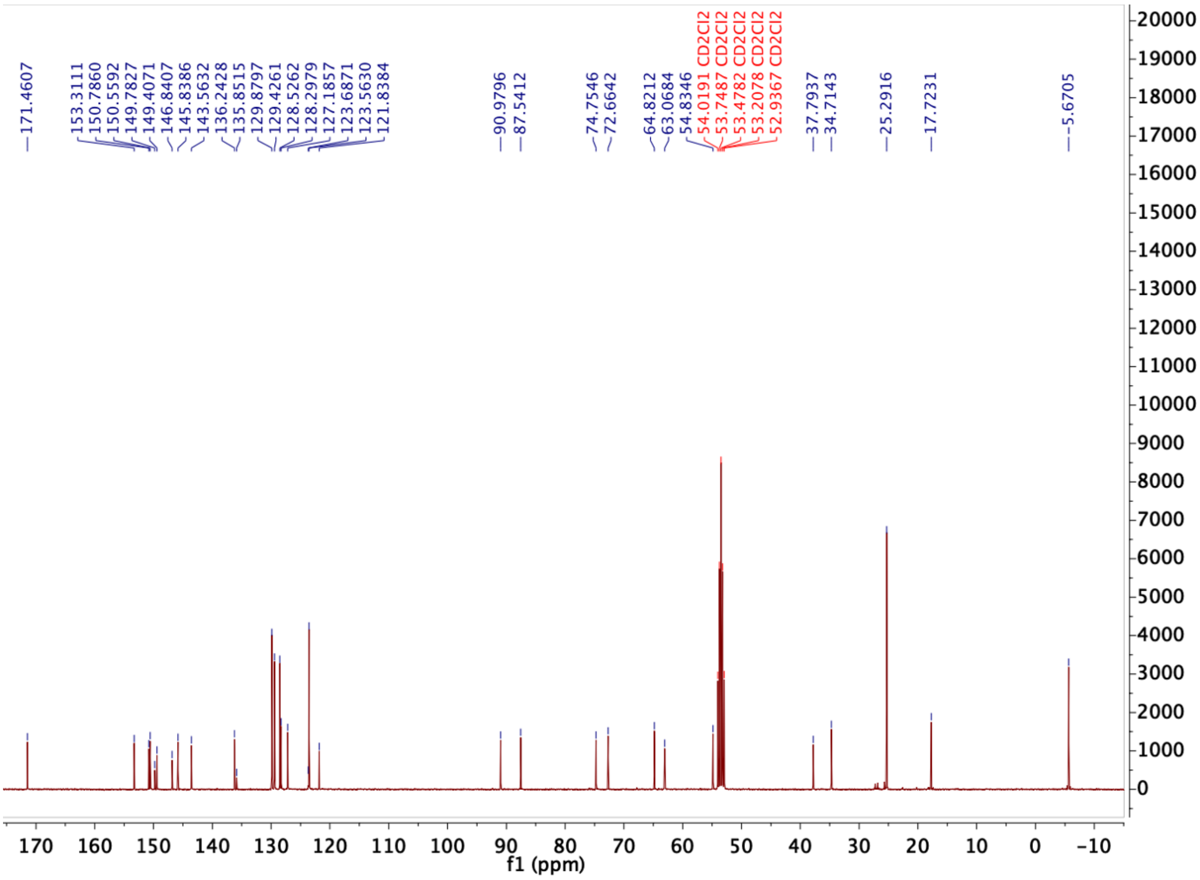

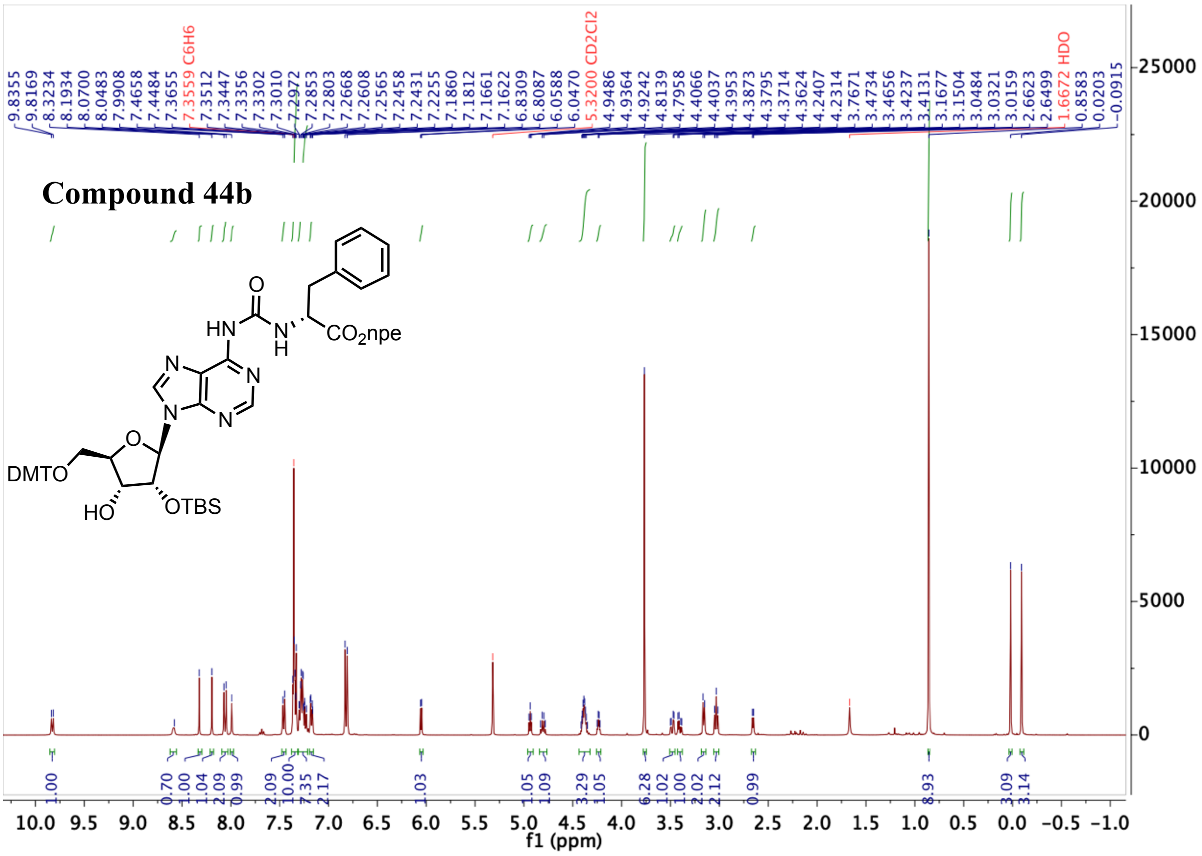

Compound 44b
9.8355
9.8169
8.3234
8.1934
8.0700
8.0483
7.9908
7.4658
7.4484
7.3655
7.3559 C6H6
7.3512
7.3447
7.3356
7.3302
7.3010
7.2972
7.2853
7.2803
7.2668
7.2608
7.2565
7.2458
7.2431
7.2255
7.1860
7.1812
7.1661
7.1622
6.8309
6.8087
6.0588
6.0470
5.3200 CD2Cl2
4.9486
4.9364
4.9242
4.8139
4.7958
4.4066
4.4037
4.3953
4.3873
4.3795
4.3714
4.3624
4.2407
4.2314
3.7671
3.4734
3.4656
3.4237
3.4131
3.1677
3.1504
3.0484
3.0321
3.0159
2.6623
2.6499
1.6672 HDO
0.8583
0.0203
-0.0915
HN
N
H
O
CO2npe
N
N
N
N
O
DMTO
HO
OTBS
1.00
0.70
1.00
1.04
2.09
0.99
2.09
10.00
7.35
2.17
1.03
1.05
1.09
3.29
1.05
6.28
1.02
1.00
2.02
2.12
0.99
8.93
3.09
3.14
f1 (ppm)

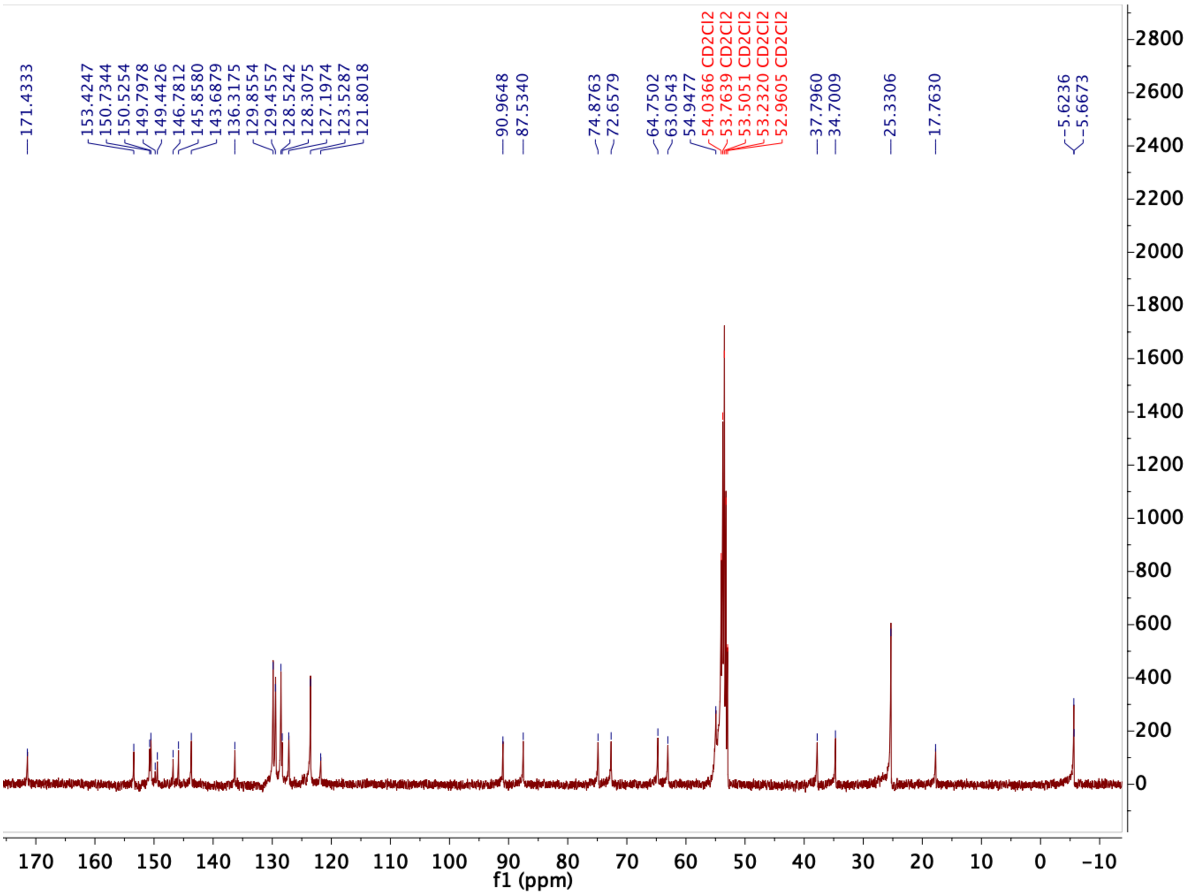

171.4333
153.4247
150.7344
150.5254
149.7978
149.4426
146.7812
145.8580
143.6879
136.3175
129.8554
129.4557
128.5242
128.3075
127.1974
123.5287
121.8018
90.9648
87.5340
74.8763
72.6579
64.7502
63.0543
54.9477
54.0366 CD2Cl2
53.7639 CD2Cl2
53.5051 CD2Cl2
53.2320 CD2Cl2
52.9605 CD2Cl2
37.7960
34.7009
25.3306
17.7630
-5.6236
-5.6673
f1 (ppm)

Compound 45

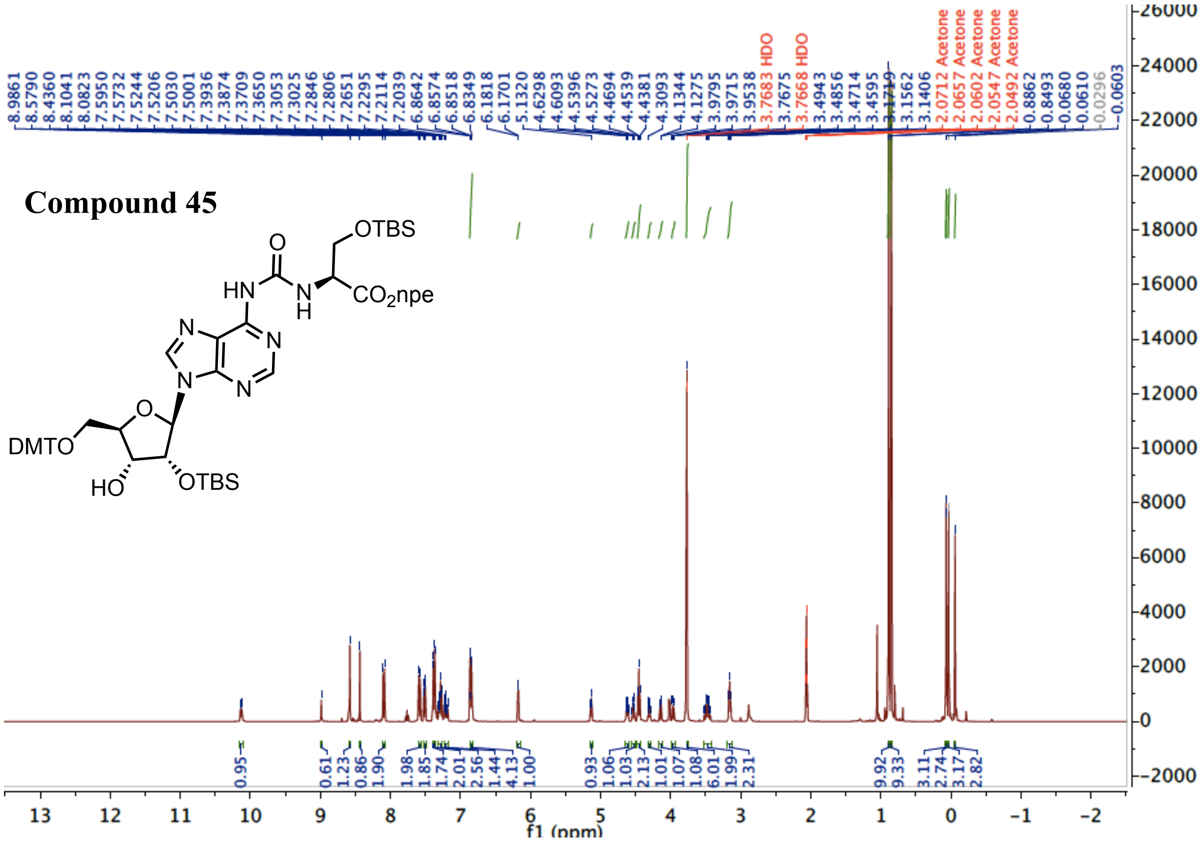

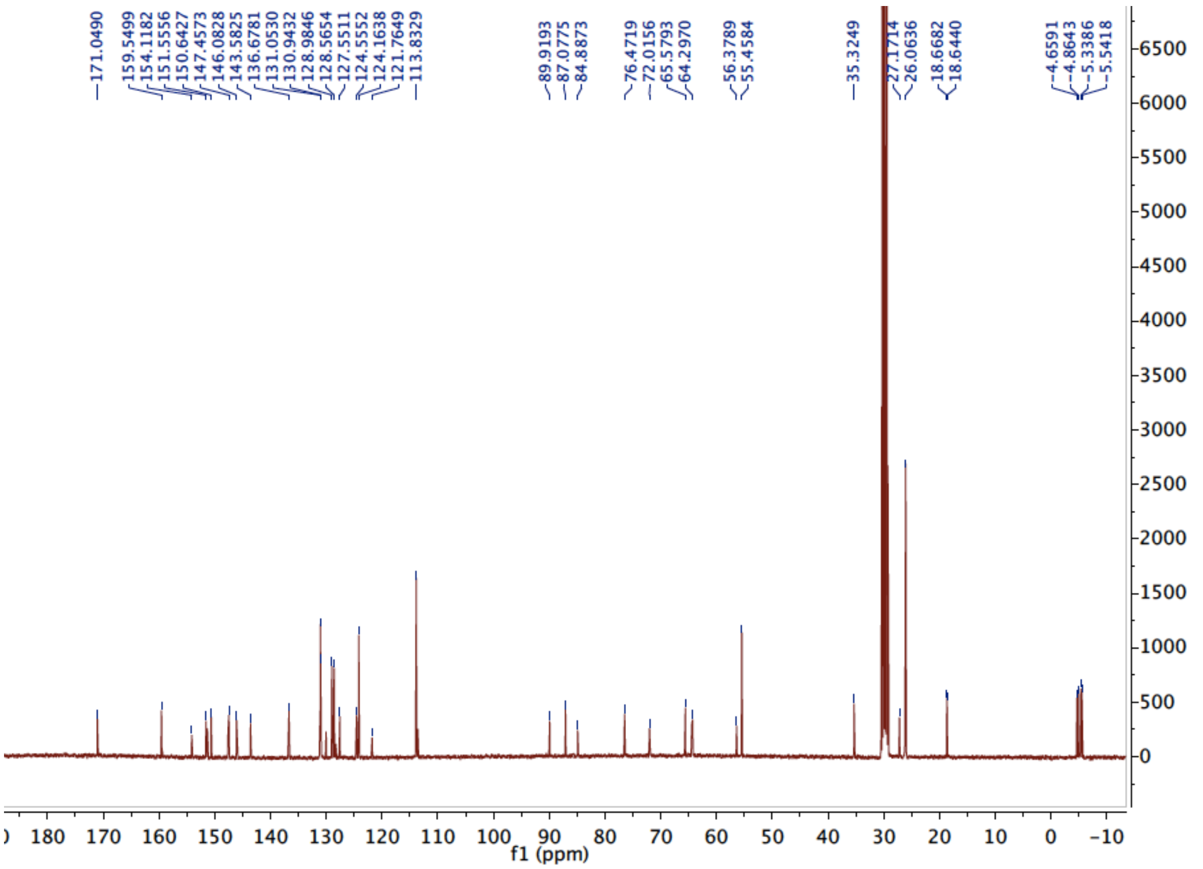

Compound 46

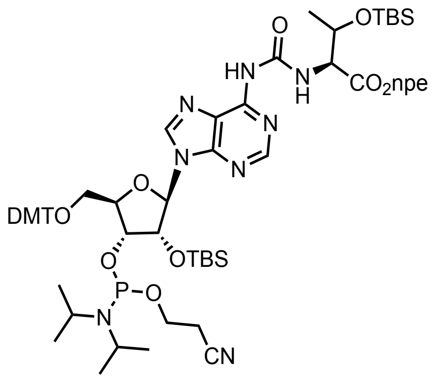

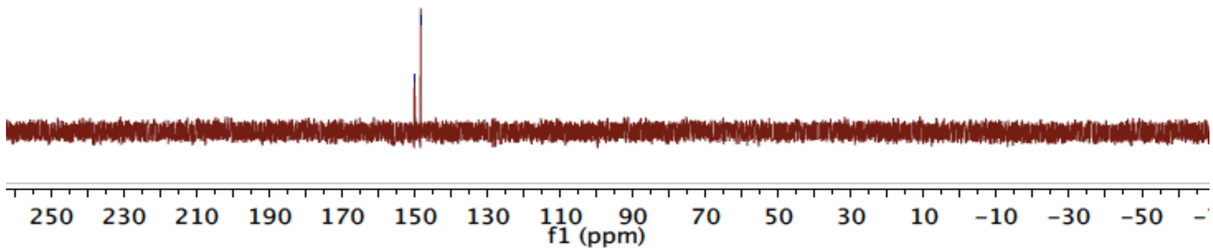

Compound 47

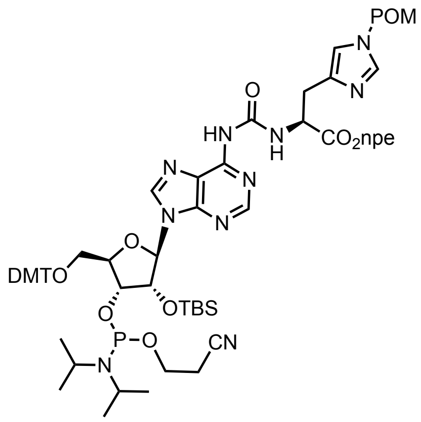

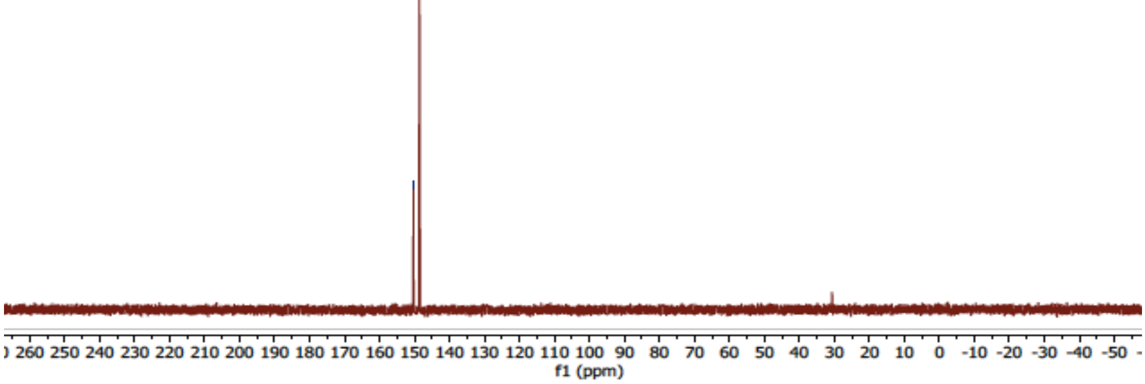

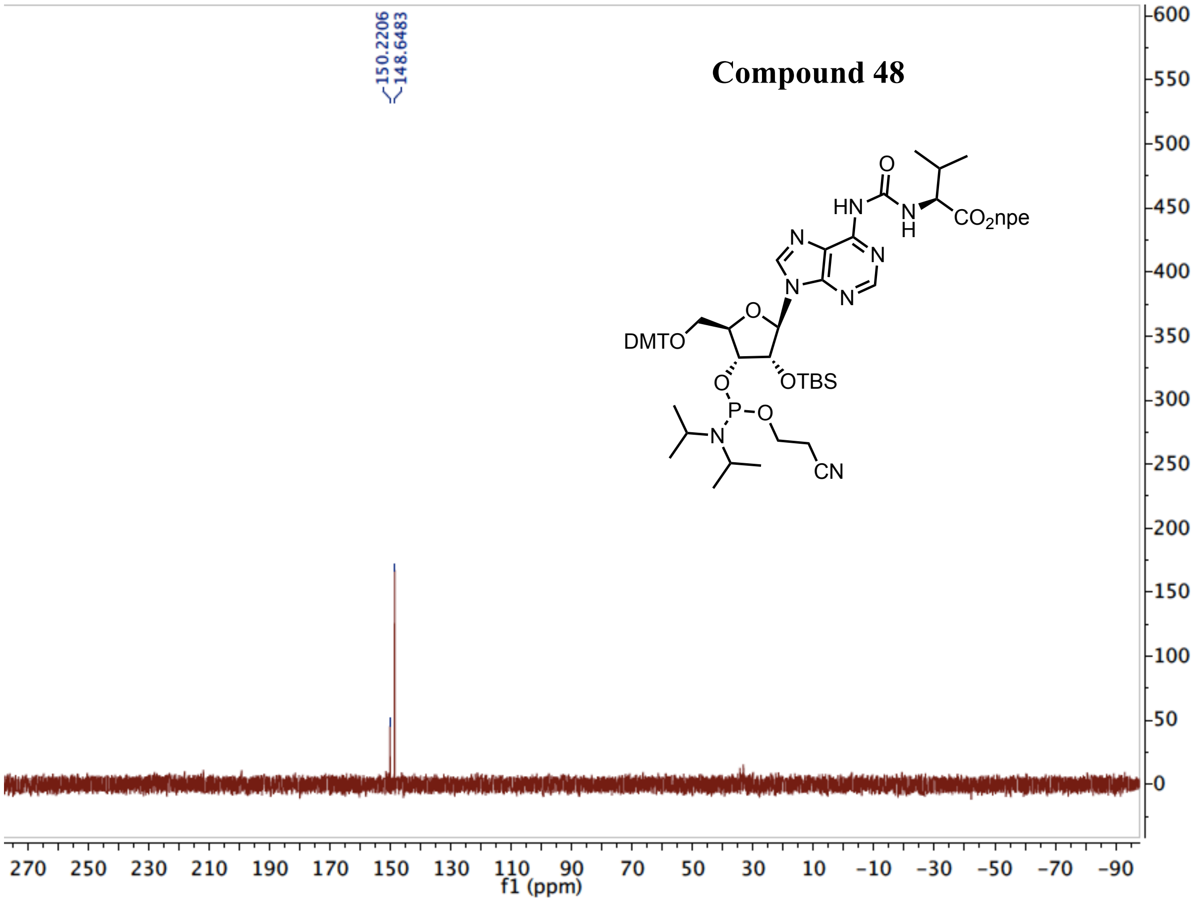
150.2206
148.6483
Compound 48
DMTO
OTBS
CN
CO2npe
f1 (ppm)

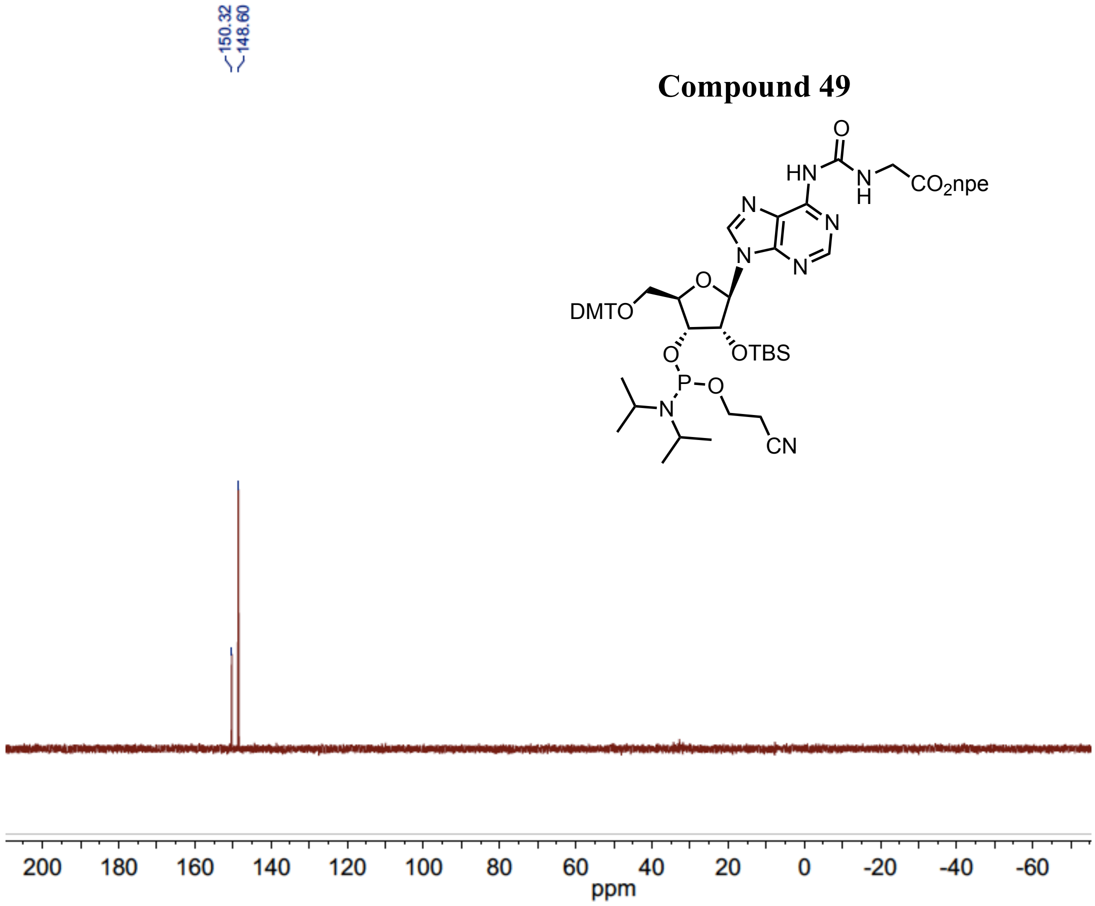
150.32
148.60
Compound 49
DMTO
OTBS
CN
CO2npe
ppm

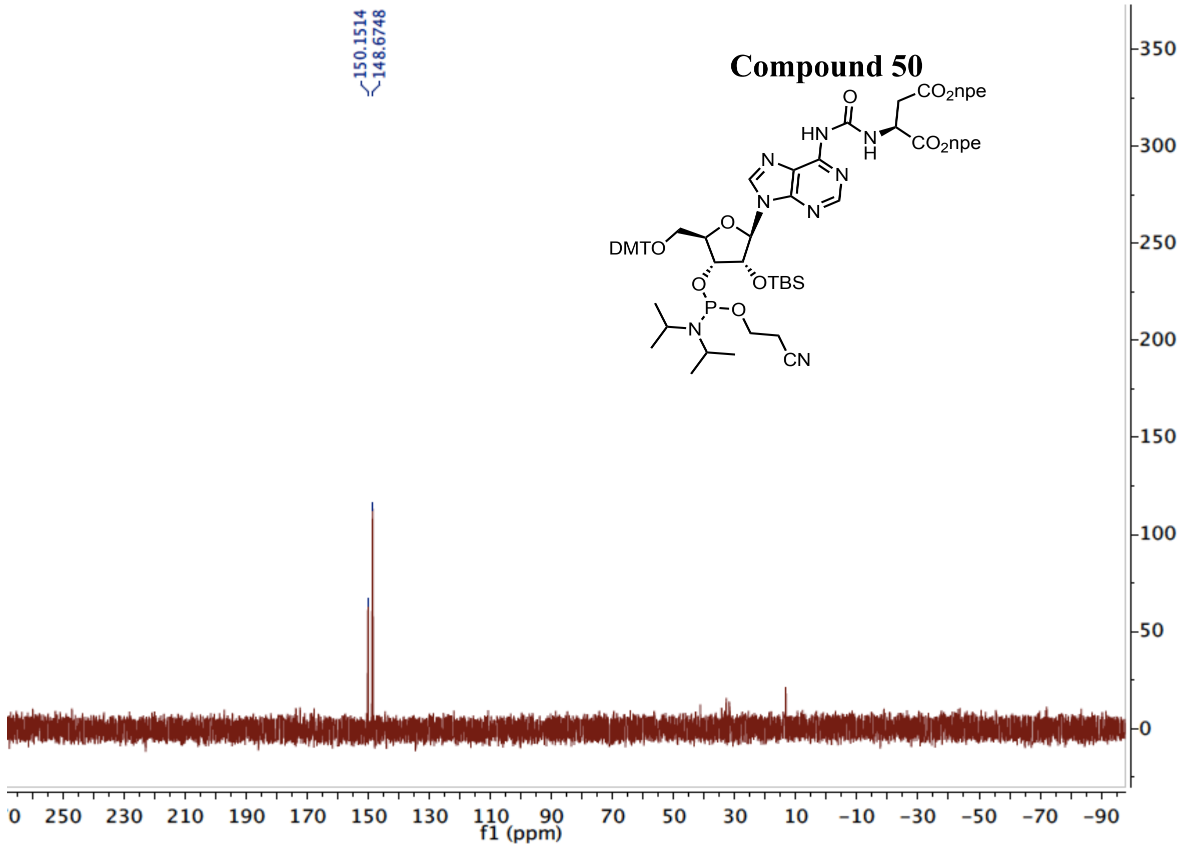
150.1514
148.6748
Compound 50
CO2npe
CO2npe
HN
N
H
O
N
N
N
N
O
DMTO
O
OTBS
P
O
N
CN
f1 (ppm)

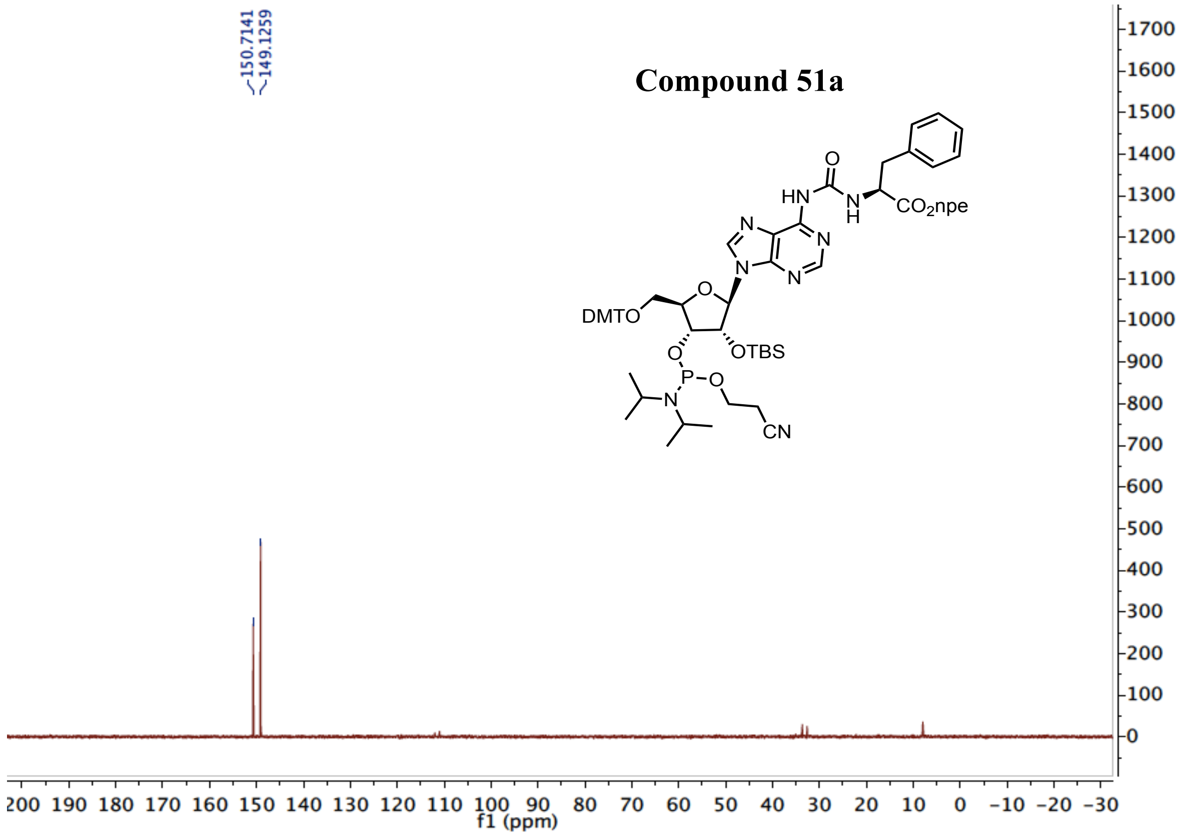
150.7141
149.1259
Compound 51a
CO2npe
HN
N
H
O
DMTO
OTBS
P
N
CN
f1 (ppm)

Compound 51b

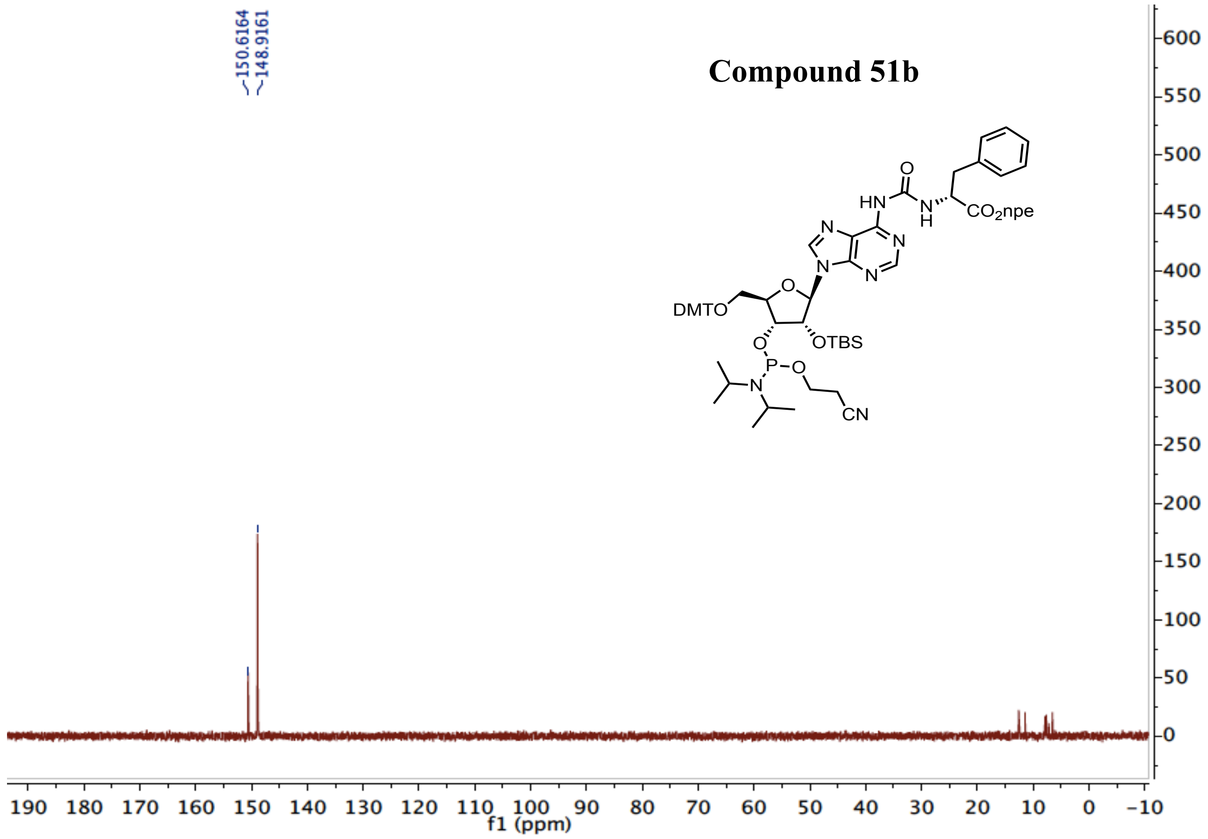

Compound 52

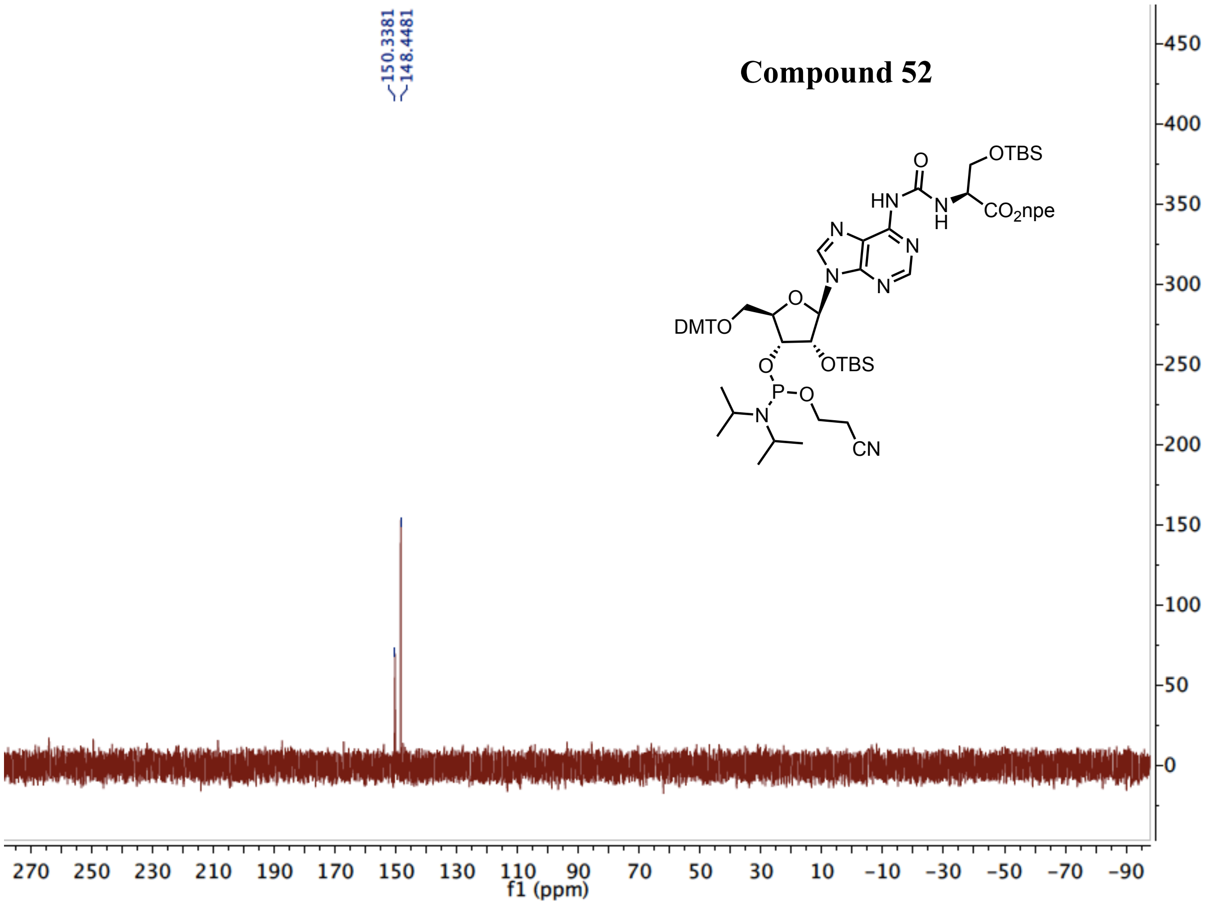

Compound 55

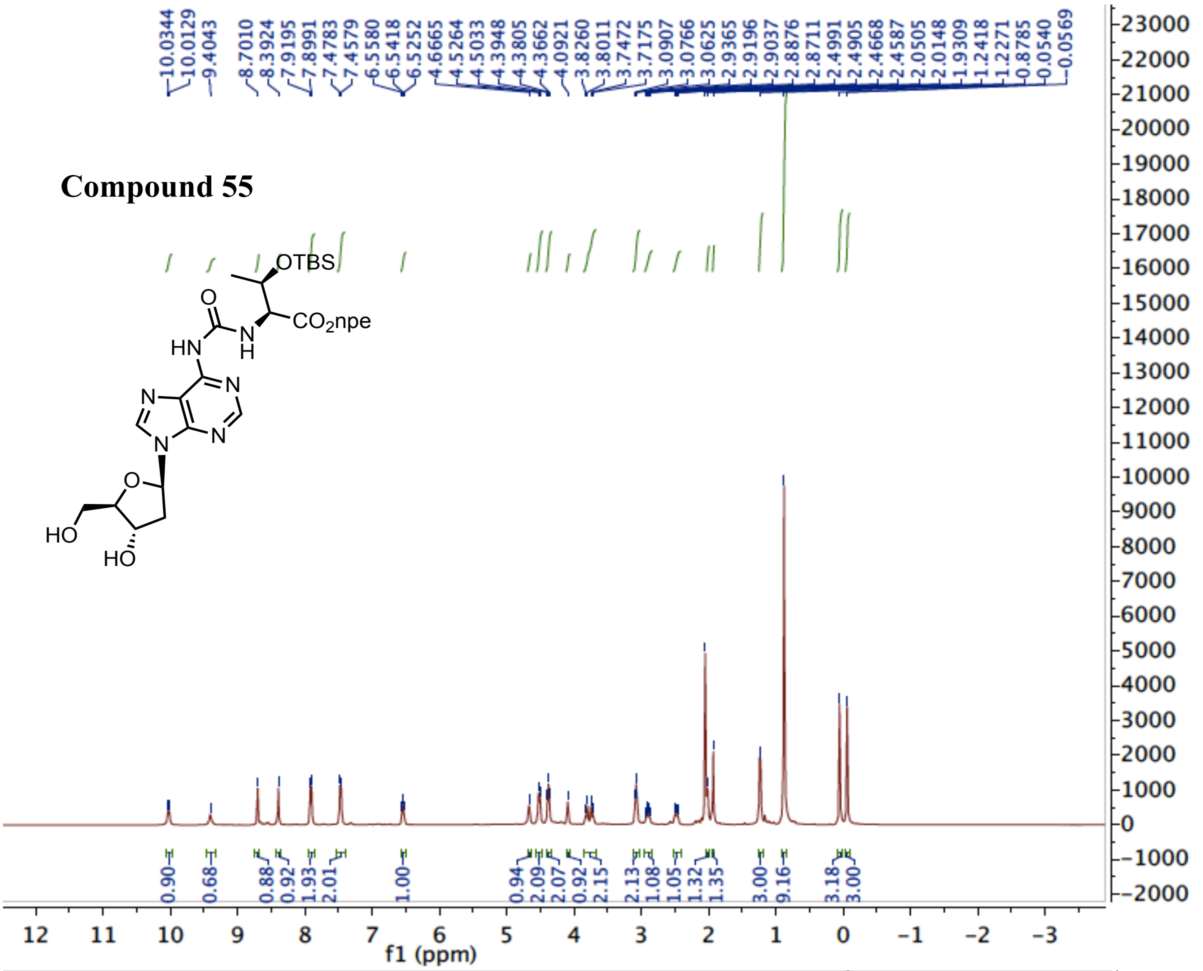
10.0344
10.0129
9.4043
8.7010
8.3924
7.9195
7.8991
7.4783
7.4579
6.5580
6.5418
6.5252
4.6665
4.5264
4.5033
4.3948
4.3805
4.3662
4.0921
3.8260
3.8011
3.7472
3.7175
3.0907
3.0766
3.0625
2.9365
2.9196
2.9037
2.8876
2.8711
2.4991
2.4905
2.4668
2.4587
2.0505
2.0148
1.9309
1.2418
1.2271
0.8785
0.0540
-0.0569
OTBS
CO2npe
O
N
H
HN
N
N
N
N
O
HO
HO
0.90
0.68
0.88
0.92
1.93
2.01
1.00
0.94
2.09
2.07
0.92
2.15
2.13
1.08
1.05
1.32
1.35
3.00
9.16
3.18
3.00
f1 (ppm)

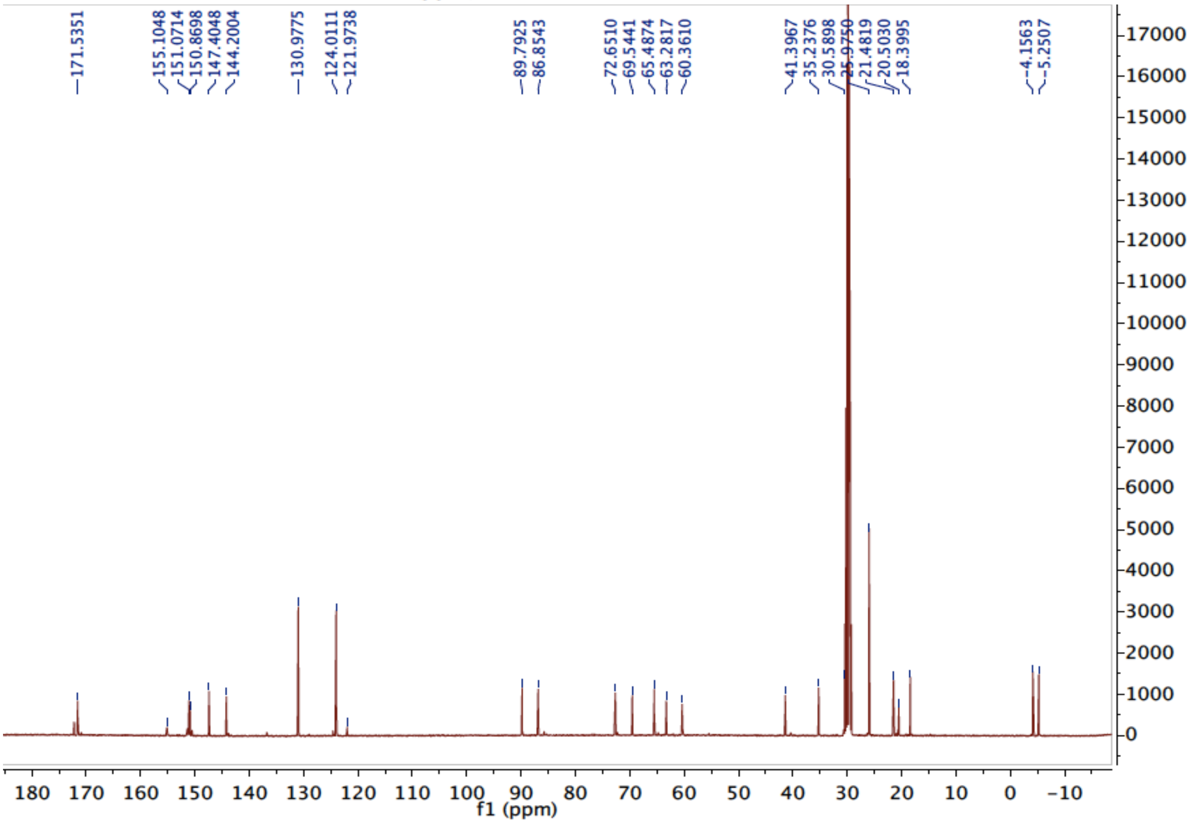
171.5351
155.1048
151.0714
150.8698
147.4048
144.2004
130.9775
124.0111
121.9738
89.7925
86.8543
72.6510
69.5441
65.4874
63.2817
60.3610
41.3967
35.2376
30.5898
21.4819
20.5030
18.3995
-4.1563
-5.2507
f1 (ppm)

Compound 56

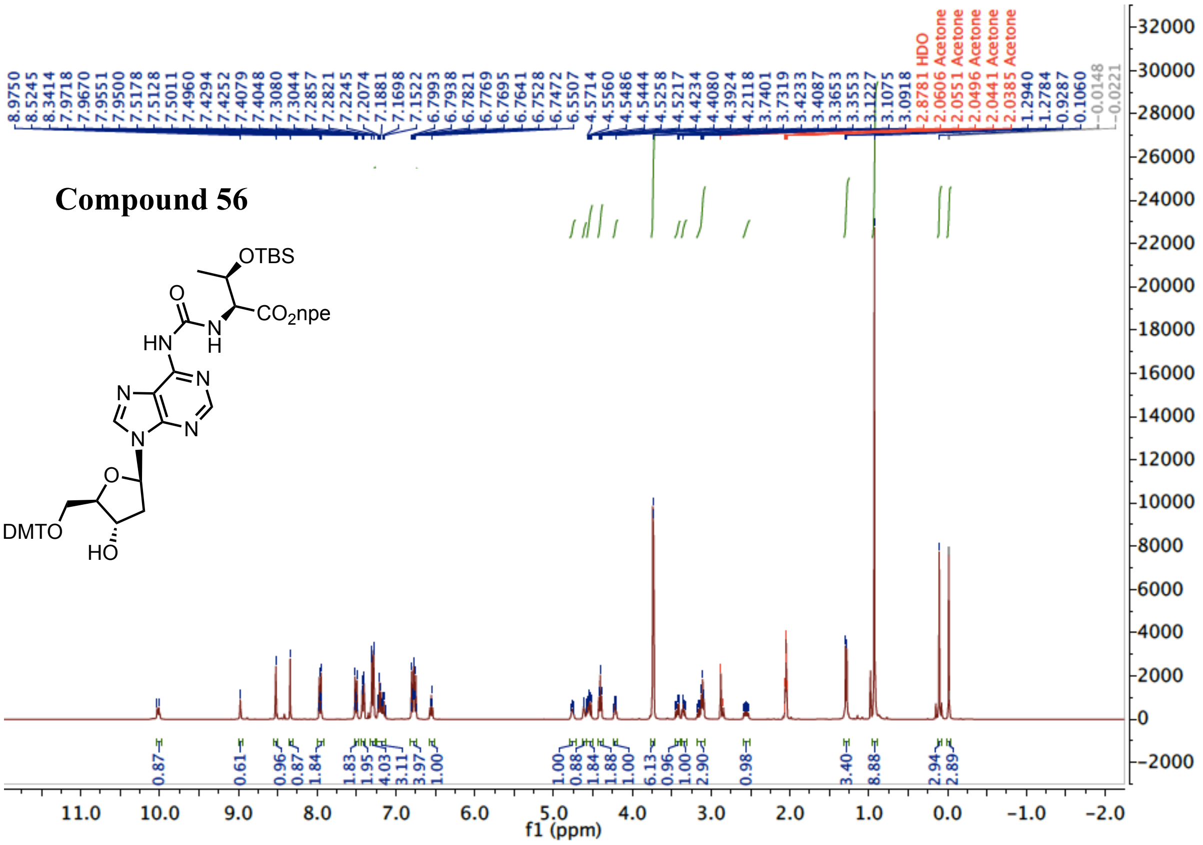
8.9750
8.5245
8.3414
7.9718
7.9670
7.9551
7.9500
7.5178
7.5128
7.5011
7.4960
7.4294
7.4252
7.4079
7.4048
7.3080
7.3044
7.2857
7.2821
7.2245
7.2074
7.1881
7.1698
7.1522
6.7993
6.7938
6.7821
6.7769
6.7695
6.7641
6.7528
6.7472
6.5507
4.5714
4.5560
4.5486
4.5444
4.5258
4.5217
4.4234
4.4080
4.3924
4.2118
3.7401
3.7319
3.4233
3.4087
3.3653
3.3553
3.1227
3.1075
3.0918
2.8781 HDO
2.0606 Acetone
2.0551 Acetone
2.0496 Acetone
2.0441 Acetone
2.0385 Acetone
1.2940
1.2784
0.9287
0.1060
-0.0148
-0.0221
OTBS
O
N
H
CO2npe
HN
N
N
N
N
O
DMTO
HO
0.87
0.61
0.96
0.87
1.84
1.83
1.95
4.03
3.11
3.97
1.00
1.00
0.88
1.84
1.88
1.00
6.13
0.96
1.00
2.90
0.98
3.40
8.88
2.94
2.89
f1 (ppm)

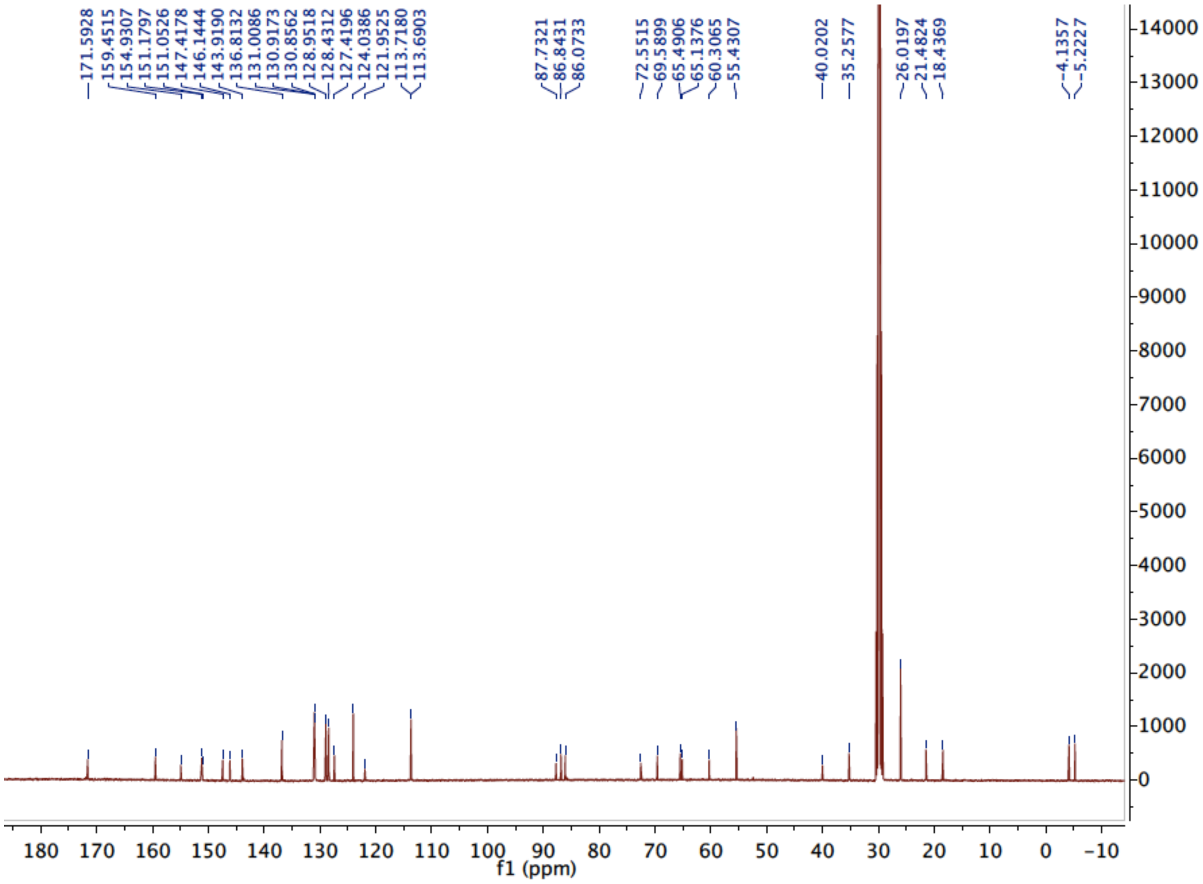
171.5928
159.4515
154.9307
151.1797
151.0526
147.4178
146.1444
143.9190
136.8132
131.0086
130.9173
130.8562
128.9518
128.4312
127.4196
124.0386
121.9525
113.7180
113.6903
87.7321
86.8431
86.0733
72.5515
69.5899
65.4906
65.1376
60.3065
55.4307
40.0202
35.2577
26.0197
21.4824
18.4369
-4.1357
-5.2227
f1 (ppm)

Compound 57

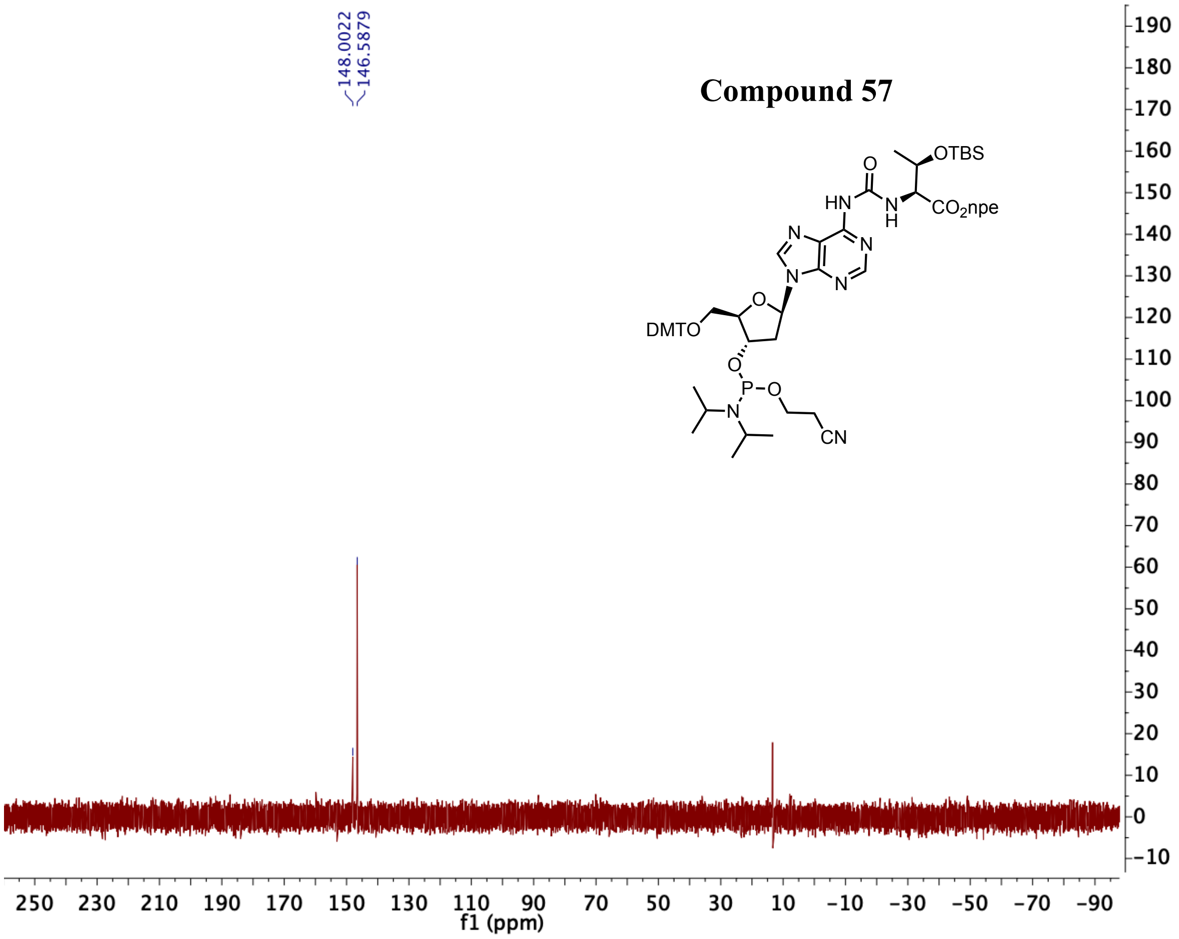

6. References

[1] V. Boudou, J. Langridge, A. Van Aerschot, C. Hendrix, A. Millar, P. Weiss, P. Herdewijn, *Helvetica Chimica Acta* **2000**, *83*, 152 – 161.

[2] G. Lezczynska, P. Leonczak, A. Dziergowska, A. Malkiewicz, *Nucleosides, Nucleotides and Nucleic Acids* **2013**, *32*, 599 – 616.

[3] F. Himmelsbach, B. S. Schultz, T. Trichtinger, R. Charubala, W. Pfleiderer, *Tetrahedron* **1984**, *40*, 59 – 72.

[4] V. Serebryany, L. Beigelman, *Tetrahedron Lett*. **2002**, *43*, 1983 – 1985.

[5] E. M. van der Wenden, J. K. von Frijtag Drabbe Künzel, R. A. A. Mathot, M. Danhof, A. P. IJzerman, W. Soudijn, *J. Med. Chem.* **1995**, *38*, 4000-4006.
